# NHC-Cu Three-Coordinate Complex as a Promising Photocatalyst for Energy and Electron Transfer Reactions

**DOI:** 10.1021/acs.joc.4c00450

**Published:** 2024-06-03

**Authors:** Krzysztof Grudzień, Zuzanna Szeptuch, Hubert Kubiszewski, Wojciech Chaładaj, Katarzyna Rybicka-Jasińska

**Affiliations:** † Institute of Organic Chemistry, Polish Academy of Sciences, Kasprzaka 44/52, Warsaw 01-224, Poland; ‡ Faculty of Chemistry, Warsaw University of Technology, Noakowskiego 3, Warsaw 00-664, Poland; § Faculty of Medicine, Medical University of Warsaw, Żwirki i Wigury 61, Warsaw 02-091, Poland

## Abstract

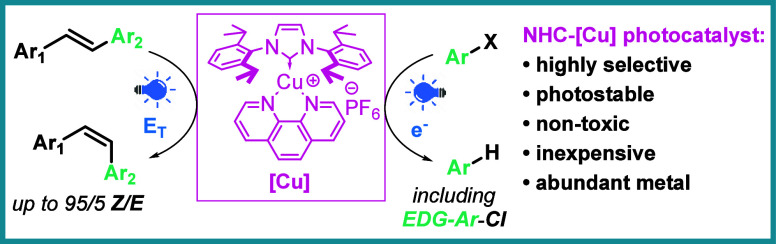

Herein, we describe a simple three-coordinate complex of Cu(I) with an NHC and 1,10-phenanthroline ligands as an effective photocatalyst for energy (e.g., olefin *E*/*Z* isomerization) and electron transfer (e.g., aryl halide dehalogenation) reactions under blue-light irradiation. This complex can be obtained in a one-pot procedure starting from commercially available reagents and green solvents (EtOH, water). We hereby present a study of its activity and mechanistic insight into its mode of operation.

## Introduction

At this current time, there is an intense search for alternative and easily renewable energy sources, of which visible light has quickly become a powerful tool for organic synthesis and drug discovery.^1−3^ The most typical and comprehensive photocatalysts, in terms of their portfolio of catalyzed transformations, are low-spin ruthenium and iridium complexes. A major drawback is their high price, resulting from low natural abundance and toxicity, which translates into limited usefulness in large-scale industrial (i.e., pharmaceutical) applications. Among the known available alternatives, the most promising and thoroughly studied in recent years are based on copper.^4−9^ McMillin et al.^10^ and Meyer et al.’s^11^ pioneering research on tetracoordinate bisphenanthroline Cu[I] complexes laid the foundations for the family of homoleptic complexes with the schematic structure [Cu(N^N)2]+, which are widely used in photocatalysis.^9^ An important note is that due to steric and electronic factors, the excited-state lifetimes and redox potentials for simple nonsubstituted complexes are very limited. Several strategies were developed to overcome this issue, including the application of heteroleptic Cu[I] complexes.^9^ In order to maximize the potential of those catalysts there should be no dynamic exchange of ligands in solution, which is ensured by the use of certain biphosphines (such as Xantphos)^12^ or bisisonitriles.^13^

**Figure 1:**
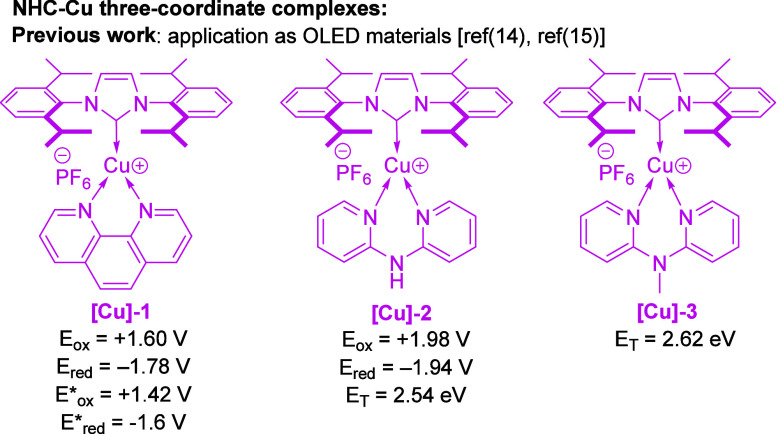
Selected photoactive copper complexes with their respective ground-state redox potentials (*E*_ox_ and *E*_red_, vs SCE)^14^ and experimental emission energies (as triplet energy estimation ~ E_T_)^15^

In 2014, Linares et al. showed photophysical and electrochemical properties of a series of new three-coordinate, heteroleptic NHC-Cu[I] complexes containing bidentate N^N ligands (Figure 1).^14,15^ In general, these compounds absorb light in the blue and near UV range, possessing high redox potentials for both reduction and oxidation and, for some of them, long lifetimes in the excited state as well as high triplet energies (Figure 1). Even though these properties make the reported NHC-Cu(I) complexes perfectly suited for the application in photocatalysis, their use as such has never been explored. Therefore, considering the photophysical properties of reported NHC-Cu(I) complexes along with the fact that they are straightforward to synthesize, nontoxic, and inexpensive, we envisioned that their application as photocatalysts might lead to the development of a general alternative for Ir- and Ru-based complexes.

## Results and Discussion

### Energy Transfer

To prove our hypothesis, we decided to check their photocatalytic activity in both energy and electron transfer reactions. Since *E* → *Z* photoisomerization of olefins was already documented in Cu(I) photochemistry,^12^ we decided to check the effectiveness of NHC-Cu(I) photocatalysts as potential photosensitizers in this type of transformation. The synthesis of **[Cu]-1**, following literature procedures as well as our newly developed one-pot, green protocol, is presented in the SI. We initiated our studies by exploring the reactivity of *trans*-stilbene (**1-***E*, *E*_T_ = 214.3 kJ/mol, *E*_1/2_ = 1.48 V vs Ag/AgCl) under the action of **[Cu]-1**. The starting conditions were based on previous protocols for stilbene isomerization.^16,17^ The reaction produced the desired *cis-*isomer **1-***Z* in an 86/14 ratio (Table 1, entry 1). Subsequently, several reaction parameters (catalyst loading, light source, concentration, and time) were optimized (Table 1). Overall, photoisomerization of **1-***E* under the action of **[Cu]-1** at room temperature was complete in only 4 h and lead to the *Z/E*-mixture in a 95/5 ratio (much faster rate compared to previous protocols^16,17^ that typically require overnight irradiation). Control experiments confirmed that the desired transformation cannot take place in absence of light (entry 13) or a photocatalyst (entry 14).

**Table 1:**
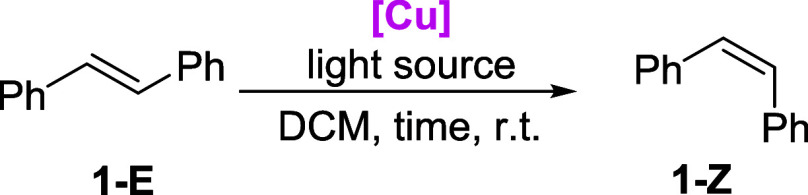
Optimization of Reaction Conditions—Photoisomerization *E***→***Z*^a^

entry	light source^e^ (power)	time [h]	*Z*/*E* [%]^f^
1^a^	405 nm (12.5 W)	18	86/14
2^a^	450 nm (12.5 W)	18	94/6
3^b^	450 nm (12.5 W)	18	95/5
4^b^^,^^c^	450 nm (12.5 W)	18	95/5
5^b^	450 nm (12.5 W)	6	95/5
**6**^b^	450 nm (12.5 W)	**4**	95/5
7^b^	450 nm (12.5 W)	2	94/6
8^b^	450 nm (12.5 W)	1	85/15
9^b^	450 nm (10 W)	4	85/15
10^b^	446 nm (7 W)	4	57/43
11^b^	440 nm (20 W)	4	39/61
12^b^	455 nm (10 W)	4	27/72
13^b^^,^^d^	no light	4	0/100
14^b^^,^^d^	no light, no catalyst	4	0/100

a Reaction conditions: *trans*-stilbene **1-***E* (0.2 mmol), DCM (*c*= 0.1 M), [Cu] catalyst (2.5 mol %), RT, xx h, on air.

b **1-***E* (0.2 mmol), DCM (*c* = 0.2 M), **[Cu]-1** catalyst (2.5 mol %), RT, on air.

c Argon atmosphere.

d Checked for 405 and 450 nm (12.5 W).

e For detailed characteristic of the photoreactors see the SI.

f Based on GC.

**Scheme 1:**
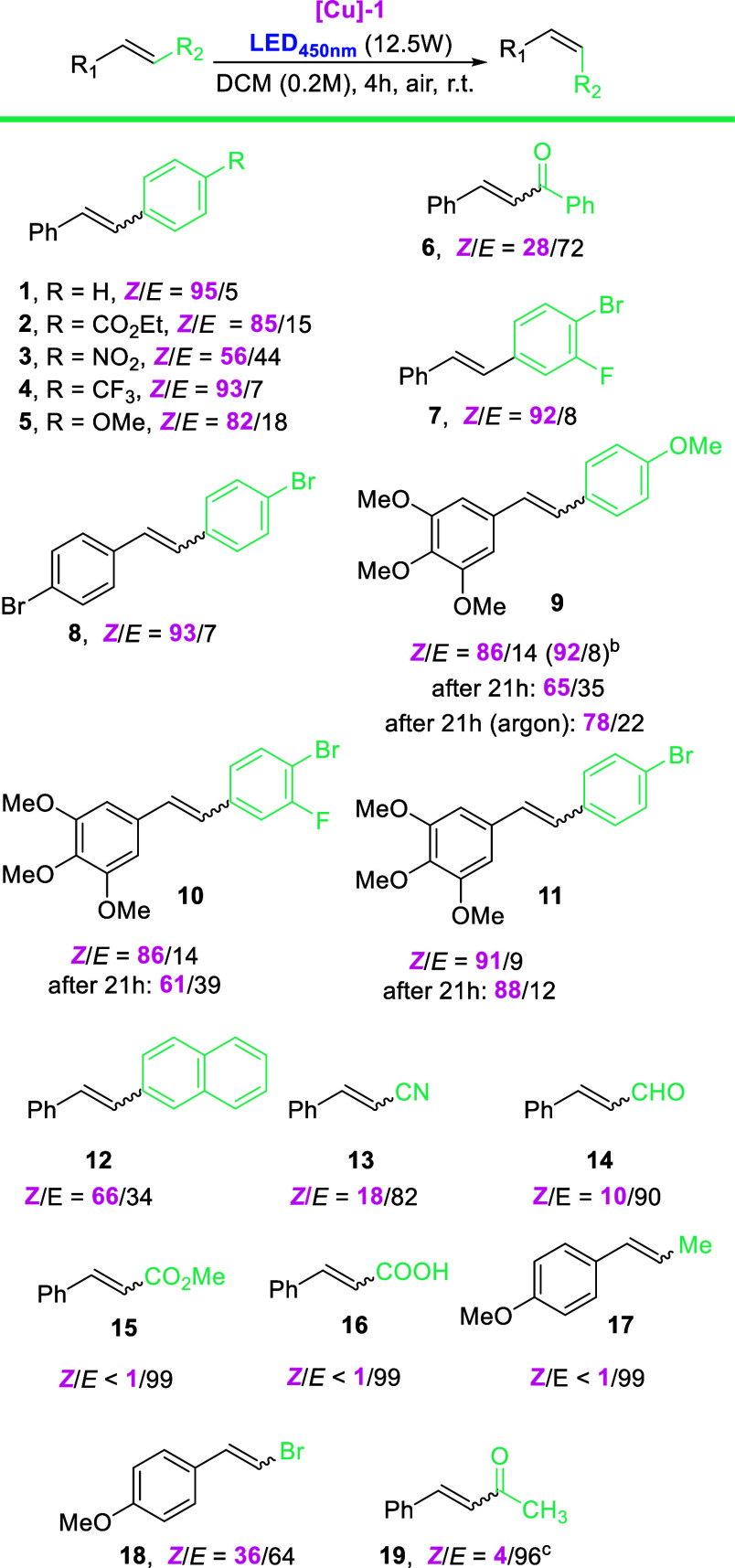
Scope and Limitations Study for Photoisomerization Standard reaction conditions: *E*-olefin (0.2 mmol), **[Cu]-1** (2.5 mol %), 450 nm (irradiation using a UOSlab photoreactor), DCM (*c* = 0.2 M), RT, 4 h, air atmosphere. *Z*/*E* ratio based on 1 H NMR. 2 h of reaction time.

Next, we decided to test the scope of the catalyzed isomerization under the optimized conditions (Scheme 1). In general, variously substituted stilbenes were susceptible to the reaction conditions (56 to 95% of isomerization). Reactions were mostly clean and gave no other products besides the *Z*-isomer and could be observed via ^1^H NMR and/or GC after 4 h of irradiation (see the **SI**). 4-Nitrostilbene **3-**E and π-extended stilbene **12-***E* were significantly less reactive. On the other hand, for substrates possessing multiple EDG—OMe: methoxystilbenes **9-***E*, **10-***E*, and **11-***E*, we observed that prolonged irradiation resulted in a decreased *Z*/*E* ratio. After short reoptimization of the reaction conditions for olefin **9-***E* (which is a proven anticancer agent named DMU-212),^18^ we found that the most optimal time for the reaction is 2 h, giving a 92/8 ratio. Presumably the reason for the back-isomerization is a lower triplet excited-state *E*/*Z* energy barrier resulting in a lesser degree of stereochemical control and/or gradual decomposition of **[Cu]-1** into photoactive derivatives leading to a different photostationary state (for details see the SI). Cinnamonitrile **13**-*E* and cinnamaldehyde **14**-*E* as well as bromostyrene **18**-*E* and styryl ketones **6**-*E* and **19**-*E* were subject to isomerization to a very limited extent while olefin derivatives **15**–**17** proved unreactive under developed conditions, presumably because the triplet energy of **[Cu]-1** (*E*_T_ ∼ 245 kJ/mol) is not high enough for these derivatives (**15**—246.8 kJ/mol, **17**—256.1 kJ/mol; for details, see the SI).

**Scheme 2:**
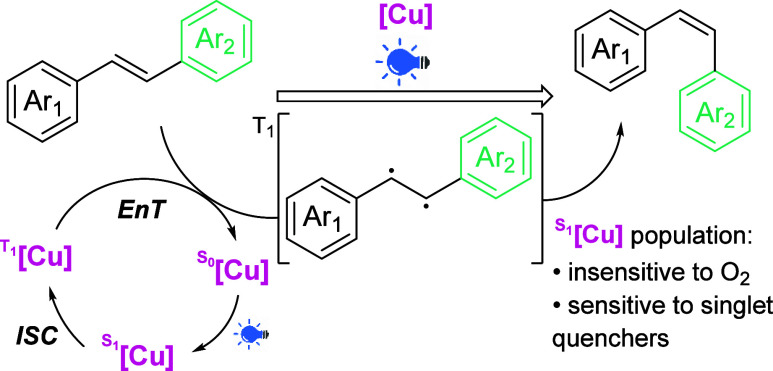
Proposed Reaction Mechanism

Based on our results and the literature data,^16,17^ we propose a plausible reaction pathway for the *E* → *Z* photoisomerization of olefins (Scheme 2). Most of the literature data suggests a triplet energy transfer pathway; however, during our studies, we established that the reaction was noticeably slowed down in the presence of a singlet-state quencher, TEMPO, and both singlet and triplet quenchers benzoquinone and azulene (see the SI). On the other hand, addition of a triplet quencher, 1,3-cyclohexadiene, does not slow down isomerization of **1-***E* in the presence of **[Cu]-1**, which, together with little sensitivity to the presence of oxygen, might suggest the involvement of a singlet state mechanism for this isomerization in agreement with previous propositions by Poisson for Cu^II^/BINAP-catalyzed *E*/*Z* isomerization of cinnamates^19^ and BINOL-catalyzed *E*/*Z* isomerization of vinyl boronates.^20^ Still, we have to take into account that singlet quenchers might affect the outcome of a triplet-energy transfer-based isomerization due to the inhibition of the photosensitizer’s S_0_ → S_1_ excitation, which produces T_1_ via ISC. To further investigate this problem, we performed reactions in the presence of triplet quenchers (O_2_ atmosphere and 1,3-cyclohexadiene) with lower catalyst loading (1%), decreased irradiation power (12.5 W), and shorter time. With this approach, we were able to see a subtle decrease of the rate of isomerization with both additives (see the SI). This observation along with previous reports^16,17^ makes us presume that reaction follows the triplet energy transfer mechanism yet is somewhat resistant toward triplet quenching. Photoisomerization of pure cis-olefins **1**-*Z* and **9**-*Z* under standard conditions leads to a very similar Z/E mixture as when the appropriate trans-olefins are used as starting materials (see the SI). This result suggests to us the presence of a specific photostationary state of the reaction and is further evidence for the triplet energy transfer as a reaction mechanism.We have also synthesized **[Cu]-2** and **[Cu]-3**, dipyridylamine analogues of [**Cu]-1**, and examined that both complexes exhibit a much slower rate of isomerization in every studied case (see the SI).

Additionally, we decided to check the photostability of **[Cu]-1** (for the procedure details, see the SI). The amount of remaining catalysts was monitored by ^1^H NMR, showing less than 1% of decomposition after 4 h and 20% decomposition after 24 h, which indicates the high photorobustness of the tested catalyst even in the presence of atmospheric oxygen. Encouraged by these results, we decided to investigate the reusability of the photocatalyst by carrying out the isomerization reaction of stilbene **11-***E* with successive addition of subsequent portions of the substrate to the reaction mixture (for a detailed procedure, see the SI). It turned out that the NHC-Cu photocatalyst not only is stable under photoirradiation but also can be used at least four times for the photoisomerization.

### Electron Transfer

After successful application of the NHC-Cu(I) photocatalyst for energy transfer reactions, we turned our attention toward its application for electron transfer reaction. Inspired by the work of Cibulka et al.^21a^ and our results, we decided to check the effectiveness of this NHC-Cu(I) (**[Cu]-1**) photocatalyst for the reduction of aryl halides. To our delight, the photoreduction of **18a** (*E*_1/2_ = −2.75 V vs SCE) under the action of **[Cu]-1** as photocatalyst yields the reduced product (**18b**) in 34% yield (Table 2). Subsequently, some reaction parameters (concentration, catalyst loading, and time; for details, see the SI) were optimized. Overall, photoreduction of **18a** catalyzed by NHC-Cu(I) (2.5 mol %) under blue-light irradiation (405 nm), in the presence of triethylamine (2.1 equiv) and Cs_2_CO_3_ (1.1 equiv) at room temperature for 24 h, gives the product **18b** in 65% yield. Background experiments confirmed that the application of photocatalyst, light, and base is crucial for the reaction to proceed (Table 2, entries 2, 3, and 5).

**Table 2:**
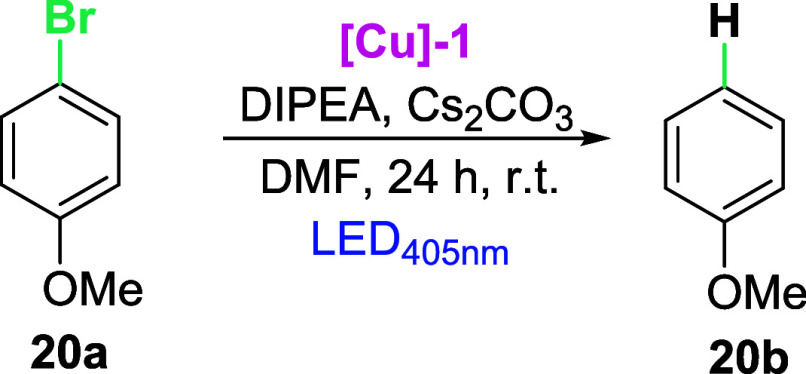
Optimization of Reaction Conditions—Photoreduction of Aryl Halides^a^

entry	variation from standard conditions	yield [%]^b^
**1**	**none**	**65**
2	no photocatalyst	0
3	no light	0
4	solvent concentration *C* = 0.0625 M	34
5	no base or pyridine instead of DIPEA	0
6	LED_390nm_ instead of LED_405nm_	20
7	19 h instead of 24 h	59

a Reaction conditions: aromatic halide **1a** (0.25 mmol, 1 equiv), dry DMF (*c* = 0.125 M), [Cu] catalyst (2.5 mol %), DIPEA (2.1 equiv), Cs_2_CO_3_ (1.1 equiv), r.t., 24 h, under an Ar atmosphere.

b GC yield.

**Scheme 3:**
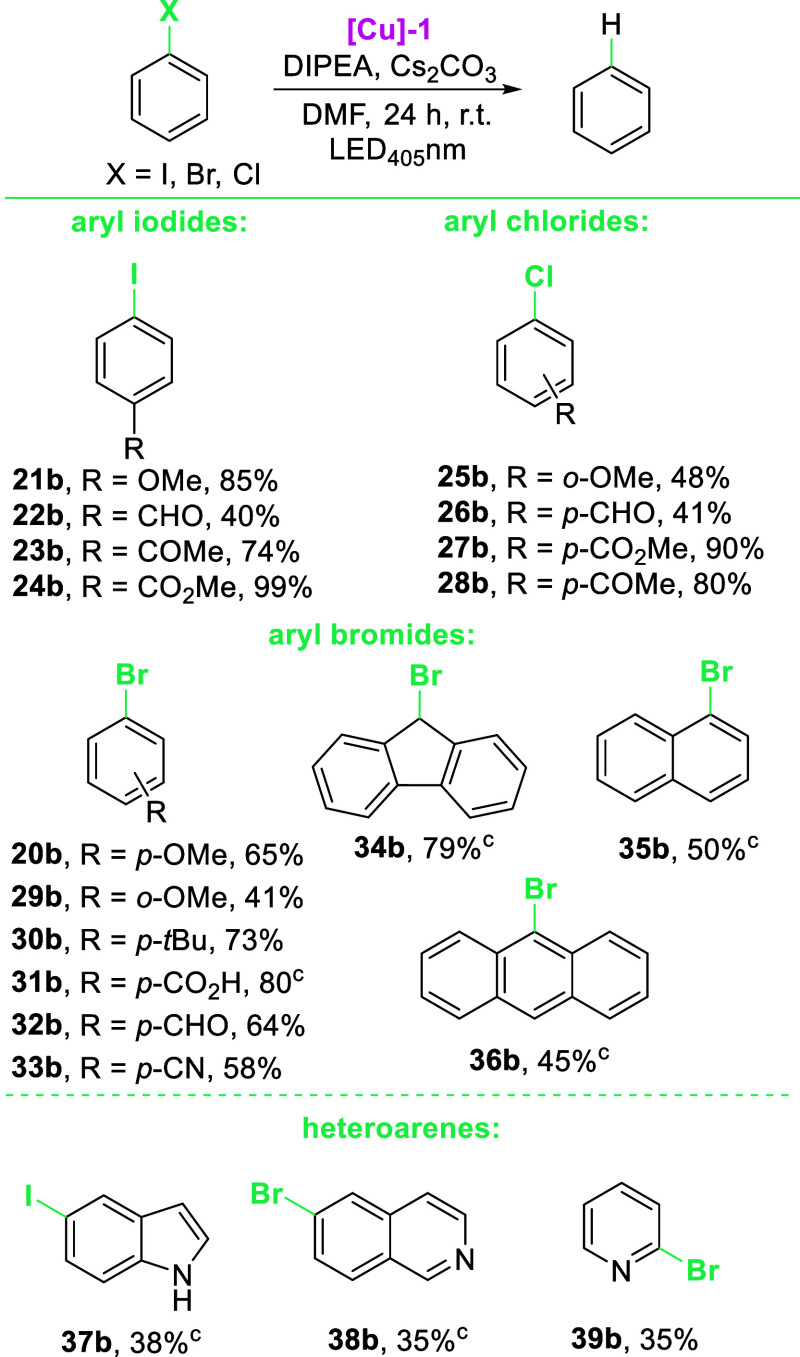
Scope and Limitations Study for Reduction of Aryl Halides Reaction conditions: aromatic halide **1a** (0.25 mmol), dry DMF (*c* = 0.125 M), [Cu] catalyst (2.5 mol %), DIPEA (0.5 mmol, 2 equiv), Cs_2_CO_3_ (0.25 mmol, 1 equiv), RT, 24 h, under an Ar atmosphere. GC yield, in brackets—substrate conversion. Isolated yields.

**Scheme 4:**
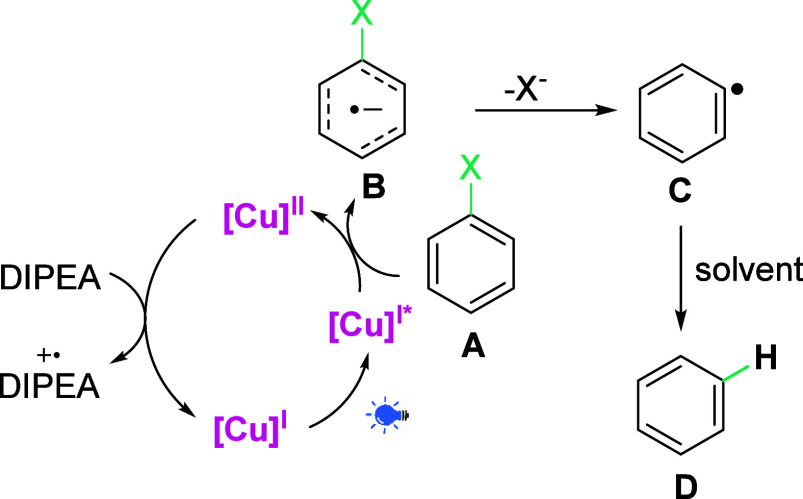
Proposed Reaction Mechanism

With the optimized conditions in hand, we examined a scope and limitations study (Scheme 3). The reduction of aryl halides proceeds nicely for arenes with various EWG functional groups (COMe, CO_2_Me, CN, and COOH) giving the desired products (**21b**, **23b**, **26b**, and **27b**) in good to superb yields (Scheme 3). Furthermore, the NHC-Cu photocatalyst is also active in the photoreduction of difficult to reduce EDG-substituted aryl halides (*E*_red_ <−2.5 V vs SCE), giving the desired product in 41–85% (**18b**, **19b**, **24b**, **28b**), depending on the halide derivative). However, the reaction proved difficult for CHO substituents for all derivatives (ArI—40%, ArBr—64%, ArCl—41%). The reduction of arenes possessing more than one aromatic ring (**34a**, **35a**, and **36a**) gives products with good to moderate yields (45–79%). On the other hand, reduction of halogen-substituted heteroarenes is also possible by the use of the NHC-Cu photocatalyst (**37**–**39**), however, with diminished yields (35–38%). Based on the aforementioned results and the literature data,^21^ we propose a plausible radical reaction pathway for the photoreduction of aryl halides under the action of the **[Cu]-1** photocatalyst (Scheme 4). In this approach, the first event is photoexcitation of [**Cu]-1** to its excited state NHC-Cu(I)*, which reduces aromatic halide **A** (for the quenching experiment, see the SI) to radical anion **B**, which after dehalogenation forms aryl radical **C**, which readily reacts with the solvent (H-donor) to form reduced product **D**. At the same time, the photocatalyst is regenerated by a sacrificial reductant (DIPEA).

## Conclusions

In summary, NHC-Cu(I) complexes, which are well-researched in conventional organometallic catalysis^22^ and as potential OLED materials,^14,15^ can also promote photoinduced energy and electron transfer processes when exposed to visible light irradiation. They act as efficient energy transfer catalysts (*E* → *Z* photoisomerization of olefins) and electron transfer photocatalysts (photoreduction of aryl halides). Studied NHC-Cu(I) photocatalysts exhibit features superior to those of other metal-based photocatalysts that work under visible-light irradiation as inexpensive and ready to make and showing great potential in future applications in biological or industrial systems. Hence, we believe that NHC-Cu(I) complexes are valuable additions to the visible-light photocatalyst library and that this study will lead to more applications.

## Data Availability

The data underlying this study are available in the published article and its online Supporting Information.

## Supporting Information Available

The Supporting Information is available free of charge at https://pubs.acs.org/doi/10.1021/acs.joc.4c00450. The Supporting Information is available free of charge on the ACS Publications Web site at DOI: Optimization details, experimental procedures, DFT calculations and characterization data for all new compounds (PDF)
