# Supplementary material for: NHC-Cu Three-Coordinate Complex as a Promising Photocatalyst for Energy and Electron Transfer Reactions
Source: J Org Chem. 2024 Jun 3;89(12):8546–50. doi: 10.1021/acs.joc.4c00450 (PMC11197101; doi:10.1021/acs.joc.4c00450)

Supporting Information (SI)

for

NHC-Cu three-coordinate complex as a promising photocatalyst for energy and electron transfer reactions

Krzysztof Grudzień[a]*, Zuzanna Szeptuch[a,b], Hubert Kubiszewski[a,c], Wojciech Chaładaj[a]* and Katarzyna Rybicka-Jasińska[a]*

a Institute of Organic Chemistry, Polish Academy of Sciences, Kasprzaka 44/52, 01-224 Warsaw, Poland
b Faculty of Chemistry, Warsaw University of Technology, Noakowskiego 3, 00-664 Warsaw, Poland
c Faculty of Medicine, Medical University of Warsaw, Żwirki i Wigury 61, 02-091 Warsaw, Poland

krzysztof.grudzien@icho.edu.pl

wojciech.chaładaj@icho.edu.pl

katarzyna.rybicka-jasinska@icho.edu.pl

Table of contest:

1. General information **S3**

2. Photoreactors setup **S4**

3. Synthesis of catalysts and substrates **S6**

4. Photoreaction procedures **S18**

4.1. Energy transfer reactions – E→ Z photoisomerization **S18**

4.2. Electron transfer reaction - [Cu]-three coordinated complex as photoredox catalysts **S74**

5. References **S84**

6. NMR spectra of new isolated compounds **S86**

1. GENERAL INFORMATION

All solvents and commercially available reagents were purchased from Sigma-Aldrich, TCI, Acros Organics, Angene, AmBeed as reagent grade and were used without further purification, unless otherwise stated.

Dry solvents were taken from Solvent Purification System (SPS) or purchased from Sigma Aldrich. Deuterated solvents were purchased from Eurisotop.

All the photochemical reactions were performed in 10 mL glass vials sealed with aluminum caps containing a rubber septa.

Reactions were monitored by thin layer chromatography (TLC), using 0.20 mm Merck silica plates (60F-254) and visualized using UV-light, potassium permanganate, cerium molybdate or anisaldehyde stain, with heat as a developing agent. Colum chromatography was performed on Merck silica gel 60 (230-400 mesh).

NMR spectra were recorded at ambient temperature (unless otherwise stated) on Bruker 400MHz or Varian 500, 600 MHz. Chemical shifts are reported in ppm relative to tetramethyl silane signal or a residual undeuterated solvent peak (TMS – 0 ppm for ^{1}H and ^{13}C, $CHCl_3$ – 7.26 ppm for ^{1}H and 77.00ppm for ^{13}C, acetone-d_6 – 2.05 ppm for ^{1}H). Multiplicities are given as: singlet (s), doublet (d), triplet (t), quartet (q), septet (sept), multiplet (m), broad singlet (bs),.

HRMS High-resolution mass spectra (HRMS) were recorded on Synapt G2-S HDMS (Waters Inc) mass spectrometer equipped with an electrospray ion (atmospheric pressure chemical ionization) source and q-TOF type mass analyzer. The instrument was controlled and recorded data was processed using MassLynx V4.1 software package (Waters Inc). The Leucine-Enkephalin solution was used as the Lock-Spray reference material and the exact mass measurements for all peaks were performed within 3 mDa mass error.

UV-Vis absorption spectra were recorded on UV-3600i Plus UV-Vis-NIR Spectrophotometer.

Fluorescence measurements were performed on Edinburgh Instruments FS5 Spectrofluorometer.

GC analyses were performed using Shimadzu GCMS-QP2010 SE gas chromatograph with FID detector and Zebron ZB 5MSi column

2. PHOTOREACTOR SETUPS

1. **UOSlab** miniphoto photoreactor (commercially available). Violet (λ_{max} = 405 nm) or blue (λ_{max} = 450 nm) light was supplied to each reaction vial with the use of 7 LUMINUS LED units (of overall 25W intensity when 100% power applied). The ambient temperature of LED block was maintained by cooling with Huber MiniChiller 300.

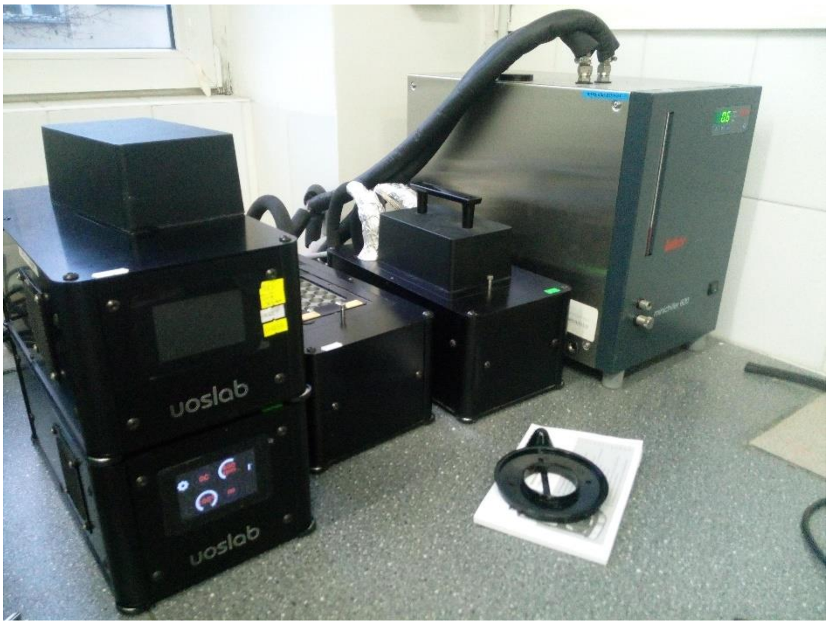

Figure S1

2. **Homemade1** constructed photoreactor setup with an aluminum cooling block The LED plates are commercially available radiators (Fischer Electronic part no. SK 105 100 SA) with 6 epoxy-glued star-cased LEDs connected in series (constant current 0.7 A power supply). Reactions were performed under blue light irradiation on a single diode (LT-2855 royal blue, λ_{max}: 446 nm, 7W) per vial with 6 mm distance from the plate. The temperature of LED block was controlled with Huber MiniChiller 300.

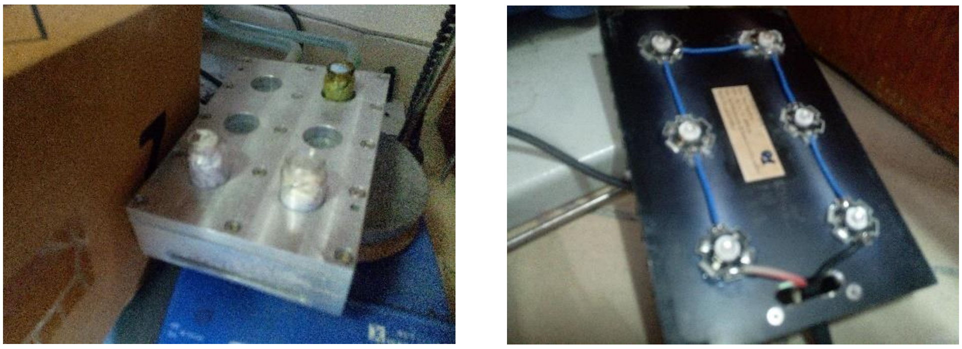

Figure S2

3. Single **Kessil** lamp (commercially available) providing blue light (λ_{max} = 440 nm) irradiation with 40W intensity when 100% power is applied. The ambient temperature of reaction vessel was maintained by cooling with the use of fan.

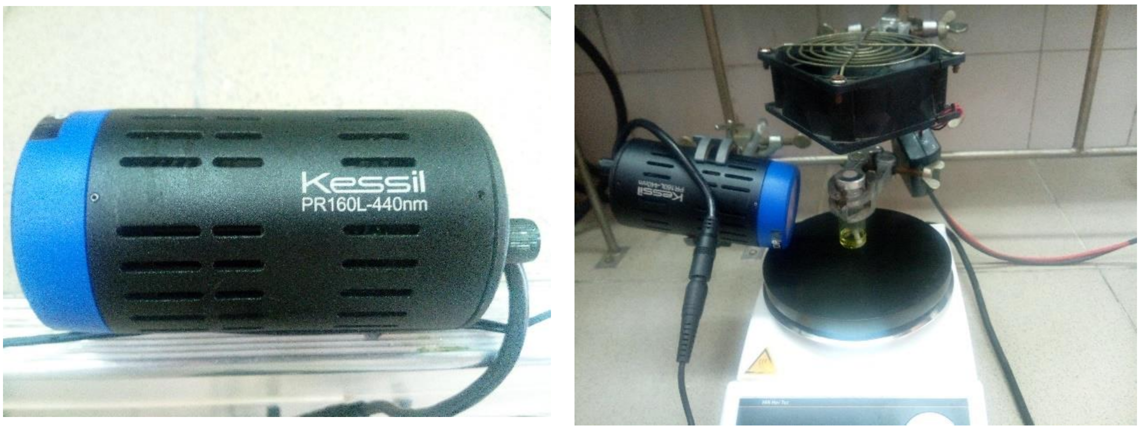

Figure S3

4. **Homemade2** photoreactor employing a single lamp providing blue light (λ_{max} = 455 nm) irradiation with 10W intensity. The ambient temperature of reaction vessel was maintained by cooling with the use of fan.

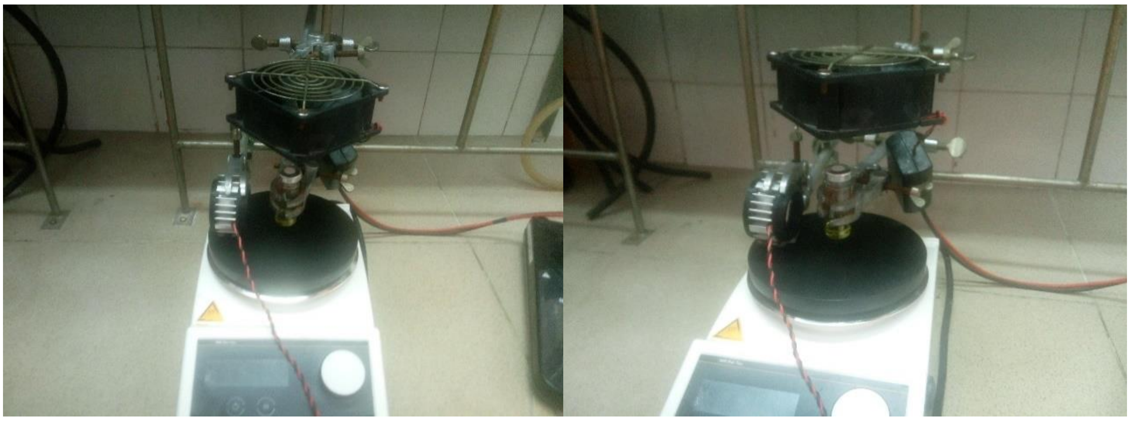

Figure S4

3. SYNTHESIS OF CATALYSTS AND SUBSTRATES

3.1. Synthesis of [Cu]-photocatalysts

Synthesis of **IPrCuCl**

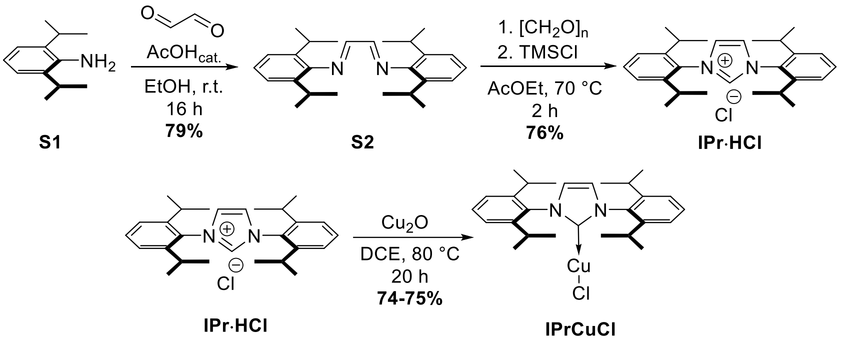

IPrCuCl was synthesized starting from 2,6-diisopropylphenylaniline (**S1**), following a 3-step procedure.

Step 1. Glyoxal (40% solution in H_2O, 4.79 g, 33.0 mmol), S1 (12.87 g, 72.6 mmol) and EtOH (125 mL) were placed in 250 mL round-bottom flask along with PTFE-coated magnetic stirring bar. Concentrated acetic acid (0.1 mL) was added, resulting mixture was stirred overnight (16 h) at r.t. Yellow precipitate was collected on a Schott funnel and washed thoroughly with cold MeOH (3 x 50 mL), then dried in vacuo. 6.393 g (17.0 mmol, 51%) **S2** as a bright yellow solid was obtained. Combined filtrated were placed in freezer (-20 °C) for few days which resulted in a second crop of precipitation that was collected on a Schott funnel, washed with cold MeOH and dried to give 3.43 g (9.1 mmol, 28%) of **S2**. Total yield of diamine obtained this way: 79%. Product was directly used in the next step.

Step 2. S2 (3.43 g, 9.1 mmol) and paraformaldehyde (285 mg, 9.5 mmol) were placed in 150 mL round-bottom flask along degassed AcOEt (90 mL) and PTFE-coated magnetic stirring bar. Flask was sealed with rubber septum and placed in pre-heated oil bath (70 °C), stirred for 15 min, then solution of TMSCl (1.3 mL, 10.0 mmol) in degassed AcOEt (10 mL) was added dropwise (for about 5 min). Mixture was stirred at 70 °C for 2.5 h, then cooled down to r.t. Resulting precipitate was collected on a Schott funnel, washed with AcOEt and Et_2O then dried in vacuo to give 2.95 g (6.95 mmol, 76%) of **IPr·HCl** as a white solid.

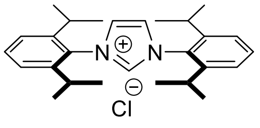

IPr·HCl

IPr·HCl

^{1}H NMR (500 MHz, $CDCl_3$) δ 10.13 (s, 1H), 8.14 (d, *J* = 1.6 Hz, 2H), 7.57 (t, *J* = 7.8 Hz, 2H), 7.35 (d, *J* = 7.9 Hz, 4H), 2.45 (sept, *J* = 6.8 Hz, 4H), 1.29 (d, *J* = 6.8 Hz, 12H), 1.25 (d, *J* = 6.9 Hz, 12H).

Literature NMR (*Angew. Chem. Int. Ed.* **2018**, *57*, 4668–4672):

^{1}H NMR (400 MHz, $CDCl_3$) δ 10.14 (s, 1H), 8.15 (d, *J* = 1.6 Hz, 2H), 7.58 (t, *J* = 7.9 Hz, 2H), 7.35 (d, *J* = 7.8 Hz, 4H), 2.45 (sept, *J* = 6.8 Hz, 4H), 1.29 (d, *J* = 6.8 Hz, 12H), 1.25 (d, *J* = 6.9 Hz, 12H) ppm

Step 3. IPr·HCl (425 mg, 1.00 mmol) Cu_2O (143 mg, 1.00 mmol) and degassed DCE (10 mL) were placed in 50 mL round-bottom flask along with PTFE-coated magnetic stirring bar. Flask was sealed with rubber septum and placed in pre-heated oil bath (80 °C), then stirred for 20 h. After cooling down to r.t. crude mixture was filtered through pad of SiO_2 on a Schott funnel that was washed with additional portion of DCM (20 mL). Combined filtrates were concentrated in vacuo, residue was crystallized

(DCM/hexane, rotary evaporation), resulting precipitate was filtered off, washed with hexane and dried in vacuo to give 360 mg (0.74 mmol, 74%) of **IPrCuCl** as a white crystalline solid. Reaction was repeated in 5.00 mmol scale in 75% yield.

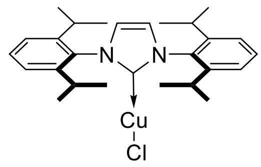

IPrCuCl

IPrCuCl

^{1}H NMR (500 MHz, $CDCl_3$) δ = 7.49 (t, *J* = 7.8 Hz, 2H), 7.29 (d, *J* = 7.8 Hz, 4H), 7.13 (s, 2H), 2.57 (p, *J* = 6.9 Hz, 4H), 1.30 (d, *J* = 6.9 Hz, 12H), 1.23 (d, *J* = 6.9 Hz, 12H) ppm.

Literature NMR[1] (*Inorg. Chem.* **2014**, 53, *17*, 9181–9191):

^{1}H NMR ($CDCl_3$, 400MHz): δ = 7.49 (t, J = 7.8 Hz, 2H), 7.30 (d, J = 7.8 Hz, 4H), 7.13 (s, 2H), 2.56 (sept, J = 6.8 Hz, 4H), 1.30 (d, J = 6.8 Hz, 12H), 1.22 (d, *J* = 6.9 Hz, 12H) ppm.

Intermediate **IPr·HCl** was also utilized in one pot synthesis of **[Cu]-1** as well as a ligand in Heck coupling.

Synthesis of **[Cu]-1**

Standard procedure

(following Marion et al. *Inorg. Chem.* **2014**, 53, *17*, 9181–9191)[1]

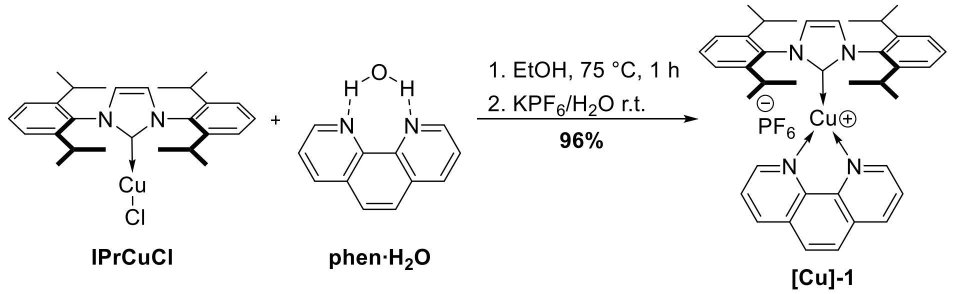

IPrCuCl (975, 2.00 mmol), phen·H_2O (396 mg, 2.00 mmol) and a stirring bar were placed in round-bottom flask (100 mL). EtOH (20 mL) was added, flask was sealed with rubber septum, vigorously stirred mixture was degassed by purging with the stream of argon through a needle piercing the septum for 20 min. Then sealed flask was placed in pre-heated oil bath (75 °C), mixture was stirred vigorously (620 rpm) for 60 min. After cooling down to r.t. solution of KPF_6 (1472 mg, 8.00 mmol) in distilled water (30 mL) was added. Resulting precipitate was collected on a Schott funnel, washed with distilled water (2 x 20 mL) and Et_2O (2 x 40 mL). Product was dried for 15 min on air then overnight at high vacuum to give 1490 mg (1.92 mmol, 96%) of **[Cu]-1** as a pale yellow solid.

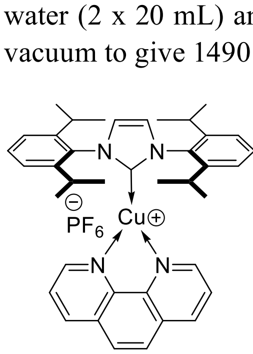

[Cu]-1

^{1}H NMR (500 MHz, $CDCl_3$) δ = 8.46 (dd, *J* = 8.3, 1.6 Hz, 2H), 7.91 (s, 2H), 7.79 (t, *J* = 7.8 Hz, 2H), 7.59 (dd, *J* = 8.2, 4.7 Hz, 2H), 7.52 (d, *J* = 7.9 Hz, 4H), 7.39 (s, 2H), 6.76 (dd, *J* = 4.7, 1.5 Hz, 2H), 2.70 (p, *J* = 6.9 Hz, 4H), 1.30 (d, *J* = 6.8 Hz, 12H), 1.07 (d, *J* = 6.7 Hz, 12H) ppm.

^{13}C{1H}NMR (126 MHz, $CDCl_3$) δ 149.7, 146.4, 143.5, 138.9, 135.9, 130.7, 129.0, 127.0, 125.2, 124.8, 123.6, 28.7, 25.0, 23.6 ppm.

One-pot procedure

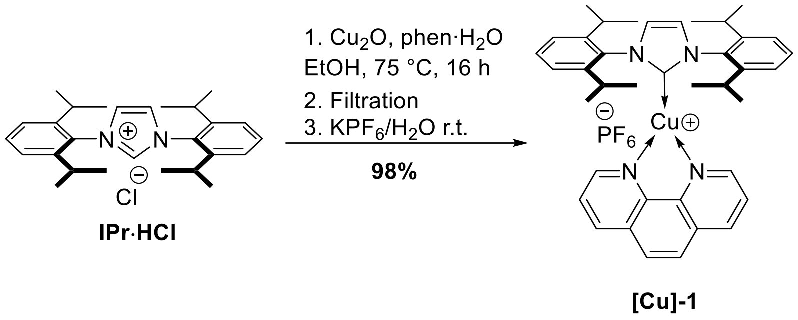

IPr·HCl (425 mg, 1.00 mmol), Cu_2O (143 mg, 1.00 mmol) and phen·H_2O (198 mg, 1.00 mmol) and a stirring bar were placed in round-bottom flask (50 mL). EtOH (10 mL) was added, flask was sealed with rubber septum, vigorously stirred mixture was degassed by purging with the stream of argon through a needle piercing the septum for 20 min. Then sealed flask was placed in pre-heated oil bath (75 °C), mixture was stirred vigorously (620 rpm) overnight (around 16 h). After cooling down to r.t. mixture was filtered through the pad of celite on a Schott funnel, which was then rinsed with additional portion of EtOH (10 mL). Solution of KPF_6 (736 mg, 4.00 mmol) in distilled water (20 mL) was added. Resulting precipitate was collected on a Schott funnel, washed with distilled water (2 x 10 mL) and Et_2O (2 x 20 mL). Product was dried for 15 min on air then overnight at high vacuum to give 762 mg (0.98 mmol, 98%) of **[Cu]-1** as a pale yellow solid.

^{1}H NMR (600 MHz, $CDCl_3$) δ = 8.46 (dd, *J* = 8.2, 1.5 Hz, 2H), 7.91 (s, 2H), 7.79 (t, *J* = 7.9 Hz, 2H), 7.59 (dd, *J* = 8.2, 4.7 Hz, 2H), 7.52 (d, *J* = 7.8 Hz, 4H), 7.40 (s, 2H), 6.75 (dd, *J* = 4.7, 1.5 Hz, 2H), 2.69 (p, *J* = 6.9 Hz, 4H), 1.30 (d, *J* = 6.9 Hz, 12H), 1.07 (d, *J* = 6.9 Hz, 12H) ppm.

Literature NMR[1] of **[Cu]-1**

^{1}H NMR (400 MHz, $CDCl_3$) δ = 1.07 (d, *J* = 6.8 Hz, 12H), 1.30 (d, *J* = 6.8 Hz, 12H), 2.69 (sept, *J* = 6.8 Hz, 4H), 6.74 (d, *J* = 3.6 Hz, 2H), 7.40 (s, 2H), 7.52 (d, *J* = 7.7 Hz, 4H), 7.59 (dd, *J* = 7.6 Hz et *J* = 4.8 Hz, 2H), 7.79 (t, *J* = 7.7 Hz, 2H), 7.91 (s, 2H), 8.45 (d, *J* = 8.0 Hz, 2H) ppm.

^{13}C{1H}NMR ($CDCl_3$, 100 MHz) δ = 23.6 (q×4), 25.1 (q×4), 28.9 (d×4), 123.6 (d×2), 124.8 (d×4), 125.3 (d×2), 127.0 (d×2), 128.9 (s×2), 130.7 (d×2), 135.9 (s×2), 138.9 (d×2), 143.5 (s×2), 146.4 (s×4), 149.7 (d×2), 183.0 (s) ppm.

Synthesis of **[Cu]-2** and **[Cu]-3**

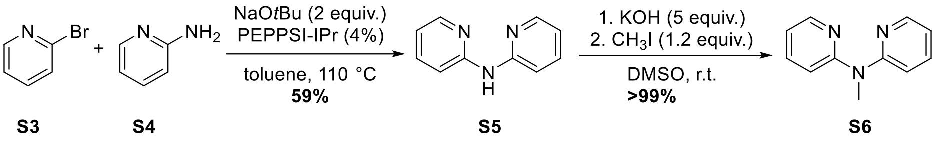

S6 was synthesized following two-step procedure

Step 1. Commercially available PEPPSI-IPr catalyst (85 mg, 0.125 mmol) was placed along 2-bromopyridine (**S3**, 780 mg, 5.00 mmol), 2-aminopyridine (**S4**, 471 mg, 5.00 mmol), NaO*t*Bu (960 mg, 10.00 mmol) and PTFE-coated magnetic stirring bar in 50 ml round-bottom flask under argon, then anhydrous toluene was added and flask was sealed with a rubber septum. Mixture was stirred at 100 °C (oil bath) for 24 h. After cooling to r.t. water (around 20 mL) was added, mixture was transferred into a separation funnel using AcOEt (around 40 mL) to rinse flask. Phases were separated, aqueous phase was extracted with DCM (around 20 mL) and AcOEt (around 40 mL). Combined organic phases were dried

over anhydrous Na_2SO_4. Drying agent was filtered off, residue was purified by column chromatography (SiO_2, n-hex/AcOEt 1:1) to give 505 mg (2.95 mmol, 59%) of **S5** as an orange solid.

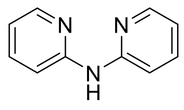

S5

2,2'-dipyridylamine, **S5**

^{1}H NMR (400 MHz, $CDCl_3$) δ 8.26 (ddd, *J* = 5.1, 1.9, 0.9 Hz, 2H), 7.60 (ddd, *J* = 8.9, 7.0, 1.9 Hz, 2H), 7.54 (dt, *J* = 8.4, 1.1 Hz, 2H), 6.85 (ddd, *J* = 7.0, 5.0, 1.2 Hz, 2H).

Literature NMR[2] (*J. Org. Chem.* **1996**, *61*, 21, 7240–7241)

^{1}H NMR (300 MHz, $CDCl_3$) δ 8.28 (d, J = 4.9 Hz, 2H), 8.15 (s, 1 H), 7.55-7.63 (m, 4H), 6.85 (t, *J* = 5.4 Hz, 2H).

Step 2. **S5** (361 mg, 2.10 mmol), DMSO (reagent grade, 4 mL) and KOH (589 mg, 10.50 mmol) were placed along a PTFE-coated magnetic stirring bar in a round-bottom flask. The reaction mixture was stirred at r.t. for 30 min. CH_3I (156 µL, 2.50 mmol) was added and the reaction mixture was stirred at r.t. for 1 h. Water (50 mL) was added and the aqueous layer was extracted with Et_2O (3 x 50mL). The combined organic layers were washed with water (3 x 50mL) and brine (50 mL) then dried over Na_2SO_4, filtered and concentrated under vacuum. The crude product was purified by column chromatography (SiO_2, hexane/AcOEt 1:1) to obtain **S6** as a yellow oil (383 mg, 2.07 mmol, >99% yield).

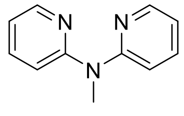

S6

N-methyl-2,2'-dipyridylamine, **S6**

^{1}H NMR (400 MHz, $CDCl_3$) δ 8.35 (ddd, *J* = 5.0, 2.0, 0.9 Hz, 2H), 7.53 (ddd, *J* = 8.4, 7.2, 2.0 Hz, 2H), 7.17 (dt, *J* = 8.4, 1.0 Hz, 2H), 6.85 (ddd, *J* = 7.2, 4.9, 0.9 Hz, 2H), 3.62 (s, 3H) ppm.

Literature NMR[3] (*ACS Appl. Mater. Interfaces* **2016**, *8*, 23, 14678–14691)

^{1}H NMR (400 MHz, $CDCl_3$): δ), 8.35 (d, *J* = 4.2 Hz, 2H), 7.55 (td, *J* = 8.4, 4.2 Hz, 2H), 7.17 (d, *J* = 8.4 Hz, 2H), 6.87 (t, *J* = 8.4 Hz, 2H), 3.64(s, 3H) ppm.

[Cu]-2 was obtained following literature procedure[1,3]

(*ACS Appl. Mater. Interfaces* **2016**, *8*, 23, 14678–14691)

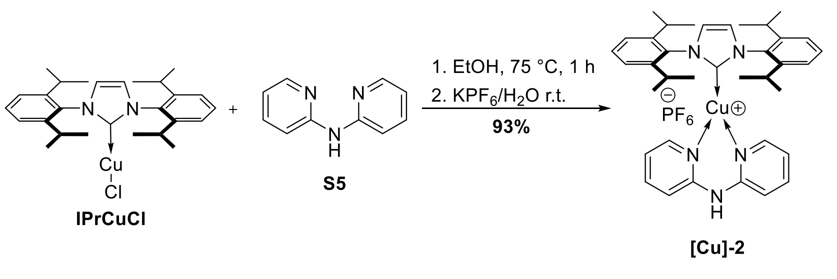

IPrCuCl (366 mg, 0.75 mmol), **S5** (128 mg, 0.75 mmol) and a PTFE-coated magnetic stirring bar were placed in round-bottom flask (50 mL). EtOH (8 mL) was added, flask was sealed with rubber septum, vigorously stirred mixture was degassed by purging with the stream of argon through a needle piercing the septum for 20 min. Then sealed flask was placed in pre-heated oil bath (75 °C), mixture was stirred vigorously (620 rpm) for 60 min. After cooling down to r.t. solution of KPF_6 (414 mg, 2.25 mmol) in distilled water (12 mL) was added. Resulting precipitate was collected on a Schott funnel, washed with distilled water (2 x 10 mL) and Et_2O (2 x 15 mL). Product was dried for 15 min on air then overnight at high vacuum to give 537 mg (0.70 mmol, 93%) of **[Cu]-2** as a yellow solid.

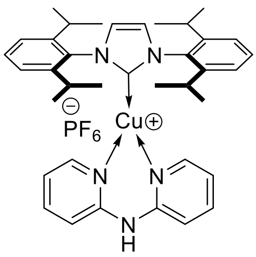

[Cu]-2

[Cu]-2

^{1}H NMR (400 MHz, $CDCl_3$) δ 8.07 (s, 1H), 7.57 (t, *J* = 7.8 Hz, 2H), 7.50 – 7.40 (m, 2H), 7.33 (d, *J* = 7.8 Hz, 4H), 7.22 (s, 2H), 7.13 (d, *J* = 8.4 Hz, 2H), 6.37 – 6.27 (m, 2H), 6.18 (dd, *J* = 5.6, 1.9 Hz, 2H), 2.66 (p, *J* = 6.9 Hz, 4H), 1.22 (d, *J* = 6.9 Hz, 12H), 1.07 (d, *J* = 6.8 Hz, 12H) ppm.

Literature NMR[1] (*Inorg. Chem.* **2014**, 53, *17*, 9181–9191)

^{1}H NMR (400 MHz, $CDCl_3$): 8.08–8.13 (*br* s, NH), 7.58 (t, *J* = 7.8 Hz, 2H), 7.48 (t, *J* = 7.5 Hz, 2H), 7.33 (d, *J* = 7.8 Hz, 4H), 7.22 (s, 2H), 7.16 (d, *J* = 8.5 Hz, 2H), 6.31 (t, *J* = 6.4 Hz, 2H), 6.17 (d, *J* = 5.4 Hz, 2H), 2.65 (sept, *J* = 6.9 Hz, 4H), 1.23 (d, *J* = 6.9 Hz, 12H), 1.08 (d, *J* = 6.9 Hz, 12H) ppm.

[Cu]-3 was obtained following literature procedure[1,3]

(*ACS Appl. Mater. Interfaces* **2016**, *8*, 23, 14678–14691)

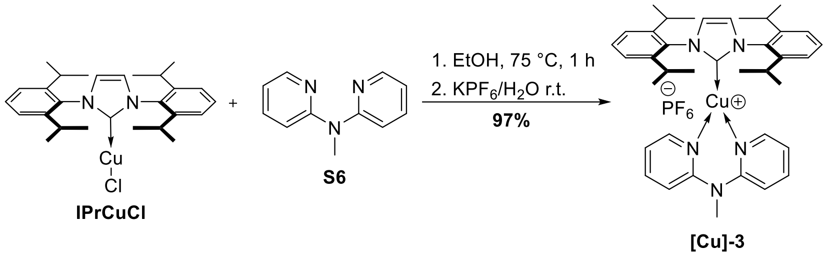

IPrCuCl (731 mg, 1.50 mmol), **S6** (278 mg, 1.50 mmol) and a PTFE-coated magnetic stirring bar were placed in round-bottom flask (100 mL). EtOH (16 mL) was added, flask was sealed with rubber septum, vigorously stirred mixture was degassed by purging with the stream of argon through a needle piercing the septum for 20 min. Then sealed flask was placed in pre-heated oil bath (75 °C), mixture was stirred vigorously (620 rpm) for 60 min. After cooling down to r.t. solution of KPF_6 (828 mg, 4.50 mmol) in distilled water (25 mL) was added. Resulting precipitate was collected on a Schott funnel, washed with distilled water (2 x 20 mL) and hexane (2 x 40 mL). Product was dried for 15 min on air then overnight at high vacuum to give 1140 mg (1.46 mmol, 97%) of **[Cu]-3** as a pale yellow solid.

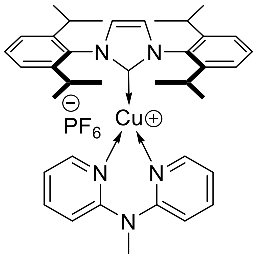

[Cu]-3

[Cu]-3

^{1}H NMR (400 MHz, $CDCl_3$) δ 7.72 (ddd, *J* = 8.9, 5.3, 4.1 Hz, 2H), 7.51 (t, *J* = 7.8 Hz, 2H), 7.33 – 7.21 (m, 6H), 7.12 (d, *J* = 8.5 Hz, 2H), 6.64 (d, *J* = 4.7 Hz, 3H), 3.32 (s, 3H), 2.62 (p, *J* = 6.9 Hz, 4H), 1.22 (d, *J* = 6.9 Hz, 12H), 1.08 (d, *J* = 6.6 Hz, 12H) ppm.

Literature NMR[3] (*ACS Appl. Mater. Interfaces* **2016**, *8*, 23, 14678–14691)

^{1}H NMR (400 MHz, $CDCl_3$): δ 7.73 (tt, *J* = 6.4, 2.6 Hz, 2H), 7.52 (t, *J* = 7.8 Hz, 2H), 7.27 (d, *J* = 7.8 Hz, 4H), 7.26 (s, 2H), 7.15 (d, *J* = 8.6 Hz, 2H), 6.63 (d, *J* = 4.6 Hz, 4H), 3.34 (s, 3H), 1.22 (d, *J* = 6.7 Hz, 12H), 1.08 (d, *J* = 6.7 Hz, 12H) ppm.

Synthesis of **[Cu]-4** and **[Cu]-5**

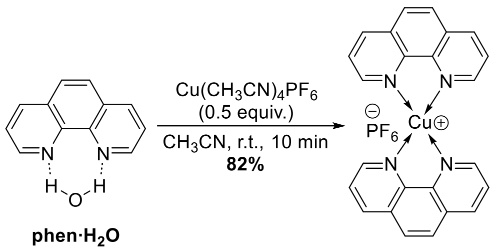

phen·H$_2$O **[Cu]-4**

[Cu]-4 was synthesized following literature procedure[4] (*Inorg. Chem.* **1996**, 35, *22*, 6406–6412). Magnetically stirred solution of phen·H$_2$O (396 mg, 2.00 mmol) in CH$_3$CN (HPLC grade, 10 mL) was charged with Cu(CH$_3$CN)$_4$PF$_6$ (373 mg, 1.00 mmol) at r.t. Resulting mixture was stirred at r.t. for 10 min after which excess Et$_2$O (around 50 mL) was added. Resulting dark precipitate was collected on a Schott funnel. Crude product was re-dissolved in minimal amount of DCM and precipitated again with excess Et$_2$O, collected on a Schott funnel, washed with Et$_2$O and dried in vacuo. Product obtained as violet solid (467 mg,0.82 mmol, 82%).

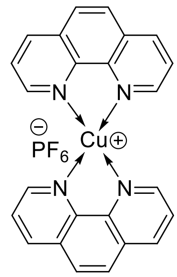

[Cu]-4

[Cu]-4

^{1}H NMR (500 MHz, acetone-*d$_6$*) δ 9.12 (bs, 4H), 8.88 (d, *J* = 8.1 Hz, 4H), 8.33 (bs, 4H), 8.08 (bs, 4H).

Literature NMR (*Inorg. Chem.* **1996**, 35, *22*, 6406–6412)

^{1}H NMR (300 MHz, acetone-*d$_6$*) δ: 9.13 (d,4H), 8.89 (d, 4H), 8.34 (s, 4H), 8.08 (m, 4H).

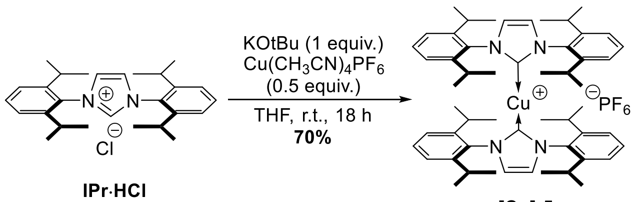

IPr·HCl **[Cu]-5**

[Cu]-5 was synthesized following literature procedure[5] (*Organometallics* **2006**, 25, *9*, 2355–2358). **IPr·HCl** (425 mg, 1.00 mmol), Cu(CH$_3$CN)$_4$PF$_6$ (186 mg, 0.50 mmol) and KO*t*Bu (123 mg, 1.10 mmol) were placed along with PTFE-coated magnetic stirring bar in a round bottom flask under argon. Anhydrous THF (10 mL) was added, resulting mixture was stirred at r.t. for 18h. Contents of the flask were filtered through a pad of celite, then filtrate was mixed with excess hexane (around 50 mL), resulting in precipitation of white solid that was collected on a Schott funnel, washed with additional hexane and dried in vacuo to give 350 mg (0.35 mmol, 70%) of **[Cu]-5** as an off-white solid.

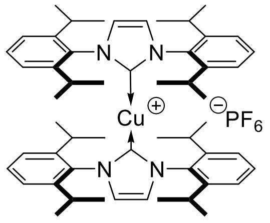

[Cu]-5

[Cu]-5

^{1}H NMR (500 MHz, CDCl$_3$) δ 7.43 (t, *J* = 7.8 Hz, 2H), 7.10 (d, *J* = 7.8 Hz, 4H), 6.96 (s, 2H), 2.30 – 2.22 (m, 4H), 1.00 (d, *J* = 6.9 Hz, 12H), 0.81 (d, *J* = 7.0 Hz, 12H).

Literature NMR[6] (*Angew. Chem. Int. Ed.* **2021**, 61, e202113841):

^{1}H NMR (400 MHz, CDCl$_3$): 7.45 (2H, t, *J* = 7.78 Hz), 7.12 (4H, d, *J* = 7.80 Hz), 6.98 (2H, s), 2.32 – 2.25 (4H, m), 1.02 (12H, d, *J* = 6.84 Hz), 0.83 (12H, d, *J* = 6.92 Hz).

Attempted synthesis of **[Cu]-6**

In order to check the reactivity of **[Cu]-1** analogues with modified electronic properties, we also attempted the synthesis of a complex containing the 5,6-phenanthroquinone (**S9**) ligand in place of the parent phenanthroline.

Synthesis of **S7**

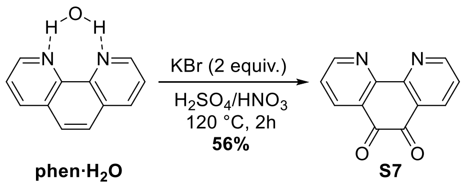

S7 was synthesized following modified literature procedure[7] (*Molecules* **2021**, 26(11), 3244)

Concentrated sulfuric acid (10 mL) was placed in 100 mL round-bottom flask which was then placed in cooling bath (water/ice). Mixture of **phen·H_2O** (1.09 g, 5.5 mmol) and KBr (1.31 g, 11.0 mmol) was added in portions. HNO_3 (65% aqueous solution, 5 mL) was added slowly, resulting mixture was stirred at r.t. for 15 min. Flask was equipped with a reflux condenser (running cold water as a cooling medium), mixture was stirred at 120 °C for 2h. After cooling down to r.t. mixture was poured into ice water (200 mL), neutralized with K_2CO_3 and transferred into a separation funnel. Reaction mixture was extracted with DCM (3 × 100 mL) dried over anhydrous Na_2SO_4 and concentrated in vacuo. Crude product was recrystallized from EtOH to give 645 mg (3.1 mmol, 56%) of **S7** as fine orange needles.

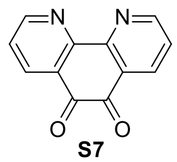

S7

^{1}H NMR (500 MHz, $CDCl_3$) δ 9.09 (dd, *J* = 4.7, 1.8 Hz, 2H), 8.48 (dd, *J* = 7.9, 1.9 Hz, 2H), 7.57 (dd, *J* = 7.8, 4.7 Hz, 2H).

^{13}C{1H} NMR (126 MHz, $CDCl_3$) δ 178.60, 156.34, 152.84, 137.24, 128.02, 125.55.

Literature NMR[8] (*Dalton Trans.* **2016**, 45, 16366-16378)

^{1}H NMR ($CDCl_3$, 400 MHz) δ 9.13 (dd, J = 4.7, 1.9 Hz, 2H), 8.51 (dd, J = 7.9, 1.9 Hz, 2H), 7.59 (dd, J = 7.9, 4.7 Hz, 2H).

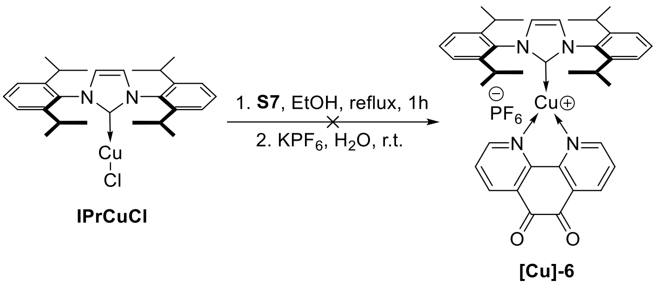

Reaction starting with **IPrCuCl** (488 mg, 1.0 mmol) and **S7** (210 mg, 1.0 mmol), following literature procedure for the synthesis of **[Cu]-1** resulted in brownish gray solid that turned out to be a mixture of starting materials (based on ^{1}H NMR analysis of crude mixture). Nevertheless we tested catalytic activity of this mixture and, to our surprise, it turned out to be somewhat effective in *E*/*Z* isomerization of olefin. This lead us to carry out a thorough background tests of our isomerization where we established that although **IPrCuCl** is not an active photocatalyst yet to some extent ligands **phen·H_2O** and **S7** (copper-free) can catalyze photoisomerization of stilbene **1-*E*** and even *trans*-anethole **(17-E)** (see: PHOTOREACTION PROCEDURES)

UV-Vis absorption spectra for catalysts **[Cu]-1**, **[Cu]-2**, **[Cu]-3**:

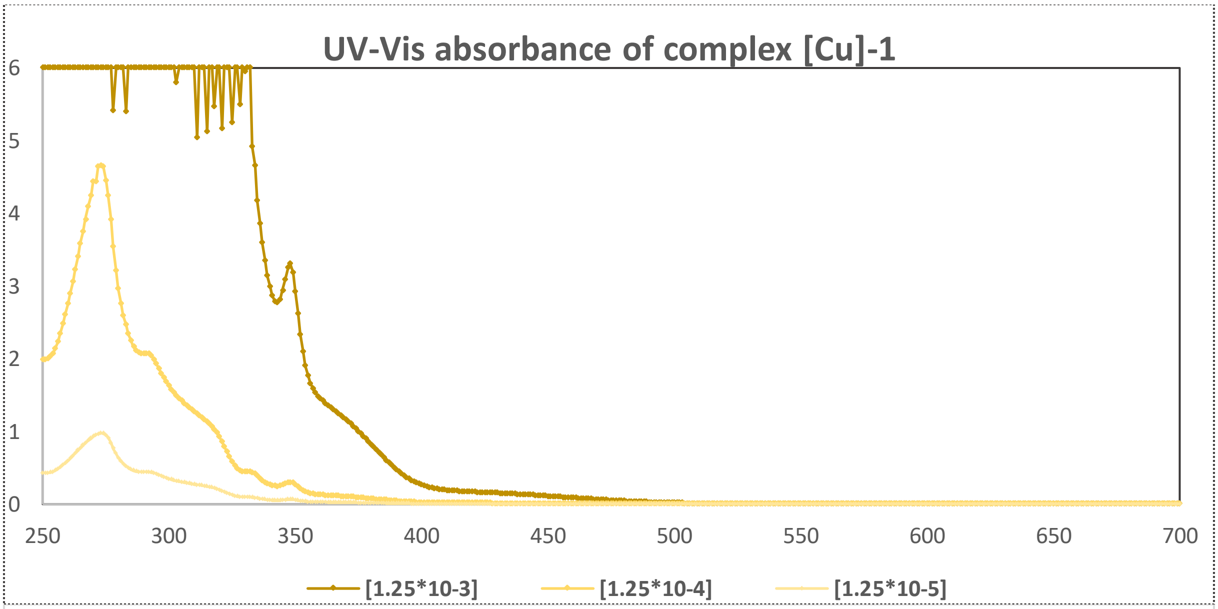
UV-Vis absorbance of complex [Cu]-1
0
1
2
3
4
5
6
250
300
350
400
450
500
550
600
650
700
[1.25*10-3]
[1.25*10-4]
[1.25*10-5]

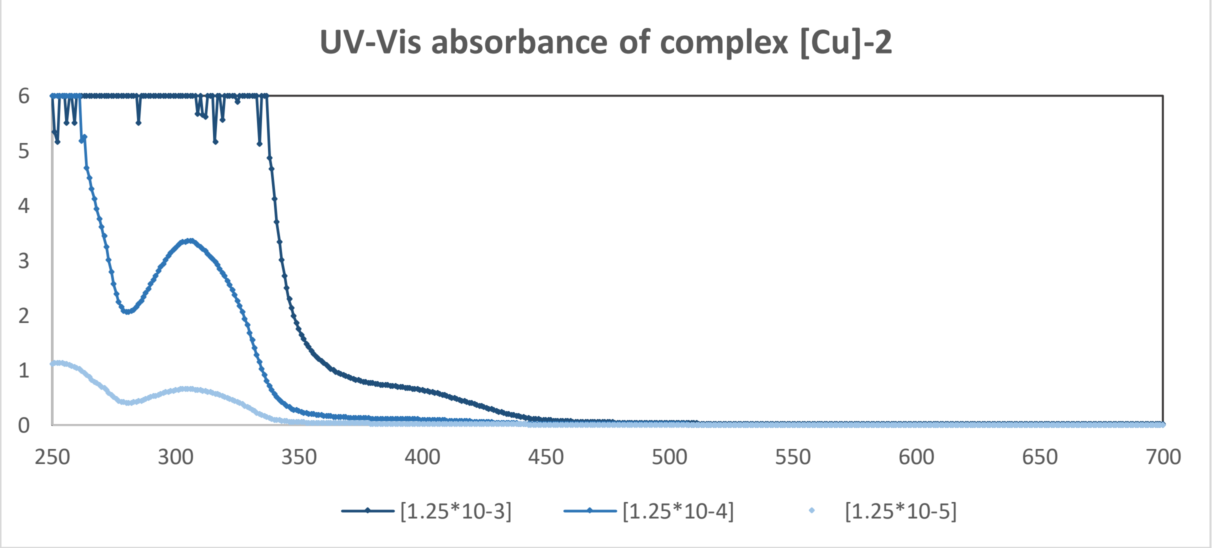
UV-Vis absorbance of complex [Cu]-2
0
1
2
3
4
5
6
250
300
350
400
450
500
550
600
650
700
[1.25*10-3]
[1.25*10-4]
[1.25*10-5]

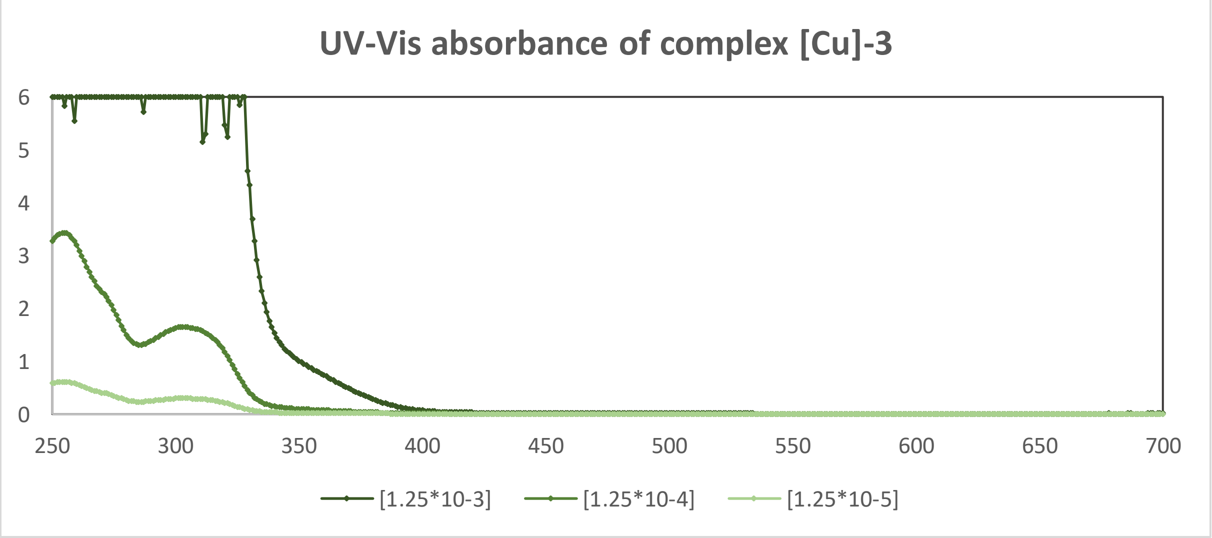
UV-Vis absorbance of complex [Cu]-3
0
1
2
3
4
5
6
250
300
350
400
450
500
550
600
650
700
[1.25*10-3]
[1.25*10-4]
[1.25*10-5]

3.2. Synthesis of *E*-stilbenes

Stilbenes **1-E**, **2-E**, **3-E** and **8-E** (as well as *E*-olefins **6-E**, **13-E**, **14-E**, **15-E**, **16-E**, **17-E** and **19-E**) were purchased from Sigma-Aldrich and TCI.

18-E was synthesized following a literature procedure (Mueller, D., Alexakis, A *Chem. Eur. J.* **2013**, 19, 45, 15226–15239)

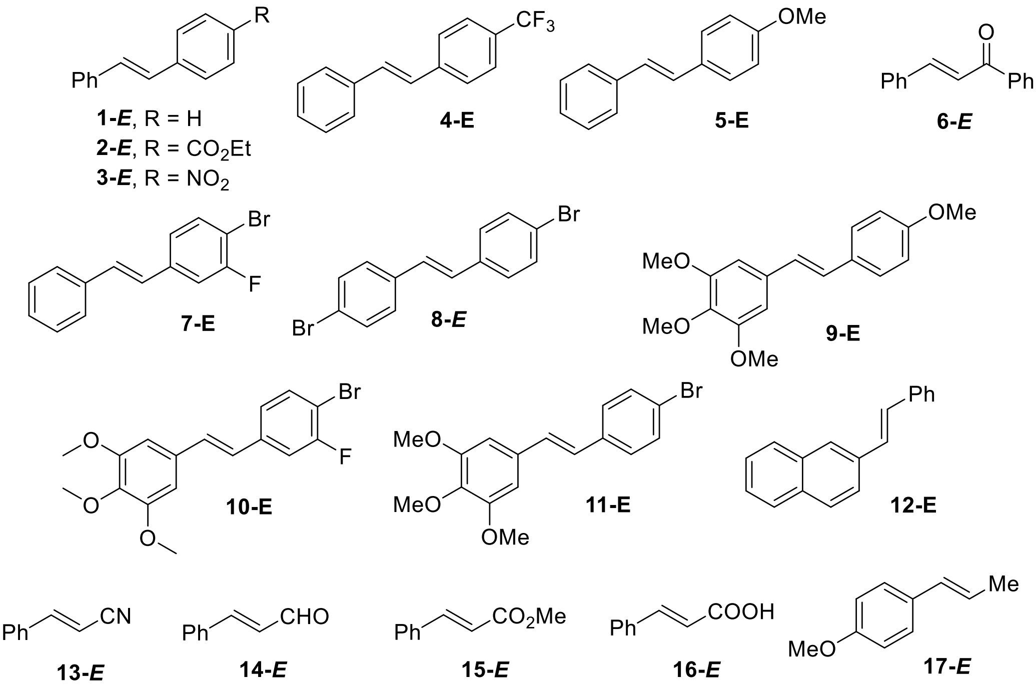

Remaining *E*-stilbenes were synthesized via Mizoroki-Heck coupling.

Synthesis of **S9**

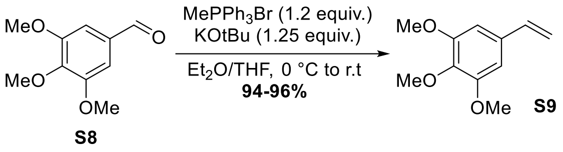

Methyltriphenylphosphonium bromide (4.28 g, 12.0 mmol) and PTFE-coated magnetic stirring bar was placed in 250 mL round-bottom flask under argon. Anhydrous Et_2O (80 mL) was added. Resulting suspension was stirred vigorously, then KOtBu (1.40 g, 12.5 mmol) was added in one portion. Mixture was stirred at r.t. for 15 min then flask was placed in water/ice bath and stirring continued for 30 min. Solution of 3,4,5-trimethoxybenzaldehyde (S3, 1.96 g, 10.0 mmol) in anhydrous THF (10 mL) was added dropwise (for about 10 min). Cooling bath was removed 15 min after dropwise addition was completed, stirring was continued for 60 min at r.t. when TLC analysis indicated full conversion of S3. Hexane (100 mL) was added, mixture was filtered through a pad of SiO_2 on a lSchott funnel (around 100 mL dry silica was used) that was then washed with hexane:Et_2O 1:1. Combined filtrates were concentrated in vacuo, residue was purified by a second filtration through a pad of SiO_2 on a Schott funnel (around 50 mL dry silica was used) that was washed with hexane:Et_2O 8:2 (150 mL). Filtrate was concentrated in vacuo to give 1.84 g (9.4 mmol, 94%) of **S9** as a colorless oil. Reaction was repeated once with 96% yield.

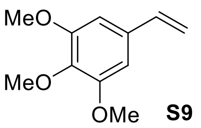

S9

^{1}H NMR (500 MHz, $CDCl_3$) δ 6.66 – 6.57 (m, 3H), 5.64 (d, *J* = 17.5 Hz, 1H), 5.19 (d, *J* = 10.8 Hz, 1H), 3.86 (s, 6H), 3.84 (s, 3H).

Literature NMR (*J. Org. Chem.* **2019**, *84*, 6, 3579–3589)

^{1}H NMR (500 MHz, $CDCl_3$) δ 6.68–6.62 (m, 3H), 5.67 (d, *J* = 17.5 Hz, 1H), 5.23 (d, *J* = 10.8 Hz, 1H), 3.90 (s, 6H), 3.86 (s, 3H).

General procedure for Mizoroki-Heck coupling (**procedure A**)

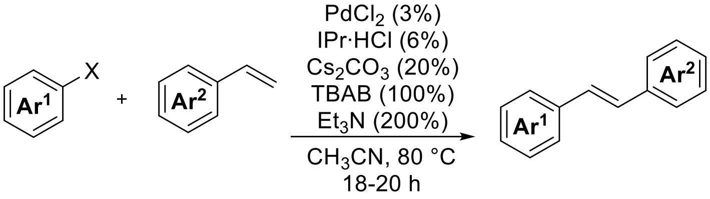

$PdCl_2$ (27 mg, 0.15 mmol, 0.03 equiv.) IPr·HCl (128 mg, 0.30 mmol, 0.06 equiv.), Cs_2CO_3 (326 mg, 1.0 mmol, 0.2 equiv.) and TBAB (1611 mg, 5.0 mmol, 1 equiv.) were placed in round bottom flask (50 mL) under argon along with PTFE-coated magnetic stirring bar. CH_3CN (5 mL, HPLC grade degassed by purging with argon for 20 min) was added, flask was sealed with rubber septum, mixture stirred at r.t. for 15 min and purged with the stream of argon through a needle piercing the septum. Et_3N (1.4 mL, 10.0 mmol, 2 equiv.) was added, purging continued for 10 min. Flask was then placed in pre-heated oil bath (50 °C), subsequently aryl halide (5.0 mmol) and vinylarene (5.0 mmol) were added in CH_3CN solution (5 mL, HPLC grade degassed by purging with argon for 20 min). Degassing was discontinued, septum was covered with parafilm. Bath temperature was increased to 80 °C, mixture was stirred vigorously (620 rpm) in this conditions overnight (18-20 h). After cooling to r.t. mixture was concentrated in vacuo to remove CH_3CN and excess Et_3N, residue was re-dissolved in AcOEt (100 mL) then washed with water (100 mL) and brine (100 mL) then dried over anhydrous Na_2SO_4. Drying agent was filtered off, solvents removed in vacuo, residue purified by flash chromatography (hexane/AcOEt 100:0 to 80:20).

(E)-1-styryl-4-(trifluoromethyl)benzene, **4-*E***

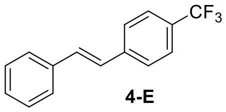

4-E

White solid, 410 mg (1.65 mmol, 33%) obtained starting from styrene and 4-trifluoromethyl-bromobenzene following **procedure A**.

^{1}H NMR (600 MHz, $CDCl_3$) δ = 7.61 (s, 4H), 7.54 (d, *J* = 7.2 Hz, 2H), 7.39 (t, *J* = 7.6 Hz, 2H), 7.31 (t, *J* = 7.4 Hz, 1H), 7.20 (d, *J* = 16.3 Hz, 1H), 7.12 (d, *J* = 16.3 Hz, 1H) ppm.

^{13}C{1H}NMR (126 MHz, $CDCl_3$) δ = 140.8, 136.6, 131.2, 129.3 (q, *J* = 32.3 Hz), 128.8, 128.3, 127.1, 126.8, 126.6, 125.6 (q, *J* = 3.9 Hz), 124.2 (q, *J* = 271.8 Hz) ppm.

Literature NMR[9] (*Angew. Chem. Int. Ed.* **2015**, 54, *48*, 14518-14522)
^{1}H NMR (500 MHz, $CDCl_3$) δ = 7.64 (s, 4 H), 7.57 (d, *J* = 7.6 Hz, 2 H), 7.41 (t, *J* =7.6 Hz, 2 H), 7.33 (t, *J* = 7.5 Hz, 1H), 7.23 (d, *J* = 16.3 Hz, 1 H), 7.15 (d, *J* = 16.3 Hz, 1 H) ppm.

^{13}C{1H} NMR (125 MHz, $CDCl_3$) δ = 140.8, 136.6, 131.2, 129.4, 129.0 (q, *J2* = 32.1 Hz),128.3, 127.1, 126.8, 126.6, 125.6 (q, *J3* = 3.7 Hz), 124.2 (q, *J1* = 270.0 Hz) ppm.

(E)-1-methoxy-4-styrylbenzene, **5-*E***

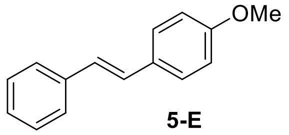

5-E

White solid, 445mg (2.12 mmol, 42%) obtained starting from styrene and 4-methoxy-iodobenzene following **procedure A**.

^{1}H NMR (500 MHz, $CDCl_3$) δ = 7.50 (d, *J* = 7.6 Hz, 2H), 7.47 (d, *J* = 8.4 Hz, 2H), 7.36 (t, *J* = 7.6 Hz, 2H), 7.25 (t, *J* = 7.1 Hz, 1H), 7.08 (d, *J* = 16.3 Hz, 1H), 6.99 (d, *J* = 16.3 Hz, 1H), 6.91 (d, *J* = 8.4 Hz, 2H), 3.84 (s, 3H) ppm.

^{13}C NMR (126 MHz $CDCl_3$) δ = 159.3, 137.6, 130.1, 128.6, 128.2, 127.7, 127.2, 126.6, 126.2, 114.1, 55.3 ppm.

Literature NMR[9] (*Angew. Chem. Int. Ed.* **2015**, 54, *48,* 14518-14522):

^{1}H NMR (500 MHz, $CDCl_3$) δ = 7.52 (d, *J* = 7.7 Hz, 2 H), 7.49 (d, *J* = 8.2 Hz, 2 H), 7.38 (t, *J* = 7.6 Hz, 2 H), 7.26 (t, *J* = 7.3 Hz, 1 H), 7.10 (d, *J* = 16.3 Hz, 1 H), 7.01 (d, *J* = 16.3 Hz, 1 H), 6.94 (d, *J* = 8.2 Hz, 2 H), 3.86 (s, 3 H) ppm.

^{13}C{1H} NMR (125 MHz, $CDCl_3$) δ 159.3, 137.7, 130.2, 128.6, 128.2, 127.7, 127.2, 126.7, 126.3, 114.2, 55.3 ppm.

(E)-1-bromo-2-fluoro-4-styrylbenzene, **7-E**

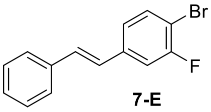

7-E

White solid, 800 mg (2.89 mmol, 58%) obtained starting from styrene and 4-bromo-3-fluoro-iodobenzene following **procedure A**.

^{1}H NMR (500 MHz, $CDCl_3$) δ = 7.55 – 7.47 (m, 3H), 7.38 (dd, *J* = 8.4, 6.9 Hz, 2H), 7.31 (t, *J* = 7.3 Hz, 1H), 7.27 (dd, *J* = 10.0, 1.9 Hz, 1H), 7.15 (dd, *J* = 8.3, 2.0 Hz, 1H), 7.11 (d, *J* = 16.3 Hz, 1H), 7.00 (d, *J* = 16.2 Hz, 1H) ppm.

^{13}C{1H} NMR (126 MHz, $cdcl_3$) δ = 160.3, 158.3, 139.0, 138.9, 136.5, 133.5, 133.5, 130.6, 128.8, 128.2, 126.7, 126.5, 126.4, 123.4, 123.4, 113.9, 113.7, 107.7, 107.5 ppm. (signal splitting observed due to a carbon-fluorine coupling, all peaks reported without a multiplet analysis)

HRMS (APCI-TOF) m/z calcd for $[M]^+$ $C_{14}H_{10}BrF$: 275.9950; found: 275.9949.

IR (film, DCM): ν 3024(m), 1891(w), 1564(s), 1480(s), 1450(s), 1409(s), 1281(s), 1237(s), 1037(s), 970(vs), 949(s), 882(s), 814(vs), 758(vs), 695(vs), 605(s), 496(m) cm^{-1}

(E)-1,2,3-trimethoxy-5-(4-methoxystyryl)benzene, **9-E**

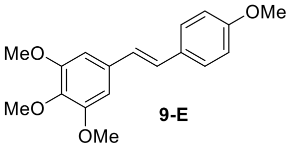

9-E

White solid, 698 mg (2.32 mmol, 46%) obtained starting from 3,4,5-trimethoxyvinylbenzene (**S9**) and 4-methoxy-iodobenzene following modified procedure A: DMF was used instead of CH_3CN, overnight reaction ran at 120 °C. Regular procedure A resulted in a very low yield (<10%).

^{1}H NMR (600 MHz, $CDCl_3$) δ 7.45 (d, *J* = 8.6 Hz, 2H), 6.97 (d, *J* = 16.3 Hz, 1H), 6.93 – 6.87 (m, 3H), 6.72 (s, 2H), 3.92 (s, 6H), 3.87 (s, 3H), 3.83 (s, 3H).

^{13}C{1H}NMR (126 MHz $CDCl_3$) δ 159.2, 153.3, 137.6, 133.4, 129.9, 127.7, 127.6, 126.5, 114.1, 114.1, 103.3, , 60.9, 56.0, 55.2ppm.

Literature NMR[10] (*Chem. Eur. J.* **2015**, 21, *24,* 8737–8740):
^{1}H NMR (400 MHz, $CDCl_3$): δ = 7.45 (d, *J* = 8.7 Hz, 2H), 6.98 (d, *J* = 16.2 Hz, 1H), 6.93 – 6.87 (m, 3H), 6.72 (s, 2H), 3.92 (s, 6H), 3.86 (s, 3H), 3.83 (s, 3H) ppm.

^{13}C{1H} NMR (100 MHz, $CDCl_3$): δ = 159.4, 153.5, 137.7, 133.6, 130.1, 127.9, 127.8, 126.7, 114.3, 103.4, 61.1,56.2, 55.6 ppm.

(E)-5-(4-bromo-3-fluorostyryl)-1,2,3-trimethoxybenzene, **10-E**

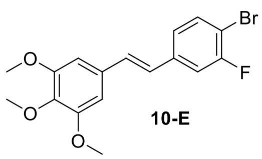

10-E

Yellow solid, 1338 mg (3.64 mmol, 73%) obtained starting from **S9** and 4-bromo-3-fluoro-iodobenzene, following procedure A.

^{1}**H NMR** (600 MHz, $CDCl_3$) δ 7 =.51 (dd, *J* = 8.3, 7.2 Hz, 1H), 7.26 (dd, *J* = 9.9, 2.0 Hz, 1H), 7.14 (dd, *J* = 8.3, 2.0 Hz, 1H), 7.03 (d, *J* = 16.2 Hz, 1H), 6.90 (d, *J* = 16.2 Hz, 1H), 6.73 (s, 2H), 3.92 (s, 6H), 3.88 (s, 3H) ppm.

^{13}**C**{1H} **NMR** (126 MHz, $CDCl_3$) δ = 160.3, 158.3, 153.5, 138.9, 138.8, 138.5, 133.5, 132.2, 130.6, 125.9, 125.8, 123.3, 123.3, 113.7, 113.6, 107.6, 107.4, 103.8, 77.3, 60.9, 56.1 ppm. (signal splitting observed due to a carbon-fluorine coupling, all peaks reported without a multiplet analysis)

HRMS (ESI-TOF) m/z calcd for $[M]^+$ $C_{17}H_{16}BrFO_3$: 367.0345; found: 367.0344

IR (film, DCM): 2938(br), 2837(m), 1634(w), 1581(vs), 1506(vs), 1421(vs), 1342(s), 1239(s), 1127(vs), 1006(s), 969(m), 819(m), 689(m), 637(m), 548(w), 445(w) cm^{-1}

(E)-5-(4-bromostyryl)-1,2,3-trimethoxybenzene, **11-*E***

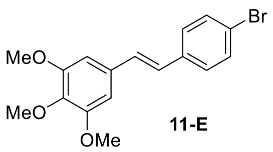

11-E

White solid, 1256 mg (3.60 mmol, 72%) obtained starting from **S9** and 4-bromo-iodobenzene, following **procedure A**.

^{1}**H NMR** (600 MHz, $CDCl_3$) δ = 7.48 (d, *J* = 8.2 Hz, 2H), 7.37 (d, *J* = 8.1 Hz, 2H), 7.02 (d, *J* = 16.2 Hz, 1H), 6.93 (d, *J* = 16.2 Hz, 1H), 6.73 (s, 2H), 3.92 (s, 6H), 3.87 (s, 3H).

^{13}**C**{1H} **NMR** (126 MHz, $CDCl_3$) δ = 153.4, 138.2, 136.2, 132.7, 131.8, 129.4, 127.9, 126.9, 121.3, 103.7, 60.9, 56.1 ppm

Literature NMR[10] (*Chem. Eur. J.* **2015**, 21, *24*, 8737–8740):

^{1}**H NMR** (400 MHz, $CDCl_3$): δ = 7.47 (d, *J* = 8.4 Hz, 2H), 7.36 (d, *J* = 8.4 Hz, 2H), 7.02 (d, *J* = 16.4 Hz, 1H), 6.93 (d, *J* = 16.4 Hz, 1H), 6.73 (s, 2H), 3.91 (s, 6H), 3.87 (s, 3H) ppm.

^{13}**C**{1H} **NMR** (100 MHz, $CDCl_3$): δ 153.4, 138.2, 136.1, 132.6, 131.8, 129.3, 127.8, 126.8, 121.2, 103.6, 60.9, 56.1 ppm

(E)-2-styrylnaphthalene, **12-E**

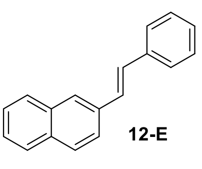
12-E

Off-white solid, 703 mg (3.05 mmol, 61%) obtained starting from styrene and 2-bromonaphthalene, following procedure A.

^{1}**H NMR** (500 MHz, $CDCl_3$) δ 7.93 – 7.82 (m, 4H), 7.79 (dd, *J* = 8.6, 1.8 Hz, 1H), 7.61 (d, *J* = 7.3 Hz, 2H), 7.55 – 7.46 (m, 2H), 7.43 (t, *J* = 7.6 Hz, 2H), 7.35 – 7.27 (m, 3H).

^{13}**C**{1H} **NMR** (126 MHz, $CDCl_3$) δ 137.4, 134.8, 133.7, 133.0, 129.0, 128.8, 128.7, 128.3, 128.0, 127.7, 127.7, 126.6, 126.3, 125., 123.5 ppm. Literature NMR[11] (*J. Am. Chem. Soc.* **2014**, 136, *26*, 9260–9263)

^{1}**H NMR** (400 MHz, $CDCl_3$): δ = 7.86–7.80 (m, 4H), 7.76–7.74 (m, 1H),7.57 (d, *J* = 7.6 Hz, 2H), 7.50–7.43 (m, 2H), 7.39 (t, *J* = 7.6 Hz, 2H), 7.31–7.25 (m, 3H) ppm.

^{13}C{1H} **NMR** (100 MHz, $CDCl_3$): δ = 137.4, 134.8, 133.7, 133.1, 129.1, 128.79, 128.75, 128.3, 128.0, 127.7, 126.6, 126.6, 126.4, 125.9, 123.5 ppm.

4. PHOTOREACTION PROCEDURES

4.1. Energy transfer reactions – E→ Z photoisomerization

4.1.1. General procedure for photoisomerization

Catalyst and *E*-olefin were placed in 10 mL glass vial along with PTFE-coated magnetic stirring bar. Solvent (freshly distilled or HPLC grade) was added, vial was capped. In case of reactions run under inert gas conditions there was an additional step of degassing by purging with argon through a needle piercing the septum for 5 min. Vial was then placed in photoreactor. In case of the optimal UOSlab photoreactor cooling plate was set to 8 °C and LED power either 12.5 or 25W (50% or 100% power), blue (λ_{max} = 450 nm) or violet (λ_{max} = 405 nm) light, stirring set to 700-800 rpm. Z/E ratio was determined using GC analysis of 0.05-0.1 mL samples of reaction mixture, in most cases crude ^{1}H NMR analysis with an internal standard (1,3,5-trimethoxybenzene, **TMB**, 0.33 equiv.) added after reaction was performed to confirm formation of Z isomers and rule out formation of side products.

Below ^{1}H NMR spectrum of the crude reaction mixture of **11-*E*** photoisomerization with the assignment of **11-*E***, **11-*Z*** and **TMB** along with some signals coming from **[Cu]-1** and solvents

Crude reaction mixture of **11-*E***

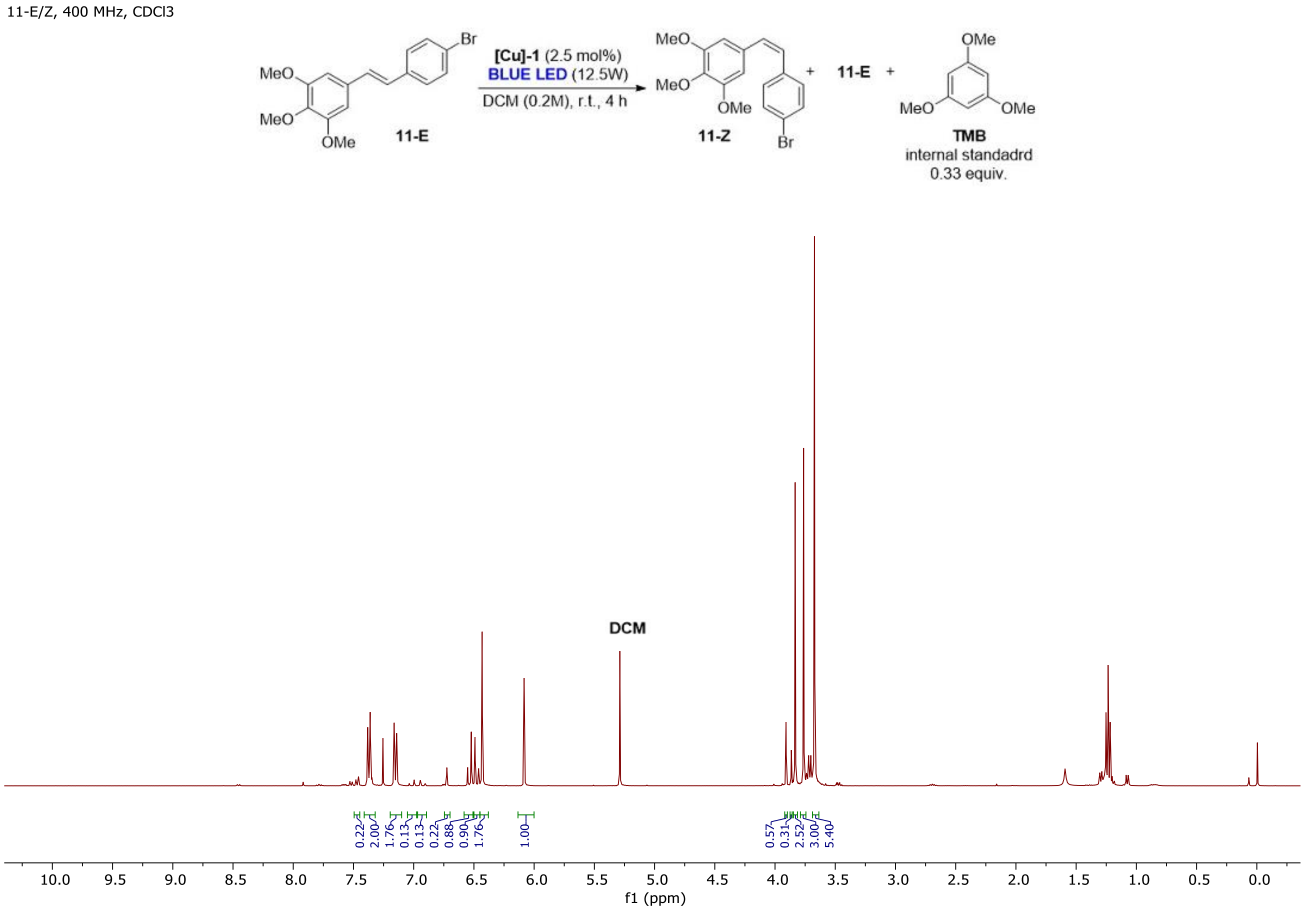

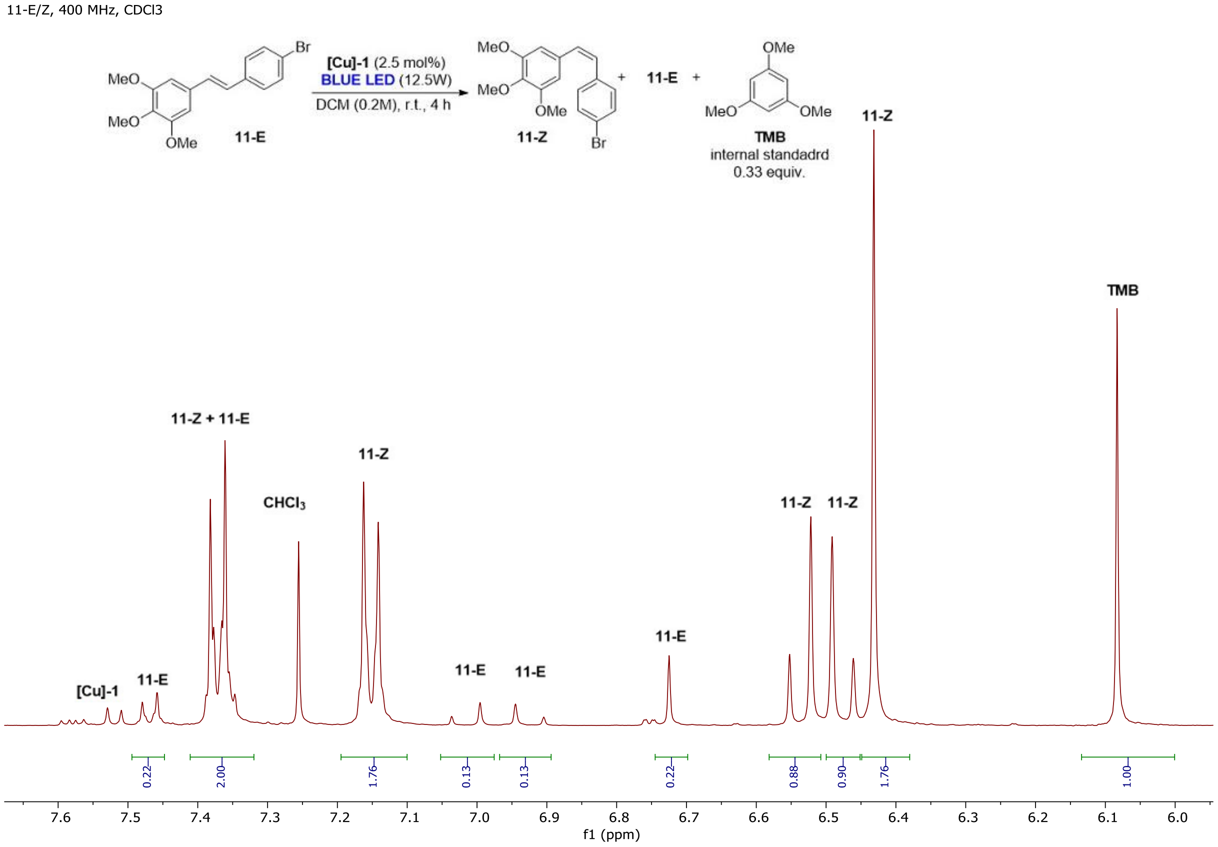

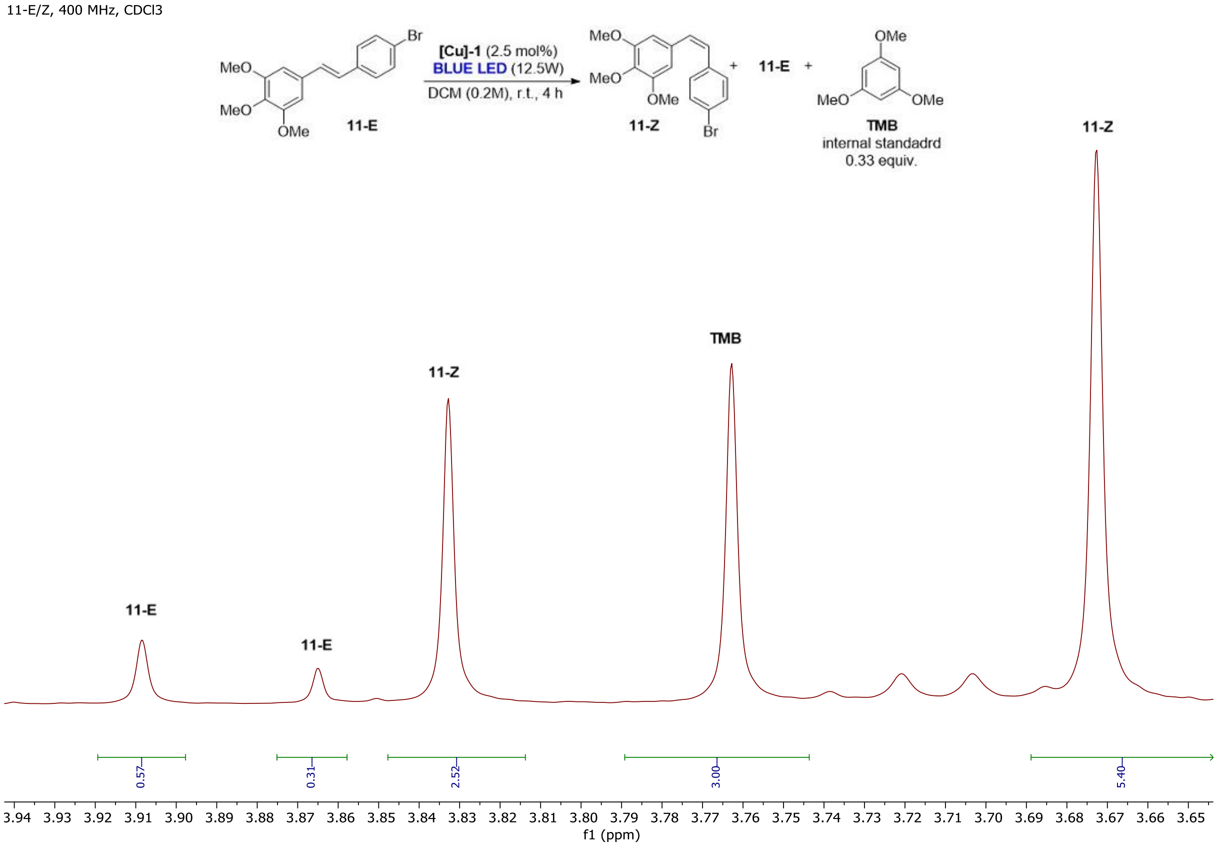

Crude reaction mixture of **1-*E***

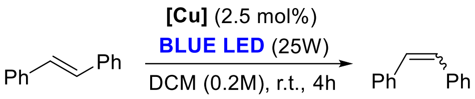

1-*E* **1-*Z* + 1-*E***

Literature NMR data for **1-E** (*Org. Lett.* **2014**, 16, 11, 3020–3023).

^{1}H NMR (400 MHz, $CDCl_3$): δ = **7.54-7.56 (m, 4H)**, **7.37-7.40 (m, 4H)**, 7.26-7.31 (m, 2H), **7.14 (s, 2H)**.

Literature NMR data for **1-Z** (*Org. Lett.* **2016**, 18, 23, 6196–6199)

^{1}H NMR (400 MHz, $CDCl_3$) δ 7.38 – 7.17 (m, 10H), **6.66 (s, 2H)**.

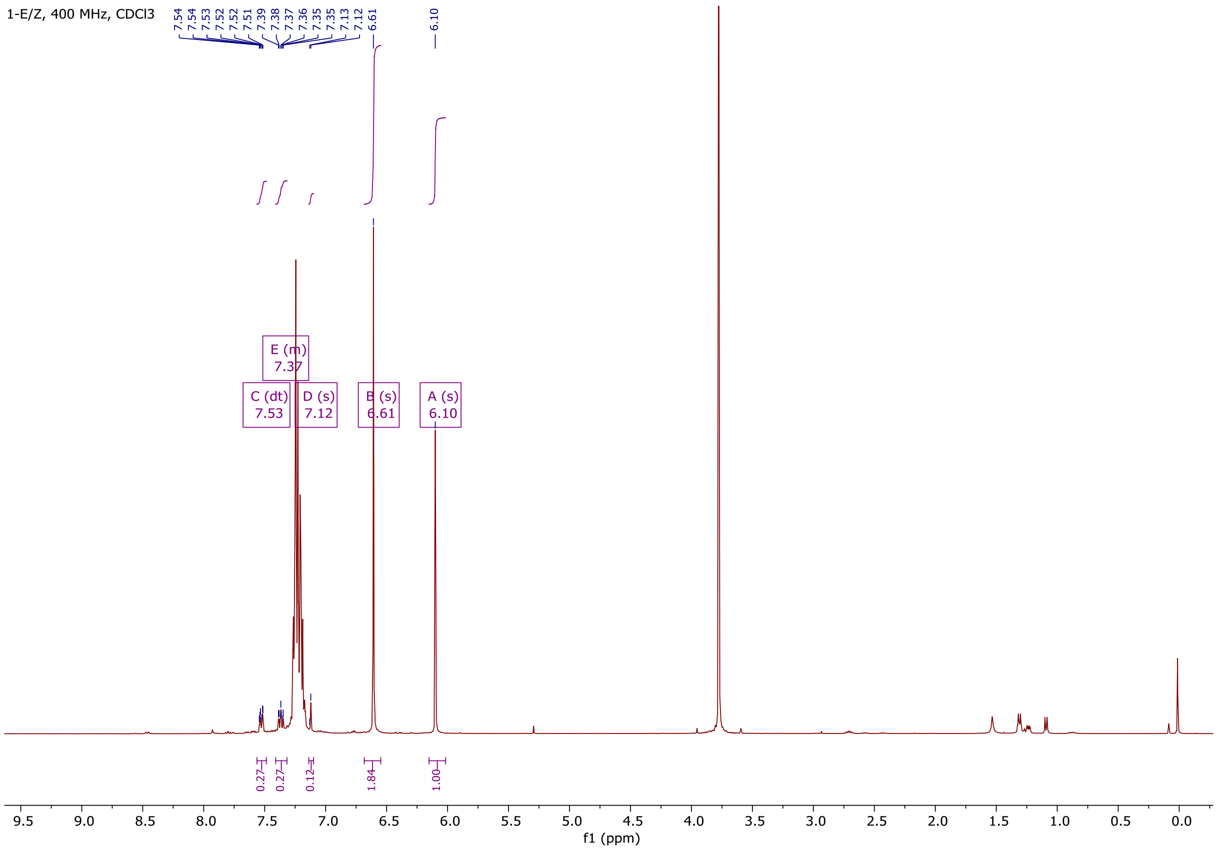

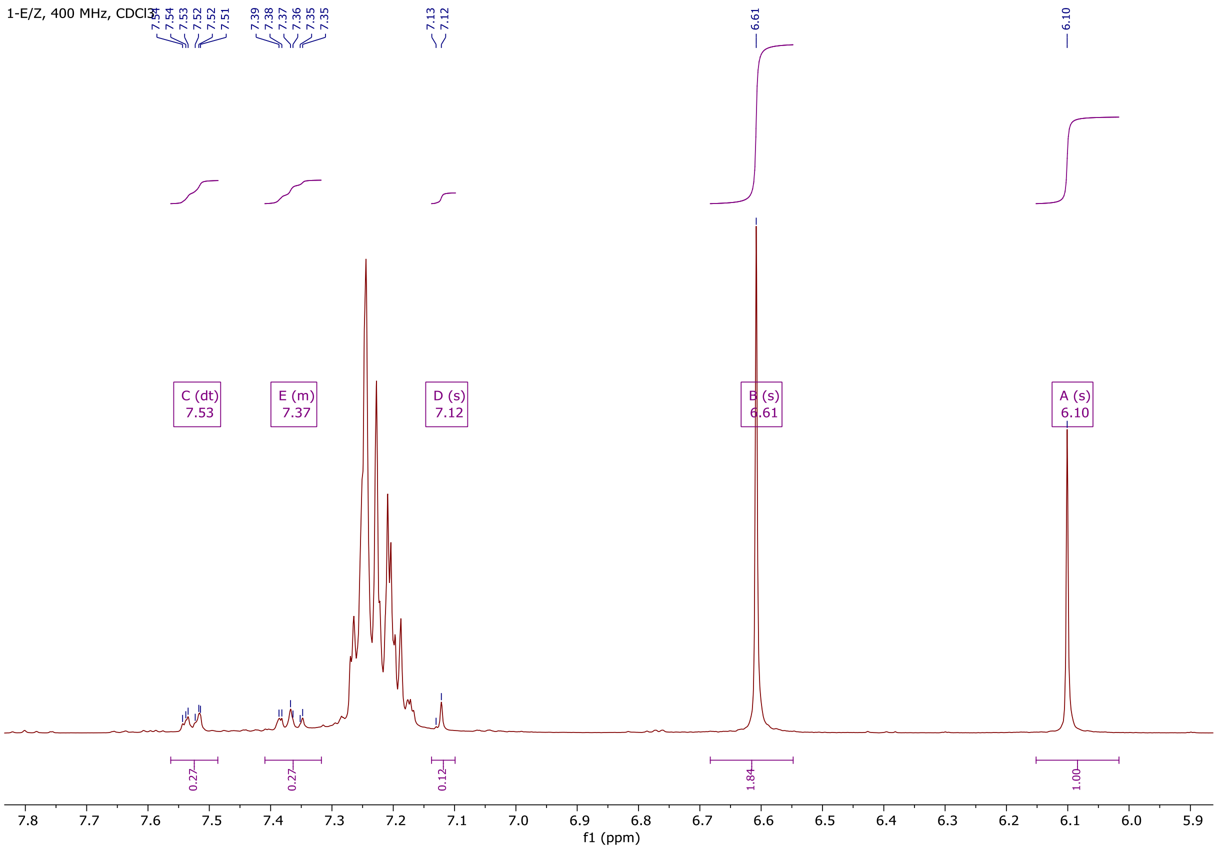

Crude reaction mixture of **2-*E***

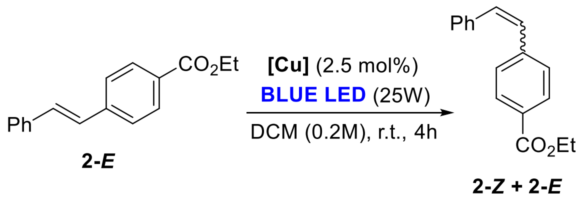

Literature NMR data for **2-E** (*Org. Lett.* **2019**, 21, 5, 1412–1416).

^{1}H NMR (400 MHz, CDCl3) δ **8.02 (d, J = 7.7 Hz, 2H)**, **7.61 – 7.46 (m, 4H)**, 7.36 (t, J = 7.4 Hz, 2H), 7.26 (dd, J = 24.0, 8.4 Hz, 1H), 7.20 – 7.06 (m, 2H), 4.37 (dd, J = 14.4, 7.2 Hz, 2H), 1.39 (t, J = 7.0 Hz, 3H).

Literature NMR data for **2-Z** (*Org. Lett.* **2019**, 21, 5, 1412–1416)

^{1}H NMR (400 MHz, CDCl3) δ **7.91 (d, J = 7.9 Hz, 2H)**, 7.31 (d, J = 7.9 Hz, 2H), 7.23 (s, 5H), **6.71 (d, J = 12.3 Hz, 1H)**, **6.61 (d, J = 12.2 Hz, 1H)**, 4.36 (q, J = 6.8 Hz, 2H), 1.39 (t, J = 7.0 Hz, 3H).

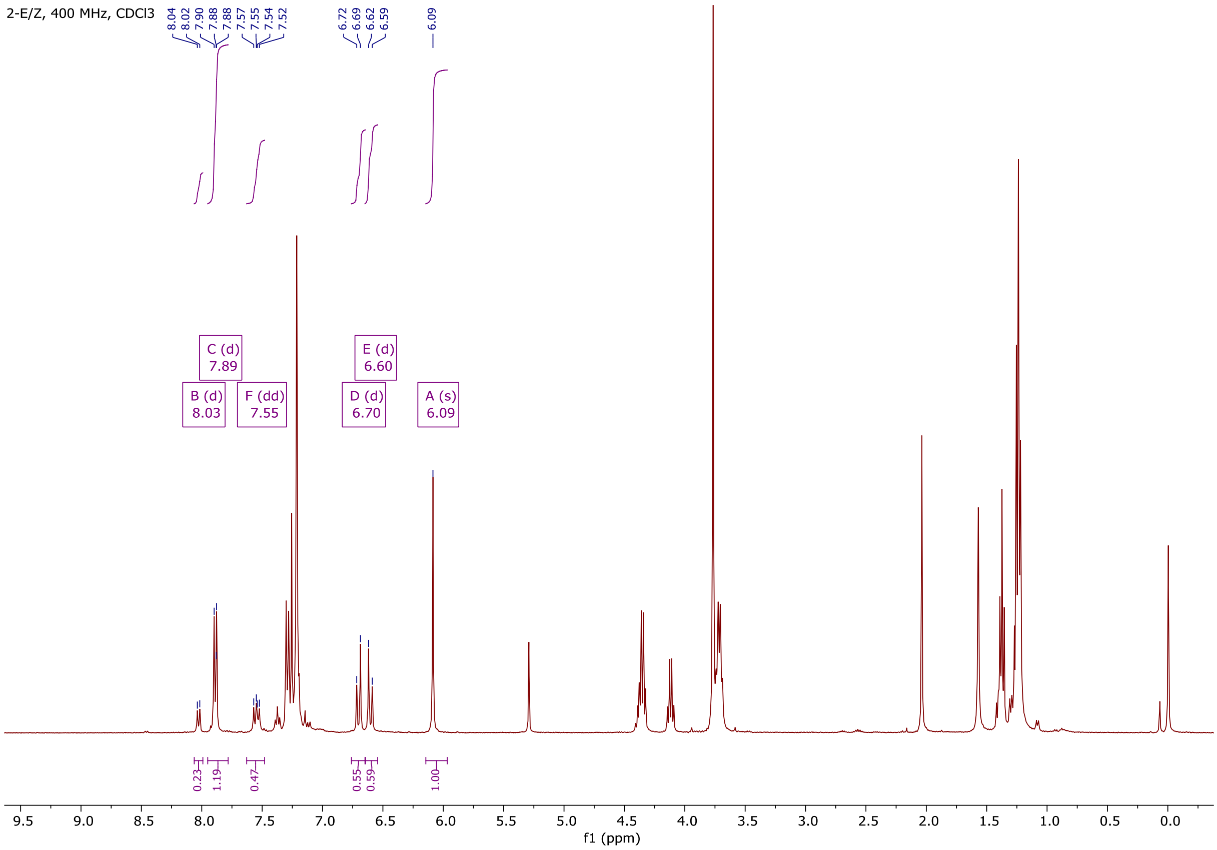
2-E/Z, 400 MHz, CDCl3
8.04
8.02
7.90
7.88
7.88
7.57
7.55
7.54
7.52
6.72
6.69
6.62
6.59
6.09
C (d)
7.89
E (d)
6.60
B (d)
8.03
F (dd)
7.55
D (d)
6.70
A (s)
6.09
0.23
1.19
0.47
0.55
0.59
1.00
f1 (ppm)

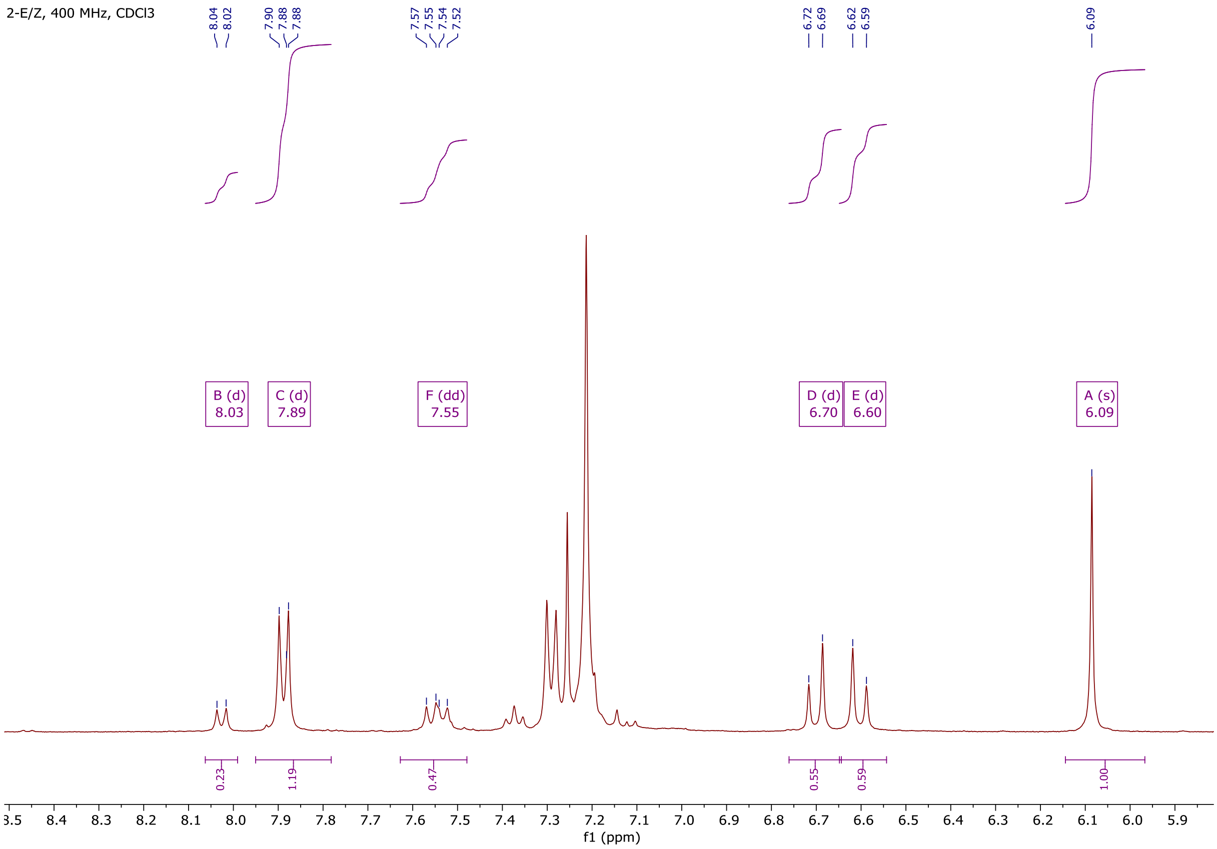
2-E/Z, 400 MHz, CDCl3
8.04
8.02
7.90
7.88
7.88
7.57
7.55
7.54
7.52
6.72
6.69
6.62
6.59
6.09
B (d)
8.03
C (d)
7.89
F (dd)
7.55
D (d)
6.70
E (d)
6.60
A (s)
6.09
0.23
1.19
0.47
0.55
0.59
1.00
f1 (ppm)

Crude reaction mixture of **3-*E***

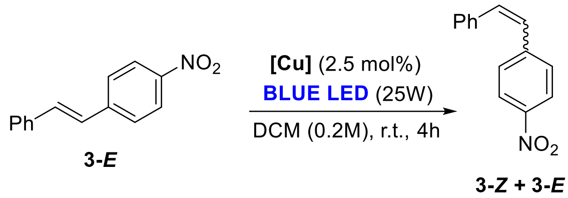

Literature NMR data for **3-E** (*Org. Lett.* **2016**, 18, 23, 6196–6199).

^{1}H NMR (400 MHz, $CDCl_3$) δ **8.21 (d, J = 8.9 Hz, 2H)**, 7.62 (d, J = 8.8 Hz, 2H), 7.54 (d, J = 7.1 Hz, 2H), 7.41 (t, J = 7.1 Hz, 2H), 7.34 (t, J = 7.2 Hz, 1H), 7.20 (d, J = 16.3 Hz, 2H).

Literature NMR data for **3-Z** (*J. Am. Chem. Soc.* **2019**, 141, 15, 6152–6156)

1H NMR (500 MHz, CDCl3): δ = **8.17 (d, J = 8.7 Hz, 2H)**, 7.74 (d, J = 8.7 Hz, 1H), 7.66 (d, J =8.1 Hz, 1H), 7.48 (d, J = 8.7 Hz, 2H), 7.37-7.35 (m, 2H), 7.32-7.30 (m, 1H), 6.92 (d, J = 11.2 Hz,1H), 6.72 (d, J = 12.4 Hz, 1H)

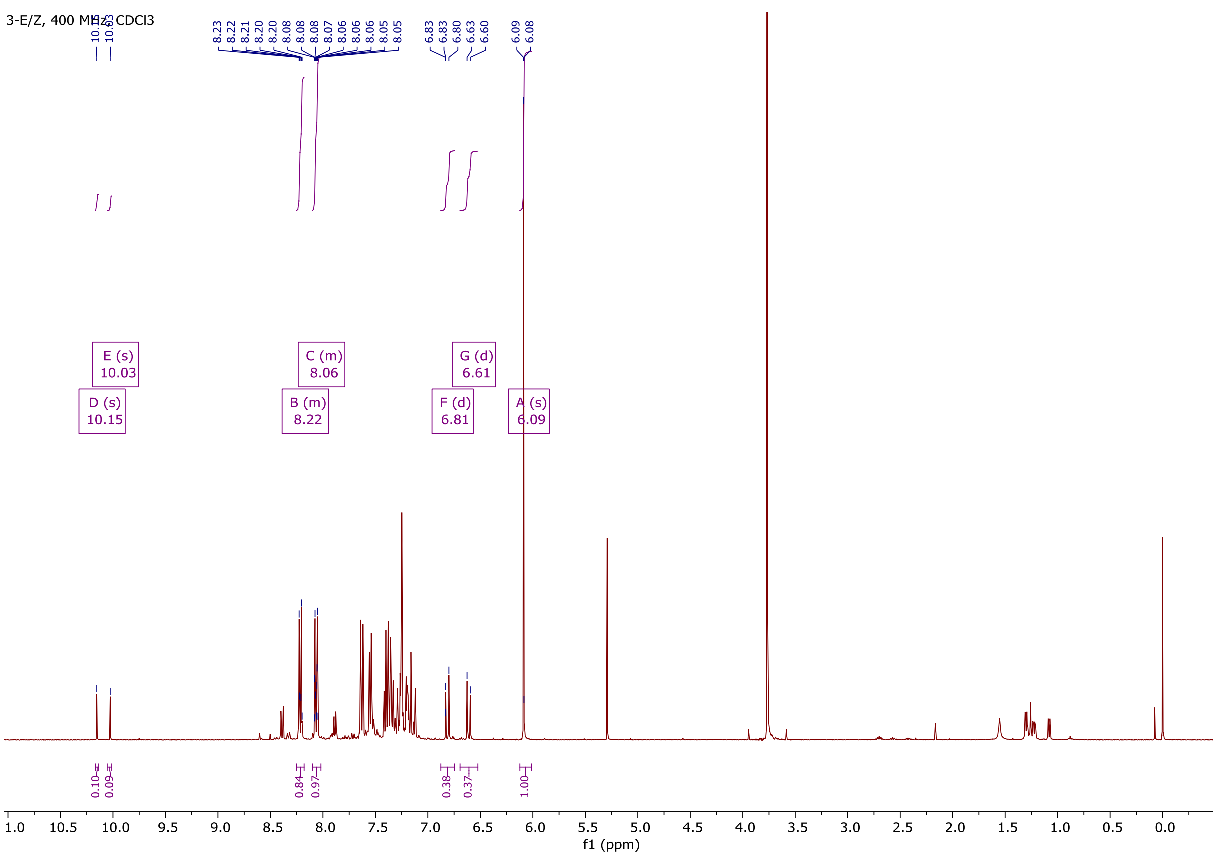

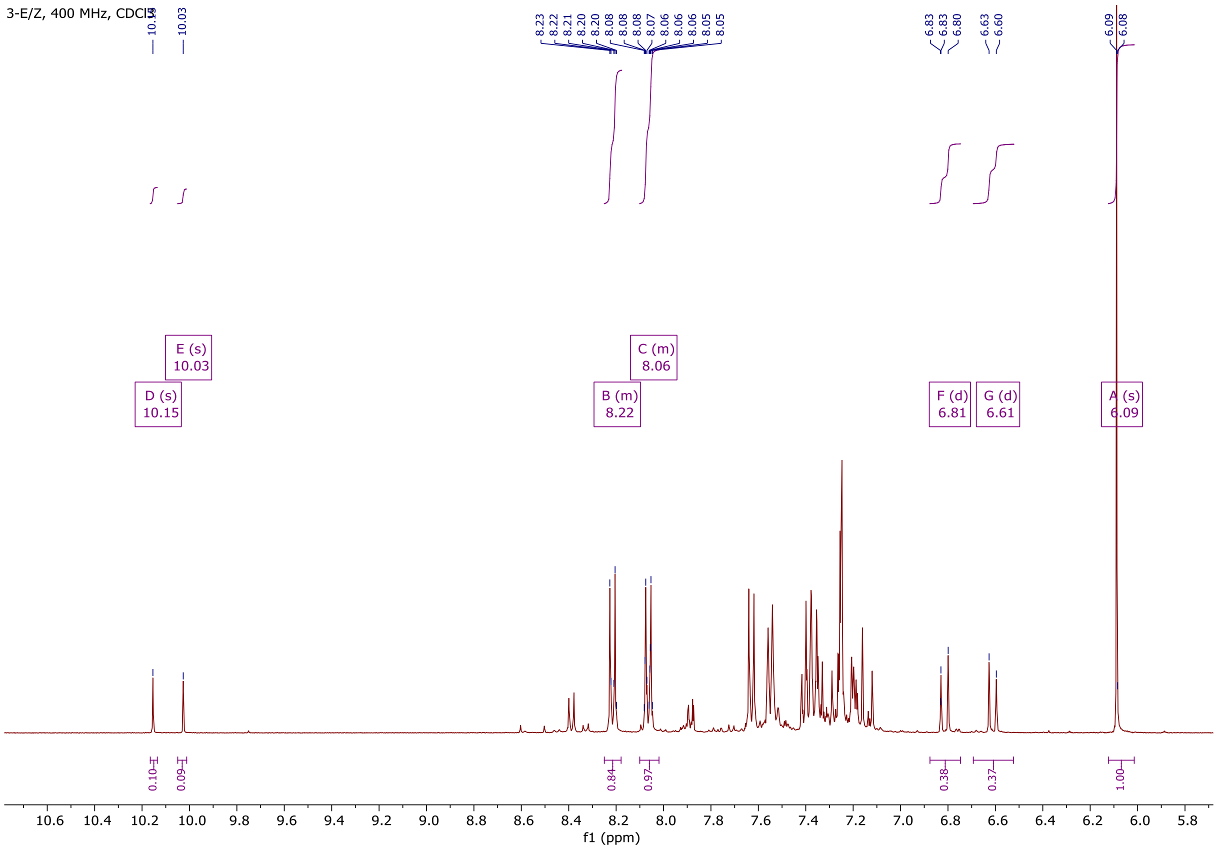

Crude reaction mixture of **4-*E***

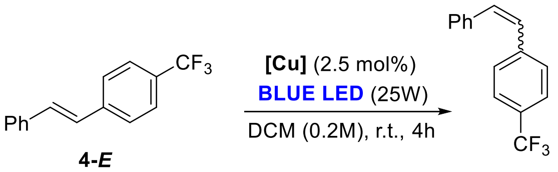

4-*E*

4-*Z* + 4-*E*

Literature NMR data for **4-E** (*JACS Au* **2021**, 1, 3, 308–315).

^{1}H NMR (400 MHz, $CDCl_3$): d 7.64-7.59 (m, 4H), **7.55 (d, J = 8 Hz, 2H)**, **7.40 (t, J = 8 Hz, 2H)**, 7.32 (t, J = 8 Hz, 1H), 7.21 (d, J = 16 Hz, 1H), 7.13 (d, J = 16 Hz, 1H)

Literature NMR data for **4-Z** (*Adv. Synth. Catal.* **2023**, 365, 1505)

^{1}H NMR (400 MHz, $CDCl_3$) δ (ppm) **7.45 (d, J = 8.2 Hz, 2H)**, **7.32 (d, J = 8.2 Hz, 2H)**, 7.18-7.26 (m, 5H), **6.70 (d, J = 12.4 Hz, 1H)**, **6.57 (d, J = 12.1 Hz, 1H)**.

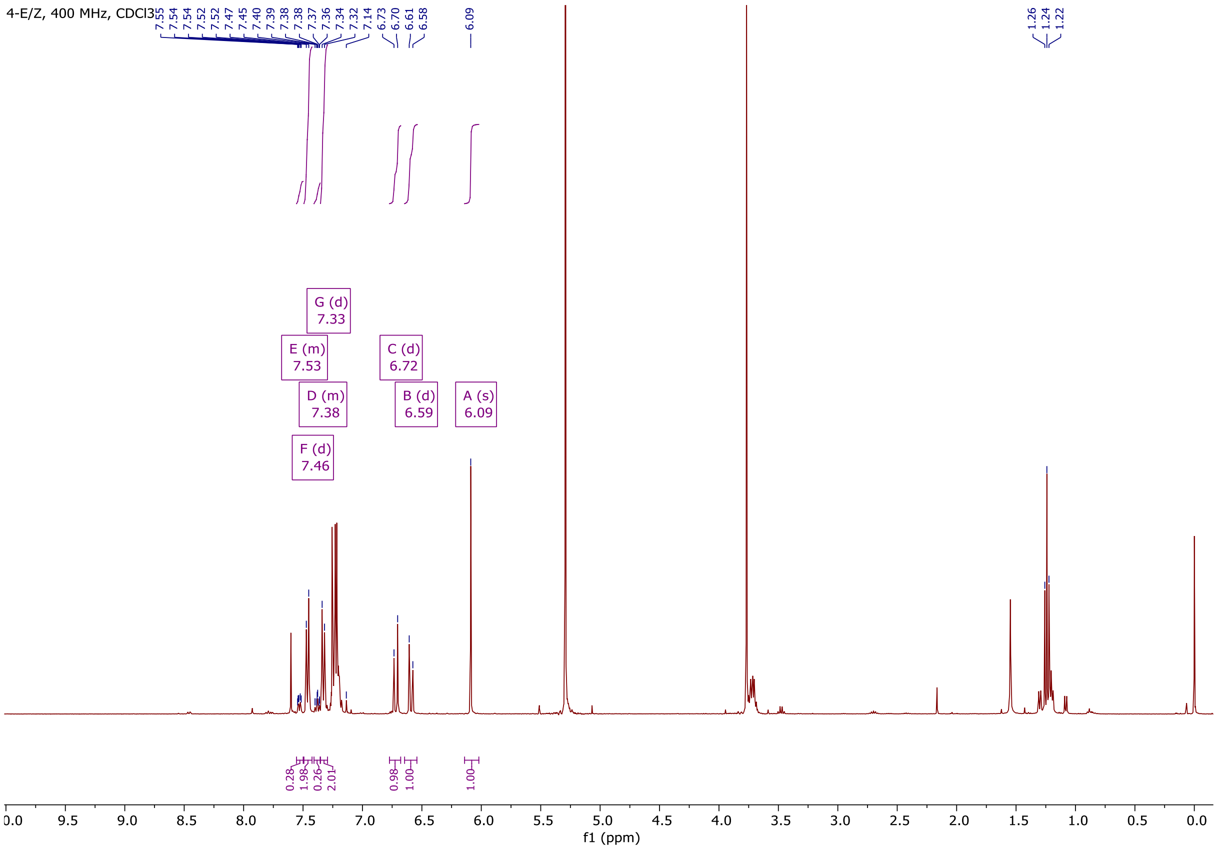
4-E/Z, 400 MHz, CDCl3
E (m)
7.53
F (d)
7.46
D (m)
7.38
G (d)
7.33
C (d)
6.72
B (d)
6.59
A (s)
6.09
f1 (ppm)

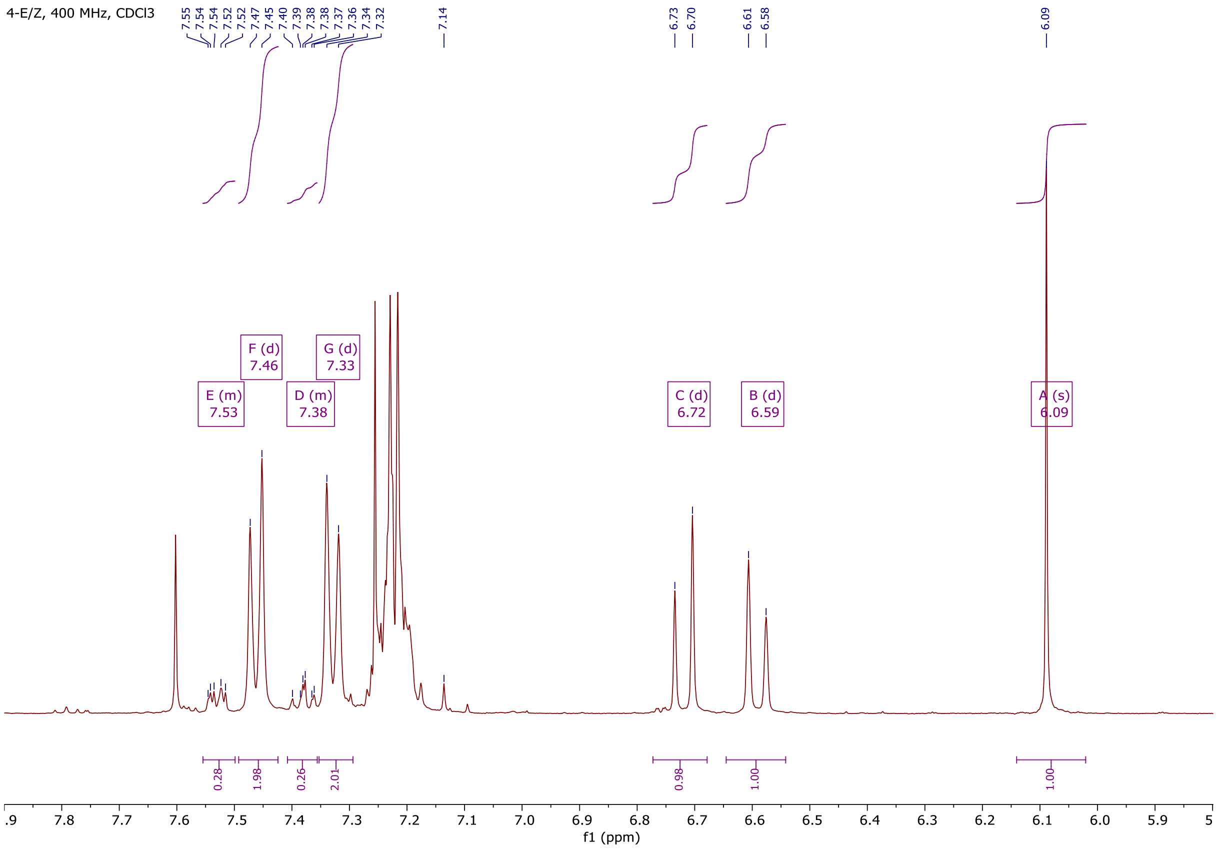
4-E/Z, 400 MHz, CDCl3
E (m)
7.53
F (d)
7.46
D (m)
7.38
G (d)
7.33
C (d)
6.72
B (d)
6.59
A (s)
6.09
f1 (ppm)

Crude reaction mixture of **5-*E***

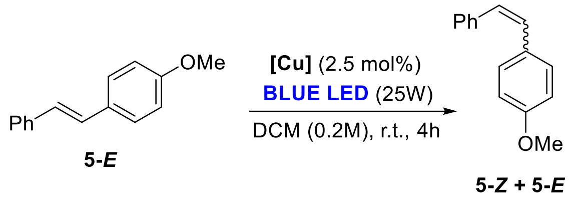

Literature NMR data for **5-E** (*J. Am. Chem. Soc.* 2021, 143, 9, 3373–3382)

^{1}H NMR (500 MHz, $CDCl_3$) δ 7.50 (dd, J =18.3, 8.1 Hz, 4H), 7.37 (t, J = 7.5 Hz, 2H), 7.26 (t, J = 7.1 Hz, 1H), **7.09 (d, J = 16.3 Hz, 1H), 7.00 (d, J =16.3 Hz, 1H), 6.93 (d, J = 8.4 Hz, 2H)**, 3.85 (s, 3H).

Literature NMR data for **5-Z** (*J. Am. Chem. Soc.* 2012, 134, 41, 16951–16954)

^{1}H NMR (500 MHz, $CDCl_3$):δ 7.28–7.22 (m, 5H), 7.20–7.17 (m, 2H), **6.76–6.74 (m, 2H)**, **6.55–6.49 (m, 2H)**, 3.78 (s, 3H)

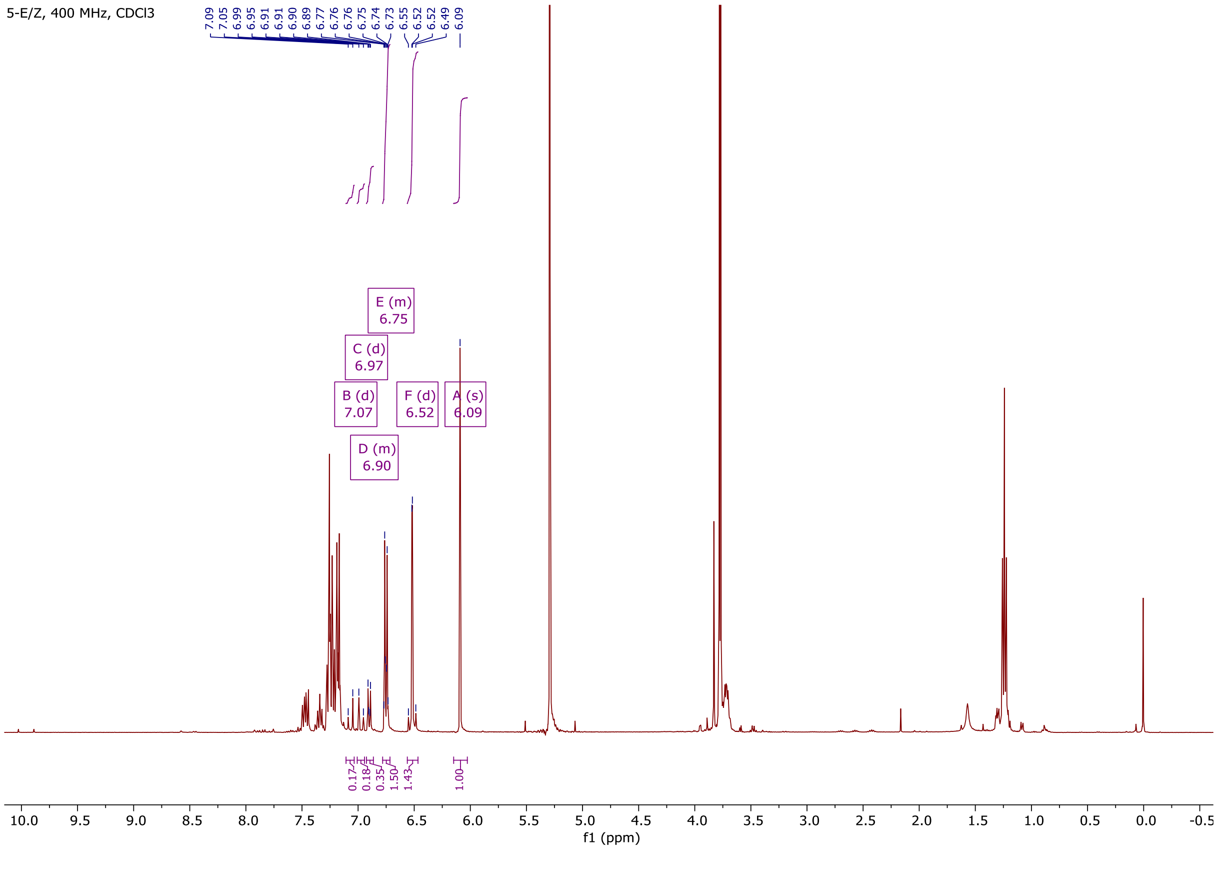

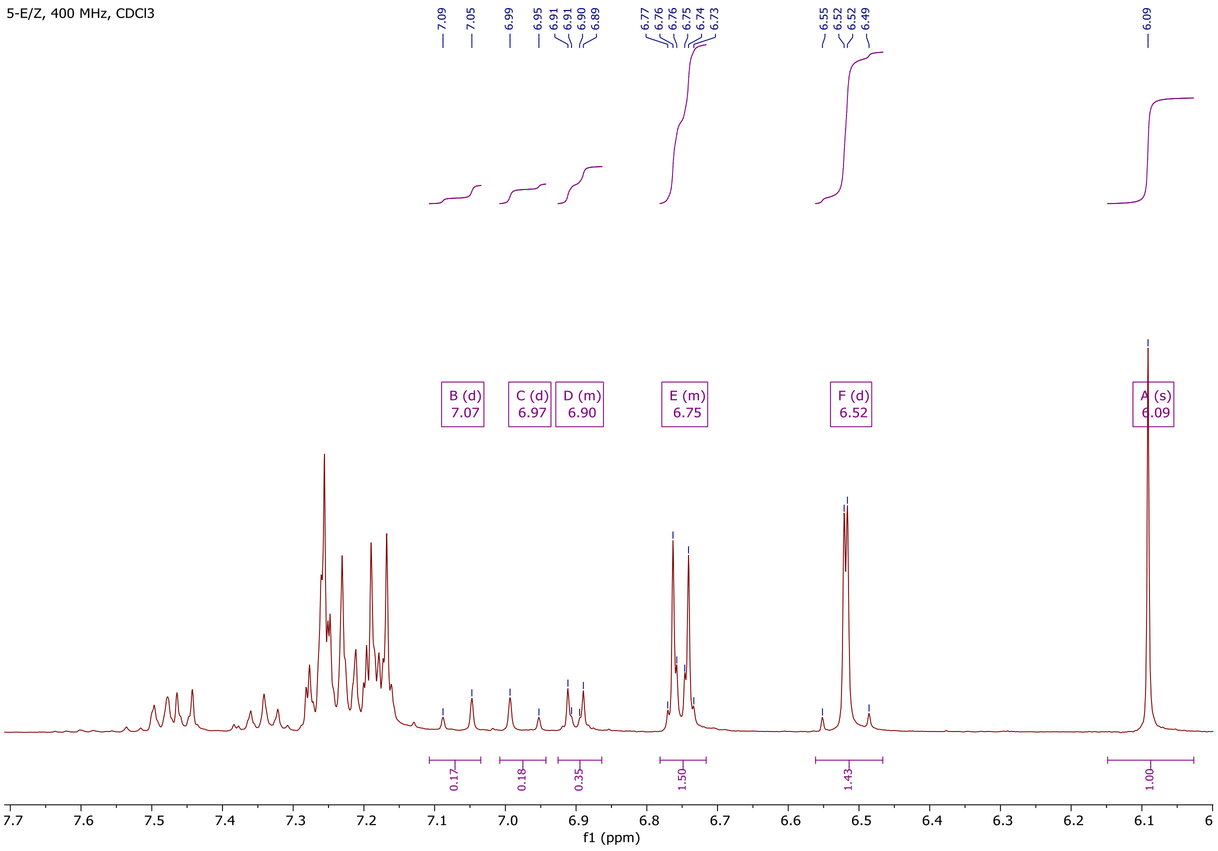

Crude reaction mixture of **6-*E***

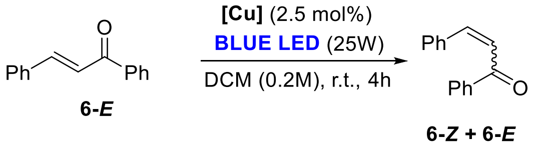

Literature NMR data for **6-E** (*Organometallics* **2018**, 37, 10, 1615–1626).

^{1}H NMR (400 MHz, $CDCl_3$): δ 7.40–7.44 (m, 3 H), 7.48–7.53 (m, 2 H), 7.53 (d, J = 15.8 Hz, 1 H), 7.56–7.59 (m, 1 H), 7.60–7.66 (m, 2 H), **7.81 (d, J = 15.8 Hz)**, **8.01–8.04 (m, 2 H, CH)**.

Literature NMR data for **6-Z** (*Chem. Eur. J.* **2015**, 21, 5350 - 5354)

^{1}H NMR (600 MHz; $CDCl_3$): **7.97-7.96 (m, 2H)**, 7.53-7.50 (m, 2H), 7.42-7.39 (m, 4H), 7.23 (dt, J = 5.2, 2.7 Hz, 2H), 7.01 (d, J = 12.8 Hz, 1H), **6.62 (d, J = 12.8 Hz, 1H)**.

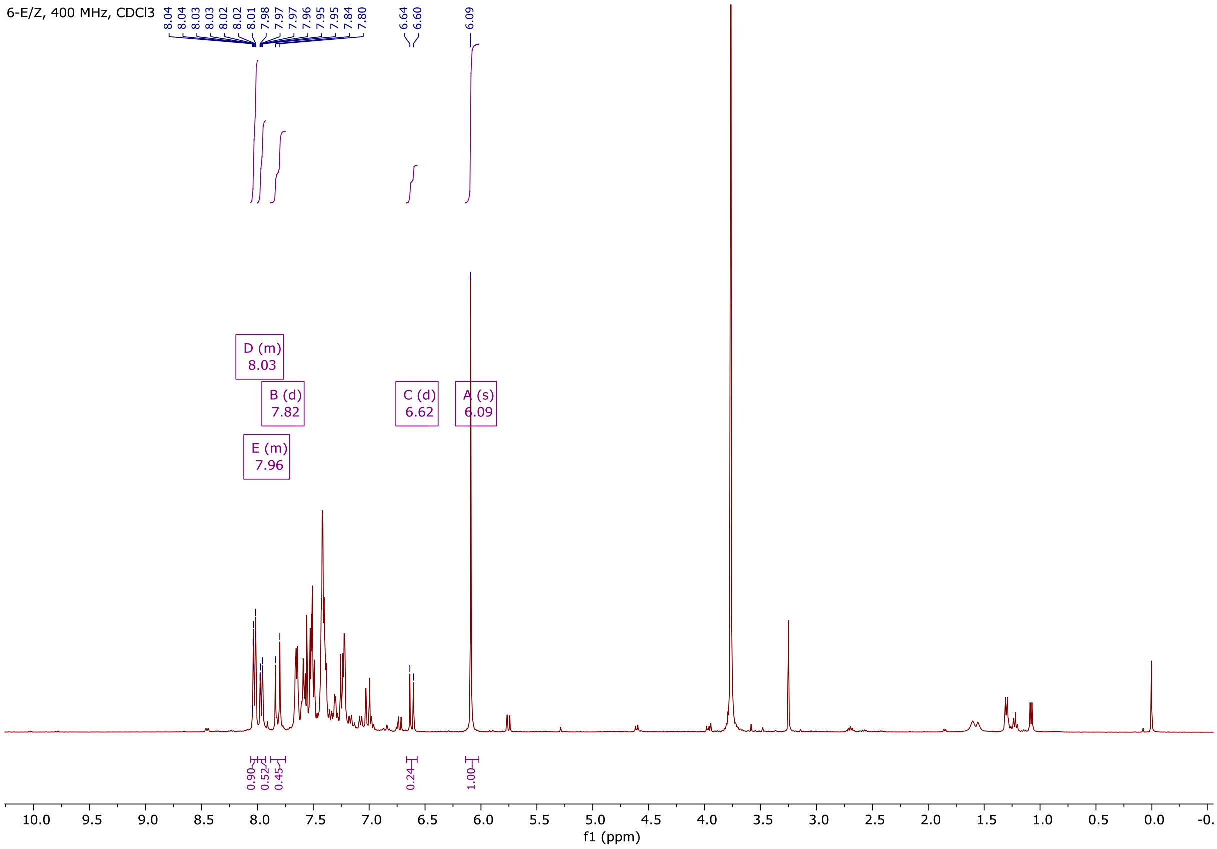

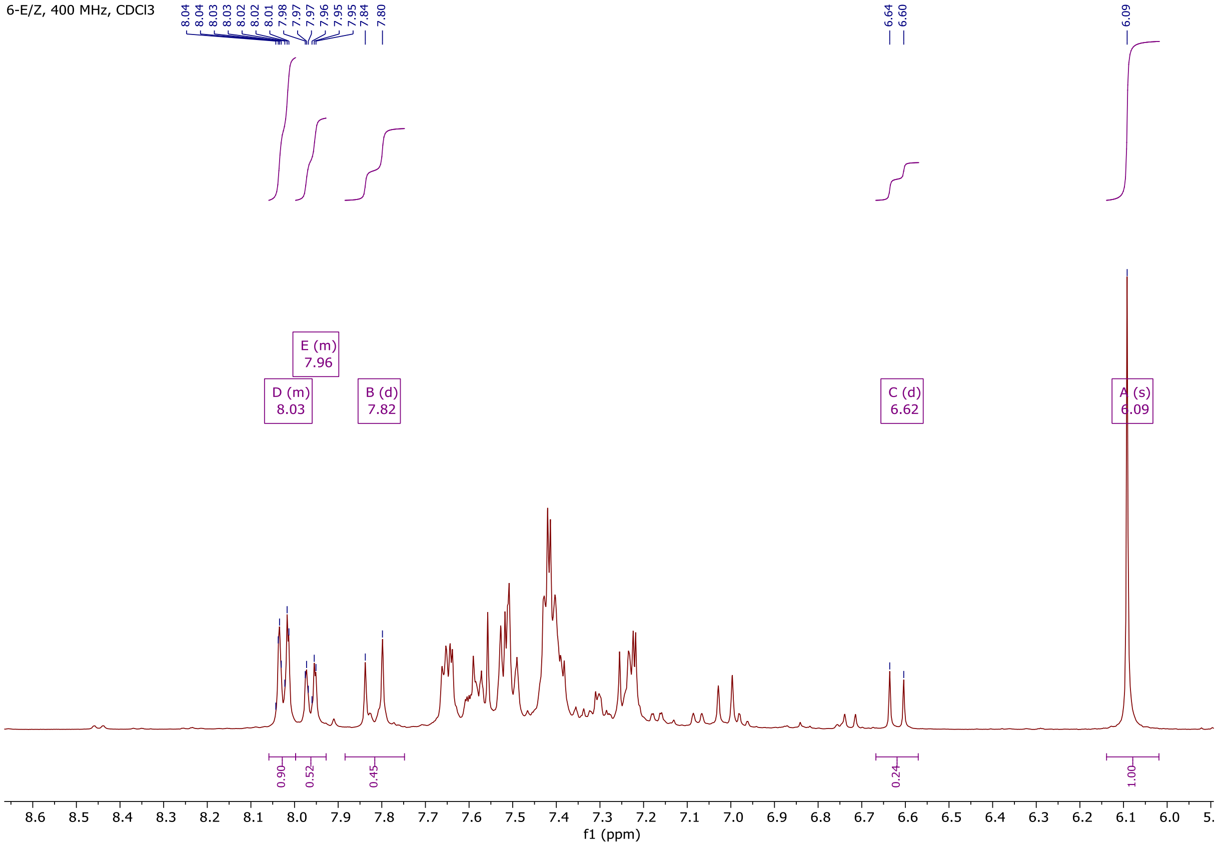

Crude reaction mixture of **7-*E***

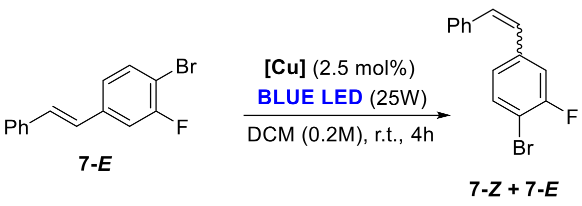

NMR data for **7-E** (this work):

^{1}H NMR (500 MHz, $CDCl_3$) δ = **7.55 – 7.47 (m, 3H)**, 7.38 (dd, *J* = 8.4, 6.9 Hz, 2H), 7.31 (t, *J* = 7.3 Hz, 1H), 7.27 (dd, *J* = 10.0, 1.9 Hz, 1H), 7.15 (dd, *J* = 8.3, 2.0 Hz, 1H), 7.11 (d, *J* = 16.3 Hz, 1H), 7.00 (d, *J* = 16.2 Hz, 1H)

NMR data for **7-Z** (this work):

^{1}H NMR (500 MHz, $CDCl_3$) δ 7.38 (t, *J* = 7.8 Hz, 1H), 7.31 – 7.18 (m, 5H), **7.00 (dd, *J* = 9.8, 1.9 Hz, 1H)**, **6.90 (dd, *J* = 8.4, 1.9 Hz, 1H)**, **6.69 (d, *J* = 12.2 Hz, 1H)**, **6.48 (d, *J* = 12.2 Hz, 1H)**.

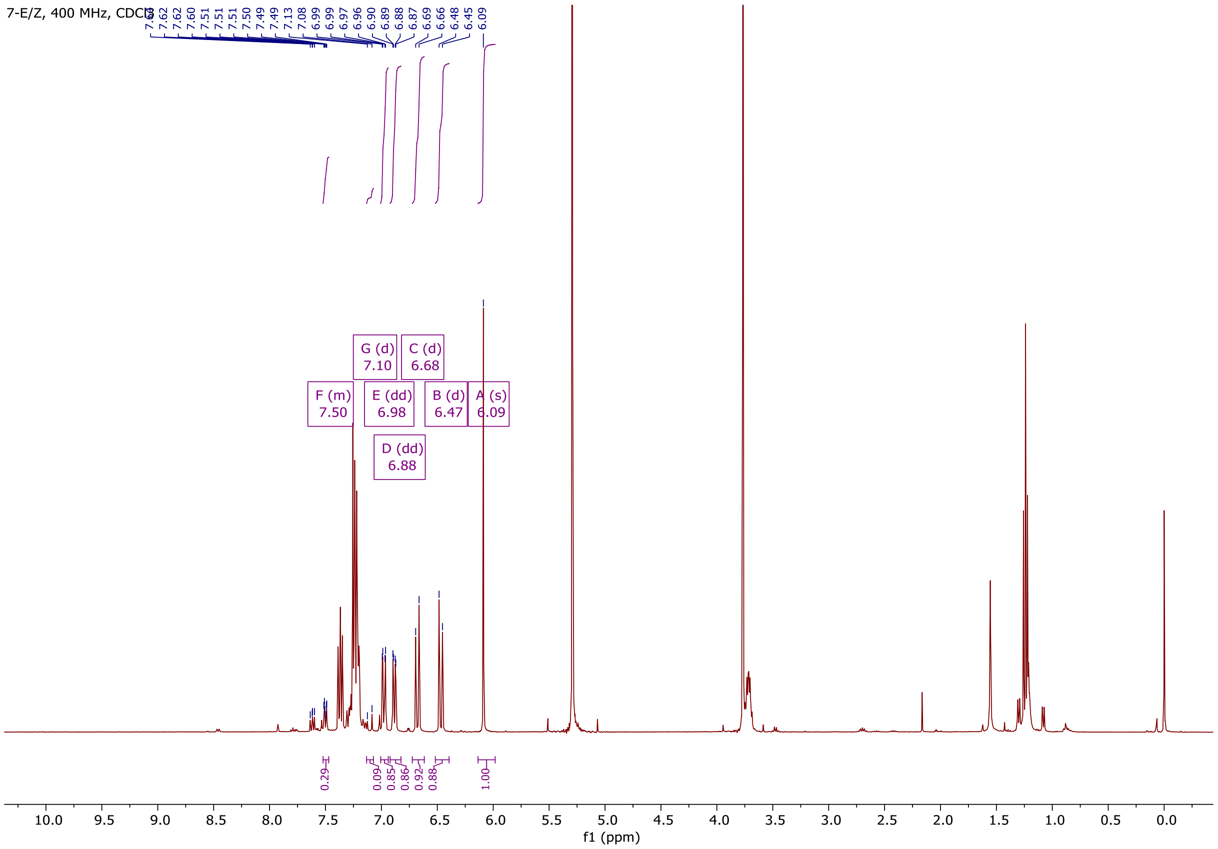

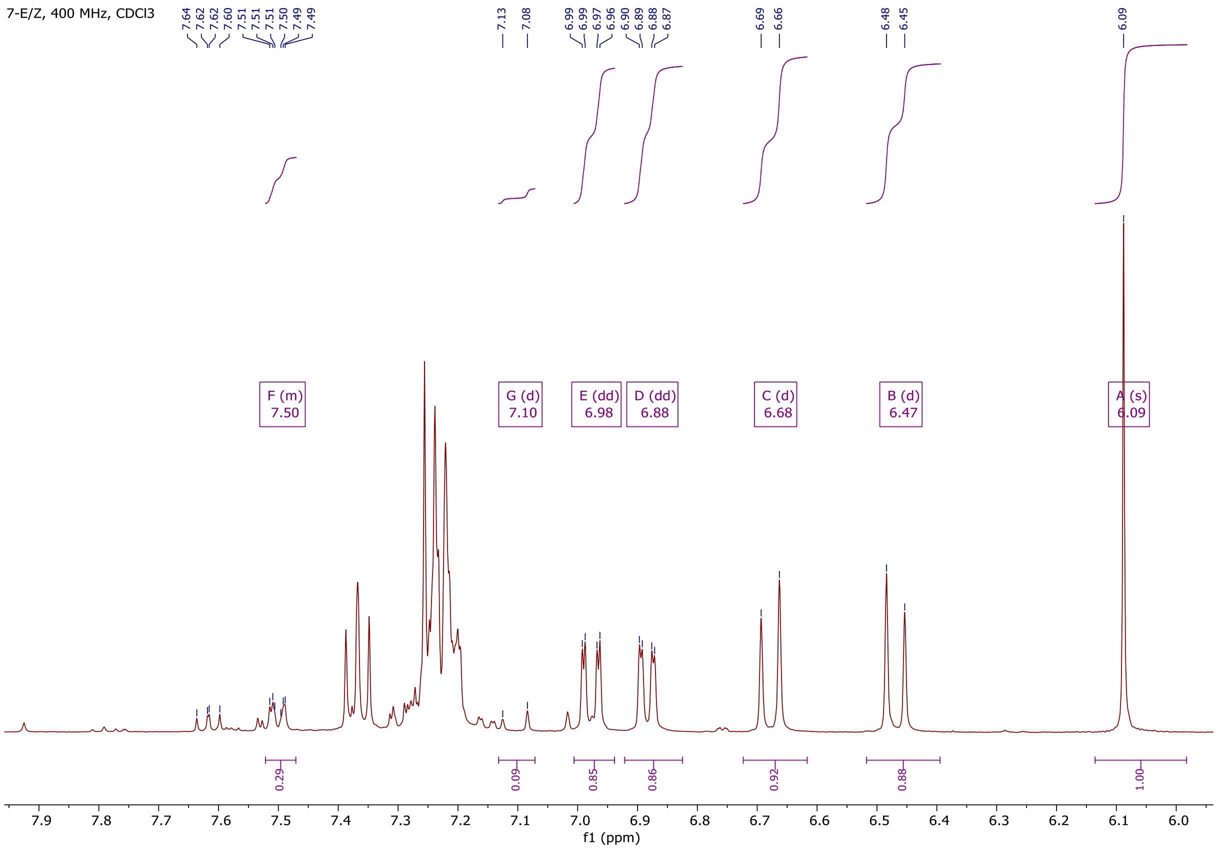

Crude reaction mixture of **8-*E***

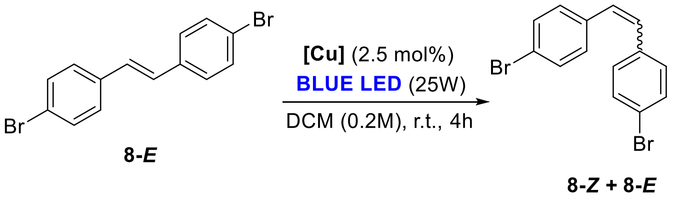

Literature NMR data for **8-E** (*Org. Lett.* 2**018**, 20, 17, 5086–5089)

^{1}H NMR (400 MHz, $CDCl_3$): δ = **7.49 – 7.45 (m, 4H)**, 7.38 – 7.33 (m, 4H), **7.01 (s, 2H)**.

Literature NMR data for **8-Z** (*J. Am. Chem. Soc.* **2019**, 141, 15, 6152–6156)

^{1}H NMR (400 MHz, CDCl3): δ = **7.37 (d, J = 8 Hz, 4H)**, 7.10 (d, J = 7.9 Hz, 4H), **6.55 (s, 2H)**

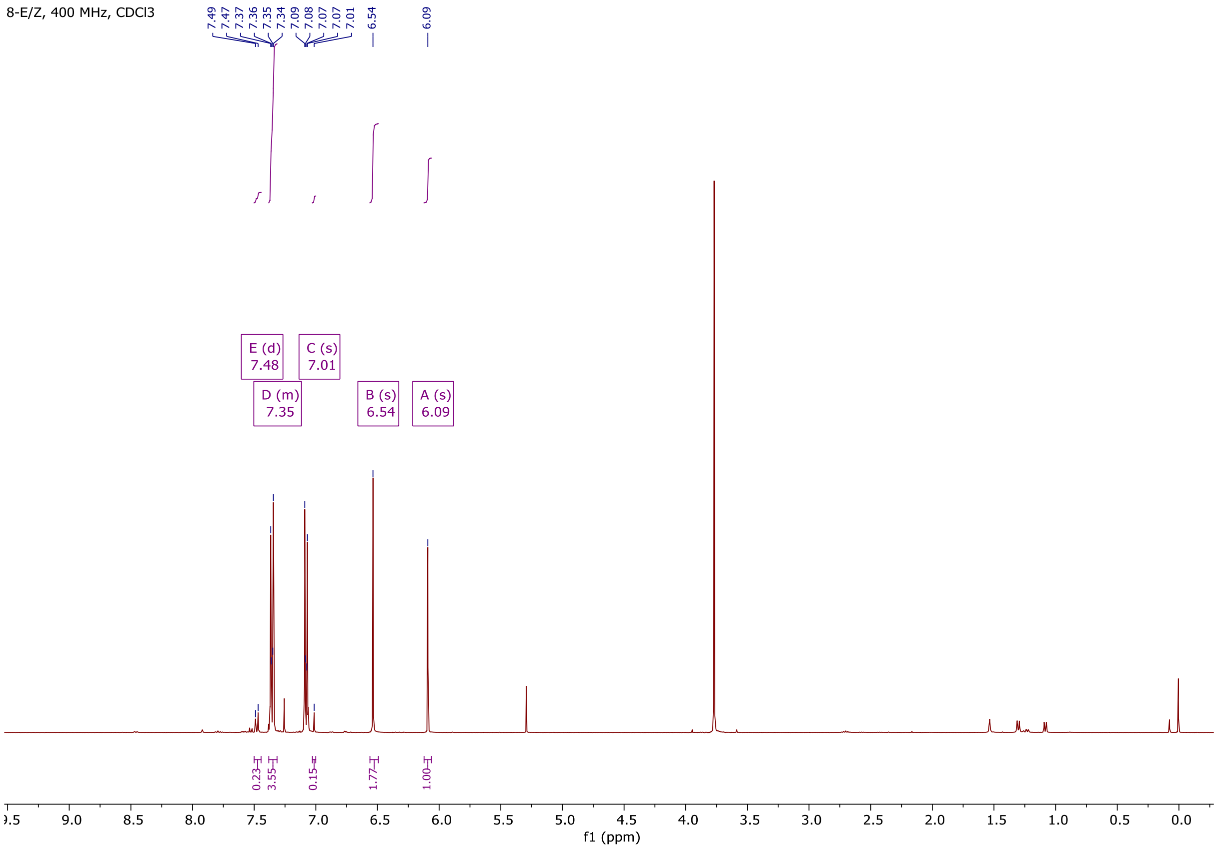

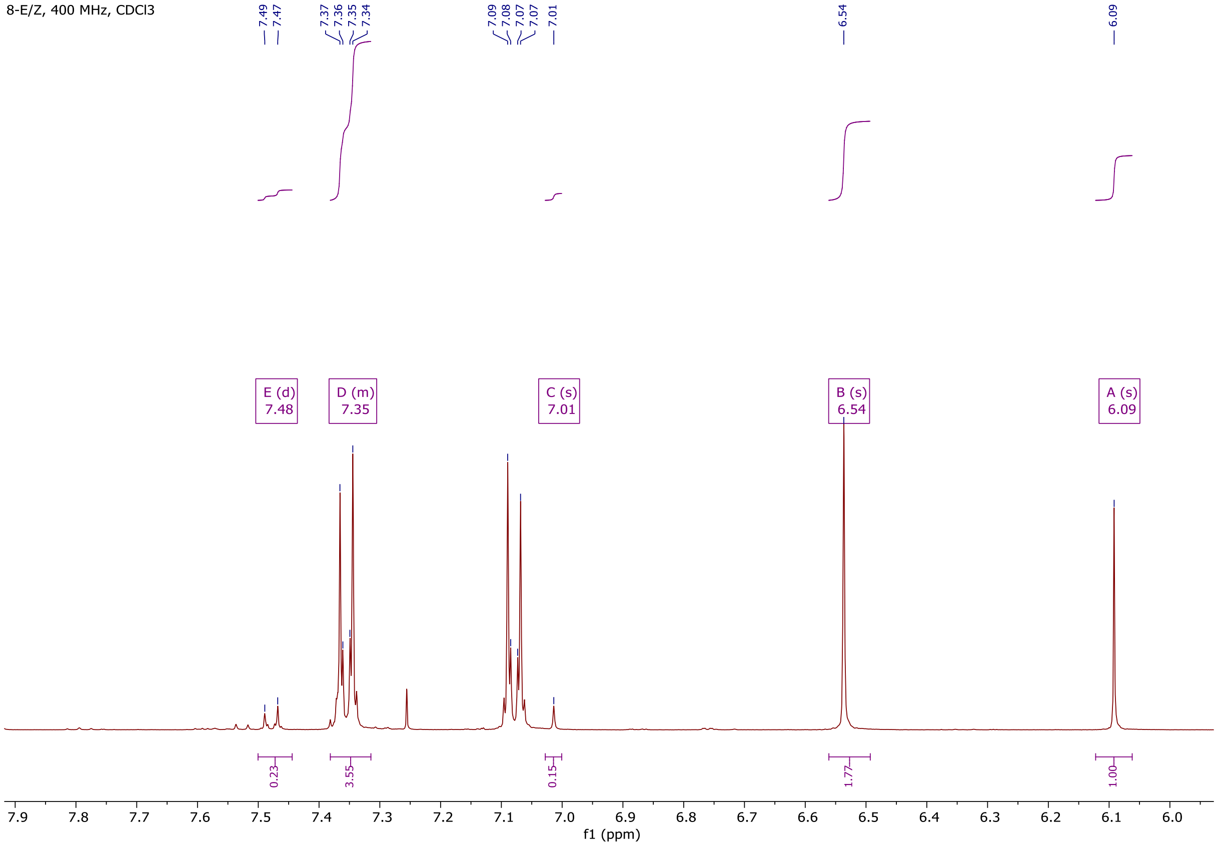

Crude reaction mixture of **9-*E***

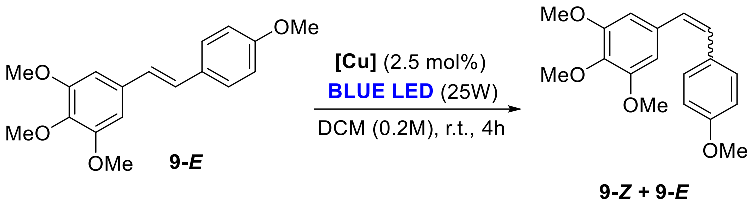

Literature NMR data for **9-E** and **9-Z** (S. Bognar, M. van Gemmeren, *Chem. Eur. J.* 2023, 29, e202203512)

^{1}H NMR (500 MHz, $CDCl_3$): δ = **7.41-7.48 (mc, 2 Htrans)**, 7.22-7.26 (mc, 2 Hcis), 6.97 (d, J = 16.2 Hz,1 Htrans), 6.87-6.93 (m, 3 Htrans), **6.77-6.81 (mc, 2 Hcis),** 6.72 (s, 2 Htrans), 6.51 (d, J = 12.1 Hz, 1 Hcis), overlaps with 6.51 (s, 2 Hcis), 6.42 (d, J = 12.2 Hz, 1 Hcis), 3.91 (s, 6 Htrans), 3.87 (s, 3 Htrans), 3.85 (s,3 Hcis), 3.83 (s, 3 Htrans), 3.78 (s, 3 Hcis), 3.69 (s, 6 Hcis) ppm.

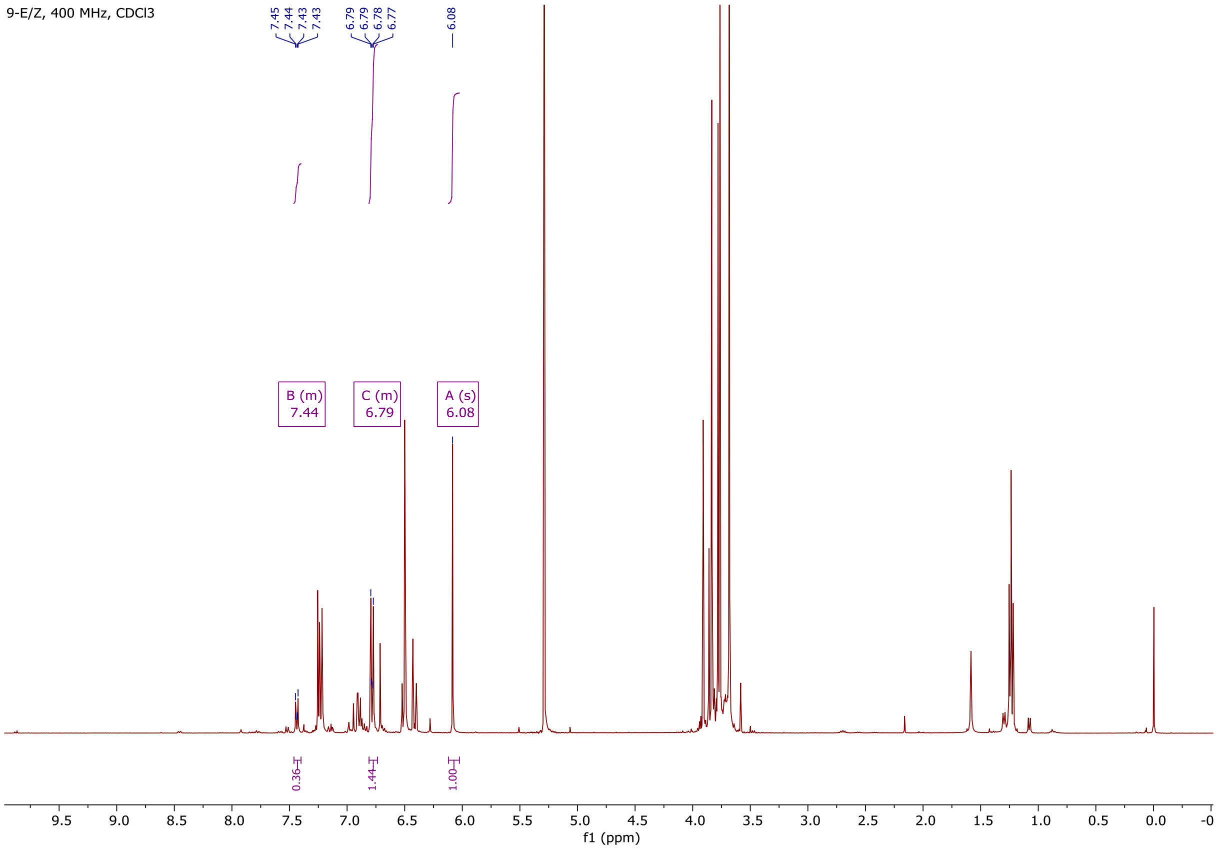

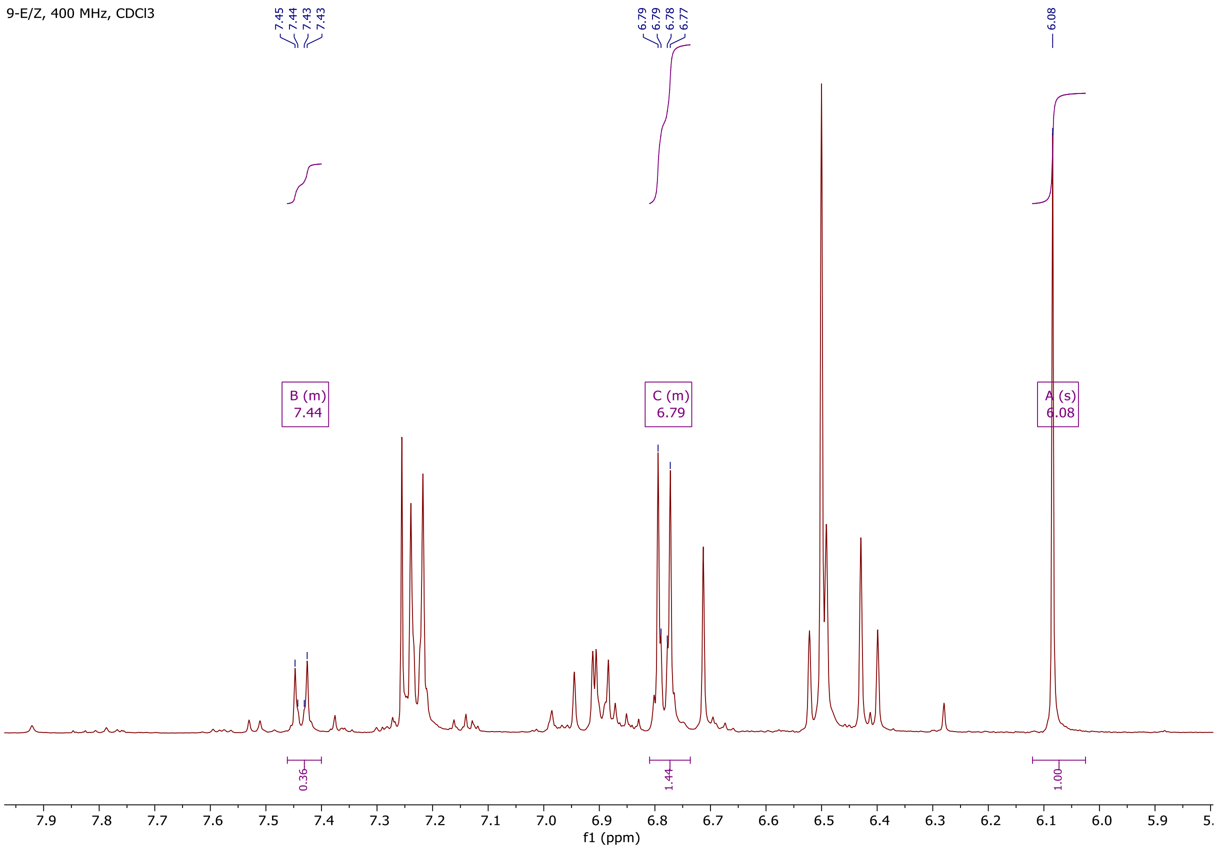

Crude reaction mixture of **10-*E***

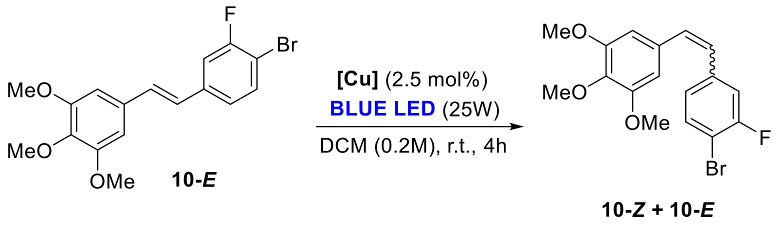

NMR data for **10-E** (this work):

^{1}H NMR (600 MHz, $CDCl_3$) δ = 7.51 (dd, *J* = 8.3, 7.2 Hz, 1H), 7.26 (dd, *J* = 9.9, 2.0 Hz, 1H), **7.14 (dd, *J* = 8.3, 2.0 Hz, 1H)**, 7.03 (d, *J* = 16.2 Hz, 1H), 6.90 (d, *J* = 16.2 Hz, 1H), 6.73 (s, 2H), 3.92 (s, 6H), 3.88 (s, 3H) ppm.

NMR data for **10-Z** (this work):

^{1}H NMR (500 MHz, $CDCl_3$) δ 7.41 (dd, *J* = 8.3, 7.2 Hz, 1H), **7.06 (dd, *J* = 9.8, 2.0 Hz, 1H)**, 6.95 (dd, *J* = 8.3, 1.9 Hz, 1H), 6.58 (d, *J* = 12.1 Hz, 1H), 6.44 (s, 2H), 6.44 (d, *J* = 12.2 Hz, 1H), 3.85 (s, 3H), 3.70 (s, 6H).

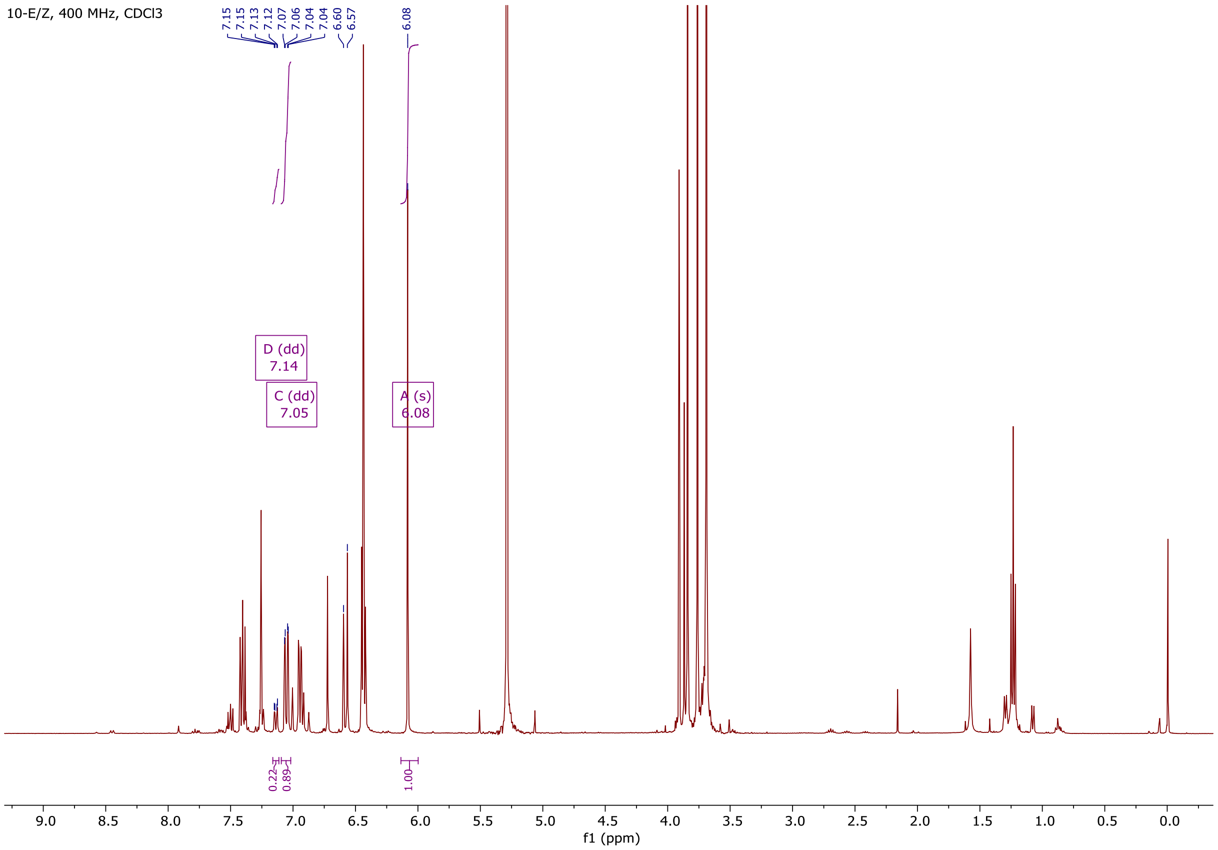

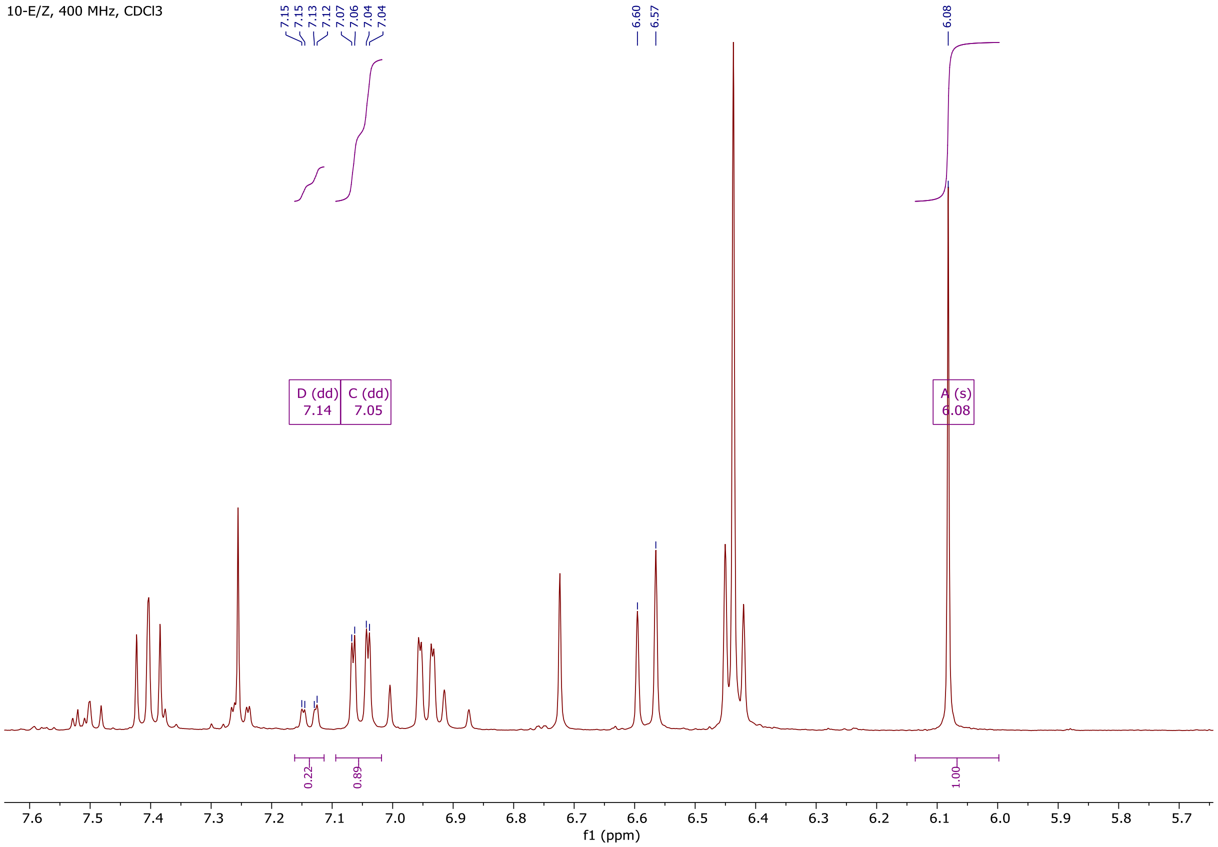

Crude reaction mixture of **12-*E***

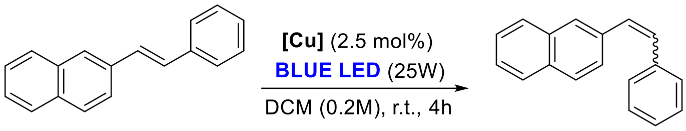

12-*E* → **12-*Z* + 12-*E***

NMR data for **12-E** (*Org. Lett.* 2016, 18, 1, 1–3)

^{1}H NMR (400 MHz, $CDCl_3$) δ 7.90 – 7.77 (m, 4H), 7.74 (d, J = 8.5 Hz, 1H), **7.56 (d, J = 7.5 Hz,2H)**, 7.51 – 7.41 (m, 2H), 7.38 (t, J = 7.4 Hz, 2H), 7.33 – 7.17 (m, 3H)

NMR data for **12-Z** (*J. Org. Chem.* 2019, 84, 6, 3579–3589)

^{1}H NMR (500 MHz, CDCl3) δ 7.84–7.75 (m, 3H), 7.70 (d, J = 8.5 Hz, 1H), 7.50–7.47 (m, 2H), 7.41 (dd, J1 = 8.5 Hz, J2 = 1.5 Hz, 1H), 7.36–7.34 (m, 2H), 7.30–7.24 (m, 3H)**, 6.82 (d, J = 12.0 Hz, 1H), 6.74 (d, J = 12.0 Hz, 1H)**.

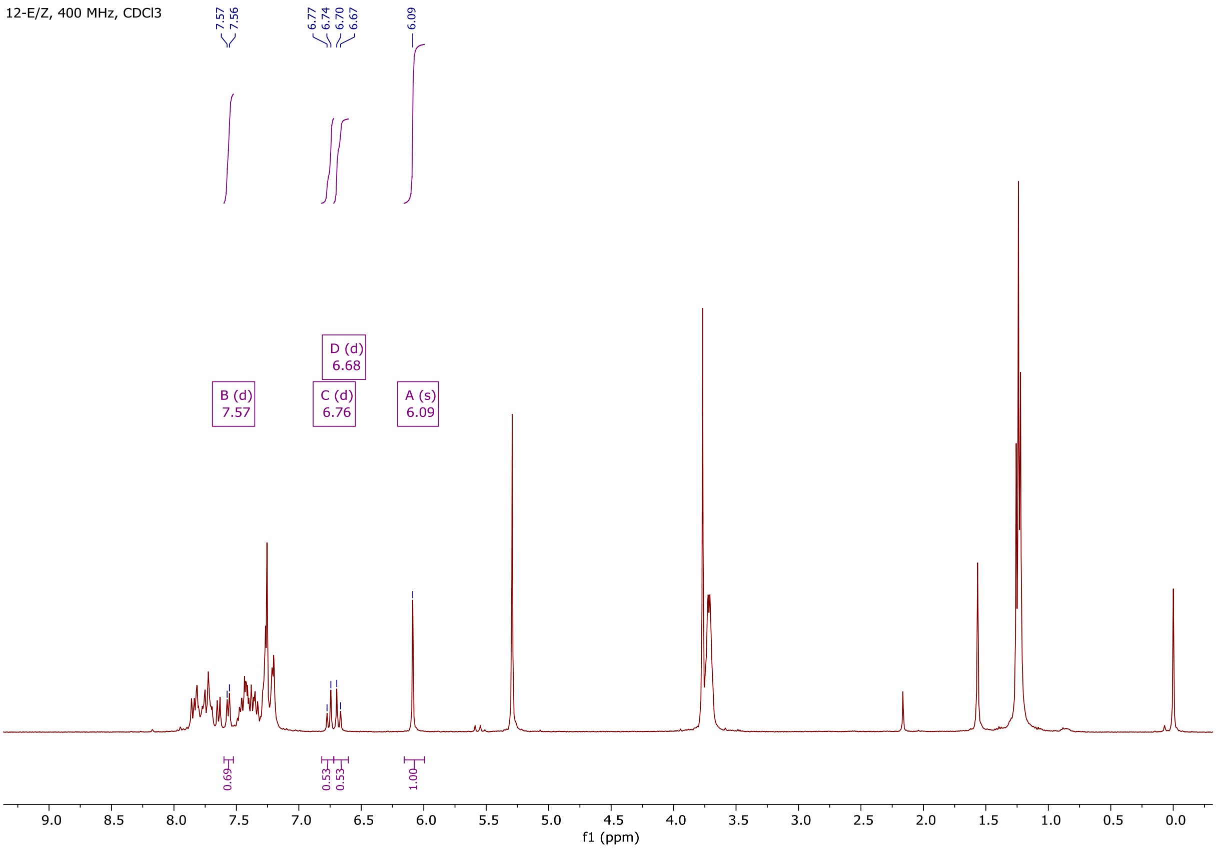

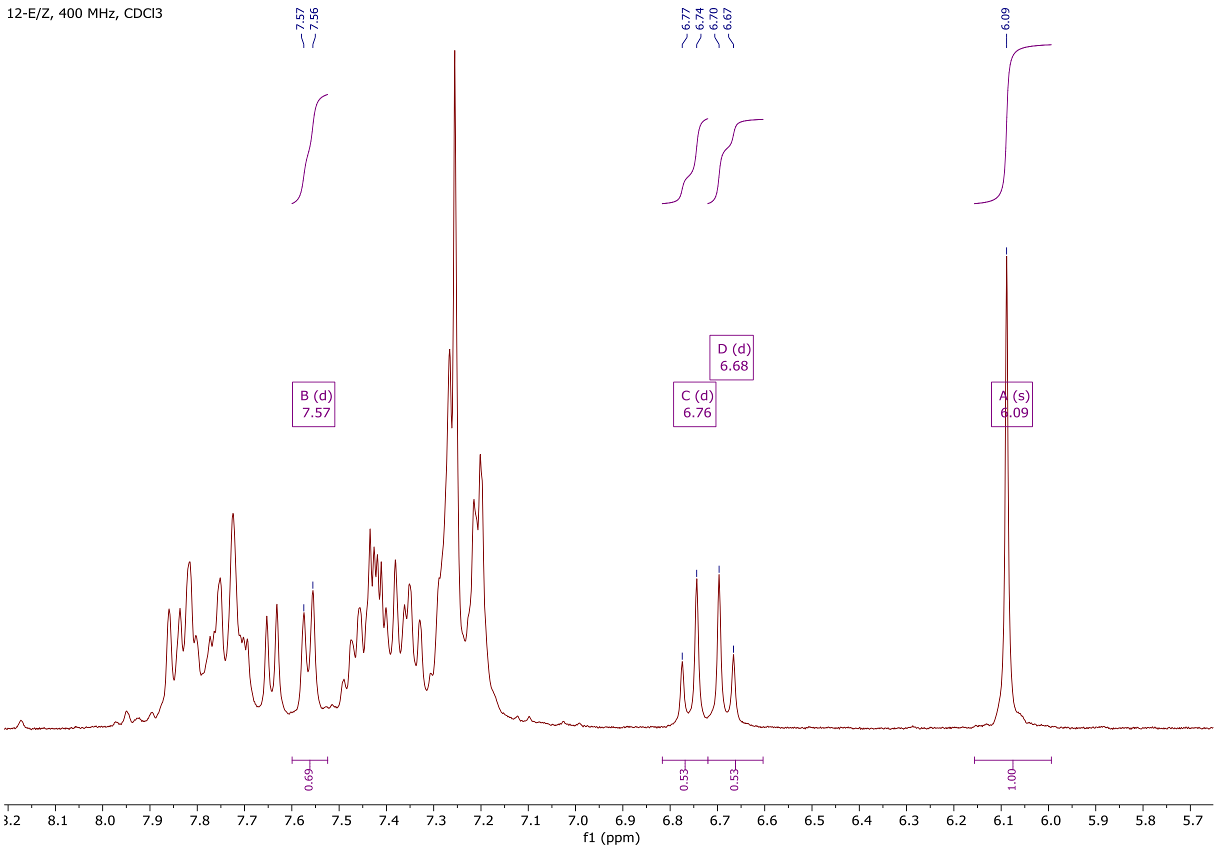

Crude reaction mixture of **13-*E***

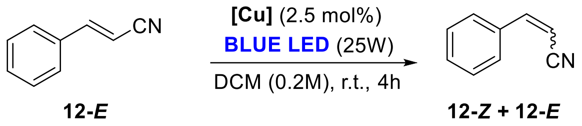

NMR data for **13-E** (*Org. Lett.* 2023, 25, 6, 917–922)

^{1}H NMR (400 MHz, CDCl3): δ 7.45 – 7.37 (m, 6H), **5.87 (d, J = 16.7 Hz, 1H)** ppm

NMR data for **13-Z** (*Org. Lett.* 2019, 21, 5, 1412–1416)

^{1}H NMR (400 MHz, CDCl3) δ 7.81 (dd, J = 6.6, 2.9 Hz, 2H), 7.47 – 7.40 (m, 3H), 7.13 (d, J = 12.1 Hz, 1H), **5.45 (d, J = 12.1 Hz, 1H)**

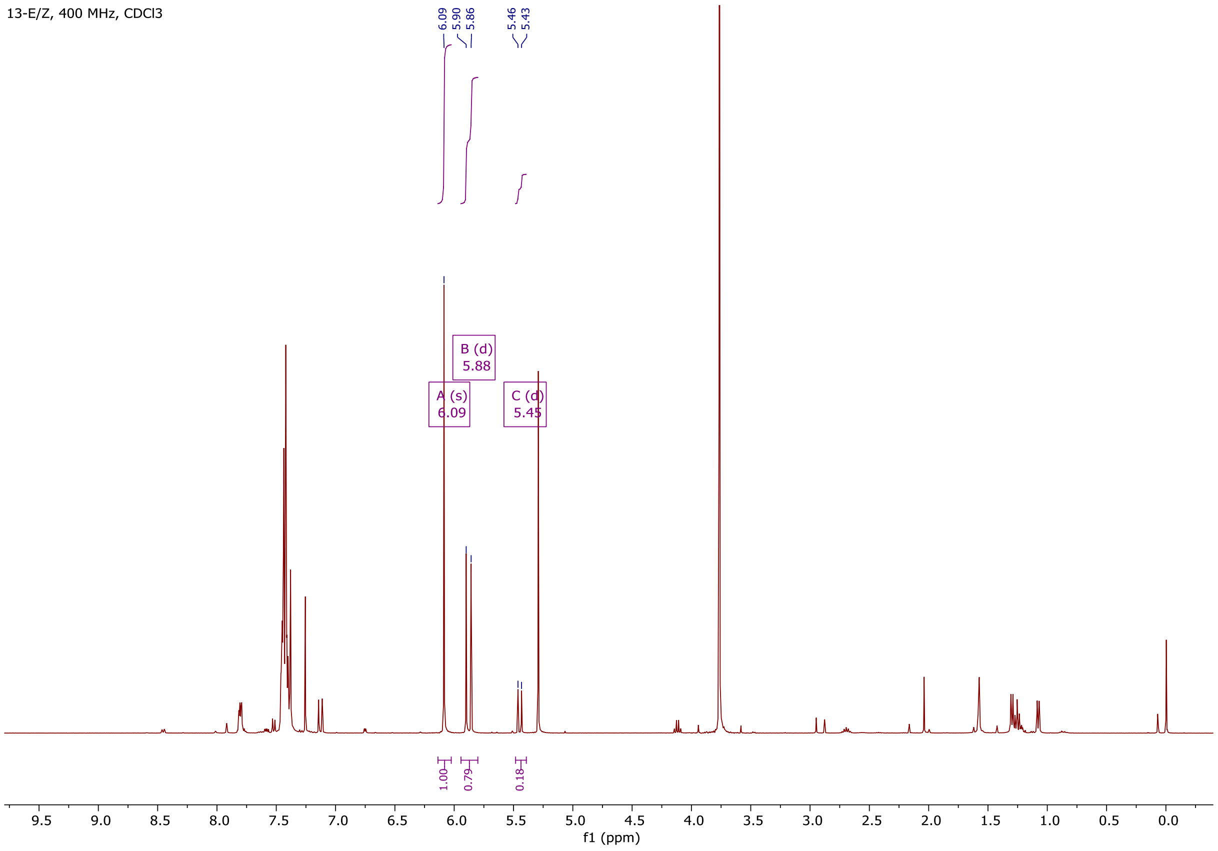

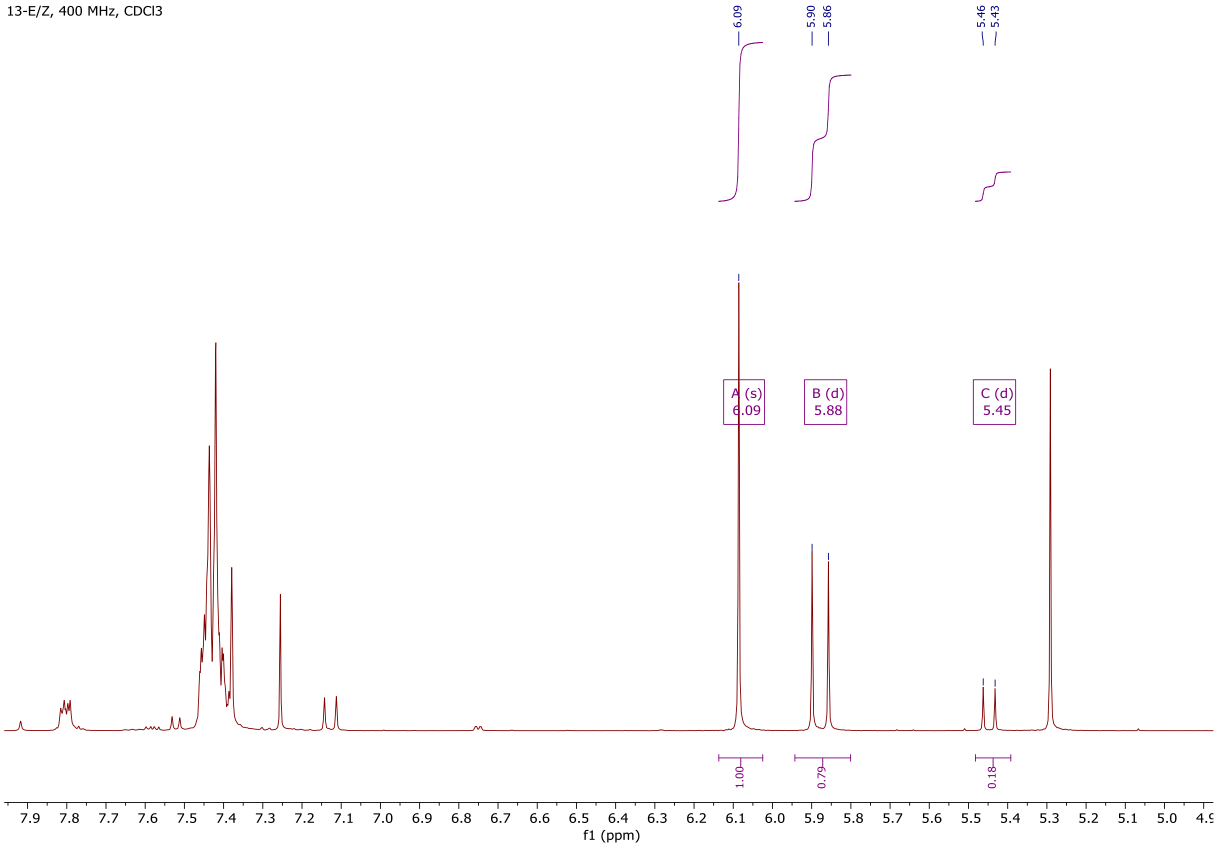

Crude reaction mixture of **14-*E***

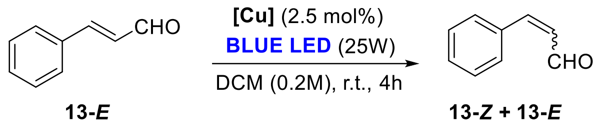

NMR data for **13-E** (*Org. Lett.* 2003, 5, 5, 777–780)

^{1}H NMR ($CDCl_3$) δ **9.73 (d, J = 7.7 Hz, 1H)**, 7.63-7.44 (m, 6H), **6.75 (dd, J 1 = 15.9 Hz, J 2 = 7.7 Hz, 1H)**

NMR data for **13-Z** (*Chem. Commun.* 2014, 50, 4119-4122)

^{1}H NMR (400 MHz, $CDCl_3$): δ **9.97 (d, J = 8.1 Hz, 1H)**, 7.63 (d, J = 11.6 Hz, 1H), 7.43 (m, 5H), **6.20 (dd, J = 11.6, 8.1 Hz, 1H)**.

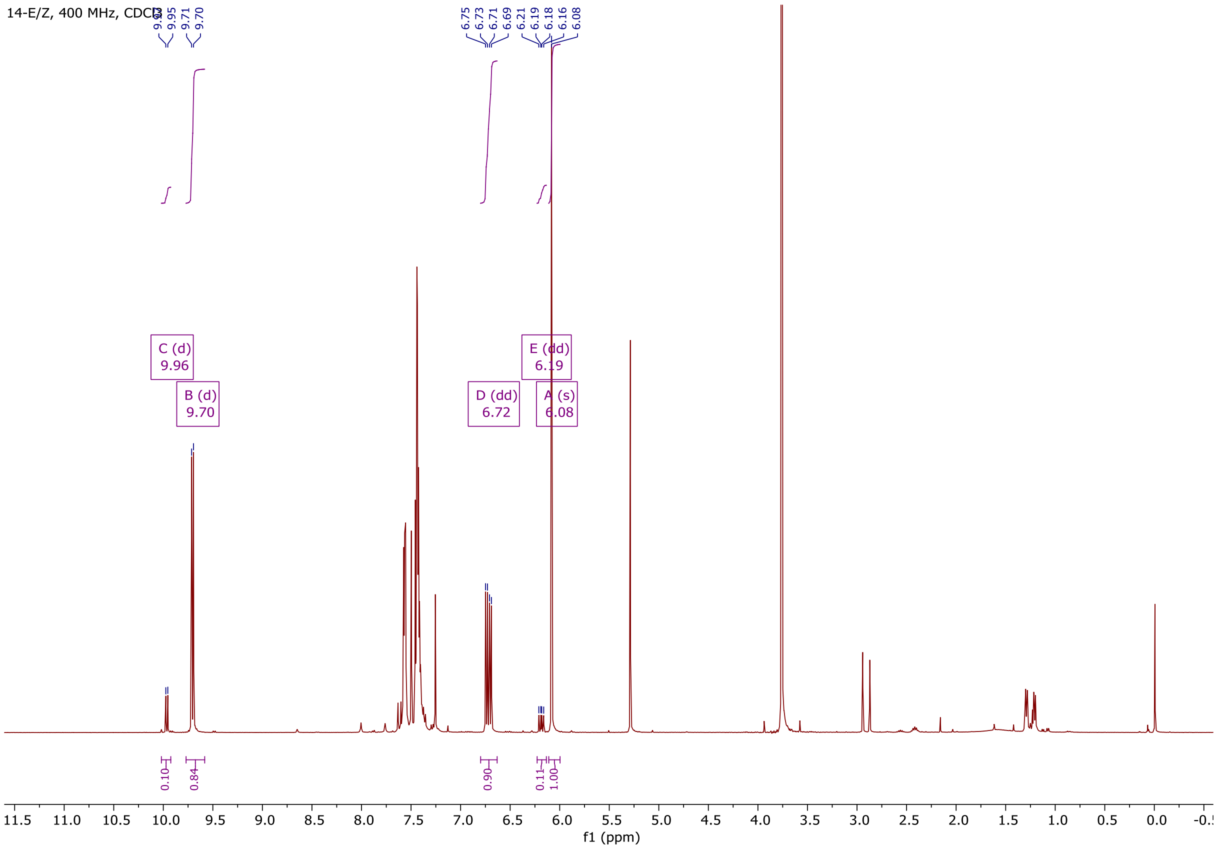

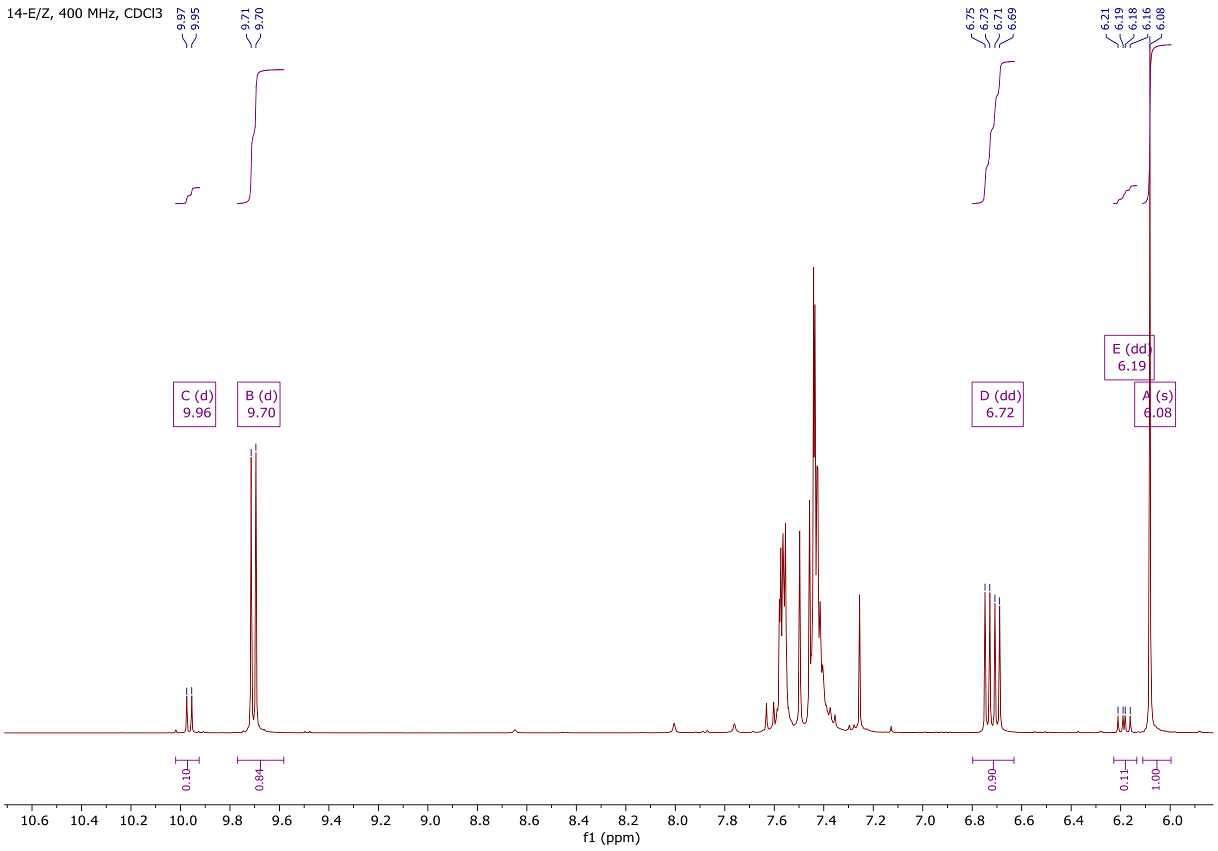

Crude reaction mixture of **18-*E***

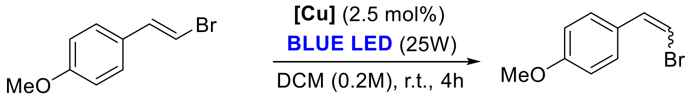

NMR data for **18-E** (*Chem. Eur. J.* **2013**, 19, 45, 15226–15239)

1H NMR (400 MHz, $CDCl_3$): δ = 7.24 (d, J = 8.7 Hz, 2H), 7.04 (d, J = 14.0Hz, 1H), 6.86 (d, J = 8.7 Hz, 2H), **6.61 (d, J = 13.9 Hz, 1H)**, 3.81 (s, 3H).

NMR data for **18-Z** (*J. Org. Chem.* 2006, 71, 5, 2009–2013)

1H NMR (500 MHz, CDCl3): δ **7.71 (d, J = 8.7 Hz, 2H)**, 7.03 (d, J = 8.1 Hz, 1H), 6.94 (d, J = 8.7 Hz, 2H), **6.34 (d, J = 8.1 Hz, 1H)**, 3.86 (s, 3H).

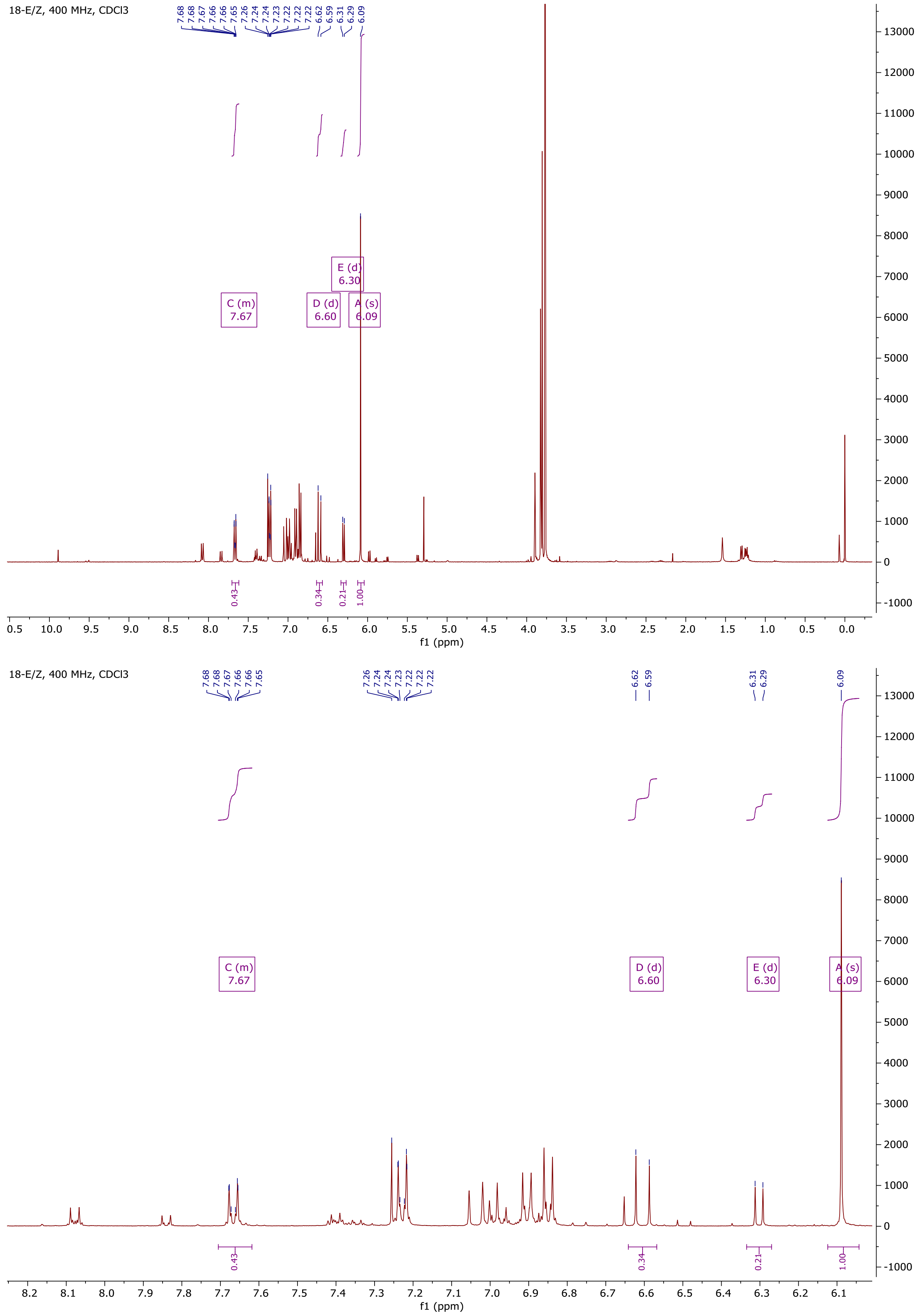

Crude reaction mixture of **19-*E***

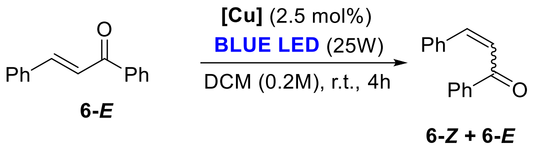

NMR data for **19-E** (*Org. Lett.* **2010**, 12, 21, 4996–4999)

^{1}H NMR (400 MHz, $CDCl_3$) δ 2.39 (s, 3H), **6.73 (d, 1H, J = 16.4 Hz)**, 7.40-7.42 (m, 3H), 7.51-7.57 (m, 3H)

NMR data for **19-Z** (*Org. Lett.* **2023**, 25, 10, 1611–1615)

^{1}H NMR (400 MHz, $CDCl_3$): 7.49–7.47 (m, 2H), 7.38–7.34 (m, 3H), **6.90 (d, J = 12.7 Hz, 1H),**

6.18 (d, J = 12.7 Hz, 1H), 2.15 (s, 3H), ppm.

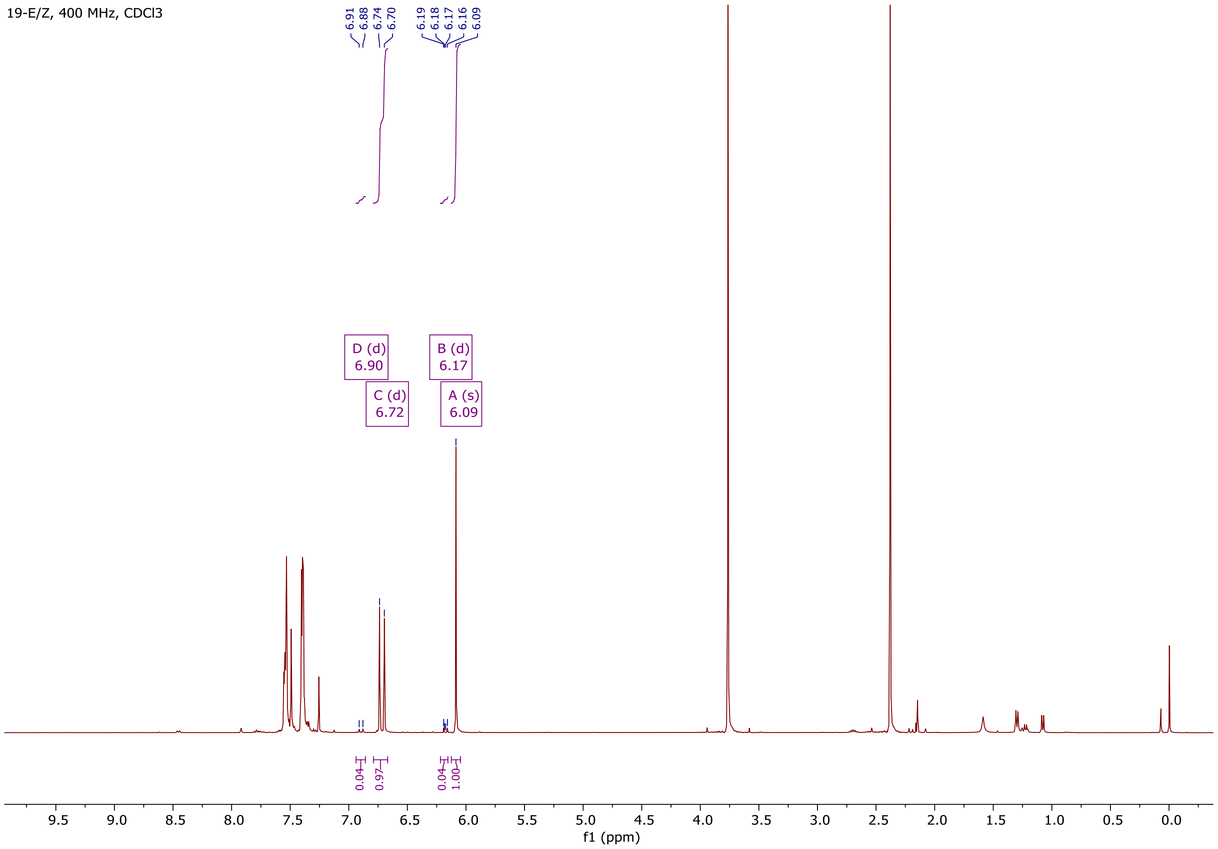

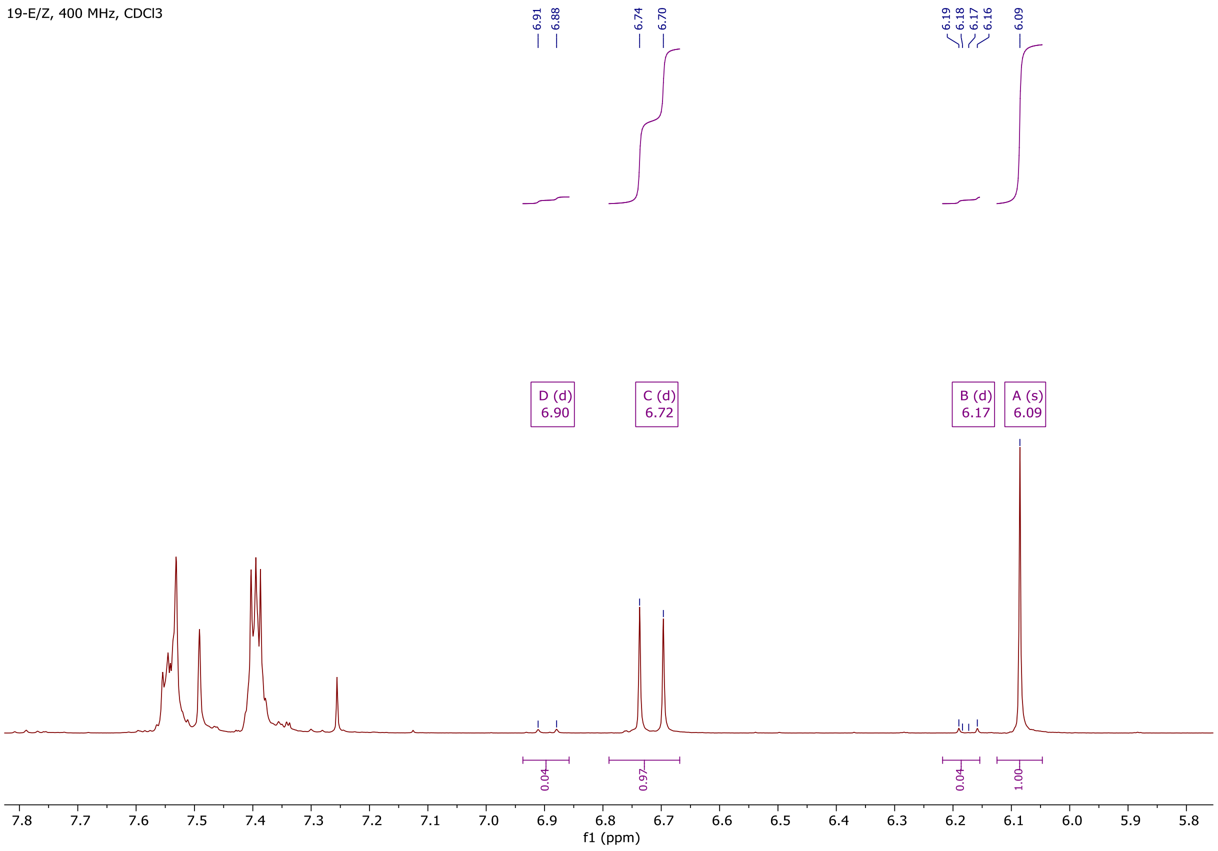

4.1.2. Analytical data for the new *Z*-compounds (7-Z and 10-Z)

Samples were obtained by column chromatography of the crude reaction mixture concentrated in vacuo (100% hexane for the mixture of **7-*E*** and **7-*Z***, 80:20 hexane/Et_2O for the mixture of **10-*E*** and **10-*Z***)

(Z)-1-bromo-2-fluoro-4-styrylbenzene, **7-*Z***

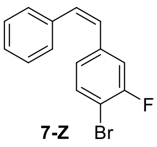

7-Z

Colorless oil

^{1}H NMR (500 MHz, $CDCl_3$) δ 7.38 (t, *J* = 7.8 Hz, 1H), 7.31 – 7.18 (m, 5H), 7.00 (dd, *J* = 9.8, 1.9 Hz, 1H), 6.90 (dd, *J* = 8.4, 1.9 Hz, 1H), 6.69 (d, *J* = 12.2 Hz, 1H), 6.48 (d, *J* = 12.2 Hz, 1H).

^{13}C{1H} NMR (126 MHz, $CDCl_3$) δ 159.8, 157.8, 138.6, 138.6, 136.4, 133.1, 132.1, 128.7, 128.4, 127.9, 127.9, 127.6, 125.9, 125.9, 116.7, 116.5, 107.4, 107.2 ppm. (signal splitting observed due to a carbon-fluorine coupling, all peaks reported without a multiplet analysis)

HRMS (APCI-TOF) m/z calcd for $[M]^+$ $C_{14}H_{10}BrF$: 275.9950; found: 275.9953

IR (film, DCM): ν 3080(m), 3058(m), 3022(s), 1886(w), 1599(s), 1567(vs), 1493(vs), 1479(vs), 1446(vs), 1417(vs), 1282(s), 1239(s), 1136(s), 1039(vs), 942(s), 919(s), 881(vs), 817(s), 772(vs), 698(vs), 609(m), 550(m), 470(m), 441(s) cm^{-1}

(Z)-5-(4-bromo-3-fluorostyryl)-1,2,3-trimethoxybenzene, **10-Z**

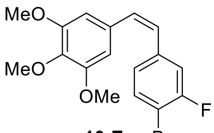

10-Z

Colorless oil

^{1}H NMR (500 MHz, $CDCl_3$) δ 7.41 (dd, *J* = 8.3, 7.2 Hz, 1H), 7.06 (dd, *J* = 9.8, 2.0 Hz, 1H), 6.95 (dd, *J* = 8.3, 1.9 Hz, 1H), 6.58 (d, *J* = 12.1 Hz, 1H), 6.44 (s, 2H), 6.44 (d, *J* = 12.2 Hz, 1H), 3.85 (s, 3H), 3.70 (s, 6H).

^{13}C{1H} NMR (126 MHz, $CDCl_3$) δ 159.8, 157.8, 153.1, 138.7, 138.7, 137.7, 133.1, 131.9, 131.6, 127.5, 127.5, 126.0, 125.9, 116.8, 116.6, 107.3, 107.2, 106.0, 60.9, 55.9 ppm.. (signal splitting observed due to a carbon-fluorine coupling, all peaks reported without a multiplet analysis)

HRMS (ESI-TOF) m/z calcd for $[M]^+$ $C_{17}H_{16}BrFO_3$: 367.0345; found: 367.0343

IR (film, DCM): ν 2937 (br), 2834(m), 1719(w), 1579(vs), 1505(vs), 1482(s), 1462(s), 1421(vs), 1329(s), 1239(vs), 1129(vs), 1038(m), 1007(s), 881(s), 852(m), 797(m), 714(m), 589(m), 454(w) cm^{-1}

4.1.3. Extended background test for the photoisomerization of 1-E and 17-E

Table S1. Extended background test for the photoisomerization of 1-E and 17-E

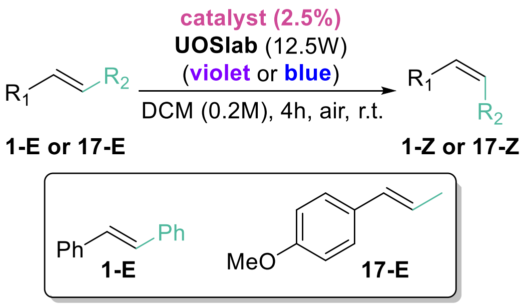

	substrate	catalyst	hν color	time [h]	Z/E [%]
1	**1-*E***	none	violet	4	0/100
2	**1-*E***	none	blue	4	0/100
3	**1-*E***	**phen**	**violet**	**4**	**76/24**
4	**1-*E***	**phen**	**blue**	**4**	**76/24**
5	**1-*E***	IPrCuCl	violet	4	2/98
6	**1-*E***	IPrCuCl	blue	4	0/98
7	**1-*E***	S7	violet	4	11/89
8	**1-*E***	S7	blue	4	11/89
9	**17-*E***	none	violet	4	0/100
10	**17-*E***	none	blue	4	0/100
11	**17-*E***	phen	violet	4	6/94
12	**17-*E***	phen	blue	4	3/97
13	**17-*E***	IPrCuCl	violet	4	2/98
14	**17-*E***	IPrCuCl	blue	4	2/98
15	**17-*E***	S7	**violet**	**4**	**90/10**
16	**17-*E***	S7	blue	4	9/91

Reaction conditions: olefin (0.2 mmol), catalyst (2.5 mol%), UOSlab photoreactor with violet (λ_{max} 405 nm) or blue (λ_{max} 450 nm) light irradiation (12.5W power), DCM (0.2M), on air

Interesting observation is that both **phen** and **S7** are able to catalyze the isomerization of **1-*E*** and/or **17-*E*** to some extent even without copper. As a plausible rationale for this observation we propose in situ formation of excimeric species in reaction mixture.

4.1.4. Catalyst reusability test – procedure

Catalyst **[Cu]-1** (7.8 mg, 0.01 mmol, 5 mol%) and and **11-E** (70 mg, 0.2 mmol) were placed in glass vial along with PTFE-coated magnetic stirring bar. Solvent (freshly distilled DCM, 2 mL) was added, vial was capped and degassed by purging with argon through a needle piercing the septum for 5 min. Flask was placed in UOSlab photoreactor with a cooling plate set at 8 °C, LED lamp (blue, λ_{max} 450 nm) set at 25W (100% power) and stirring set at 700 rpm. At 1.5 hour intervals, a sample of the reaction mixture was collected, diluted with ethyl acetate (approximately 1.5 mL) and analyzed by GC for the Z/E ratio. After first and second sample collection next portions of **11-E** (70 mg, 0.2 mmol) in DCM (1 mL) were added.

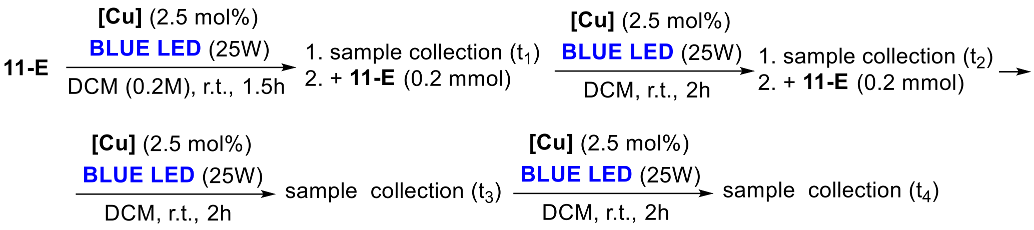

Total number of samples collected: 4. **11-Z/11-E** ratio at t_1 = 90/10, t_2 = 85/15, t_3 = 83/17, t_4 = 85/15.

4.1.5. [Cu]-1 ligand exchange study

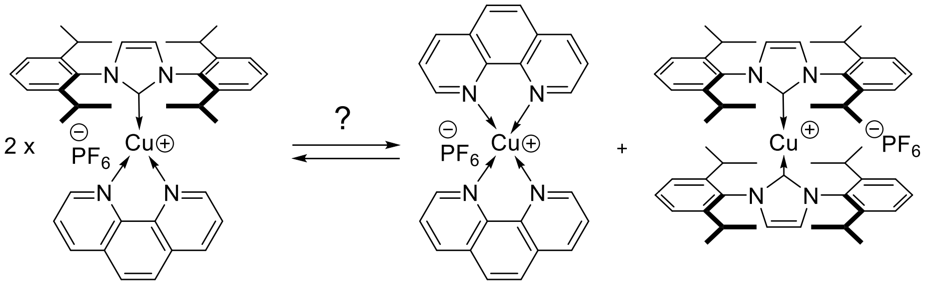

[Cu]-1 **[Cu]-4** **[Cu]-5**

To check whether the heteroleptic **[Cu]-1** complex can undergo dynamic exchange of ligands under the reaction conditions, we performed a test of the catalytic activity of the homoleptic **[Cu]-4** and **[Cu]-5** complexes. Both were found to be inactive in the isomerization of **1-*E***. Similarly, an equimolar mixture of **[Cu]-4** and **[Cu]-5** showed no reactivity with respect to **1-*E*** isomerization ruling out formation of active mixed ligand complex. It cannot be ruled out that **[Cu]-1** solution may at some level equilibrate into the mixture of **[Cu]-4** (which is a violet solid showing very poor solubility in common organic solvents), and **[Cu]-5**, however, we did not observe formation of dark precipitates in the course of our reactions and we didn't observe formation of easily soluble and ^{1}H NMR visible **[Cu]-5** at any point.

Table S2. 4.1.5.[Cu]-1 ligand exchange study

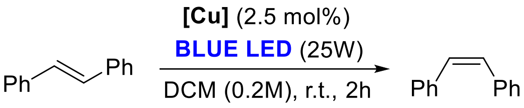

1-*E* **1-*Z***

[Cu]	1-Z / 1-E
[Cu]-1	95/5
[Cu]-4	<1/99
[Cu]-5	<1/99
[Cu]-4 +[Cu]-5 (1:1)	<1/99

Reaction conditions: olefin **1-*E*** (0.2 mmol), catalyst (2.5 mol%), UOSlab photoreactor with blue (λ_{max} 450 nm) light irradiation (25W power), DCM (0.2M), ambient air atmosphere

4.1.6. [Cu]-1 photostability study

[Cu]-1 (49 mg, 0.063 mmol) and **TMB** (3.5 mg, 0.021 mmol) were placed in 10 mL glass vial on air with PTFE-coated magnetic stirring bar. DCM (5 mL) was added and contents of the vial were stirred until fully homogenous. Sample of the mixture (1 mL) was collected, concentrated in vacuo and

analyzed (^{1}H NMR, 16 scans, 5s relaxation time) to determine the **[Cu]-1/TMB** ratio at t_0. Vial was sealed with aluminum cap containing a rubber septum, placed in UOSlab photoreactor with external cooling (8 °C), and stirred at 700 rpm with irradiation by blue (λ_{max}=450 nm) 12.5W LED. 1 mL sample of the reaction mixture was collected at t_1 = 4h and t_2 = 24 h. Samples were concentrated and analyzed in a same manner as t_0 sample. ^{1}H NMR analysis revealed >99% remaining **[Cu]-1** at t_1 and 80% remaning **[Cu]-1** at t_2. Singal broadening and reaction mixture color change (from a pale yellow to a light green with slight precipitation) indicates possible oxidative decomposition pathway with formation of poorly soluble, paramagnetic Cu^{II} species.

4.1.7. Large scale experiment

[Cu]-1 activity in isomerization of 1-E on a larger scale and concentration and reduced catalyst loading was confirmed by a single experiment following modified procedure B. 900 mg (5.0 mmol) of **1-*E*** and 38.9 mg (0.05 mmol, 1 mol%) of **[Cu]-1** were placed in 10 mL glass vial along with PTFE-coated magnetic stirring bar. Freshly distilled DCM (5 mL) was added, vial was capped then placed in UOSlab photoreactor with external cooling (8 °C), and stirred at 700 rpm with irradiation by blue (λ_{max}=450 nm) 12.5W light for 24 h. Z/E ratio was determined via GC analysis of 0.05-0.1 mL samples of the reaction mixture.

Table S3. **Large scale experiment**

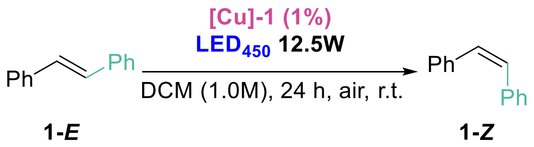

	Time [h]	Z/E
1	2	31/69
2	4	51/49
3	18	87/13
4	24	89/11

1.1.1. Mechanistic study – reaction in the presence of singlet/triplet quenchers – procedure

[Cu]-1 (2.5 mol%), **1-*E*** (0.2 mmol, 36 mg) and quencher (0.2 mmol of TEMPO, 1,4-benzoquinone, 1,3-cyclohexadiene or azulene) were placed in 10 mL glass vial along with PTFE-coated magnetic stirring bar and DCM (2 mL). Vial was sealed with aluminum cap containing a rubber septum, placed in UOSlab photoreactor with external cooling (8 °C), and stirred at 700 rpm with irradiation by blue (λ_{max}=450 nm) 12.5W LED, stirring set to 700 rpm. *Z*/*E* ratio was determined using GC analysis of 0.05-0.1 mL samples of the reaction mixture at t_1 = 1h, t_2 = 2h and t_3 = 4h

Table S4. **Mechanistic study – reaction in the presence of singlet/triplet quenchers – procedure**

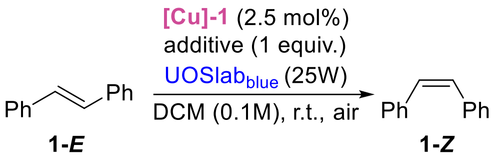

Entry	Additive	*Z*/*E* [1h]	*Z*/*E* [2h]	*Z*/*E* [4h]
1	none	71/29	88/12	95/5

2	TEMPO	28/72	44/56	61/39
3	Benzoquinone	25/75	46/56	61/39
4	1,3-cyclohexadiene	75/25	92/8	95/5
5	azulene	38/62	38/62	38/62

Quenching effect was studied further by testing the reaction profile in slightly modified procedure using milder conditions: **[Cu]-1** loading was reduced to 1.0 mol%, irradiation power was reduced to 12.5W and reaction time was shortened. In this case slight inhibition by the triplet state quenchers (O_2 and 1,3-cyclohexadiene) was observed.

Table S5. **Mechanistic study – reaction in the presence of singlet/triplet quenchers – procedure**

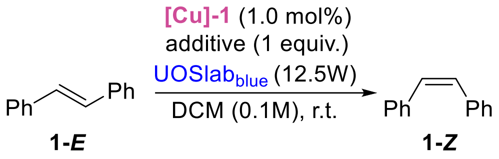

Entry	Atmosphere	Additive	*Z*/*E* [15 min]	*Z*/*E* [1h]	*Z*/*E* [2h]
1	Argon	none	11/89	46/54	69/31
2	Argon	1,3-cyclohexadiene	10/90	42/58	67/33
3	O_2	none	9/91	34/66	49/51

1.1.1. Photoisomerization of Z-olefins

Crude reaction mixture for **1-*Z***

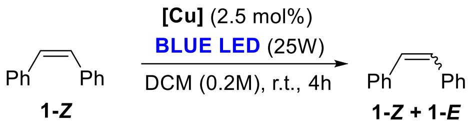

Literature NMR data for **1-E** (*Org. Lett.* **2014**, 16, 11, 3020–3023).

^{1}H NMR (400 MHz, $CDCl_3$): δ = **7.54-7.56 (m, 4H)**, **7.37-7.40 (m, 4H)**, 7.26-7.31 (m, 2H), **7.14 (s, 2H)**.

Literature NMR data for **1-Z** (*Org. Lett.* **2016**, 18, 23, 6196–6199)

^{1}H NMR (400 MHz, $CDCl_3$) δ 7.38 – 7.17 (m, 10H), **6.66 (s, 2H)**.

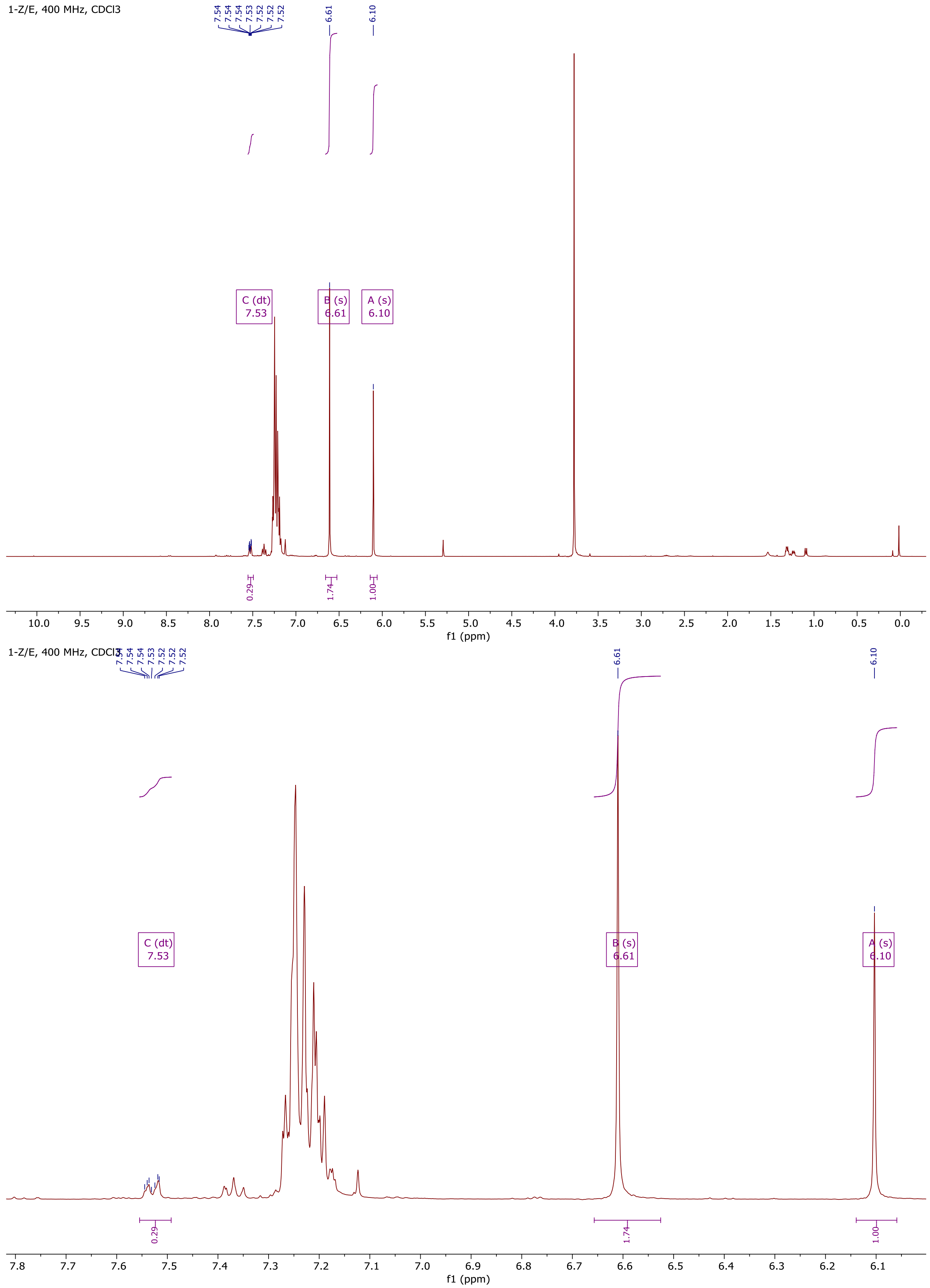

Crude reaction mixture for **9-*Z***

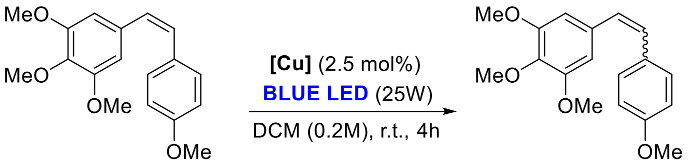

9-*Z* **9-*Z* + 9-*E***

Literature NMR data for **9-E** and **9-Z (**S. Bognar, M. van Gemmeren, *Chem. Eur. J.* 2023, 29, e202203512)

^{1}H NMR (500 MHz, CDCl3): δ **= 7.41-7.48 (mc, 2 Htrans**), 7.22-7.26 (mc, 2 Hcis), 6.97 (d, J = 16.2 Hz,1 Htrans), 6.87-6.93 (m, 3 Htrans), **6.77-6.81 (mc, 2 Hcis),** 6.72 (s, 2 Htrans), 6.51 (d, J = 12.1 Hz, 1 Hcis), overlaps with 6.51 (s, 2 Hcis), 6.42 (d, J = 12.2 Hz, 1 Hcis), 3.91 (s, 6 Htrans), 3.87 (s, 3 Htrans), 3.85 (s,3 Hcis), 3.83 (s, 3 Htrans), 3.78 (s, 3 Hcis), 3.69 (s, 6 Hcis) ppm.

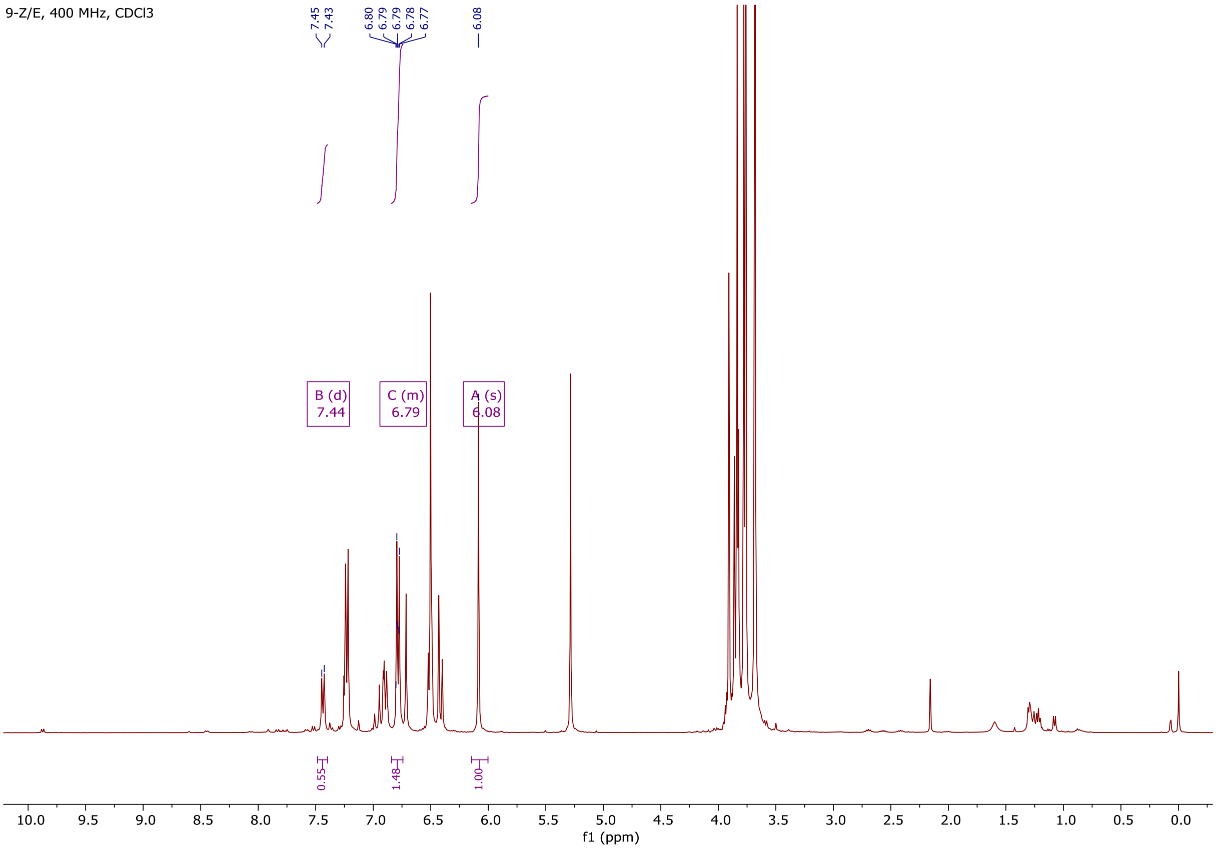

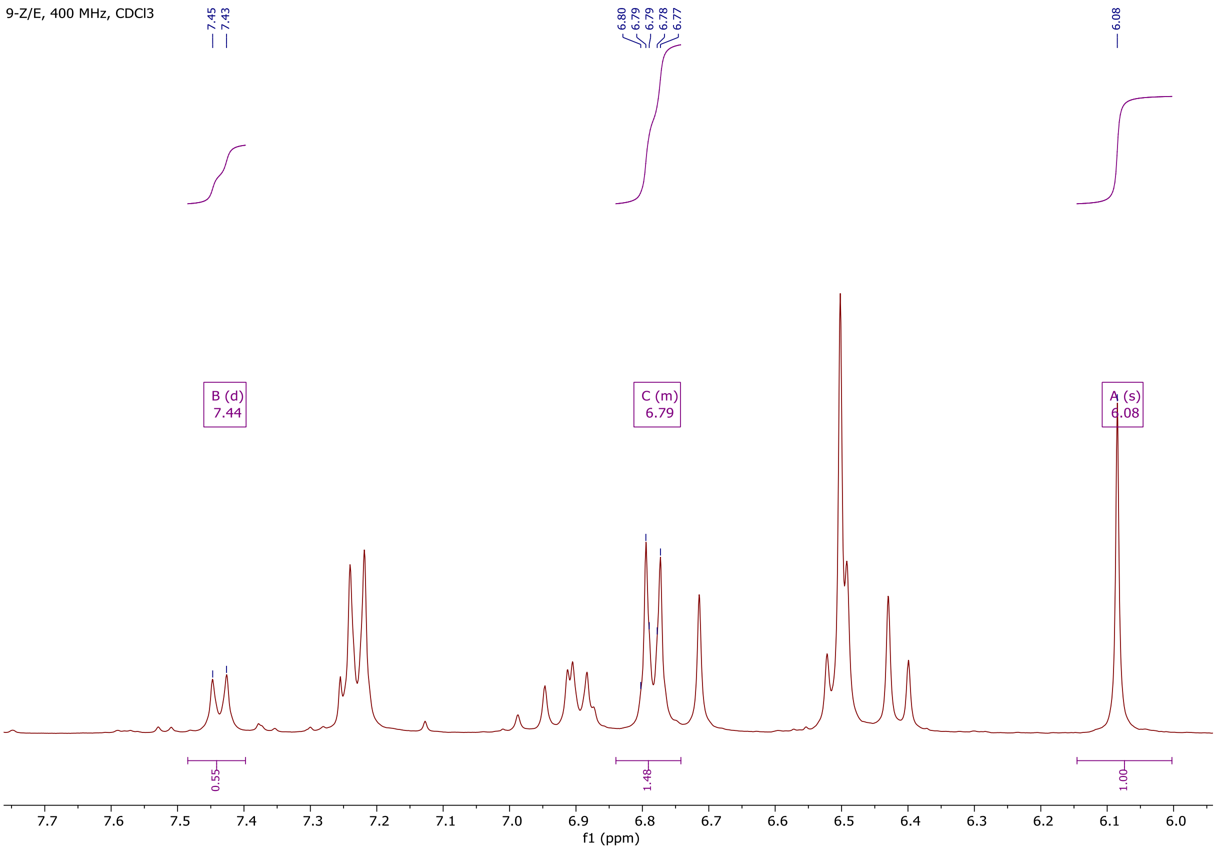

1.1.2. Activity of [Cu]-2 and [Cu]-3 compared to [Cu]-1

In all studied cases **[Cu]-2** and **[Cu]-3** catalysts bearing dipirydylamine ligand turned out to be less active than **[Cu]-1**

Table S6. Activity of [Cu]-2 and [Cu]-3 compared to [Cu]-1

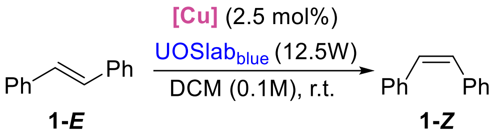

Entry	[Cu]	atm.	*Z*/*E* [15 min]	*Z*/*E* [30 min]	*Z*/*E* [60 min]	*Z*/*E* [120 min]
1	**[Cu]-1**	Air	89/11	95/5	95/5	95/5
2	**[Cu]-1**	Argon	95/5	95/5	95/5	95/5
3	**[Cu]-6**	Air	5/95	8/92	13/87	23/77
4	**[Cu]-6**	Argon	10/90	17/82	28/72	44/56
5	**[Cu]-7**	Air	2/98	5/98	9/91	17/83
6	**[Cu]-7**	Argon	2/98	5/98	11/89	19/81

Reaction conditions: olefin **1-*E*** (0.2 mmol), catalyst (2.5 mol%), UOSlab photoreactor with blue (λ_{max} 450 nm) light irradiation (12.5W power), DCM (0.1M)

Table S7. Activity of [Cu]-1 compared to [Cu]-6 and [Cu]-7

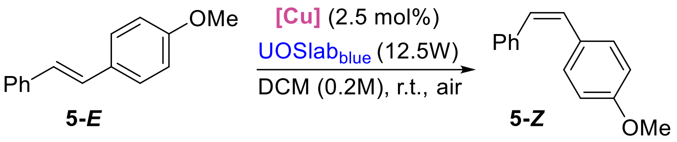

Entry	[Cu]	Z/E [30 min]	*Z/E* [1 h]	*Z/E* [2 h]	*Z/E* [4 h]
1	**[Cu]-1**	31/69	49/51	73/27	82/18
2	**[Cu]-6**	15/85	25/75	40/60	61/39
2	**[Cu]-7**	4/96	7/92	15/85	32/68

Reaction conditions: olefin 5-***E*** (0.2 mmol), catalyst (2.5 mol%), UOSlab photoreactor with blue (λ_{max} 450 nm) light irradiation (12.5W power), DCM (0.2M), on air

Table S8. Activity of [Cu]-1 compared to [Cu]-7

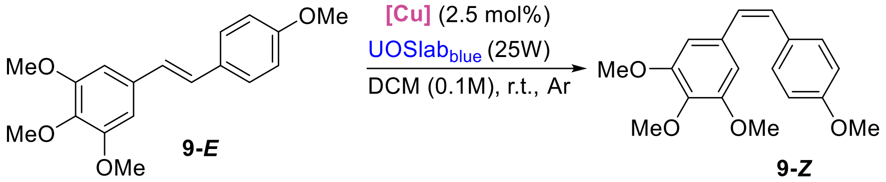

Entry	[Cu]	*Z/E* [15 min]	*Z/E* [30 min]	*Z/E* [60 min]	*Z/E* [120 min]
1	**[Cu]-1**	80/20	88/12	88/12	88/12
2	**[Cu]-7**	11/89	21/79	36/64	56/44

Reaction conditions: olefin 9-***E*** (0.2 mmol), catalyst (2.5 mol%), UOSlab photoreactor with blue (λ_{max} 450 nm) light irradiation (25W power), DCM (0.1M), under argon

1.1.1. Computational methods - estimation of triplet energy values for selected stilbenes. Comparison of observed activity with experimental E_T (emission energy) of catalysts as well as calculated E_T of substrates

Table S9. Estimation of triplet energy values for selected stilbenes.

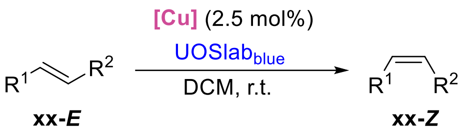

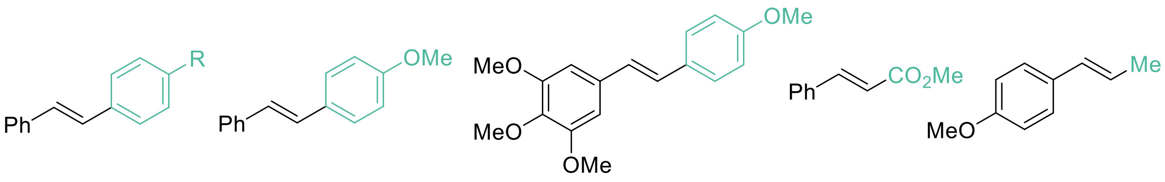

1-*E*	5-*E*	9-*E*	15-*E*	17-*E*	
**214.3**	**210.4**	**208.7**	**246.8**	**256.1**	ET[a] [kJ/mol]

	[Cu]	ET [kJ/mol]	*Z/E* **1** (4 h)[b]	Z/E **5** (4 h)[c]	*Z/E* **9** [2 h][d]	*Z/E* **15** [4 h][b]	*Z/E* **17** [4 h][b]
1	**[Cu]-1**	n.d.	95/5	82/18	88/12	<1/99	<1/99

2	**[Cu]-6**[e]	245	28/72	61/39	n.d.	n.d.	<1/99
3	**[Cu]-7**[e]	253	11/89	32/68	56/44	n.d.	<1/99

[a] see: SI part 4.1.11

[b] conditions 2.5 mol% **[Cu]**, 0.2 mmol **1**, 450nm LED (12.5W), DCM (0.1M), argon

[c] conditions 2.5 mol% **[Cu]**, 0.2 mmol **5**, 450nm LED (12.5W), DCM (0.2M), air

[d] conditions 2.5 mol% **[Cu]**, 0.2 mmol **5**, 450nm LED (25W), DCM (0.2M), argon

[e] experimental,[3] (*ACS Appl. Mater. Interfaces* **2016**, 8, *23*, 14678–14691)

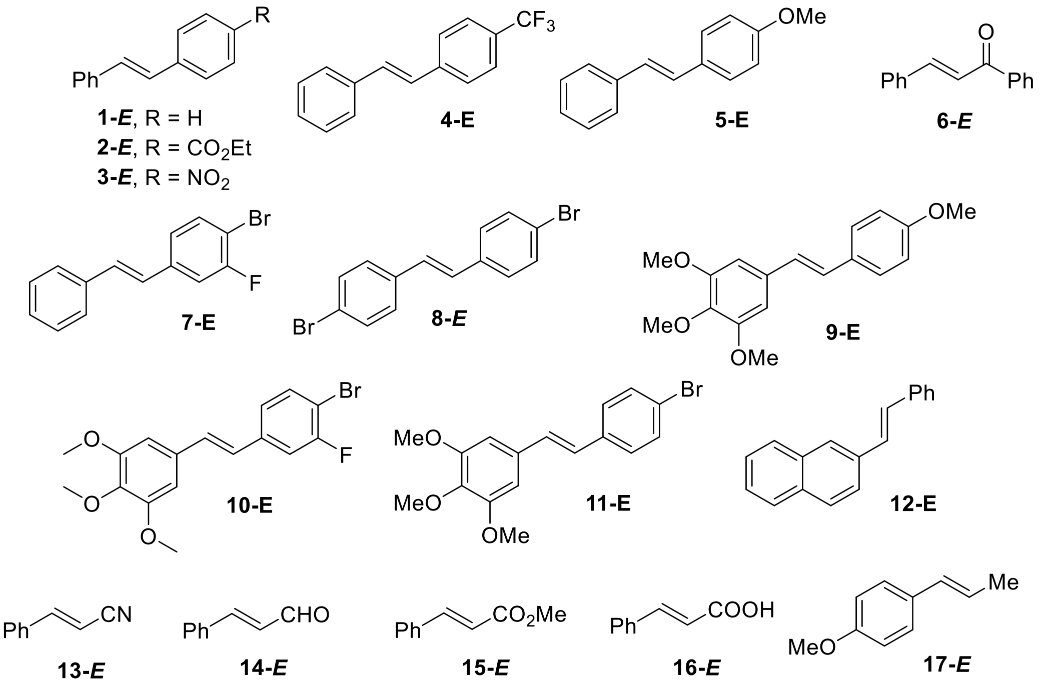

1.1.2. Calculated triplet energy values depending on geometry optimization and the calculation method - Relative Electronic Energies (kJ/mol).

Energy values corresponding to computed intersystem crossing transitions for molecules optimized both in the singlet ground and triplet states are summarized in Table below. For all analyzed compounds, triplet energy values (ET) referring to molecules of geometry optimized in S_0 state, calculated with the use of both TD-B3LYP/6-31G(d,p) and UB3LYP/6-31G(d,p) functionals are substantially higher than those observed for optimal triplet orientation. TD-M06 method considering optimal singlet state S_0 geometries gives the average ET values which correlate well with the experimental data and literature data.

Table S10. Calculated triplet energy values depending on geometry optimization and the calculation method - Relative Electronic Energies (kJ/mol).

	Opt (singlet) – isomer *E*			Opt (singlet) – isomer *Z*			Opt (triplet)
olefin	singlet	triplet (uM06)	triplet (TD-M06)	singlet	Triplet (uM06)	triplet (TD-M06)	Triplet
**1**	0	248.9	214.3	19.6	304.9	266.6	187.5
**11**	0	239.7	206.4	18.4	290.1	253.8	185.4
**10**	0	238.4	205.7	18.1	288.8	253.4	185.4
**9**	0	240.9	208.7	18.9	289.9	254.3	186.2
**5**	0	242.8	210.4	20.1	297.3	261.6	187.7

**17**	0	293.3	256.1	1.0	328.0	287.0	216.1
**8**	0	240.8	206.0	19.5	398.2	259.2	184.9
**15**	0	278.8	246.8	24.8	325.3	291.4	210.8

DFT calculations

Computational methods

All the calculations were performed with Gaussian 16 package.[12] Geometry optimizations (in singlet and triplet states) were computed at B3LYP/6-31G(d) level of theory with the D3 version of Grimme's empirical dispersion correction.[13] Frequency analysis was performed at the same level to provide correction to thermodynamic functions and confirm the nature of optimized structures (no imaginary frequency for minimum). Single point energies were computed at M06/6-311++G(d,p) level of theory with SMD model of solvation (dichloromethane).[14] For geometries optimized in singlet states single point energies were calculated in both singlet and triplet states. Molecular structures were visualized in CYLview.[15]

Optimized geometries, energies and corrections to thermodynamic functions.

Olefin 1, isomer *E* (geometry in singlet state)

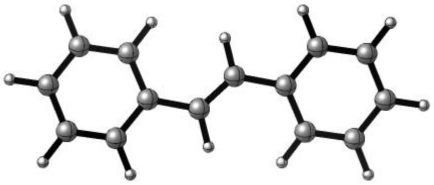

E (B3LYP-D3/6-31G(d)) = -540.727848171
E(singlet) (M06/6-311++G(2df,p)/SMD (dichloromethane)// B3LYP-D3/6-31G(d)) = -540.419342977
E(triplet) (M06/6-311++G(2df,p)/SMD (dichloromethane)// B3LYP-D3/6-31G(d)) = -540.324547036
E(triplet) (TD-M06/6-311++G(2df,p)/SMD (dichloromethane)// B3LYP-D3/6-31G(d)) = -540.3376987

Zero-point correction= 0.215478 (Hartree/Particle)
Thermal correction to Energy=0.226771
Thermal correction to Enthalpy= 0.227716
Thermal correction to Gibbs Free Energy=0.175665

Charge = 0 Multiplicity = 1

C	0.49672300	0.45593700	-0.00539400
H	0.23584600	1.51324500	0.01398100
C	-0.49671900	-0.45593800	-0.00544100
H	-0.23584200	-1.51324800	0.01383500
C	1.93794900	0.19006300	-0.00785000
C	2.82672700	1.27128600	0.14295100
C	2.49110200	-1.09752200	-0.15736000
C	4.20690400	1.07900200	0.15594600
H	2.42288000	2.27507600	0.25550900
C	3.86872600	-1.29046600	-0.14395000
H	1.83832800	-1.95436300	-0.29525400
C	4.73593300	-0.20449300	0.01407600
H	4.86891900	1.93253000	0.27630100
H	4.27069100	-2.29339500	-0.26249300
H	5.81137100	-0.35917500	0.02177000
C	-1.93794900	-0.19006300	-0.00788200
C	-2.82672500	-1.27128500	0.14294200
C	-2.49110600	1.09752100	-0.15739100
C	-4.20690100	-1.07900100	0.15596200
H	-2.42287600	-2.27507500	0.25549800
C	-3.86873000	1.29046500	-0.14395400
H	-1.83833600	1.95435900	-0.29531500
C	-4.73593400	0.20449400	0.01409800
H	-4.86891400	-1.93252700	0.27633400

H	-4.27069800	2.29339300	-0.26249800
H	-5.81137100	0.35917600	0.02181400

Olefin 1, isomer *Z* (geometry in singlet state)

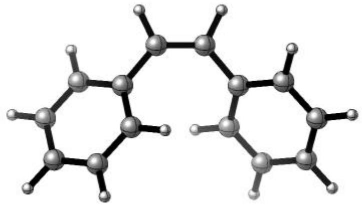

E (B3LYP-D3/6-31G(d)) = -540.722422227
E(singlet) (M06/6-311++G(2df,p)/SMD (dichloromethane)// B3LYP-D3/6-31G(d)) = -540.411866752
E(triplet) (M06/6-311++G(2df,p)/SMD (dichloromethane)// B3LYP-D3/6-31G(d)) = -540.303220935
E(triplet) (TD-M06/6-311++G(2df,p)/SMD (dichloromethane)// B3LYP-D3/6-31G(d)) = -540.317802028

Zero-point correction= 0.215850 (Hartree/Particle)
Thermal correction to Energy= 0.226830
Thermal correction to Enthalpy= 0.227774
Thermal correction to Gibbs Free Energy= 0.177518

Charge = 0 Multiplicity = 1

C	0.67426900	1.86830400	-0.00406800
H	1.15234100	2.84740900	0.03483600
C	-0.67426600	1.86832500	0.00408500
H	-1.15230100	2.84745500	-0.03467900
C	1.62246000	0.74103800	-0.07796600
C	2.86439900	0.84792300	0.57229300
C	1.36579300	-0.41930600	-0.83118400
C	3.80249600	-0.18161100	0.50682200
H	3.08866000	1.74727700	1.14156300
C	2.30503200	-1.44483200	-0.90276800
H	0.42569500	-0.50769600	-1.36530100
C	3.52516400	-1.33480000	-0.22928700
H	4.75194200	-0.08043800	1.02611900
H	2.08742900	-2.33098700	-1.49334400
H	4.25642100	-2.13656000	-0.28705600
C	-1.62247500	0.74107500	0.07797300
C	-2.86434600	0.84789400	-0.57241900
C	-1.36586000	-0.41920400	0.83131000
C	-3.80243100	-0.18165200	-0.50695800
H	-3.08856100	1.74719700	-1.14178700
C	-2.30508800	-1.44474300	0.90288100
H	-0.42581300	-0.50753200	1.36552400
C	-3.52515500	-1.33477800	0.22927100
H	-4.75182700	-0.08053900	-1.02635800
H	-2.08753100	-2.33084900	1.49354800
H	-4.25640700	-2.13654300	0.28702800

Olefin 1 (geometry in triplet state)

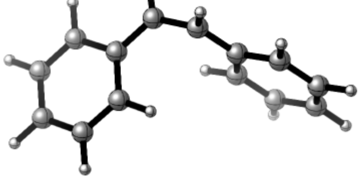

E (B3LYP-D3/6-31G(d)) = -540.656101663
E(triplet) (M06/6-311++G(2df,p)/SMD (dichloromethane)// B3LYP-D3/6-31G(d)) = -540.347930712

Zero-point correction= 0.211746 (Hartree/Particle)
Thermal correction to Energy= 0.223133
Thermal correction to Enthalpy= 0.224077
Thermal correction to Gibbs Free Energy= 0.172055

Charge = 0 Multiplicity = 3

C	0.64610500	1.56688900	0.34804000
H	0.81054800	2.33057100	1.11106000
C	-0.64612500	1.56688900	-0.34803500
H	-0.81057500	2.33058000	-1.11104600
C	1.70669000	0.66315300	0.09553400
C	2.93391700	0.76651600	0.81521300
C	1.59509200	-0.38584600	-0.86478300
C	3.97513900	-0.11865500	0.58709600
H	3.04219100	1.55778400	1.55355100
C	2.64518500	-1.26463800	-1.08377800
H	0.66748700	-0.49040600	-1.41904100
C	3.84211000	-1.14185300	-0.36380100
H	4.90035400	-0.01872100	1.14898100
H	2.53644700	-2.05733900	-1.81970800
H	4.65993300	-1.83509000	-0.53895400
C	-1.70669600	0.66315000	-0.09553800
C	-2.93393700	0.76652900	-0.81518700
C	-1.59507400	-0.38586700	0.86475400
C	-3.97515000	-0.11865100	-0.58706900
H	-3.04222600	1.55781700	-1.55350200
C	-2.64515800	-1.26466900	1.08375100
H	-0.66745500	-0.49043200	1.41899000
C	-3.84209800	-1.14187200	0.36380000
H	-4.90037700	-0.01870700	-1.14893200
H	-2.53640200	-2.05738600	1.81966100
H	-4.65991600	-1.83511400	0.53895400

Olefin 11, isomer *E* (geometry in singlet state)

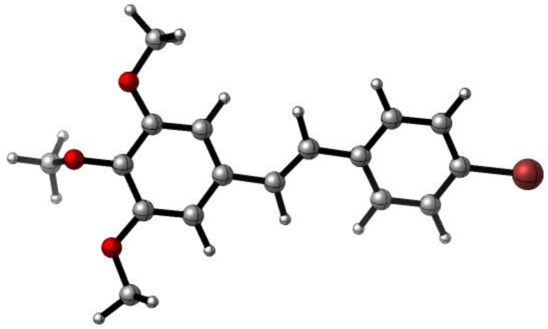

E (B3LYP-D3/6-31G(d)) = -3455.40510566
E(singlet) (M06/6-311++G(2df,p)/SMD (dichloromethane)// B3LYP-D3/6-31G(d)) = -3457.31278125
E(triplet) (M06/6-311++G(2df,p)/SMD (dichloromethane)// B3LYP-D3/6-31G(d)) = -3457.22153724
E(triplet) (TD-M06/6-311++G(2df,p)/SMD (dichloromethane)// B3LYP-D3/6-31G(d)) = -3457.234191

Zero-point correction= 0.303172 (Hartree/Particle)
Thermal correction to Energy= 0.324053
Thermal correction to Enthalpy= 0.324997
Thermal correction to Gibbs Free Energy= 0.249319

Charge = 0 Multiplicity = 1

C	-0.36873700	-0.42727400	-0.02068200
H	-0.06672400	-1.46374300	-0.16538100

C	0.58991900	0.51194800	0.11669700
H	0.29063000	1.53911600	0.32002500
C	-1.81777400	-0.22131700	0.03278300
C	-2.64560900	-1.35431100	0.11053300
C	-2.40185300	1.05759800	0.00402100
C	-4.03497700	-1.21503700	0.18279300
H	-2.19001900	-2.33769300	0.12302500
C	-3.78889600	1.19863600	0.07306400
H	-1.77184700	1.93125700	-0.09970900
C	-4.61540900	0.06396600	0.18382300
C	2.03751500	0.30271600	0.05264600
C	2.89640800	1.35815600	0.41304900
C	2.63170100	-0.90747300	-0.35806600
C	4.28176800	1.21977500	0.38336400
H	2.46869900	2.30650600	0.72954200
C	4.01283900	-1.06213100	-0.39213400
H	2.00876000	-1.74044100	-0.66909900
C	4.83200400	0.00474700	-0.01822000
H	4.92694000	2.04380100	0.66815000
H	4.45499200	-1.99944100	-0.71232100
O	-5.97031600	0.21506300	0.31356200
C	-6.68895300	0.10620500	-0.91724200
H	-6.54902000	-0.88427800	-1.36771700
H	-7.74384800	0.24776400	-0.66981400
H	-6.37312800	0.88379500	-1.62501900
O	-4.91952100	-2.25148700	0.25165400
C	-4.40168600	-3.57203300	0.28214600
H	-5.26982300	-4.23051100	0.34858900
H	-3.75694700	-3.73318000	1.15670800
H	-3.83642600	-3.80810500	-0.63008600
O	-4.45190600	2.38877600	0.02920800
C	-3.68453000	3.57863700	-0.05506900
H	-4.40570800	4.39815500	-0.05656100
H	-3.09180100	3.61582500	-0.97970000
H	-3.01258400	3.68848900	0.80711500
Br	6.73284000	-0.20380000	-0.06783300

Olefin 11, isomer *Z* (geometry in singlet state)

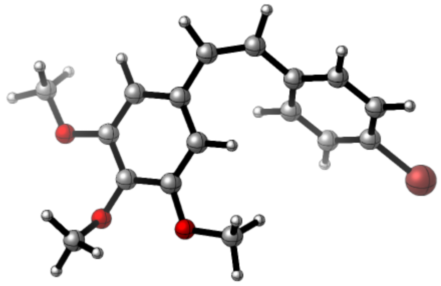

E (B3LYP-D3/6-31G(d)) = -3455.40148650
E(singlet) (M06/6-311++G(2df,p)/SMD (dichloromethane)// B3LYP-D3/6-31G(d)) = -3457.30583918
E(triplet) (M06/6-311++G(2df,p)/SMD (dichloromethane)// B3LYP-D3/6-31G(d)) = -3457.20225880
E(triplet) (TD-M06/6-311++G(2df,p)/SMD (dichloromethane)// B3LYP-D3/6-31G(d)) = -3457.21612281

Zero-point correction= 0.303699 (Hartree/Particle)
Thermal correction to Energy= 0.324198
Thermal correction to Enthalpy= 0.325142
Thermal correction to Gibbs Free Energy= 0.251417

Charge = 0 Multiplicity = 1

C	0.34423700	2.83715100	0.76847000

H	0.67021600	3.82201500	1.10292000
C	-0.98887800	2.63330200	0.72091500
H	-1.61219300	3.50050600	0.93972400
C	1.44727900	1.92008200	0.42376700
C	2.63785400	1.95851700	1.16880000
C	1.38148800	1.03297300	-0.66655800
C	3.70956500	1.11646700	0.87469300
H	2.72266100	2.65091100	2.00285400
C	2.44377400	0.18922400	-0.97670100
H	0.48399600	1.00351100	-1.27464600
C	3.59969800	0.23115900	-0.19501200
H	4.61781000	1.14771100	1.46671200
H	2.38140000	-0.48862800	-1.82126100
C	-1.74702000	1.40268100	0.43444300
C	-3.01349500	1.51809400	-0.16007600
C	-1.25970900	0.13333900	0.78170700
C	-3.76875800	0.37423800	-0.44400800
H	-3.39315500	2.50392800	-0.40248000
C	-2.00230800	-1.01029000	0.48407600
H	-0.30471900	0.05321400	1.28103200
C	-3.25734100	-0.90017600	-0.14356000
O	-3.96881600	-2.01788900	-0.49082300
C	-4.70017200	-2.60622400	0.58734800
H	-5.43928200	-1.90064300	0.98941800
H	-5.21732400	-3.47309700	0.16880600
H	-4.02653500	-2.93366900	1.38833200
O	-5.00883800	0.38293700	-1.01055100
O	-1.60194400	-2.28330900	0.76602000
C	-5.57214000	1.63518400	-1.36496800
H	-4.95518800	2.16229800	-2.10565200
H	-6.54627400	1.40971800	-1.80323000
H	-5.71077100	2.28235400	-0.48761000
C	-0.29170900	-2.46714800	1.28598000
H	0.46956100	-2.03999700	0.61969300
H	-0.18510800	-2.02092600	2.28411100
H	-0.14956100	-3.54703800	1.35758200
Br	5.05877300	-0.93650100	-0.60855400

Olefin 11 (geometry in triplet state)

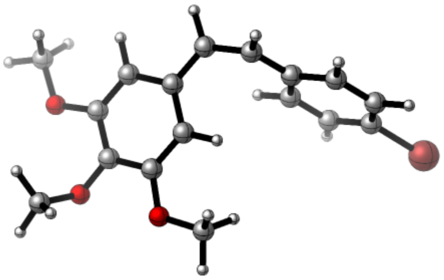

E (B3LYP-D3/6-31G(d)) = -3455.33576872
E(triplet) (M06/6-311++G(2df,p)/SMD (dichloromethane)// B3LYP-D3/6-31G(d)) = -3457.24224429

Zero-point correction= 0.299723 (Hartree/Particle)
Thermal correction to Energy= 0.320650
Thermal correction to Enthalpy= 0.321594
Thermal correction to Gibbs Free Energy= 0.246087

Charge = 0 Multiplicity = 3

C	0.47074400	2.17412400	1.40354900
H	0.56823500	2.59569600	2.40608200
C	-0.82130200	2.33286100	0.72453600

H	-1.11867200	3.34479000	0.44504200
C	1.60527700	1.52652500	0.86677500
C	2.80857800	1.41750000	1.62812900
C	1.60171100	0.93370900	-0.43318400
C	3.92217900	0.76129700	1.13527300
H	2.84417700	1.85992100	2.62068600
C	2.71599400	0.27632000	-0.92767100
H	0.70271000	0.99802400	-1.03666600
C	3.87311800	0.18906600	-0.14369500
H	4.82831600	0.68549300	1.72690500
H	2.69841600	-0.17123300	-1.91580900
C	-1.72428700	1.28725900	0.43891100
C	-2.96630000	1.57254900	-0.20386800
C	-1.41982300	-0.06727300	0.76487400
C	-3.84867400	0.54860900	-0.51480200
H	-3.20044800	2.60316000	-0.44346400
C	-2.30701300	-1.08648700	0.44496900
H	-0.48473400	-0.28101800	1.26391200
C	-3.52525200	-0.79593900	-0.21039700
O	-4.35557300	-1.81457000	-0.58403200
C	-5.45960900	-2.03450900	0.29891100
H	-6.10075800	-1.14681200	0.35541100
H	-6.02614300	-2.86807900	-0.12297300
H	-5.10731900	-2.30395200	1.30312900
O	-5.06039900	0.71487000	-1.12064700
C	-5.44700000	2.03036200	-1.48486900
H	-6.42708300	1.93369200	-1.95578200
H	-4.74189000	2.47367400	-2.20097500
H	-5.52789400	2.68662200	-0.60730600
O	-2.10236200	-2.40574400	0.71539500
C	-0.86178700	-2.77543000	1.29949300
H	-0.88578600	-3.86344200	1.38450400
H	-0.73682700	-2.33553900	2.29856700
H	-0.01419400	-2.47586700	0.66852300
Br	5.40506400	-0.72670900	-0.82277500

Olefin 10, isomer *E* (geometry in singlet state)

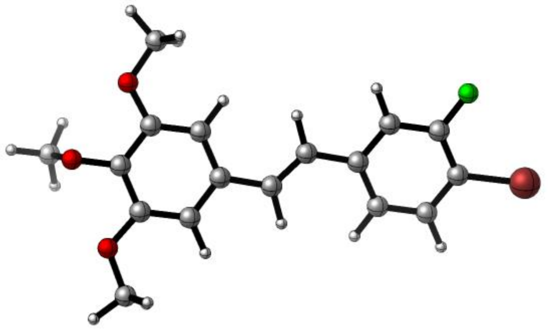

E (B3LYP-D3/6-31G(d)) = -3554.63589427
E(singlet) (M06/6-311++G(2df,p)/SMD (dichloromethane)// B3LYP-D3/6-31G(d)) = -3556.55035861
E(triplet) (M06/6-311++G(2df,p)/SMD (dichloromethane)// B3LYP-D3/6-31G(d)) = -3556.45964873
E(triplet) (TD-M06/6-311++G(2df,p)/SMD (dichloromethane)// B3LYP-D3/6-31G(d)) = -3556.47207045

Zero-point correction= 0.294966 (Hartree/Particle)
Thermal correction to Energy= 0.316708
Thermal correction to Enthalpy= 0.317652
Thermal correction to Gibbs Free Energy= 0.239893

Charge = 0 Multiplicity = 1

C	-0.60030600	-0.52503900	-0.02024200
H	-0.32087800	-1.57250100	-0.12445000
C	0.37909800	0.39851100	0.06929100
H	0.10592500	1.44059600	0.22612400
C	-2.04412000	-0.28807300	0.03380000
C	-2.89373600	-1.40284100	0.13311600
C	-2.60240600	1.00198700	-0.01473600
C	-4.28101800	-1.23692200	0.20072200
H	-2.45729800	-2.39456500	0.15942600
C	-3.98560400	1.17003200	0.05686800
H	-1.95627400	1.86250100	-0.12831600
C	-4.83516600	0.05356700	0.18292700
C	1.82037400	0.15104600	0.00487400
C	2.69992600	1.20959600	0.29368800
C	2.38570100	-1.09487300	-0.33634600
C	4.07370000	1.02253200	0.25372200
H	2.32547700	2.19327500	0.56022500
C	3.76301400	-1.27498000	-0.37329400
H	1.74500800	-1.93311000	-0.58887800
C	4.62291200	-0.21492800	-0.07617000
H	4.18502900	-2.23832800	-0.63909000
O	-6.18772400	0.21976400	0.31287500
C	-6.87970800	0.36943400	-0.92922100
H	-6.75264500	-0.52181100	-1.55757600
H	-7.93646800	0.48890900	-0.67843500
H	-6.52952800	1.25748100	-1.46962700
O	-5.18349600	-2.25478000	0.28217600
C	-4.68976400	-3.58373700	0.33389500
H	-5.57003900	-4.22508600	0.40755000
H	-4.05033400	-3.74407800	1.21264200
H	-4.12670300	-3.84410800	-0.57318200
O	-4.62655000	2.37261200	0.00290400
C	-3.83792600	3.54819000	-0.09432300
H	-4.54417000	4.38047700	-0.10046000
H	-3.24811900	3.56601800	-1.02121500
H	-3.16166600	3.65244900	0.76492000
Br	6.50873300	-0.44100400	-0.12589800
F	4.87770600	2.06058200	0.54018800

Olefin 10, isomer *Z* (geometry in singlet state)

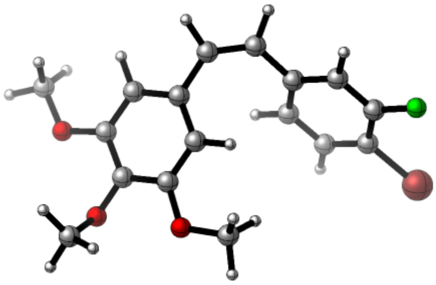

E (B3LYP-D3/6-31G(d)) = -3554.63215796
E(singlet) (M06/6-311++G(2df,p)/SMD (dichloromethane)// B3LYP-D3/6-31G(d)) = -3556.54346171
E(triplet) (M06/6-311++G(2df,p)/SMD (dichloromethane)// B3LYP-D3/6-31G(d)) = -3556.44037352
E(triplet) (TD-M06/6-311++G(2df,p)/SMD (dichloromethane)// B3LYP-D3/6-31G(d)) = -3556.45390281

Zero-point correction= 0.295502 (Hartree/Particle)
Thermal correction to Energy= 0.316861
Thermal correction to Enthalpy= 0.317805
Thermal correction to Gibbs Free Energy= 0.242085

Charge = 0 Multiplicity = 1

C	0.21777000	2.79638100	0.60446800
H	0.57728600	3.78225800	0.89815200
C	-1.12054500	2.62412600	0.59547700
H	-1.71600300	3.51404200	0.80000400
C	1.28777300	1.84072400	0.26131100
C	2.49590600	1.88183300	0.97482200
C	1.17324500	0.91859100	-0.79614700
C	3.52754600	1.00551400	0.66528800
H	2.64500500	2.58486400	1.78863000
C	2.21229500	0.04654000	-1.10354400
H	0.25920100	0.88555600	-1.37756900
C	3.39884900	0.07834200	-0.36693800
H	2.11507900	-0.65914000	-1.92173200
C	-1.91587200	1.40498000	0.36912900
C	-3.19147300	1.53375900	-0.20275800
C	-1.45362100	0.13630600	0.75111400
C	-3.98164500	0.40116400	-0.43103900
H	-3.55072400	2.52035800	-0.47163300
C	-2.23217300	-0.99683200	0.51029600
H	-0.49066500	0.04848800	1.23361100
C	-3.49687300	-0.87514100	-0.09558500
O	-4.24408600	-1.98459700	-0.38816000
C	-4.96852500	-2.51533800	0.72444700
H	-5.68059100	-1.77654700	1.11534600
H	-5.51633100	-3.38205300	0.34672900
H	-4.28734400	-2.83247300	1.52311600
O	-5.23233600	0.42266200	-0.97231400
O	-1.86002200	-2.26921300	0.82996500
C	-5.77155900	1.67629000	-1.35875600
H	-5.15751100	2.16121400	-2.12999900
H	-6.76016800	1.46096300	-1.76867200
H	-5.87502200	2.35647300	-0.50180700
C	-0.54638900	-2.47005000	1.33479300
H	0.21420700	-2.08926600	0.64014400
H	-0.41026000	-1.99041200	2.31361600
H	-0.43231100	-3.55004400	1.44429500
Br	4.82793500	-1.11056300	-0.76397600
F	4.66352600	1.06238500	1.38141600

Olefin 10 (geometry in triplet state)

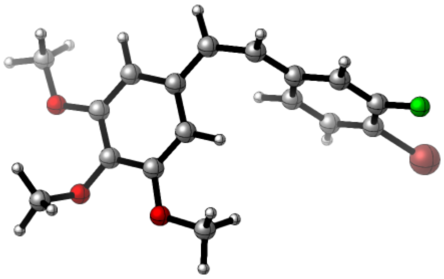

E (B3LYP-D3/6-31G(d)) = -3554.56648442
E(triplet) (M06/6-311++G(2df,p)/SMD (dichloromethane)// B3LYP-D3/6-31G(d)) = -3556.47981902

Zero-point correction= 0.291494 (Hartree/Particle)
Thermal correction to Energy= 0.313291
Thermal correction to Enthalpy= 0.314235
Thermal correction to Gibbs Free Energy= 0.236765

Charge = 0 Multiplicity = 3

C	0.31441800	2.17822200	1.24393100

H	0.44371600	2.62926000	2.22970500
C	-0.99218500	2.33104200	0.59257300
H	-1.28355400	3.33750100	0.28864700
C	1.42674800	1.50104400	0.69698400
C	2.64582600	1.40321900	1.43119200
C	1.38040100	0.86982600	-0.58398700
C	3.72337900	0.71858600	0.91310000
H	2.74358800	1.86308300	2.40966700
C	2.47486100	0.18667800	-1.08593800
H	0.46633400	0.92559400	-1.16412200
C	3.66056300	0.09972500	-0.34422000
H	2.42713800	-0.29040600	-2.05918600
C	-1.91576900	1.28891400	0.36707600
C	-3.17079300	1.57047700	-0.25159400
C	-1.62101800	-0.05807500	0.73137700
C	-4.07536100	0.54969900	-0.50339700
H	-3.39712400	2.59560400	-0.52032400
C	-2.53106100	-1.07427800	0.47189500
H	-0.67586300	-0.26886000	1.21241900
C	-3.76269200	-0.78828300	-0.16049700
O	-4.61613000	-1.80681400	-0.47600200
C	-5.70386900	-1.97652700	0.43817100
H	-6.33107800	-1.07796800	0.47413300
H	-6.29079000	-2.81708100	0.06052200
H	-5.33332100	-2.21276500	1.44411700
O	-5.29991000	0.71281100	-1.08288000
C	-5.67864700	2.02073900	-1.48180400
H	-6.67213400	1.92194000	-1.92314800
H	-4.98708900	2.42921900	-2.23102300
H	-5.72725600	2.70728700	-0.62534600
O	-2.33837000	-2.38594100	0.78321100
C	-1.09000600	-2.75398600	1.35169300
H	-1.12881600	-3.83751000	1.47788600
H	-0.93448900	-2.27940700	2.33035100
H	-0.25330000	-2.49216900	0.69010700
Br	5.16408900	-0.84220000	-1.01530600
F	4.85749400	0.64234500	1.63192500

Olefin 9, isomer *E* (geometry in singlet state)

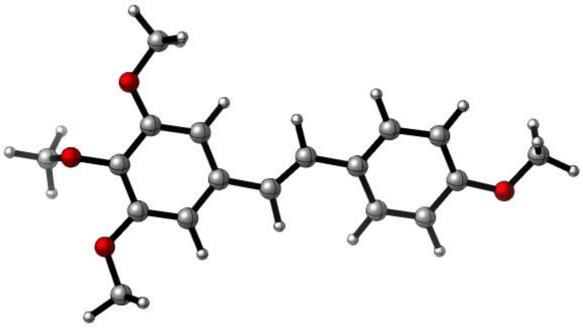

E (B3LYP-D3/6-31G(d)) = -998.824801113
E(singlet) (M06/6-311++G(2df,p)/SMD (dichloromethane)// B3LYP-D3/6-31G(d)) = -998.378774593
E(triplet) (M06/6-311++G(2df,p)/SMD (dichloromethane)// B3LYP-D3/6-31G(d)) = -998.294180641
E(triplet) (TD-M06/6-311++G(2df,p)/SMD (dichloromethane)// B3LYP-D3/6-31G(d)) = -998.306478867

Zero-point correction=	0.346013 (Hartree/Particle)
Thermal correction to Energy=	0.368003
Thermal correction to Enthalpy=	0.368947
Thermal correction to Gibbs Free Energy=	0.291602

Charge = 0 Multiplicity = 1

C	1.15463500	-2.53389800	-0.80011200
H	1.57861000	-3.45871800	-1.19265600
C	-0.19283600	-2.44993400	-0.81767400
H	-0.72222400	-3.34634200	-1.14139700
C	2.16032700	-1.57006400	-0.31842300
C	3.40445000	-1.48849200	-0.95914800
C	1.95862600	-0.74911900	0.81104500
C	4.39910300	-0.60246600	-0.53853900
H	3.60058500	-2.12553700	-1.81875600
C	2.93598600	0.13221500	1.24513000
H	1.01950200	-0.81007700	1.35003500
C	4.16398500	0.22049400	0.56821500
H	5.34133000	-0.56841800	-1.07326500
H	2.78147800	0.75819500	2.11860300
C	-1.07673600	-1.31897600	-0.48380300
C	-2.36278200	-1.59243900	0.00884400
C	-0.68942700	0.01322800	-0.69554800
C	-3.23757200	-0.54667000	0.32547100
H	-2.66410300	-2.62446200	0.14671600
C	-1.55206300	1.05854700	-0.36306900
H	0.28516400	0.21691100	-1.11560300
C	-2.82892800	0.78799800	0.16268700
O	-3.66195900	1.80805800	0.54366100
O	5.05326900	1.12378000	1.07396200
C	-4.38180000	2.40692900	-0.53537800
H	-5.02600900	1.66999000	-1.03337400
H	-5.00279700	3.18980700	-0.09265400
H	-3.69704200	2.85357600	-1.26661400
C	6.31840600	1.24485500	0.44274200
H	6.21969100	1.56267800	-0.60444100
H	6.86071000	2.00988300	1.00164400
H	6.88085400	0.30180100	0.47973300
O	-4.50631200	-0.71135100	0.79989600
O	-1.25309300	2.38157500	-0.51603300
C	-4.97050600	-2.03321000	1.01622700
H	-4.35502900	-2.56362800	1.75589500
H	-5.98755200	-1.93251400	1.40044400
H	-4.99162300	-2.61567100	0.08439800
C	0.06600100	2.71865600	-0.92330300
H	0.81811400	2.29982900	-0.24148200
H	0.27663100	2.37126100	-1.94413300
H	0.11525900	3.80901200	-0.89715600

Olefin 9, isomer *Z* (geometry in singlet state)

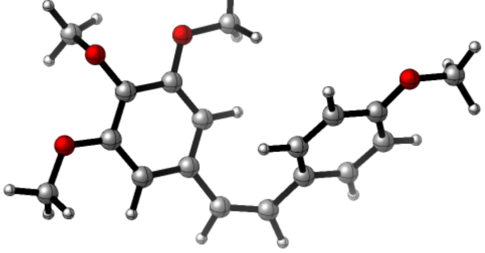

E (B3LYP-D3/6-31G(d)) = -998.821050691
E(singlet) (M06/6-311++G(2df,p)/SMD (dichloromethane)// B3LYP-D3/6-31G(d)) = -998.385951563
E(triplet) (M06/6-311++G(2df,p)/SMD (dichloromethane)// B3LYP-D3/6-31G(d)) = -998.275546412
E(triplet) (TD-M06/6-311++G(2df,p)/SMD (dichloromethane)// B3LYP-D3/6-31G(d)) = -998.289095659

Zero-point correction= 0.346632 (Hartree/Particle)
Thermal correction to Energy= 0.368214
Thermal correction to Enthalpy= 0.369159
Thermal correction to Gibbs Free Energy= 0.294265

Charge = 0 Multiplicity = 1

C	0.36631900	-0.53946100	-0.05058200
H	0.62110900	-1.58714300	-0.20506300
C	1.36577900	0.35746400	0.08089200
H	1.10849900	1.39578600	0.28793300
C	-1.07225100	-0.27019500	0.01958300
C	-1.95000400	-1.36490900	0.10172100
C	-1.60152900	1.03286100	0.00167300
C	-3.33114400	-1.16477400	0.18824300
H	-1.53839000	-2.36759600	0.10535100
C	-2.98015400	1.23424400	0.08702500
H	-0.93500500	1.87854800	-0.10580500
C	-3.85521500	0.13778500	0.20204600
C	2.80304100	0.09692500	0.00180300
C	3.70589200	1.12701400	0.30982200
C	3.35441500	-1.14804800	-0.37441500
C	5.08865200	0.94509400	0.26358300
H	3.31726100	2.10081700	0.59974000
C	4.72342500	-1.34622500	-0.42459600
H	2.69895600	-1.97172300	-0.64107600
C	5.60658800	-0.30133300	-0.10410800
H	5.74231600	1.77286700	0.51336400
H	5.14364300	-2.30375000	-0.71631600
O	-5.20273000	0.34588500	0.34516800
O	6.93420300	-0.60422500	-0.18767900
C	7.87444100	0.41331900	0.11993700
H	7.76893600	0.76135200	1.15667500
H	8.85944700	-0.03904100	-0.00977300
H	7.77751000	1.27193100	-0.55867400
C	-5.92750700	0.30833200	-0.88528600
H	-5.83273000	-0.67283300	-1.36758200
H	-6.97509900	0.48848500	-0.63098700
H	-5.57892000	1.09268500	-1.56996200
O	-4.26003600	-2.16281800	0.25984200
C	-3.79922300	-3.50389700	0.27572200
H	-4.69405600	-4.12551100	0.34564400
H	-3.15358600	-3.69986200	1.14263900
H	-3.25267000	-3.75742700	-0.64333600
O	-3.59050400	2.45415400	0.05497200
C	-2.77123400	3.60789400	-0.03263400
H	-3.45420900	4.45966100	-0.02224400
H	-2.18729300	3.62317200	-0.96358000
H	-2.08494400	3.68306800	0.82204900

Olefin 9 (geometry in triplet state)

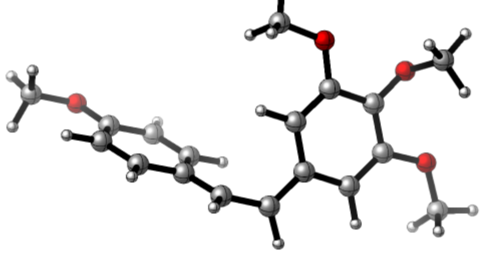

E (B3LYP-D3/6-31G(d)) = -998.755037987
E(triplet) (M06/6-311++G(2df,p)/SMD (dichloromethane)// B3LYP-D3/6-31G(d)) = -998.315017744

Zero-point correction=	0.342557 (Hartree/Particle)
Thermal correction to Energy=	0.364614
Thermal correction to Enthalpy=	0.365558
Thermal correction to Gibbs Free Energy=	0.288479

Charge = 0 Multiplicity = 3

C	-1.25094500	-1.86398700	1.39146000
H	-1.43964300	-2.16387200	2.42429400
C	0.07400600	-2.16124200	0.83180300
H	0.33134000	-3.21022800	0.67295000
C	-2.30870300	-1.23058100	0.70143900
C	-3.55740500	-0.98494700	1.34243300
C	-2.18571900	-0.78338500	-0.65255300
C	-4.60403600	-0.34213700	0.69775200
H	-3.68934700	-1.31127100	2.37144400
C	-3.22532600	-0.14361600	-1.29473800
H	-1.24948100	-0.94965200	-1.17477600
C	-4.44687800	0.08722100	-0.63167100
H	-5.53444300	-0.17761300	1.22962600
H	-3.12811200	0.19640100	-2.32137900
C	1.05490300	-1.19969900	0.50626300
C	2.31893200	-1.61233700	-0.01196700
C	0.80627100	0.19482200	0.66696600
C	3.27679800	-0.67172500	-0.36166500
H	2.51058300	-2.67308100	-0.12468900
C	1.76848800	1.12900800	0.30687000
H	-0.14825900	0.50382800	1.07022900
C	3.00887700	0.71117900	-0.22509500
O	3.92173800	1.64093200	-0.64135900
O	-5.40168900	0.73165800	-1.36213200
C	4.94671800	1.92663500	0.31358500
H	5.53223500	1.02780600	0.54243400
H	5.59467700	2.67621900	-0.14745600
H	4.51666400	2.33511700	1.23749300
C	-6.65762100	0.98821600	-0.75298400
H	-6.55480200	1.63524300	0.12899600
H	-7.25780300	1.50047100	-1.50734400
H	-7.16197600	0.05760300	-0.45894000
O	4.51783300	-0.96128100	-0.85341700
C	4.85261500	-2.32542300	-1.04774300
H	5.86738500	-2.32961700	-1.45050500
H	4.17527300	-2.80892000	-1.76485300
H	4.83389500	-2.88655400	-0.10305300
O	1.61976500	2.47946600	0.42267600
C	0.36371200	2.96722000	0.86960500
H	0.43999100	4.05596500	0.84080700
H	0.14560600	2.64541500	1.89740400
H	-0.45308200	2.63921300	0.21260400

Olefin 5, isomer *E* (geometry in singlet state)

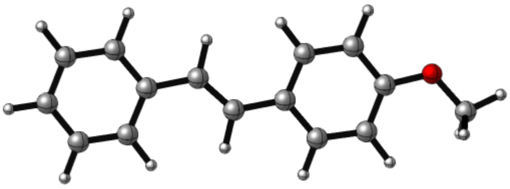

E (B3LYP-D3/6-31G(d)) = -655.255382479

E(singlet) (M06/6-311++G(2df,p)/SMD (dichloromethane)// B3LYP-D3/6-31G(d)) = -654.914173779
E(triplet) (M06/6-311++G(2df,p)/SMD (dichloromethane)// B3LYP-D3/6-31G(d)) = -654.821700933
E(triplet) (TD-M06/6-311++G(2df,p)/SMD (dichloromethane)// B3LYP-D3/6-31G(d)) = -654.834045938

Zero-point correction= 0.248256 (Hartree/Particle)
Thermal correction to Energy= 0.261206
Thermal correction to Enthalpy= 0.262151
Thermal correction to Gibbs Free Energy= 0.207801

Charge = 0 Multiplicity = 1

C	-0.44953300	-0.43725700	-0.00005000
H	-0.69707400	-1.49811800	0.00008200
C	-1.45640900	0.46084300	-0.00011400
H	-1.21047900	1.52193700	-0.00008500
C	0.98626200	-0.15812000	-0.00006100
C	1.89216200	-1.23076100	0.00053800
C	1.53379000	1.14418400	-0.00065700
C	3.27432800	-1.03951100	0.00059300
H	1.50644600	-2.24788200	0.00099000
C	2.90201500	1.35213400	-0.00060800
H	0.87666500	2.00858300	-0.00120300
C	3.78841700	0.26153800	0.00002500
H	3.93039000	-1.90229400	0.00107300
H	3.31940800	2.35425800	-0.00107600
C	-2.89393400	0.17708300	-0.00013400
C	-3.79751900	1.25695000	0.00047300
C	-3.43296600	-1.12556000	-0.00074900
C	-5.17582500	1.05096300	0.00052000
H	-3.40642400	2.27207900	0.00093400
C	-4.80859300	-1.33216400	-0.00069900
H	-2.77021500	-1.98585200	-0.00131900
C	-5.69016100	-0.24621500	-0.00005900
H	-5.84804600	1.90510100	0.00100600
H	-5.19770000	-2.34719500	-0.00118800
O	5.11465500	0.57953000	0.00000600
C	6.05853400	-0.48053800	0.00068400
H	5.95938100	-1.11048100	-0.89392800
H	7.04189900	-0.00659800	0.00059600
H	5.95910600	-1.10957900	0.89590000
H	-6.76400500	-0.41161200	-0.00003300

Olefin 5, isomer *Z* (geometry in singlet state)

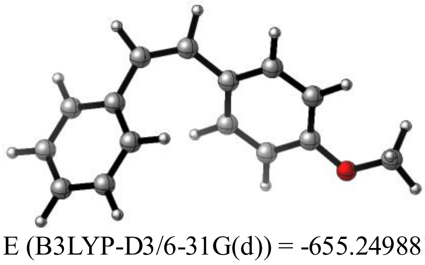

E (B3LYP-D3/6-31G(d)) = -655.249881979
E(singlet) (M06/6-311++G(2df,p)/SMD (dichloromethane)// B3LYP-D3/6-31G(d)) = -654.906499518
E(triplet) (M06/6-311++G(2df,p)/SMD (dichloromethane)// B3LYP-D3/6-31G(d)) = -654.800944368
E(triplet) (TD-M06/6-311++G(2df,p)/SMD (dichloromethane)// B3LYP-D3/6-31G(d)) = -654.81452568

Zero-point correction= 0.248746 (Hartree/Particle)
Thermal correction to Energy= 0.262260
Thermal correction to Enthalpy= 0.263204
Thermal correction to Gibbs Free Energy= 0.207056

Charge = 0 Multiplicity = 1

C	-1.81548700	1.88511600	0.00518100
H	-2.47083100	2.75538700	-0.03656900
C	-0.49071000	2.14261200	-0.00124800
H	-0.21292100	3.19639000	0.03854300
C	-2.53301600	0.59887600	0.08027600
C	-3.77124400	0.46481500	-0.57245800
C	-2.06502600	-0.48843200	0.84127600
C	-4.49882500	-0.72254800	-0.50134700
H	-4.15943600	1.30218600	-1.14811800
C	-2.79386500	-1.67251900	0.91771600
H	-1.12642300	-0.39463700	1.37734500
C	-4.01125800	-1.79876900	0.24213100
H	-5.44894100	-0.80518800	-1.02285300
H	-2.41409200	-2.49838900	1.51375600
C	0.65886200	1.22616600	-0.07825500
C	1.87355700	1.59422100	0.51797000
C	0.62937500	0.00840600	-0.78986500
C	3.00664300	0.78077200	0.45655100
H	1.93597300	2.53870800	1.05398300
C	1.74641600	-0.80717700	-0.86698900
H	-0.28323400	-0.29184600	-1.29307300
C	2.94468500	-0.43289400	-0.23716600
H	3.92023800	1.10394900	0.94209200
H	1.72400000	-1.74132000	-1.41953700
O	3.98129100	-1.31009800	-0.37130800
C	5.22093000	-0.98159300	0.23617700
H	5.12372600	-0.88207600	1.32605400
H	5.89525000	-1.80947000	0.00885200
H	5.63832500	-0.05138200	-0.17326800
H	-4.57818700	-2.72384700	0.30357100

Olefin 5 (geometry in triplet state)

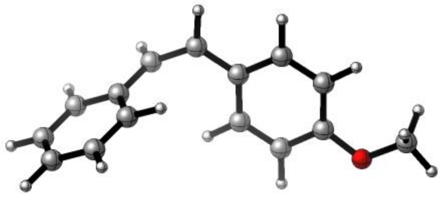

E (B3LYP-D3/6-31G(d)) = -655.183783788
E(triplet) (M06/6-311++G(2df,p)/SMD (dichloromethane)// B3LYP-D3/6-31G(d)) = -654.842688422

Zero-point correction=	0.244659 (Hartree/Particle)
Thermal correction to Energy=	0.258606
Thermal correction to Enthalpy=	0.259550
Thermal correction to Gibbs Free Energy=	0.201539

Charge = 0 Multiplicity = 3

C	-1.70147400	1.61241200	-0.42953400
H	-1.97489300	2.29543200	-1.23696400
C	-0.43101200	1.85003400	0.26660700
H	-0.38686100	2.68353900	0.97046500
C	-2.61319900	0.57099100	-0.12943600
C	-3.83039300	0.43845200	-0.86150800
C	-2.35503200	-0.38971400	0.89318000
C	-4.72451500	-0.58377100	-0.58701400
H	-4.04893400	1.15835200	-1.64703000
C	-3.25868400	-1.40774000	1.15809100

H	-1.43157500	-0.31358800	1.45875600
C	-4.44869800	-1.51637100	0.42451500
H	-5.64522600	-0.66283400	-1.15965900
H	-3.03921800	-2.12904100	1.94136100
C	0.74985100	1.09578000	0.07819200
C	1.94580800	1.41856400	0.78024000
C	0.80851900	-0.02655000	-0.80582100
C	3.11426800	0.68749300	0.62070100
H	1.93933200	2.26544700	1.46244500
C	1.96910700	-0.75410700	-0.96668100
H	-0.08561200	-0.30886300	-1.35264300
C	3.13690700	-0.40917500	-0.25800700
H	3.99930200	0.97257700	1.17834100
H	2.01058200	-1.60730500	-1.63696400
O	4.22354400	-1.19902000	-0.49292700
C	5.43166500	-0.90464000	0.19148400
H	5.30843700	-0.98154100	1.28044200
H	6.15507700	-1.65070000	-0.14278000
H	5.80365300	0.09841900	-0.05826500
H	-5.15111800	-2.31766900	0.63584600

Olefin 17, isomer *E* (geometry in singlet state)

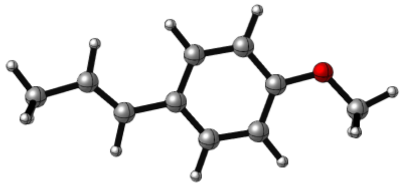

E (B3LYP-D3/6-31G(d)) = -463.503158110
E(singlet) (M06/6-311++G(2df,p)/SMD (dichloromethane)// B3LYP-D3/6-31G(d)) = -463.268177990
E(triplet) (M06/6-311++G(2df,p)/SMD (dichloromethane)// B3LYP-D3/6-31G(d)) = -463.156482522
E(triplet) (TD-M06/6-311++G(2df,p)/SMD (dichloromethane)// B3LYP-D3/6-31G(d)) = -463.170618632

Zero-point correction= 0.194405 (Hartree/Particle)
Thermal correction to Energy= 0.204731
Thermal correction to Enthalpy= 0.205675
Thermal correction to Gibbs Free Energy= 0.157598

Charge = 0 Multiplicity = 1

C	-2.33514100	-0.51074100	-0.00017000
H	-2.56971000	-1.57750100	-0.00052200
C	-3.36197600	0.35249200	0.00020700
H	-3.16536600	1.42354800	0.00061300
C	-0.89878900	-0.20384100	-0.00013700
C	0.02855700	-1.25571200	-0.00008300
C	-0.38013100	1.10866800	-0.00016700
C	1.40748500	-1.03471600	-0.00002200
H	-0.33485700	-2.28111200	-0.00007700
C	0.98404700	1.34694400	-0.00010400
H	-1.05679100	1.95812400	-0.00026900
C	1.89352700	0.27636900	-0.00002300
H	2.08166000	-1.88352300	0.00002000
H	1.37922600	2.35813200	-0.00013400
O	3.21420900	0.62322400	0.00002100
C	4.17865900	-0.41712300	0.00019700
H	4.09260200	-1.04918000	-0.89450600
H	5.15271200	0.07596800	0.00028300
H	4.09238700	-1.04907500	0.89495400
C	-4.81342600	-0.06603500	0.00014700

H	-5.48013400	0.80187300	0.00007900
H	-5.06109200	-0.67048200	-0.88238500
H	-5.06118400	-0.67039900	0.88271200

Olefin 17, isomer *Z* (geometry in singlet state)

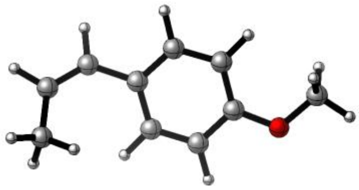

E (B3LYP-D3/6-31G(d)) = -463.503008034
E(singlet) (M06/6-311++G(2df,p)/SMD (dichloromethane)// B3LYP-D3/6-31G(d)) = -463.267785006
E(triplet) (M06/6-311++G(2df,p)/SMD (dichloromethane)// B3LYP-D3/6-31G(d)) = -463.143249534
E(triplet) (TD-M06/6-311++G(2df,p)/SMD (dichloromethane)// B3LYP-D3/6-31G(d)) = -463.158864434

Zero-point correction=	0.195438 (Hartree/Particle)
Thermal correction to Energy=	0.206001
Thermal correction to Enthalpy=	0.206946
Thermal correction to Gibbs Free Energy=	0.158995

Charge = 0 Multiplicity = 1

C	-3.52568700	0.33103400	0.01194600
H	-4.46039600	0.86992500	-0.14214200
C	-2.39410900	0.98220900	-0.30809900
H	-2.49983600	2.01196400	-0.65144300
C	-1.00143900	0.51000100	-0.22690900
C	0.02119600	1.43064100	0.04923600
C	-0.61775800	-0.82810400	-0.45198600
C	1.35973600	1.04900600	0.14257400
H	-0.23694000	2.47540400	0.20752800
C	0.70953700	-1.22464700	-0.36723500
H	-1.36734000	-1.56121400	-0.72999000
C	1.71013300	-0.29148400	-0.06030800
H	2.11140600	1.79703700	0.36728600
H	1.00209200	-2.25429400	-0.54837500
O	2.98199000	-0.78484200	0.00206800
C	4.03669200	0.11685200	0.29732800
H	3.90679100	0.58344700	1.28361300
H	4.95057900	-0.48041800	0.29946300
H	4.12114400	0.90496100	-0.46368800
C	-3.66907800	-1.04698900	0.59741900
H	-4.48124700	-1.06473900	1.33435700
H	-3.92495000	-1.79269200	-0.16946100
H	-2.75257200	-1.38175700	1.09251100

Olefin 17 (geometry in triplet state)

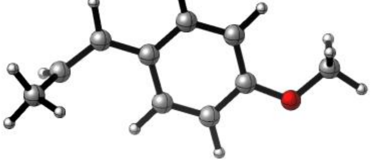

E (B3LYP-D3/6-31G(d)) = -463.420069210
E(triplet) (M06/6-311++G(2df,p)/SMD (dichloromethane)// B3LYP-D3/6-31G(d)) = -463.185882329

Zero-point correction=	0.190381 (Hartree/Particle)
Thermal correction to Energy=	0.201791

Thermal correction to Enthalpy= 0.202735
Thermal correction to Gibbs Free Energy= 0.151808

Charge = 0 Multiplicity = 3

C	-3.44337200	-0.03954900	-0.44326200
H	-3.86062100	-0.19289800	-1.43977900
C	-2.30764400	0.86844600	-0.27336900
H	-2.51379800	1.93867200	-0.17185500
C	-0.95174900	0.46853000	-0.19373400
C	0.08711800	1.42049500	0.00290000
C	-0.55276700	-0.89888600	-0.30281500
C	1.42229900	1.04906400	0.08687400
H	-0.17643000	2.47205500	0.09013300
C	0.77272300	-1.27217000	-0.21966200
H	-1.31874000	-1.65370700	-0.45760100
C	1.77827000	-0.30533000	-0.02345700
H	2.17721300	1.81264500	0.23752300
H	1.07238800	-2.31254400	-0.30322100
O	3.05367800	-0.78661100	0.04418300
C	4.11187900	0.13818700	0.23960400
H	4.00669600	0.67853900	1.19046400
H	5.02840200	-0.45456100	0.26184100
H	4.17058700	0.86547700	-0.58183500
C	-4.21122100	-0.56032400	0.73471800
H	-4.89416800	-1.36891200	0.45033700
H	-3.53434400	-0.93313700	1.51543800
H	-4.81982300	0.23048200	1.20831100

Olefin 8, isomer *E* (geometry in singlet state)

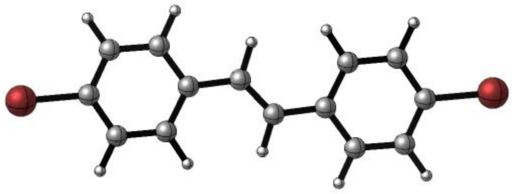

E (B3LYP-D3/6-31G(d)) = -5682.94286471
E(singlet) (M06/6-311++G(2df,p)/SMD (dichloromethane)// B3LYP-D3/6-31G(d)) = -5687.26229776
E(triplet) (M06/6-311++G(2df,p)/SMD (dichloromethane)// B3LYP-D3/6-31G(d)) = -5687.17060849
E(triplet) (TD-M06/6-311++G(2df,p)/SMD (dichloromethane)// B3LYP-D3/6-31G(d)) = -5687.18385373

Zero-point correction= 0.195381 (Hartree/Particle)
Thermal correction to Energy= 0.209636
Thermal correction to Enthalpy= 0.210580
Thermal correction to Gibbs Free Energy= 0.148594

Charge = 0 Multiplicity = 1

C	0.47123100	-0.48250400	0.00973000
H	0.15467700	-1.52433500	-0.00149700
C	-0.47122800	0.48247800	0.00965700
H	-0.15467100	1.52430600	-0.00171700
C	1.92313600	-0.29504400	0.01127800
C	2.75395700	-1.42791700	-0.07466400
C	2.54731800	0.96559000	0.09665800

C	4.14228000	-1.32026800	-0.08270400
H	2.30228300	-2.41491000	-0.13923300
C	3.93156500	1.08988200	0.08966500
H	1.94740500	1.86682000	0.17593500
C	4.72321300	-0.05689400	-0.00125200
H	4.76599700	-2.20486000	-0.15108200
H	4.39770500	2.06691300	0.15699100
C	-1.92313400	0.29502500	0.01122700
C	-2.75394900	1.42790400	-0.07468500
C	-2.54732300	-0.96560700	0.09659400
C	-4.14227300	1.32026700	-0.08269200
H	-2.30226900	2.41489500	-0.13925000
C	-3.93157100	-1.08988800	0.08963400
H	-1.94741600	-1.86684400	0.17582200
C	-4.72321300	0.05689600	-0.00124200
H	-4.76598500	2.20486400	-0.15104300
H	-4.39771700	-2.06691700	0.15694700
Br	6.62732400	0.11166500	-0.00952800
Br	-6.62732500	-0.11164900	-0.00947500

Olefin 8, isomer *Z* (geometry in singlet state)

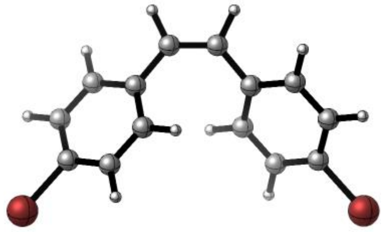

E (B3LYP-D3/6-31G(d)) = -5682.93764293
E(singlet) (M06/6-311++G(2df,p)/SMD (dichloromethane)// B3LYP-D3/6-31G(d)) = -5687.25487614
E(triplet) (M06/6-311++G(2df,p)/SMD (dichloromethane)// B3LYP-D3/6-31G(d)) = -5687.14870476
E(triplet) (TD-M06/6-311++G(2df,p)/SMD (dichloromethane)// B3LYP-D3/6-31G(d)) = -5687.16355863

Zero-point correction=	0.195798 (Hartree/Particle)
Thermal correction to Energy=	0.209662
Thermal correction to Enthalpy=	0.210607
Thermal correction to Gibbs Free Energy=	0.150994

Charge = 0 Multiplicity = 1

C	0.67367100	3.02495700	0.02931400
H	1.15111100	4.00267800	0.09260900
C	-0.67367000	3.02495900	-0.02930900
H	-1.15110800	4.00268100	-0.09260500
C	1.61869300	1.89416300	0.00065500
C	2.82614000	1.98938100	0.71363000
C	1.39688600	0.73749200	-0.76888500
C	3.76380300	0.95865300	0.69842100
H	3.03122500	2.88262400	1.29862800
C	2.32615200	-0.29762900	-0.79976300
H	0.48844800	0.65214000	-1.35531700
C	3.50244000	-0.18207600	-0.05776200
H	4.68677900	1.04047700	1.26203700
H	2.14556700	-1.18310300	-1.39948400
C	-1.61869400	1.89416500	-0.00065400
C	-2.82612800	1.98937200	-0.71365100
C	-1.39689800	0.73750600	0.76890700
C	-3.76379100	0.95864300	-0.69844300
H	-3.03120400	2.88260700	-1.29866500
C	-2.32616400	-0.29761600	0.79978400

H	-0.48847000	0.65216300	1.35535400
C	-3.50244000	-0.18207400	0.05776200
H	-4.68675800	1.04045900	-1.26207500
H	-2.14558900	-1.18308100	1.39952100
Br	-4.78106700	-1.60412800	0.09245400
Br	4.78106700	-1.60413000	-0.09245500

Olefin 8 (geometry in triplet state)

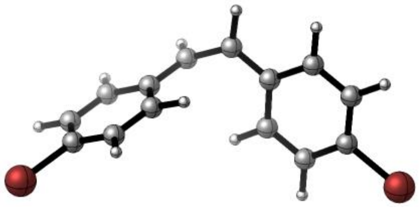

E (B3LYP-D3/6-31G(d)) = -5682.87222190
E(triplet) (M06/6-311++G(2df,p)/SMD (dichloromethane)// B3LYP-D3/6-31G(d)) = -5687.19186075
E(triplet) (TD-M06/6-311++G(2df,p)/SMD (dichloromethane)// B3LYP-D3/6-31G(d)) = -537.216633176

Zero-point correction= 0.191833 (Hartree/Particle)
Thermal correction to Energy= 0.206108
Thermal correction to Enthalpy= 0.207052
Thermal correction to Gibbs Free Energy= 0.145638

Charge = 0 Multiplicity = 3

C	0.61512900	2.58372700	0.40031900
H	0.72366200	3.35872200	1.16136300
C	-0.61512900	2.58372800	-0.40031900
H	-0.72366200	3.35872200	-1.16136300
C	1.68171500	1.66888700	0.24818600
C	2.84571400	1.76641000	1.06803700
C	1.64340800	0.60905600	-0.70707500
C	3.89488000	0.87269400	0.94531500
H	2.90463900	2.56299900	1.80553500
C	2.69301100	-0.28590100	-0.83162100
H	0.76926400	0.50413100	-1.34164800
C	3.81638000	-0.15346100	-0.00613900
H	4.77323500	0.95909300	1.57597500
H	2.65044900	-1.08803600	-1.56071000
C	-1.68171600	1.66888700	-0.24818600
C	-2.84571400	1.76641000	-1.06803700
C	-1.64340700	0.60905600	0.70707500
C	-3.89488000	0.87269400	-0.94531500
H	-2.90463900	2.56299900	-1.80553500
C	-2.69301100	-0.28590100	0.83162100
H	-0.76926400	0.50413100	1.34164800
C	-3.81638000	-0.15346100	0.00613900
H	-4.77323500	0.95909300	-1.57597400
H	-2.65044800	-1.08803600	1.56071000
Br	5.26001000	-1.39044000	-0.17489200
Br	-5.26001000	-1.39044000	0.17489200

Olefin 15, isomer *E* (geometry in singlet state)

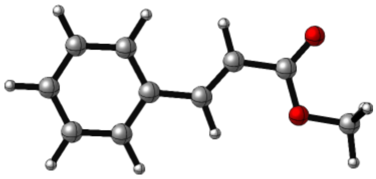

E (B3LYP-D3/6-31G(d)) = -537.544764799

E(singlet) (M06/6-311++G(2df,p)/SMD (dichloromethane)// B3LYP-D3/6-31G(d)) = -537.310647148
E(triplet) (M06/6-311++G(2df,p)/SMD (dichloromethane)// B3LYP-D3/6-31G(d)) = -537.204457503
E(triplet) (TD-M06/6-311++G(2df,p)/SMD (dichloromethane)// B3LYP-D3/6-31G(d)) = -537.199661174

Zero-point correction= 0.177665 (Hartree/Particle)
Thermal correction to Energy= 0.188749
Thermal correction to Enthalpy= 0.189693
Thermal correction to Gibbs Free Energy= 0.139160

Charge = 0 Multiplicity = 1

C	1.07040000	0.73459000	-0.00013900
H	0.78986800	1.78290900	-0.00038100
C	0.16387000	-0.26075000	0.00011300
H	0.54188500	-1.28109900	0.00025800
C	-1.29474600	-0.13054500	0.00007200
C	-2.07518500	-1.30106900	-0.00019400
C	-1.95905400	1.11165800	0.00029500
C	-3.46739400	-1.23858600	-0.00026300
H	-1.57822600	-2.26838600	-0.00035000
C	-3.34830300	1.17458900	0.00022600
H	-1.38482500	2.03309900	0.00057100
C	-4.10927800	0.00046300	-0.00006500
H	-4.04970900	-2.15590200	-0.00047400
H	-3.84271900	2.14220400	0.00041900
H	-5.19447300	0.05418900	-0.00011400
C	2.53055600	0.53085300	-0.00008600
O	2.89220900	-0.77840200	0.00042900
O	3.33804000	1.44164500	-0.00046600
C	4.30716500	-1.01438700	0.00008200
H	4.77215400	-0.57565700	-0.88774300
H	4.77185800	-0.57922300	0.88985200
H	4.42400200	-2.09897300	-0.00197900

Olefin 15, isomer *Z* (geometry in singlet state)

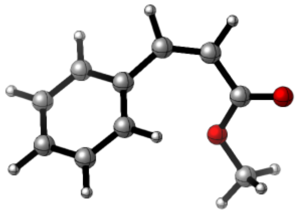

E (B3LYP-D3/6-31G(d)) = -537.536280980
E(singlet) (M06/6-311++G(2df,p)/SMD (dichloromethane)// B3LYP-D3/6-31G(d)) = -537.301216655
E(triplet) (M06/6-311++G(2df,p)/SMD (dichloromethane)// B3LYP-D3/6-31G(d)) = -537.186743142

Zero-point correction= 0.177726 (Hartree/Particle)
Thermal correction to Energy= 0.188657
Thermal correction to Enthalpy= 0.189601
Thermal correction to Gibbs Free Energy= 0.139415

Charge = 0 Multiplicity = 1

C	1.44258600	1.57770800	0.17596500
H	2.05539300	2.46790800	0.28486200
C	0.10297700	1.72886000	0.15873000
H	-0.24073200	2.76404000	0.18367800
C	-0.99626300	0.75084200	0.12218800
C	-2.18012400	1.10581100	-0.54904500
C	-0.95180800	-0.48640000	0.78772000
C	-3.26597900	0.23399500	-0.59945000

H	-2.23978500	2.07111700	-1.04663000
C	-2.04292900	-1.35027800	0.75236600
H	-0.06323500	-0.75997300	1.34359500
C	-3.20005800	-0.99936500	0.05136100
H	-4.16550400	0.52149000	-1.13695800
H	-1.99339700	-2.29924500	1.27966400
H	-4.04892900	-1.67726200	0.02460200
C	2.28520500	0.36643600	0.05198300
O	1.64627700	-0.70620800	-0.46812500
O	3.46574800	0.35575000	0.34783700
C	2.45220800	-1.88442300	-0.61738400
H	1.78361000	-2.64350400	-1.02505300
H	3.28582200	-1.69697300	-1.30043500
H	2.85566300	-2.20305100	0.34837300

Olefin 15 (geometry in triplet state)

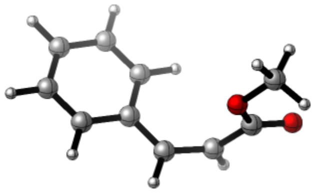

E (B3LYP-D3/6-31G(d)) = -537.462255573
E(triplet) (M06/6-311++G(2df,p)/SMD (dichloromethane)// B3LYP-D3/6-31G(d)) = -537.230362763

Zero-point correction=	0.173657 (Hartree/Particle)
Thermal correction to Energy=	0.185023
Thermal correction to Enthalpy=	0.185967
Thermal correction to Gibbs Free Energy=	0.133773

Charge = 0 Multiplicity = 3

C	1.26772900	-1.21462400	-0.68882500
H	1.42096900	-1.76904200	-1.61351800
C	0.07076200	-1.41100600	0.12451700
H	0.09717300	-2.20837500	0.86893100
C	-1.10868400	-0.63325800	0.02579300
C	-2.23188400	-0.91618800	0.85532400
C	-1.22250700	0.45292800	-0.88941700
C	-3.39181900	-0.16239700	0.76912000
H	-2.16675200	-1.73866000	1.56365000
C	-2.38855100	1.19906700	-0.96675800
H	-0.37765200	0.69381200	-1.52894900
C	-3.48204400	0.90022300	-0.14168400
H	-4.23574300	-0.39744600	1.41247300
H	-2.45260100	2.02360300	-1.67205700
H	-4.39259700	1.48896700	-0.20615100
C	2.35009500	-0.29490400	-0.35955600
O	2.12892000	0.37251700	0.80316000
O	3.34432600	-0.13084700	-1.05284300
C	3.15520100	1.30561200	1.16697200
H	4.11523500	0.79595600	1.29493500
H	3.26858600	2.07548500	0.39743900
H	2.82762300	1.74961400	2.10779700

4.2. Electron transfer reaction - [Cu]-three coordinated complex as photoredox catalysts

4.2.1. General procedure for photoreduction of aryl halides

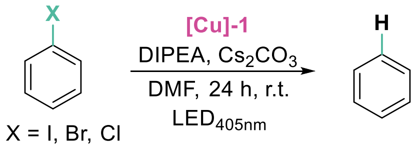

In a 5 mL snap vial with magnetic stirring bar the respective aryl halide (0.25 mmol, 1 equiv.) and photocatalyst **[Cu]-1** (5 mol%) were dissolved in dry DMF (2 mL) and the resulting mixture was degassed argon via a syringe needle. DIPEA (2 equiv.) and Cs_2CO_3 (1.1 equiv.) were added under Ar atmosphere and the reaction mixture was irradiated through the plane bottom side of the snap vial using a 405 nm LED (UOSlab photoreactor). The reaction was stirred under light irradiation for 24 hours. Photoreduction yields were calculated from GC measurements using internal standards (dodecane or anthracene).

4.2.2. Optimization and background studies

Table S11. Optimization and background studies

No	Concentration [mmol/ml]	Photocatalyst, Catalyst loading	Additives	Time [h]	Light source	Yield [%]
1	0.0625	**[Cu]-1**, 2.5 mol%	DIPEA (2eq), Cs_2CO_3 (1 eq)	19h	390 nm	35
2	0.0625	-	DIPEA (2eq), Cs_2CO_3 (1 eq)	19h	390 nm	0
3	0.0625	**[Cu]-1**, 2.5 mol%	-	19h	390 nm	0
4	0.0625	**[Cu]-1**, 2.5 mol%	pyridine (40 mol%)	24h	405 nm	0
5	0.0625	**[Cu]-1**, 5 mol%	DIPEA (2eq), Cs_2CO_3 (1 eq)	19h	405 nm	26
6	0.0625	phen, 5 mol%	DIPEA (2eq), Cs_2CO_3 (1 eq)	19h	390 nm	0
7	0.0625	phen, 5 mol%	DIPEA (2eq), Cs_2CO_3 (1 eq)	19h	405 nm	13
8	0.0625	iPrCuCl, 5 mol%	DIPEA (2eq), Cs_2CO_3 (1 eq)	19h	390 nm	10
9	0.0625	iPrCuCl, 5 mol%	DIPEA (2eq), Cs_2CO_3 (1 eq)	19h	405 nm	0
10	0.0625	**[Cu]-1**, 2.5 mol%	DIPEA (2eq), Cs_2CO_3 (1 eq)	19h	405 nm	32
11	0.083	**[Cu]-1**, 2.5 mol%	DIPEA (2eq), Cs_2CO_3 (1 eq)	19h	405 nm	40
12	0.125	**[Cu]-1**, 2.5 mol%	DIPEA (2eq), Cs_2CO_3 (1 eq)	19h	405 nm	62
13	0.25	**[Cu]-1**, 2.5 mol%	DIPEA (2eq), Cs_2CO_3 (1 eq)	19h	405 nm	31
**14**	**0.25**	**[Cu]-1, 2.5 mol%**	**DIPEA (2.1 eq), Cs_2CO_3 (1.1 eq)**	**24h**	**405 nm**	**65**

4.2.3. Scope and limitations studies – according to general procedure for photoreduction of aryl halides

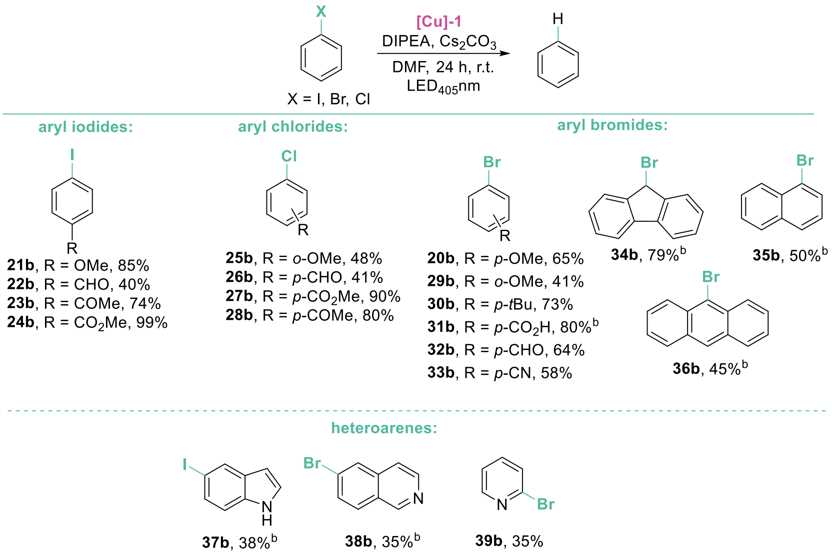

Anisole (21b), (synthesized from 1-iodo-4-methoxybenzene, GC yield = 85%, full substrate conversion, too volatile to isolate at the scale 0.25 mmol without any loss, GC yield established from three calibration points with commercial anisole and dodecane as standard).

GC chromatogram of reaction mixture:

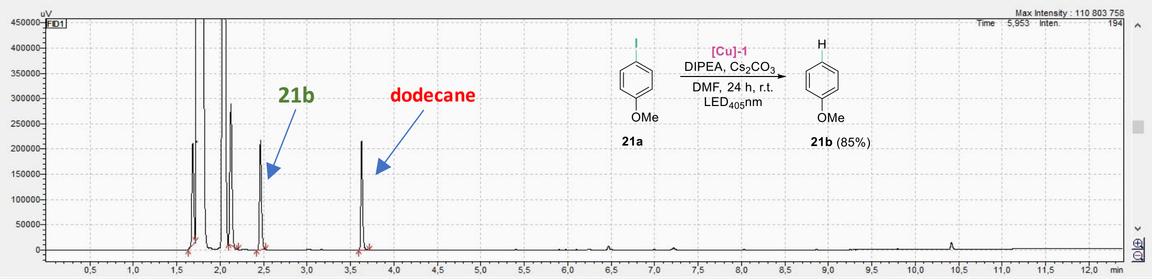

Results View - Peak Table

Peak#	Ret. Time	Area	Height	Mark	Conc.	Unit	ID#	Name	Area%
1	1,684	296451	198695	M	21,316				21,316
2	2,122	467556	277238	M	33,619				33,619
3	2,463	336617	214804	M	24,204				24,204
4	3,627	290142	203176	M	20,862				20,862
Total		1390767	893912		100,000				100,000

Benzaldehyde (22b), (synthesized from 4-iodobenzaldehyde, GC yield = 40%, full substrate conversion, too volatile to isolate at the scale 0.25 mmol without any loss, GC yield established from three calibration points with commercial benzaldehyde and dodecane as standard).

GC chromatogram of reaction mixture:

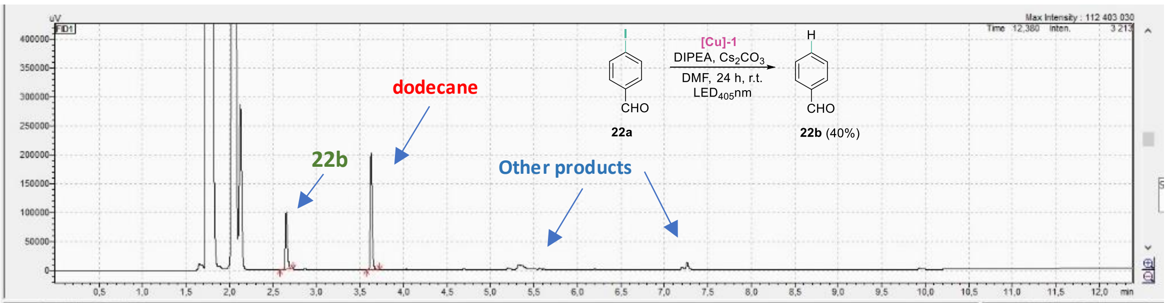

Results View - Peak Table

Peak#	Ret. Time	Area	Height	Mark	Conc.	Unit	ID#	Name	Area%
1	2,653	155051	98367	M	35,599				35,599
2	3,628	280494	198368	M	64,401				64,401
Total		435546	296735		100,000				100,000

Acetophenone (**23b**), (synthesized from 1-(4-iodophenyl)ethan-1-one, GC yield = 74%, full substrate conversion, too volatile to isolate at the scale 0.25 mmol without any loss, GC yield established from three calibration points with commercial acetophenone and dodecane as standard)
GC chromatogram of reaction mixture:

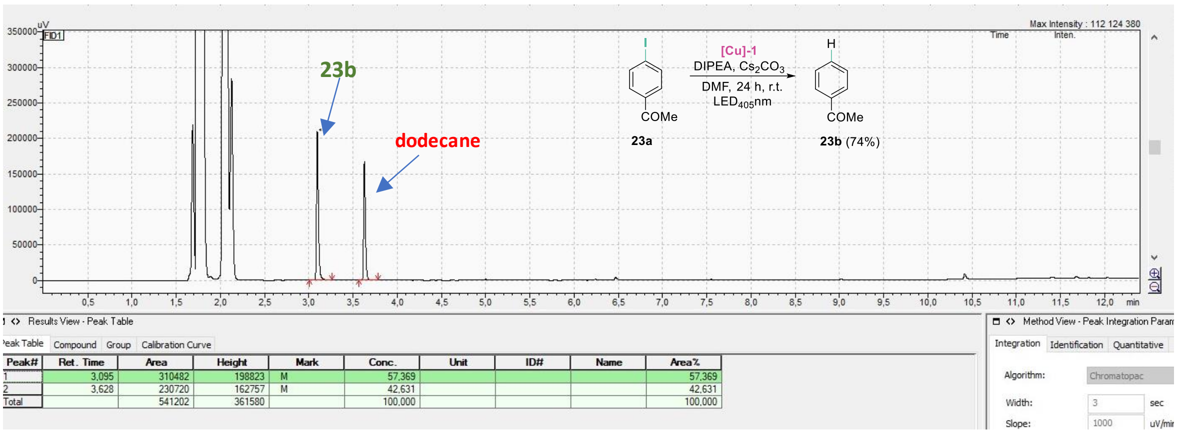

Peak#	Ret. Time	Area	Height	Mark	Conc.	Unit	ID#	Name	Area%
1	3,095	310482	198823	M	57,369				57,369
2	3,628	230720	162757	M	42,631				42,631
Total		541202	361580		100,000				100,000

Methyl benzoate (**24b**), (synthesized from methyl 4-iodobenzoate, GC yield = 99%, full substrate conversion, too volatile to isolate at the scale 0.25 mmol without any loss, GC yield established from three calibration points with commercial methyl benzoate and dodecane as standard).
GC chromatogram of reaction mixture:

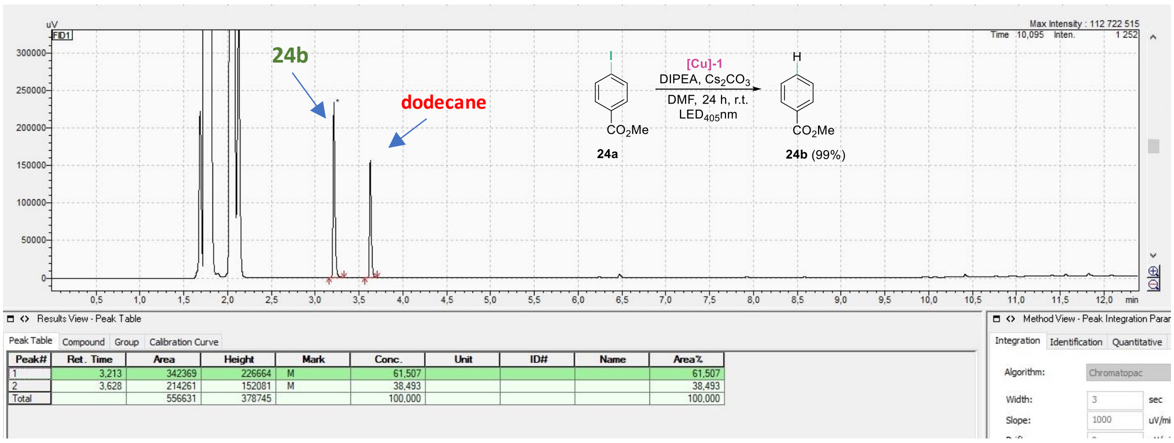

Peak#	Ret. Time	Area	Height	Mark	Conc.	Unit	ID#	Name	Area%
1	3,213	342369	226664	M	61,507				61,507
2	3,628	214261	152081	M	38,493				38,493
Total		556631	378745		100,000				100,000

Anisole (**25b**), (synthesized from 1-chloro-2-methoxybenzene, GC yield = 48%, full substrate conversion, too volatile to isolate at the scale 0.25 mmol without any loss, GC yield established from three calibration points with commercial anisole and dodecane as standard).
GC chromatogram of reaction mixture:

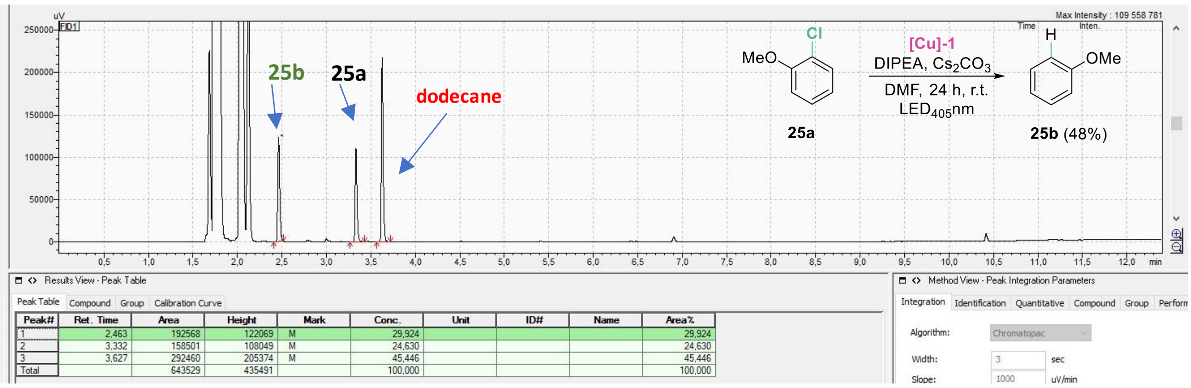

Peak#	Ret. Time	Area	Height	Mark	Conc.	Unit	ID#	Name	Area%
1	2,463	192568	122069	M	29,924				29,924
2	3,332	158501	108049	M	24,630				24,630
3	3,627	292460	205374	M	45,446				45,446
Total		643529	435491		100,000				100,000

Benzaldehyde (**26b**), (synthesized from 4-chlorobenzaldehyde, GC yield = 40%, full substrate conversion, too volatile to isolate at the scale 0.25 mmol without any loss, GC yield established from three calibration points with commercial benzaldehyde and dodecane as standard).

GC chromatogram of reaction mixture:

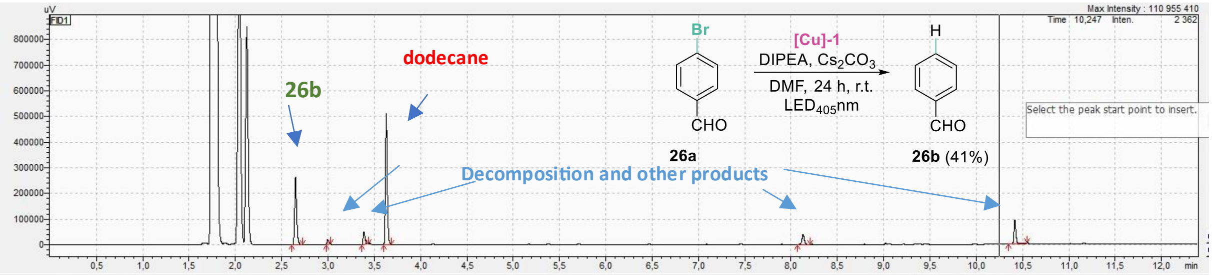

Results View - Peak Table

Peak#	Ret. Time	Area	Height	Mark	Conc.	Unit	ID#	Name	Area%
1	2,650	400973	259522	M	29,514				29,514
2	2,998	23875	17552	M	1,757				1,757
3	3,388	70862	47509	M	5,216				5,216
4	3,629	690864	502062	M	50,852				50,852
5	8,130	69187	37971	M	5,093				5,093
6	10,416	102809	88139	M	7,567				7,567
Total		1358570	952755		100,000				100,000

Methyl benzoate (**27b**), (synthesized from methyl 4-chlorobenzoate, GC yield = 90%, full substrate conversion, too volatile to isolate at the scale 0.25 mmol without any loss, GC yield established from three calibration points with commercial methyl benzoate and dodecane as standard).

GC chromatogram of reaction mixture:

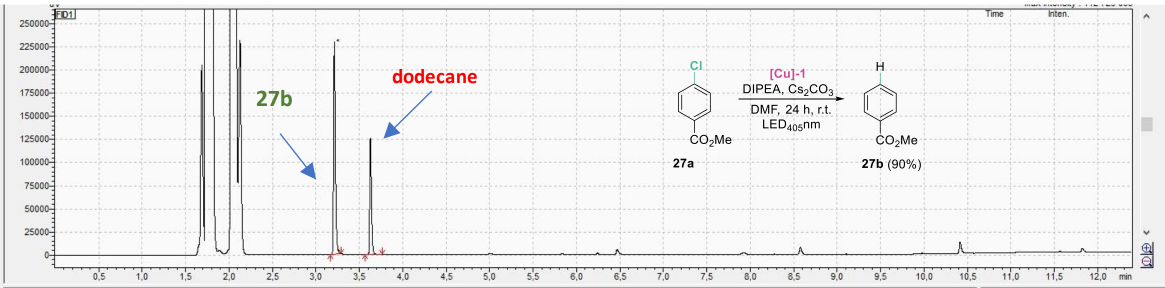

Results View - Peak Table

Peak#	Ret. Time	Area	Height	Mark	Conc.	Unit	ID#	Name	Area%
1	3,213	335208	222337	M	65,716				65,716
2	3,628	174877	121307	M	34,284				34,284
Total		510084	343644		100,000				100,000

Acetophenone (**28b**), (synthesized from 1-(4-chlorophenyl)ethan-1-one, GC yield = 80%, too volatile to isolate at the scale 0.25 mmol without any loss, GC yield established from three calibration points with commercial acetophenone and dodecane as standard)

GC chromatogram of reaction mixture:

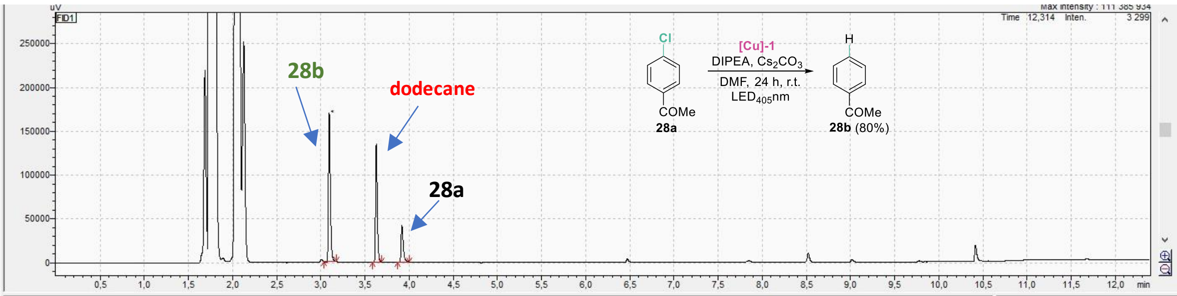

Results View - Peak Table

Peak#	Ret. Time	Area	Height	Mark	Conc.	Unit	ID#	Name	Area%
1	3,094	248183	163051	M	49,356				49,356
2	3,627	184150	130347	M	36,622				36,622
3	3,918	70509	41462	M	14,022				14,022
Total		502842	334860		100,000				100,000

Anisole (**20b**), (synthesized from 1-bromo-2-methoxybenzene, GC yield = 65%, too volatile to isolate at the scale 0.25 mmol without any loss, GC yield established from three calibration points with commercial anisole and dodecane as standard).

GC chromatogram of reaction mixture:

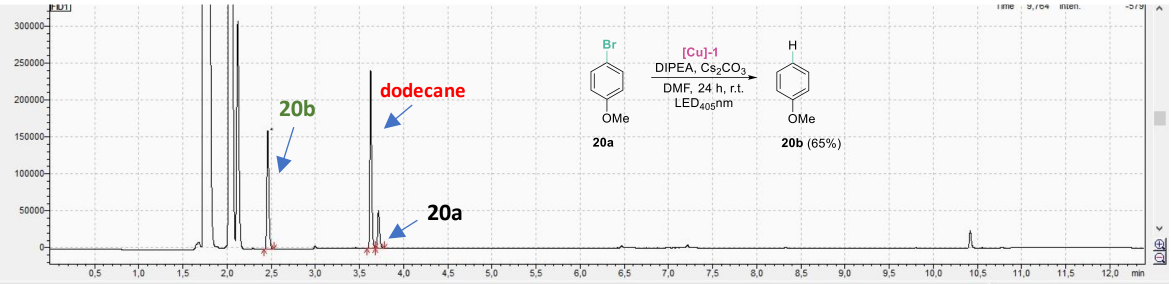

Results View - Peak Table

Peak#	Ret. Time	Area	Height	Mark	Conc.	Unit	ID#	Name	Area%
1	2,461	250846	153444	M	38,629				38,629
2	3,626	323204	234444	M	49,772				49,772
3	3,715	75323	49891	M	11,599				11,599
Total		649373	437778		100,000				100,000

Anisole (**29b**), (synthesized from 1-bromo-2-methoxybenzene, GC yield = 41%, too volatile to isolate at the scale 0.25 mmol without any loss, GC yield established from three calibration points with commercial anisole and dodecane as standard).

GC chromatogram of reaction mixture:

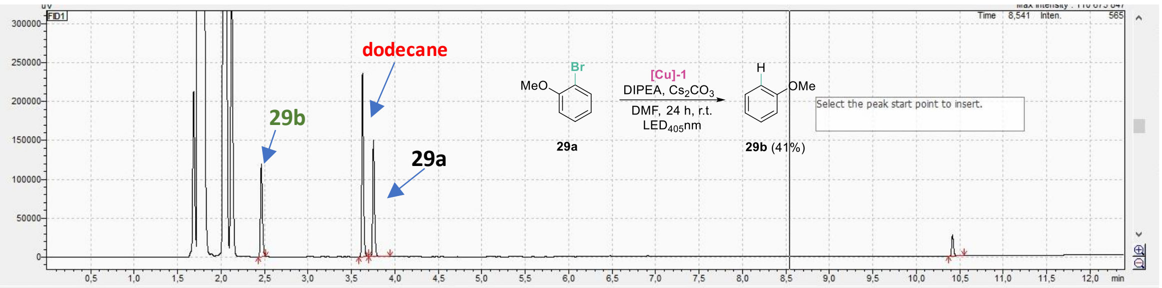

Results View - Peak Table

Peak#	Ret. Time	Area	Height	Mark	Conc.	Unit	ID#	Name	Area%
1	2,463	185604	118100	M	24,466				24,466
2	3,627	316286	226036	M	41,691				41,691
3	3,753	216951	142378	M	28,598				28,598
4	10,416	39793	25932	M	5,245				5,245
Total		758635	512447		100,000				100,000

***Tert*-butylbenzene** (**30b**), (synthesized from 1-Bromo-4-(tert-butyl)benzene, GC yield = 73%, too volatile to isolate at the scale 0.25 mmol without any loss, GC yield established from three calibration points with commercial *t*-butylbenzene and dodecane as standard).

GC chromatogram of reaction mixture:

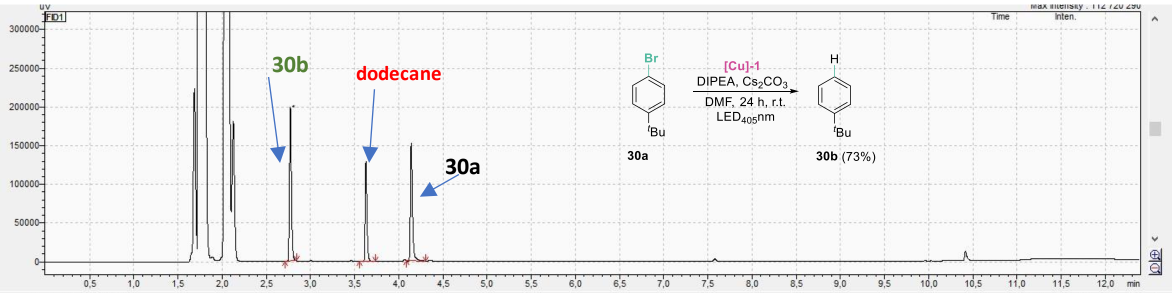

Results View - Peak Table

Peak#	Ret. Time	Area	Height	Mark	Conc.	Unit	ID#	Name	Area%
1	2,772	299630	194103	M	41,400				41,400
2	3,628	177442	124429	M	24,517				24,517
3	4,141	246675	145537	M	34,083				34,083
Total		723747	464070		100,000				100,000

Benzoic acid (31b)[16], (synthesized from 4-bromobenzoic acid, white solid, isolated yield 80% (40 mg), GC yield 85%)

^{1}H NMR (400 MHz, $CDCl_3$) δ 8.19 – 8.07 (m, 2H), 7.66 – 7.57 (m, 1H), 7.48 (t, J = 7.8 Hz, 2H) ppm.

^{13}C{1H} NMR (101 MHz, $CDCl_3$) δ 172.3, 133.8, 130.2, 129.3, 128.5 ppm.

GC chromatogram of reaction mixture:

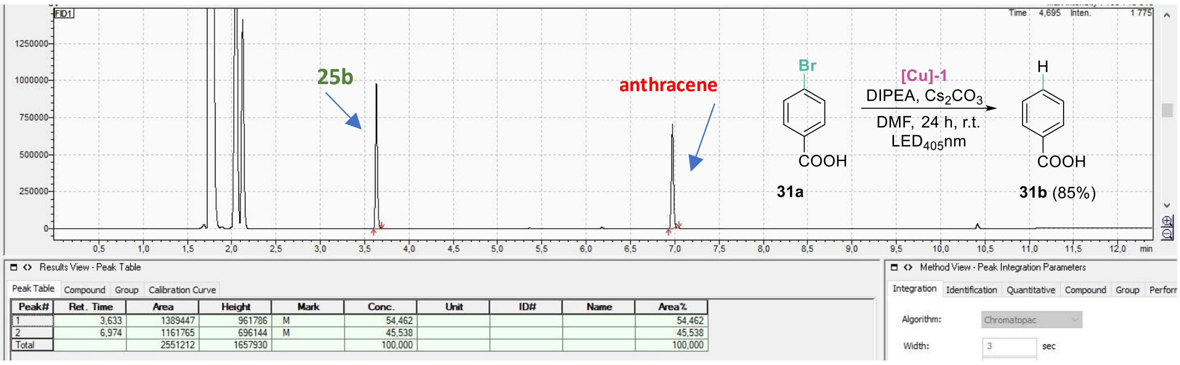

Benzaldehyde (32b), (synthesized from 4-bromobenzaldehyde, GC yield = 40%, full substrate conversion, too volatile to isolate at the scale 0.25 mmol without any loss, GC yield established from three calibration points with commercial benzaldehyde and dodecane as standard).

GC chromatogram of reaction mixture:

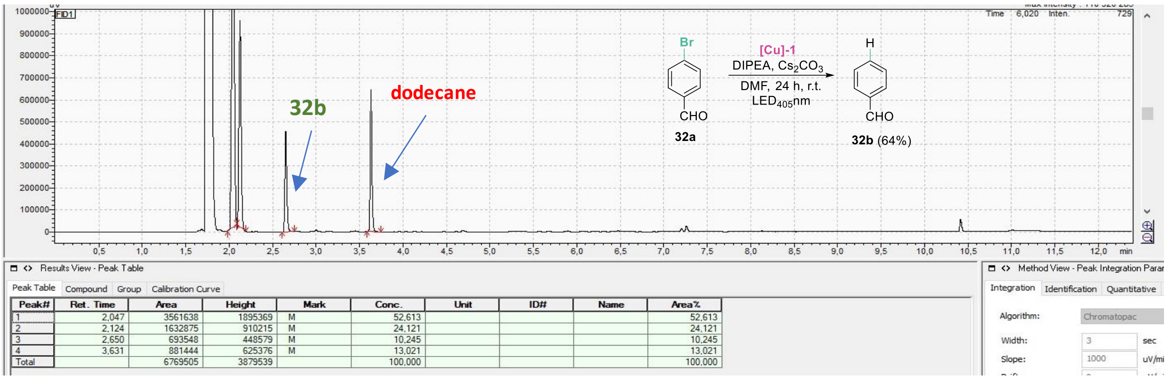

Benzonitrile (33b), (synthesized from 4-bromobenzonitrile, GC yield = 58%, full substrate conversion, too volatile to isolate at the scale 0.25 mmol without any loss, GC yield established from three calibration points with commercial benzonitrile and dodecane as standard).

GC chromatogram of reaction mixture:

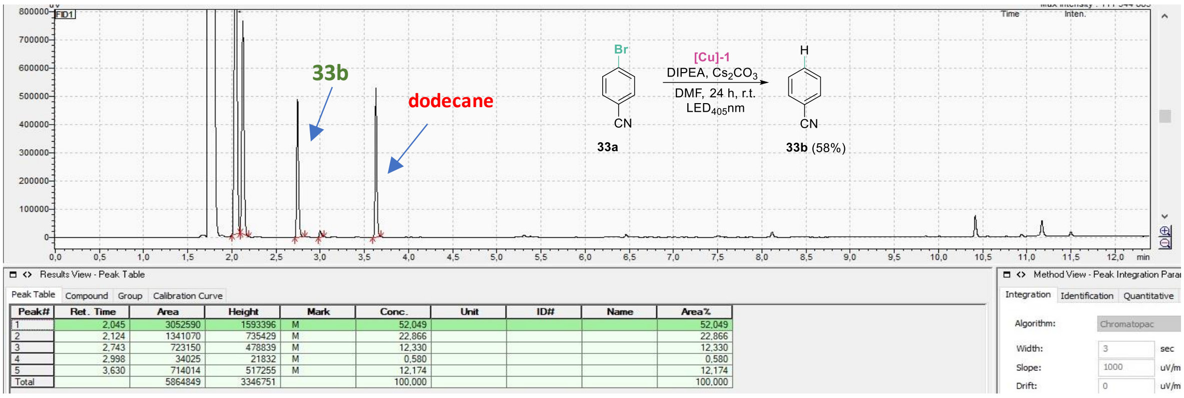

9H-fluorene (34b)[17], (synthesized from 9-bromo-9H-fluorene, white solid, isolated yield 79%, 33 mg, GC yield 85%)

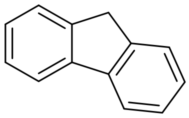

^{1}H NMR (400 MHz, $CDCl_3$) δ 7.83 (dd, *J* = 7.5, 1.2 Hz, 1H), 7.58 (dt, *J* = 7.4, 1.0 Hz, 1H), 7.41 (td, *J* = 7.5, 1.1 Hz, 1H), 7.34 (td, J = 7.4, 1.3 Hz, 1H), 3.93 (s, 1H) ppm.

^{13}C{1H} NMR (101 MHz, $CDCl_3$) δ 143.3, 141.8, 126.8, 126.7, 125.1, 119.9, 36.9 ppm.

GC chromatogram of reaction mixture:

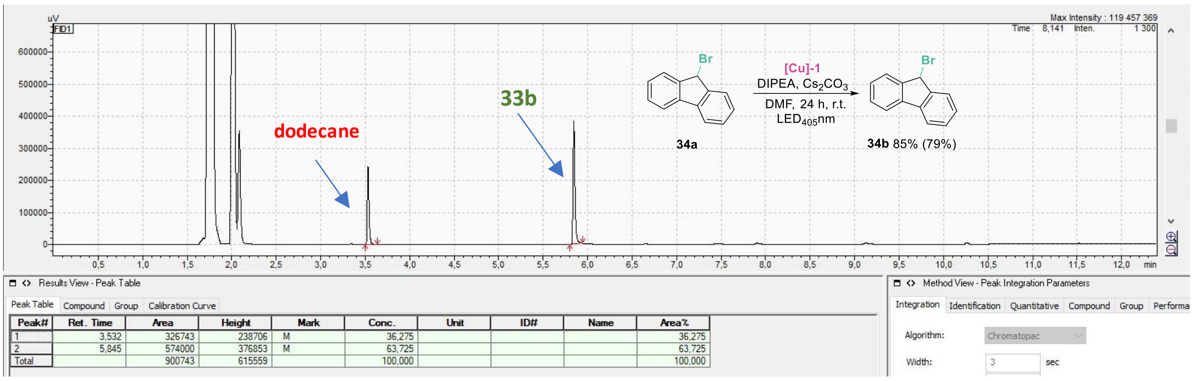

Peak#	Ret. Time	Area	Height	Mark	Conc.	Unit	ID#	Name	Area%
1	3,532	326743	238706	M	36,275				36,275
2	5,845	574000	376853	M	63,725				63,725
Total		900743	615559		100,000				100,000

Naphthalene (35b)[18], (synthesized from 1-bromonaphthalene, white solid, isolated yield 50%, 16 mg)

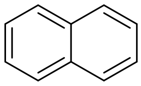

^{1}H NMR (400 MHz, $CDCl_3$) δ 8.03 – 7.77 (m, 1H), 7.71 – 7.45 (m, 1H) ppm.

^{13}C{1H} NMR (101 MHz, $CDCl_3$) δ 133.5, 127.9, 125.8 ppm.

Anthracene (36b)[19], (synthesized from 9-bromoanthracene, white solid, isolated yield 45%, 20 mg)

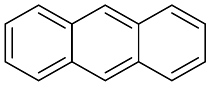

^{1}H NMR (400 MHz, $CDCl_3$) δ 8.44 (s, 1H), 8.01 (dt, *J* = 6.0, 3.0 Hz, 2H), 7.52 – 7.41 (m, 2H) ppm.

^{13}C{1H} NMR (101 MHz, $CDCl_3$) δ 131.7, 128.2, 126.2, 125.3 ppm.

Indole (37b)[20], (synthesized from 5-iodo-1H-indole, white solid, isolated yield 38%, 11 mg, GC yield 45%)

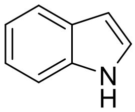

^{1}H NMR (400 MHz, $CDCl_3$) δ 8.12 (s, 1H), 7.69 – 7.61 (m, 1H), 7.40 (dq, *J* = 8.0, 0.9 Hz, 1H), 7.23 – 7.17 (m, 2H), 7.12 (ddd, *J* = 8.1, 7.1, 1.1 Hz, 1H), 6.57 (ddd, *J* = 3.1, 2.0, 1.0 Hz, 1H) ppm.

^{13}C{1H} NMR (101 MHz, $CDCl_3$) δ 127.9, 124.1, 122.0, 120.7, 119.8, 110.9, 102.6 ppm.

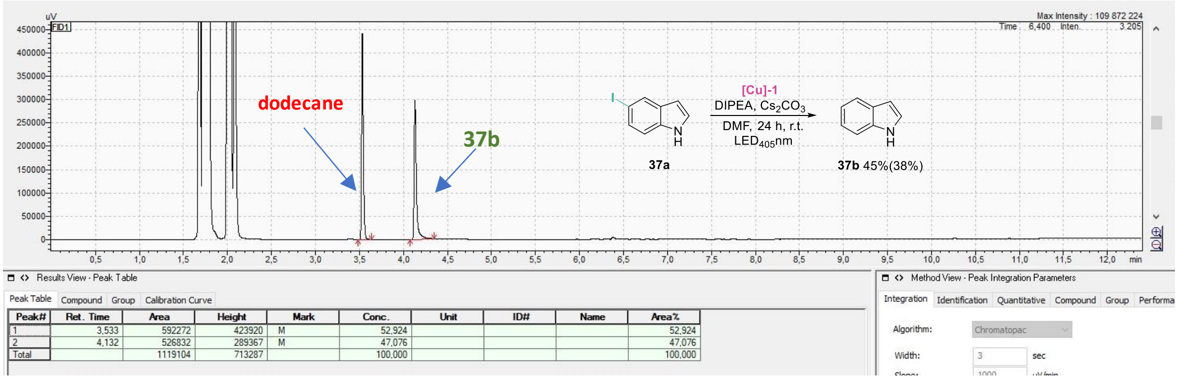

Peak#	Ret. Time	Area	Height	Mark	Conc.	Unit	ID#	Name	Area%
1	3,533	592272	423920	M	52,924				52,924
2	4,132	526832	289367	M	47,076				47,076
Total		1119104	713287		100,000				100,000

Isoquinoline (38b)[21], (synthesized from 6-bromoisoquinoline, yellowish oil 35%, 11 mg, GC yield 41%)

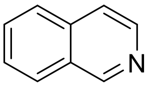

^{1}H NMR (400 MHz, $CDCl_3$) δ δ 9.24 (d, *J* = 1.0 Hz, 1H), 8.52 (d, *J* = 5.8 Hz, 1H), 7.95 (dq, *J* = 8.2, 1.0 Hz, 1H), 7.80 (dq, *J* = 8.2, 1.0 Hz, 1H), 7.67 (ddd, *J* = 8.2, 6.9, 1.3 Hz, 1H), 7.62 (dd, *J* = 5.7, 1.1 Hz, 1H), 7.58 (ddd, *J* = 8.2, 6.8, 1.2 Hz, 1H) ppm.
^{13}C{1H} NMR (101 MHz, $CDCl_3$) δ 152.5, 143.0, 135.8, 130.3, 128.7, 127.6, 127.2, 126.5, 120.4 ppm.

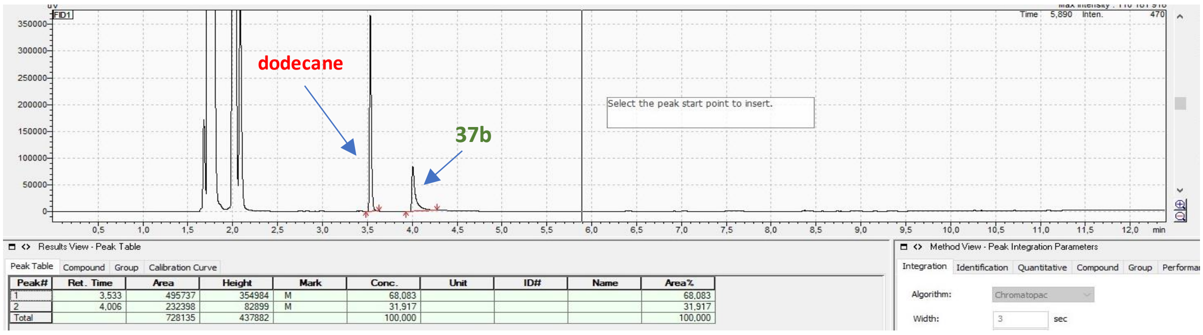

Peak#	Ret. Time	Area	Height	Mark	Conc.	Unit	ID#	Name	Area%
1	3,533	495737	354984	M	68,083				68,083
2	4,006	232398	82899	M	31,917				31,917
Total		728135	437882		100,000				100,000

Pyridine (39b), (synthesized from 2-bromopyridine, GC yield = 35%, too volatile to isolate at the scale 0.25 mmol without any loss, GC yield established from three calibration points with commercial pyridine and dodecane as standard).
GC chromatogram of reaction mixture:

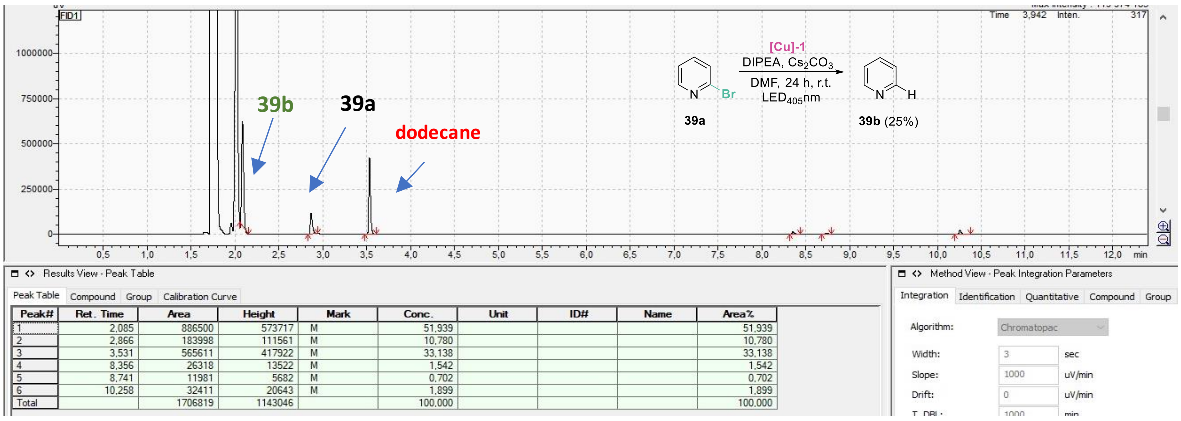

Peak#	Ret. Time	Area	Height	Mark	Conc.	Unit	ID#	Name	Area%
1	2,085	886500	573717	M	51,939				51,939
2	2,866	183998	111561	M	10,780				10,780
3	3,531	565611	417922	M	33,138				33,138
4	8,356	26318	13522	M	1,542				1,542
5	8,741	11981	5682	M	0,702				0,702
6	10,258	32411	20643	M	1,899				1,899
Total		1706819	1143046		100,000				100,000

4.2.4. Mechanistic studies – quenching experiment

The samples for Stern-Volmer analysis were prepared in a quartz screw-top cuvette equipped with a PTFE/silicone septum. The concentration of [Cu]-1 was experimentally selected as 5 µM so its absorbance at the excitation wavelength (370 nm) was around 0.2. The experiments were conducted for DCM solutions. The solvent was degassed by freeze-pump-thaw technique. DCM (2 mL for each sample) and the quencher were transferred to a cuvette under Ar atmosphere. The quenchers were added in 3 portions (10, 30 and 50 equivalents of quencher: 4-bromobenzonitrile. After each addition of the quencher, both the optical absorption and emission spectra were recorded to ensure no significant increase of absorption at excitation wavelength (370 nm) occurs.

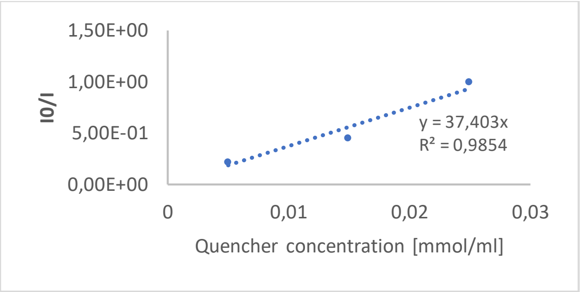

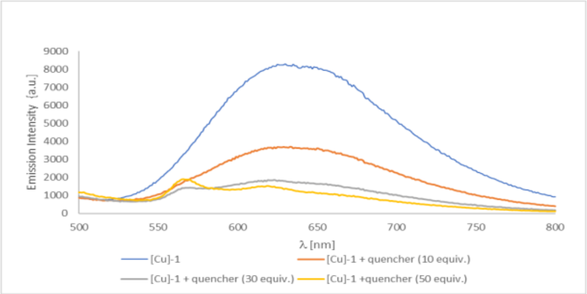

Conclusions: The Stern-Volmer analysis confirmed the interaction between excited-state [Cu]-1 catalyst and 4-bromobenzonitrile.

4.2.5. Deuterium labeling studies

In order to establish the source of H in the product structure the reaction od dehalogenation in DMF-d^7 was performed (DMF-d^7 as the deuterium source). As the model substrate methyl 4-iodobenzoate was chosen as it yielded 99% of dehalogenated product.

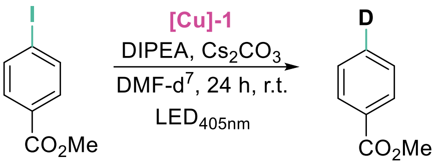

In a 5 mL snap vial with magnetic stirring bar the respective aryl halide (0.25 mmol, 1 equiv.) and photocatalyst **[Cu]-1** (5 mol%) were dissolved in dry DMF-d^7 (2 mL) and the resulting mixture was degassed argon via a syringe needle. DIPEA (2 equiv.) and Cs_2CO_3 (1.1 equiv.) were added under Ar atmosphere and the reaction mixture was irradiated through the plane bottom side of the snap vial using a 405 nm LED (UOSlab photoreactor). The reaction was stirred under light irradiation for 24 hours. After this time NMR spectrum (400 Hz) was recorded. At the same time NMR (400 Hz) spectrum in $CDCl_3$ for the reaction in DMF was recorded.

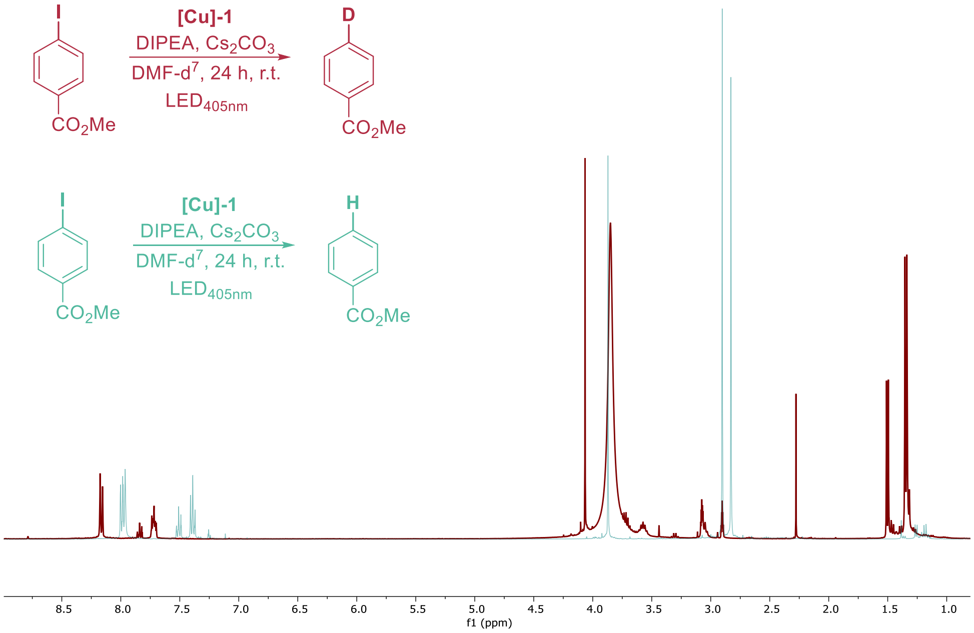

Reported NMR of methyl benzoate-4-d and methyl benzoate:[22]

benzoate-4-d: ^{1}**H NMR** of (500 MHz, CD_3CN) δ 7.97 (m, 2H), 7.48 (m, 2H), 3.84 (s, 3H) ppm;

methyl benzoate: ^{1}**H NMR** (500 MHz, CDCl3) δ 7.88 (m, 2H), 7.45 (m, 1H), 7.32 (m, 2H), 3.77 (s, 3H) ppm;

Conclusion: after comparison of two reactions (one in DMF and the other one in DMF-d^7) we conclude that the H in dehalogenation processes most likely comes from the solvent.

4.2.6. Mechanistic proposal

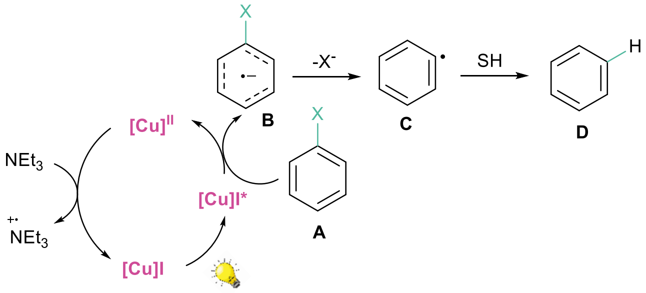

Based on the aforementioned results and the literature data, we propose a plausible radical reaction pathway for the photoreduction of aryl halides under the action of **[Cu]-1** photocatalyst. In this approach, the first event is photoexcitation of the [**Cu]-1** to its excited state NHC-Cu(I)* which reduces aromatic halide **A** (for quenching experiment see S4.2.4) to radical anion **B**, which after dehalogenation forms aryl radical **C**, which is ready readily reactions with the solvent (H-donor) to form reduced product **D**. At the same time, the photocatalyst is regenerated by a sacrificial reductant (TEA).

1. References

1 R. Marion, F. Sguerra, F. Di Meo, E. Sauvageot, J. F. Lohier, R. Daniellou, J. L. Renaud, M. Linares, M. Hamel and S. Gaillard, *Inorg. Chem.*, 2014, **53**, 9181–9191.

2 S. Wagaw and S. L. Buchwald, *J. Org. Chem.*, 1996, **61**, 7240–7241.

3 M. Elie, F. Sguerra, F. Di Meo, M. D. Weber, R. Marion, A. Grimault, J. F. Lohier, A. Stallivieri, A. Brosseau, R. B. Pansu, J. L. Renaud, M. Linares, M. Hamel, R. D. Costa and S. Gaillard, *ACS Appl. Mater. Interfaces*, 2016, **8**, 14678–14691.

4 M. Ruthkosky, F. N. Castellano and G. J. Meyer, *Photodriven Electron and Energy Transfer from Copper Phenanthroline Excited States*, Academic Press, 1996, vol. 99.

5 S. Díez-González, N. M. Scott and S. P. Nolan, *Organometallics*, 2006, **25**, 2355–2358.

6 A. Banerjee, S. Sarkar, J. A. Shah, N. C. Frederiks, E. A. Bazan-Bergamino, C. J. Johnson and M. Y. Ngai, *Angew. Chemie - Int. Ed.*, 2022, **61**, 1–7.

7 B. N. Li, Y. Y. Liu, Y. P. Wang and M. Pan, *Molecules*, , DOI:10.3390/molecules26113244.

8 C. Wang, L. Lystrom, H. Yin, M. Hetu, S. Kilina, S. A. McFarland and W. Sun, *Dalt. Trans.*, 2016, **45**, 16366–16378.

9 G. Meng and M. Szostak, *Angew. Chemie*, 2015, **127**, 14726–14730.

10 M. Das, A. Manvar, M. Jacolot, M. Blangetti, R. C. Jones and D. F. O'Shea, *Chem. - A Eur. J.*, 2015, **21**, 8737–8740.

11 T. Iwasaki, Y. Miyata, R. Akimoto, Y. Fujii, H. Kuniyasu and N. Kambe, *J. Am. Chem. Soc.* 2014, **136**, 9260–9263.

12 Frisch, M. J.; Trucks, G. W.; Schlegel, H. B.; Scuseria, G. E.; Robb, M. A.; Cheeseman, J. R.; Scalmani, G.; Barone, V.; Petersson, G. A.; Nakatsuji, H.; Li, X.; Caricato, M.; Marenich, A. V.; Bloino, J.; Janesko, B. G.; Gomperts, R.; Mennucci, B.; Hratchian, H. P.; Ortiz, J. V.; Izmaylov, A. F.; Sonnenberg, J. L.; Williams-Young, D.; Ding, F.; Lipparini, F.; Egidi, F.; Goings, J.; Peng, B.; Petrone, A.; Henderson, T.; Ranasinghe, D.; Zakrzewski, V. G.; Gao, J.; Rega, N.; Zheng, G.; Liang, W.; Hada, M.; Ehara, M.; Toyota, K.; Fukuda, R.; Hasegawa, J.; Ishida, M.; Nakajima, T.; Honda, Y.; Kitao, O.; Nakai, H.; Vreven, T.; Throssell, K.; Montgomery, J. A., Jr.; Peralta, J. E.; Ogliaro, F.; Bearpark, M. J.; Heyd, J. J.; Brothers, E. N.; Kudin, K. N.; Staroverov, V. N.; Keith, T. A.; Kobayashi, R.; Normand, J.; Raghavachari, K.; Rendell, A. P.; Burant, J. C.; Iyengar, S. S.; Tomasi, J.; Cossi, M.; Millam, J. M.; Klene, M.; Adamo, C.; Cammi, R.; Ochterski, J. W.; Martin, R. L.; Morokuma, K.; Farkas, O.; Foresman, J. B.; Fox, D. J. Gaussian 16, Revision B.01; Gaussian, Inc.: Wallingford, CT, 2016.

13 Grimme, S.; Antony, J.; Ehrlich, S.; Krieg, H. A Consistent and Accurate Ab Initio Parametrization of Density Functional Dispersion Correction (DFT-D) for the 94 Elements H-Pu. *J. Chem. Phys.* **2010**, *132* (15). https://doi.org/10.1063/1.3382344.

14 Marenich, A. V.; Cramer, C. J.; Truhlar, D. G. Universal Solvation Model Based on Solute Electron Density and on a Continuum Model of the Solvent Defined by the Bulk Dielectric Constant and Atomic Surface Tensions. *J. Phys. Chem. B* **2009**, *113* (18), 6378–6396. https://doi.org/10.1021/jp810292n.

15 CYLview20; Legault, C. Y., Université de Sherbrooke, 2020 (http://www.cylview.org)

16 Ning Xu, Xiaoqing Peng, Can Luo, Lei Huang, Chaodong Wang, Zhi Chen, Jianjun Li *Adv. Synth. Catal.* **2023**, *365*, 142-147.

17 Tengda Si, Hana Cho, Hun Young Kim, Kyungsoo Oh *Org. Lett.* **2022**, *24*, 46, 8531–8535.

18 Kuhlmann, Jan H.; Dickoff, Jan H.; Mancheño, Olga García *Chem. – Eur. J.* **2023**, *29*, E202203347.

19 Tsuruta, Takuya; Spinnato, Davide; Moon, Hye Won; Leutzsch, Markus; Cornella, Josep *J. Am. Chem. Soc.* **2023**, *145*, 47, 25538 – 25544.

20 Xu, Wei; Nagata, Yuuya; Kumagai, Naoya *J. Am. Chem. Soc.* **2023**, *145*, 2609 – 2618.

21 Fukazawa, Yasuaki; Rubtsov, Aleksandr E.; Malkov, Andrei V. *Eur. J. Org. Chem.* **2020**, *22*, 3317 – 3319.

22 Tian Luo, Zi Wang, Yinlin Chen, Hengzhao Li, Mengqi Peng, Floriana Tuna, Eric J. L. McInnes, Sarah J. Day, Jie An, Martin Schröder, Sihai Yang *Angew. Chem. Int. Ed.* **2023**, *62*, 48, e2023062.

2. NMR spectra of new isolated compounds

(*E*)-1-bromo-2-fluoro-4-styrylbenzene, **7-*E***

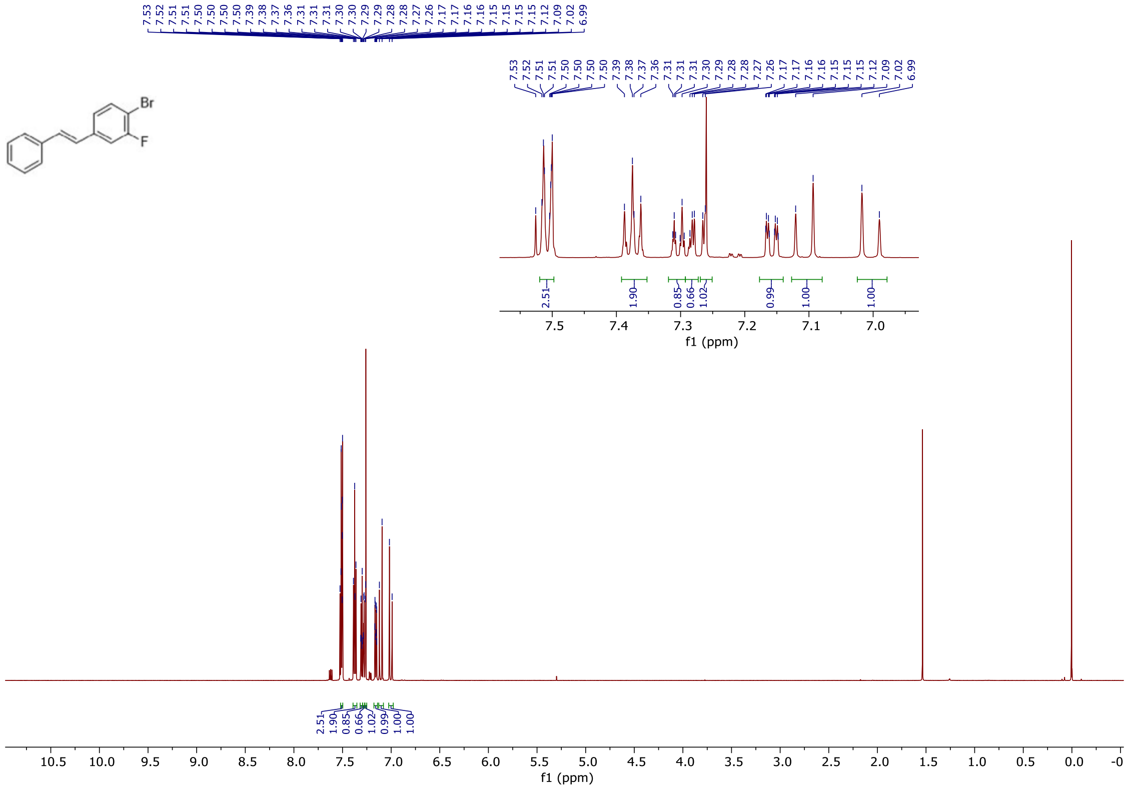

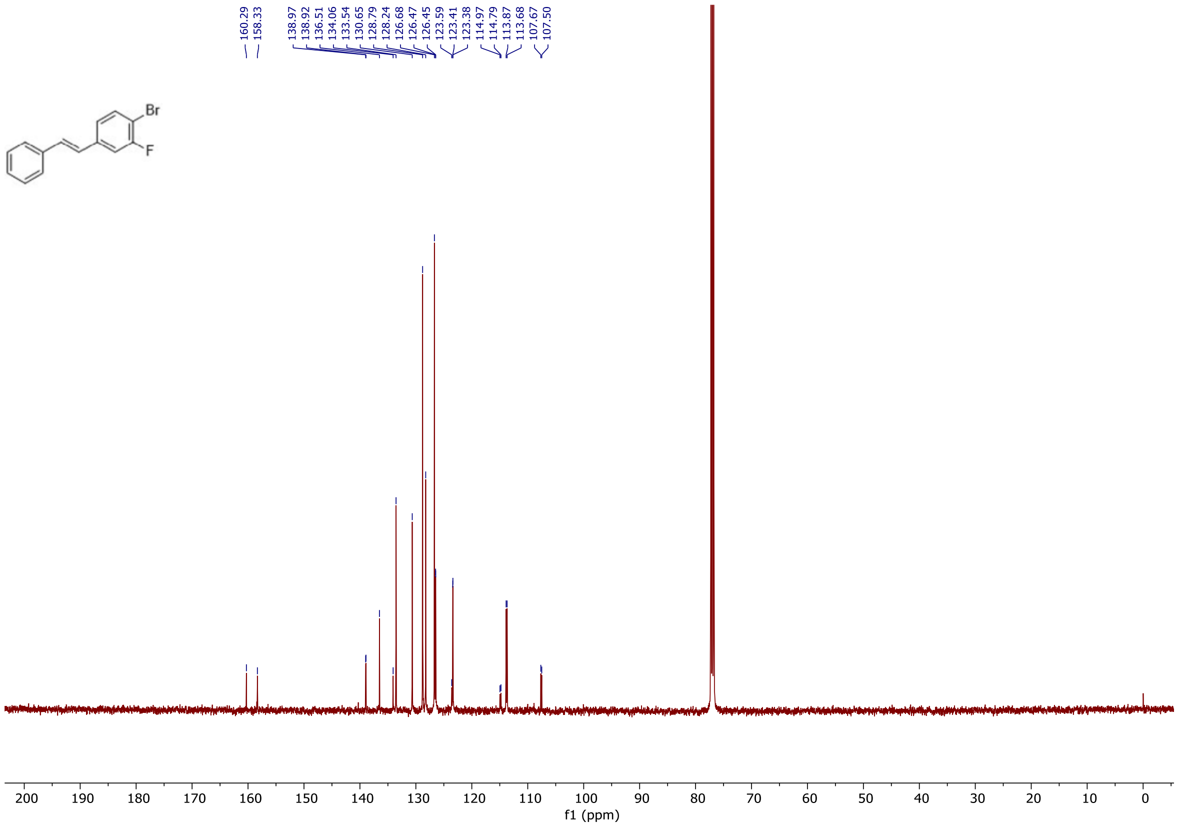

(*E*)-5-(4-bromo-3-fluorostyryl)-1,2,3-trimethoxybenzene, **10-*E***

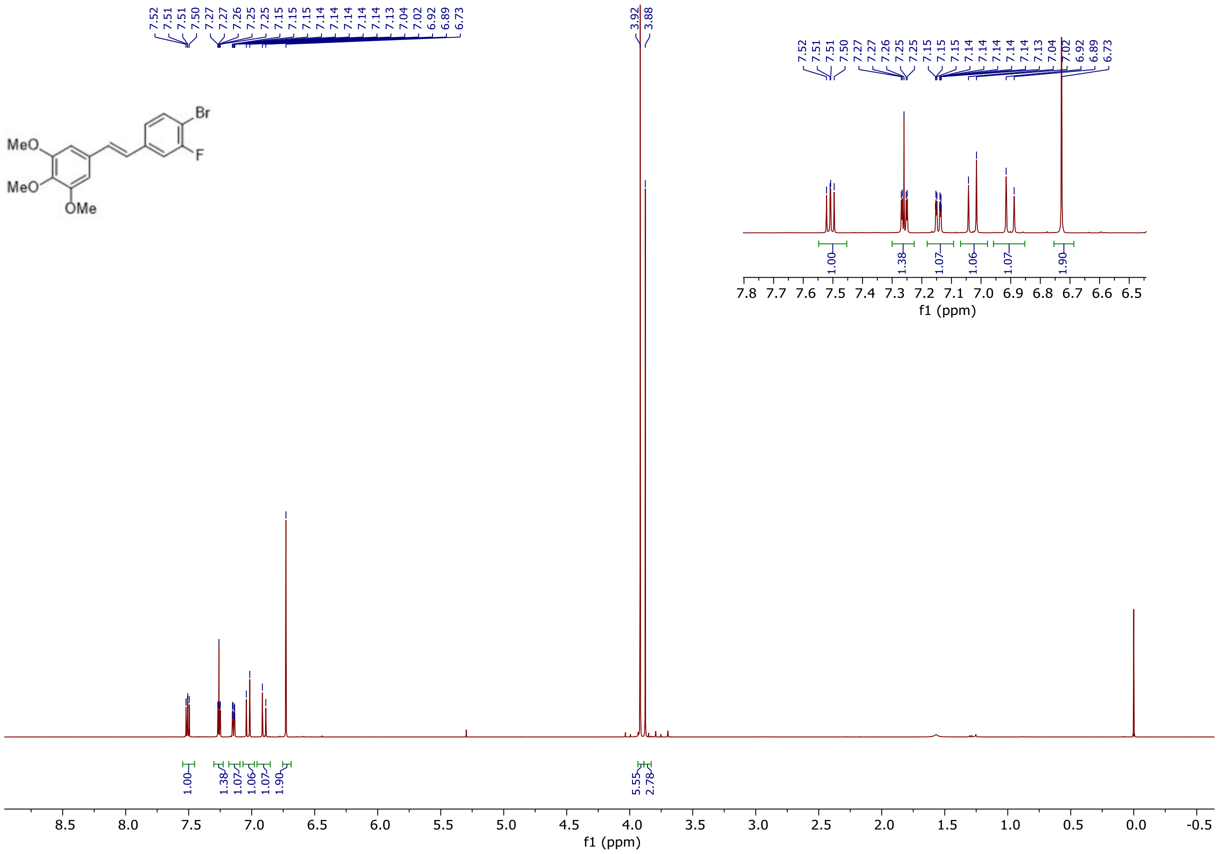

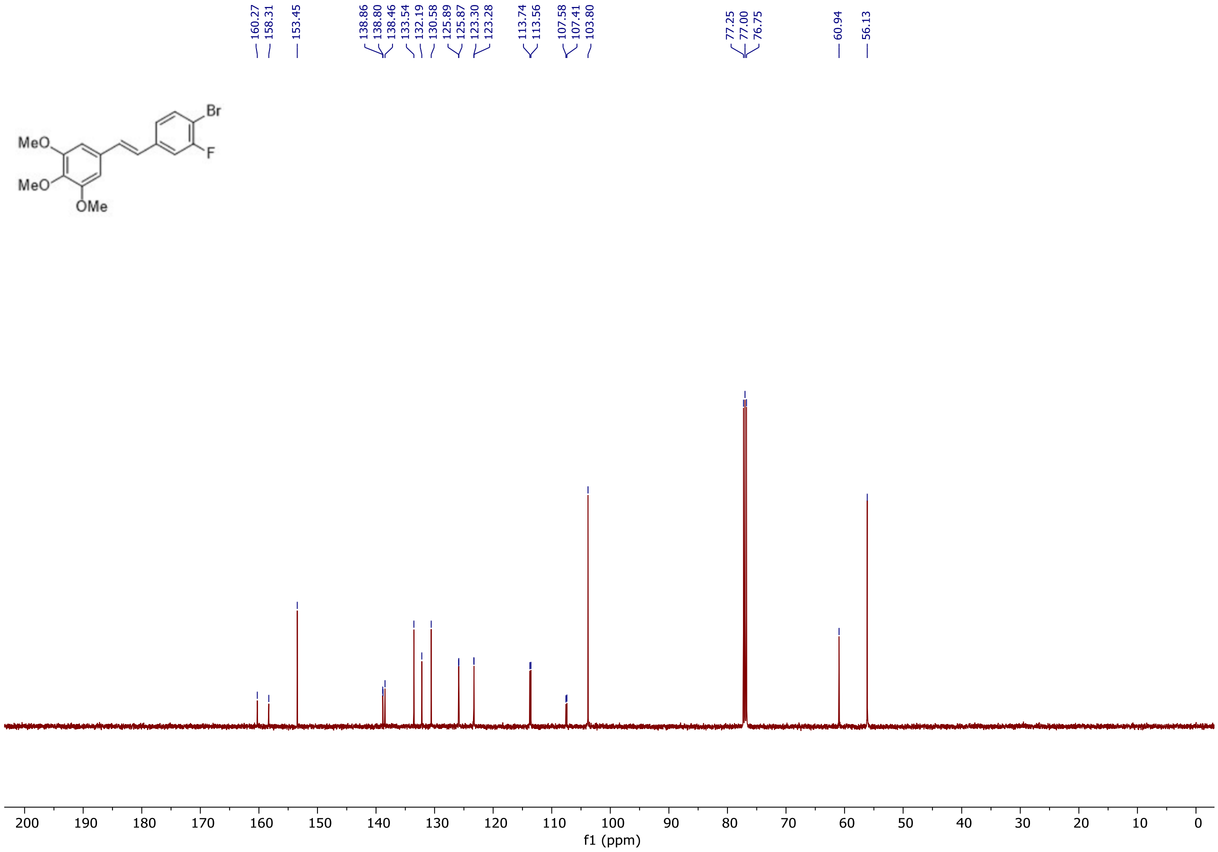

(*Z*)-1-bromo-2-fluoro-4-styrylbenzene, **7-*Z***

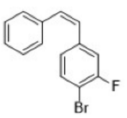

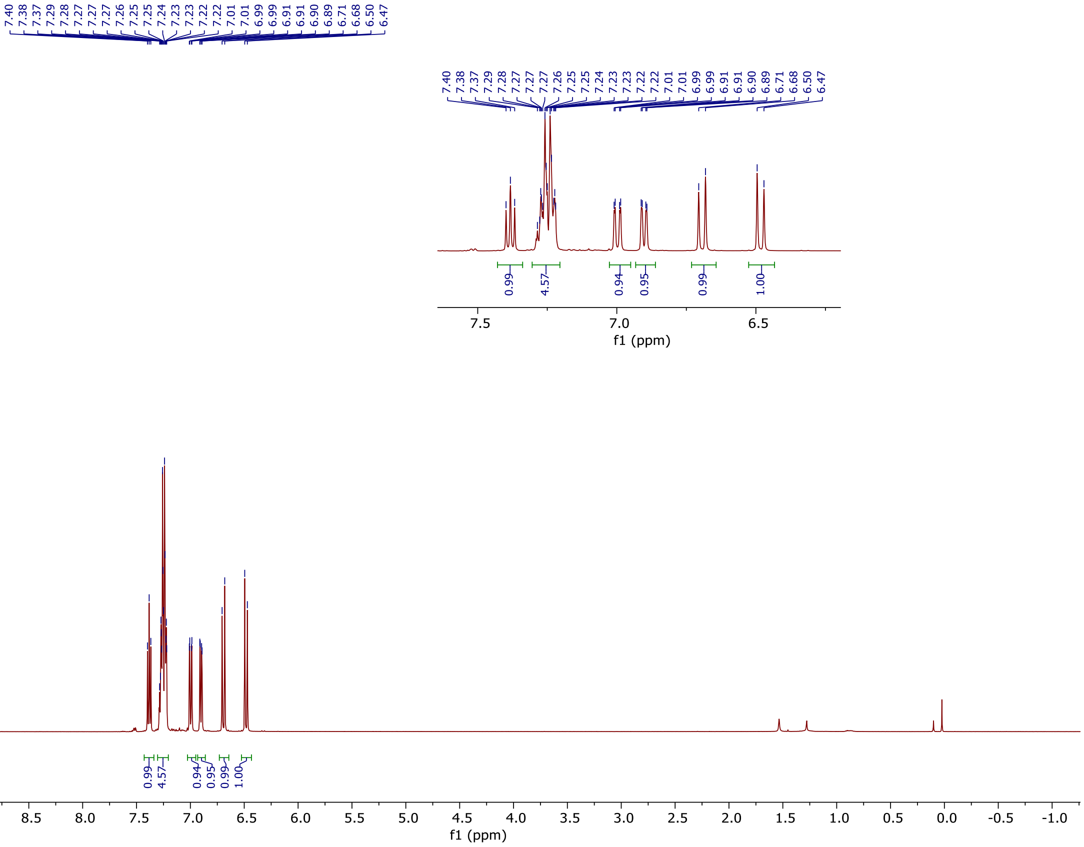

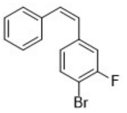

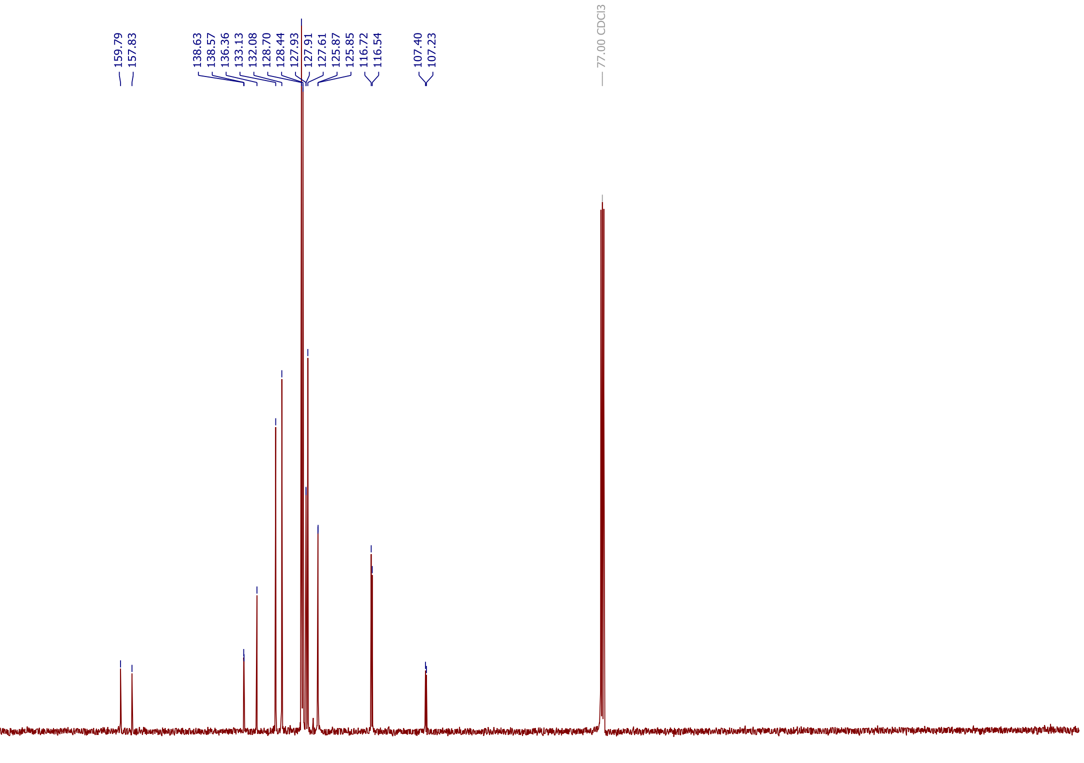

(*Z*)-5-(4-bromo-3-fluorostyryl)-1,2,3-trimethoxybenzene, **10-*Z***

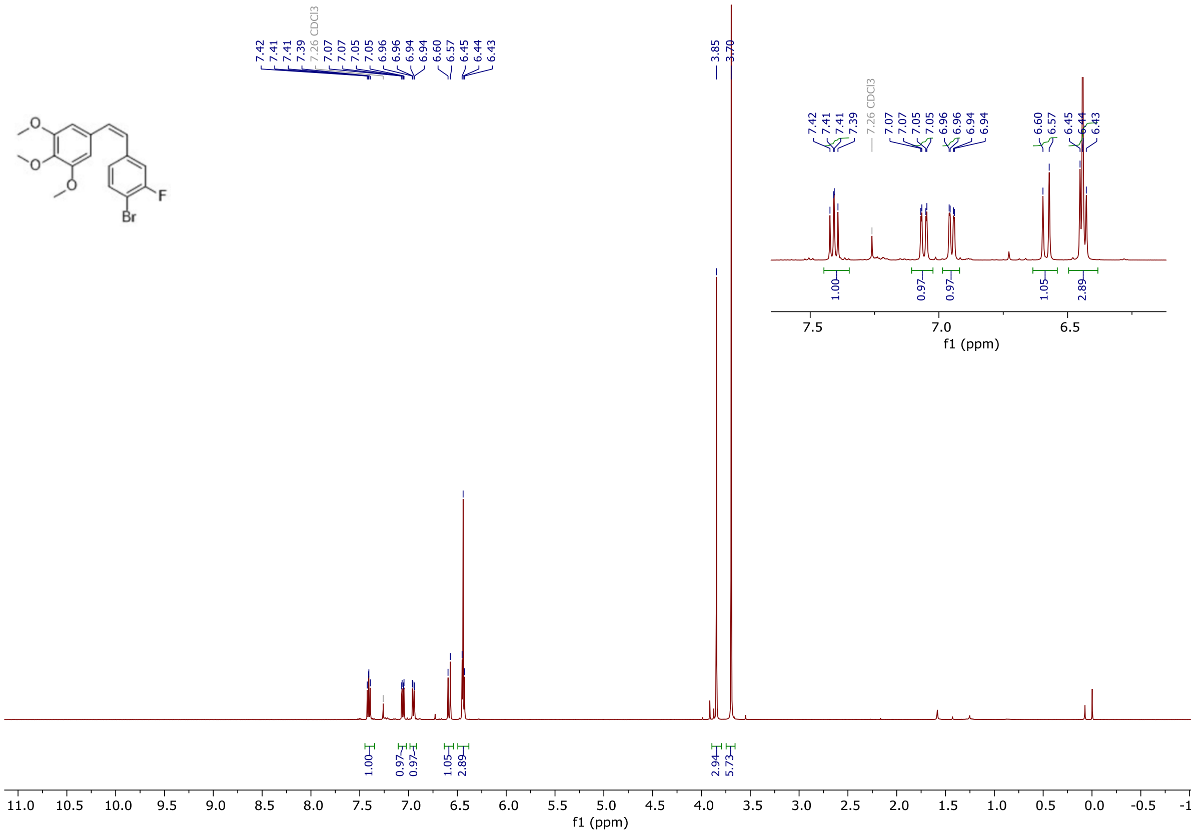

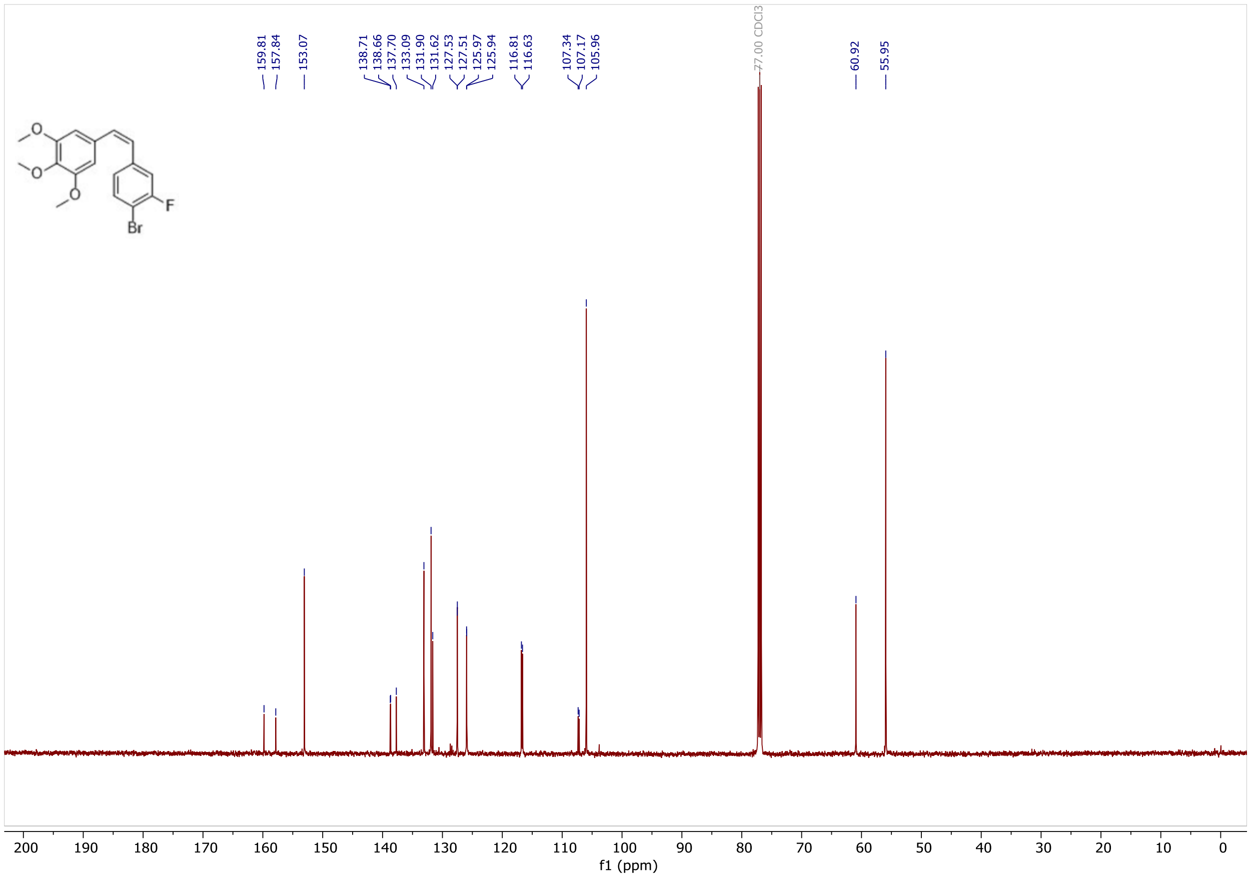

Benzoic acid (31b)

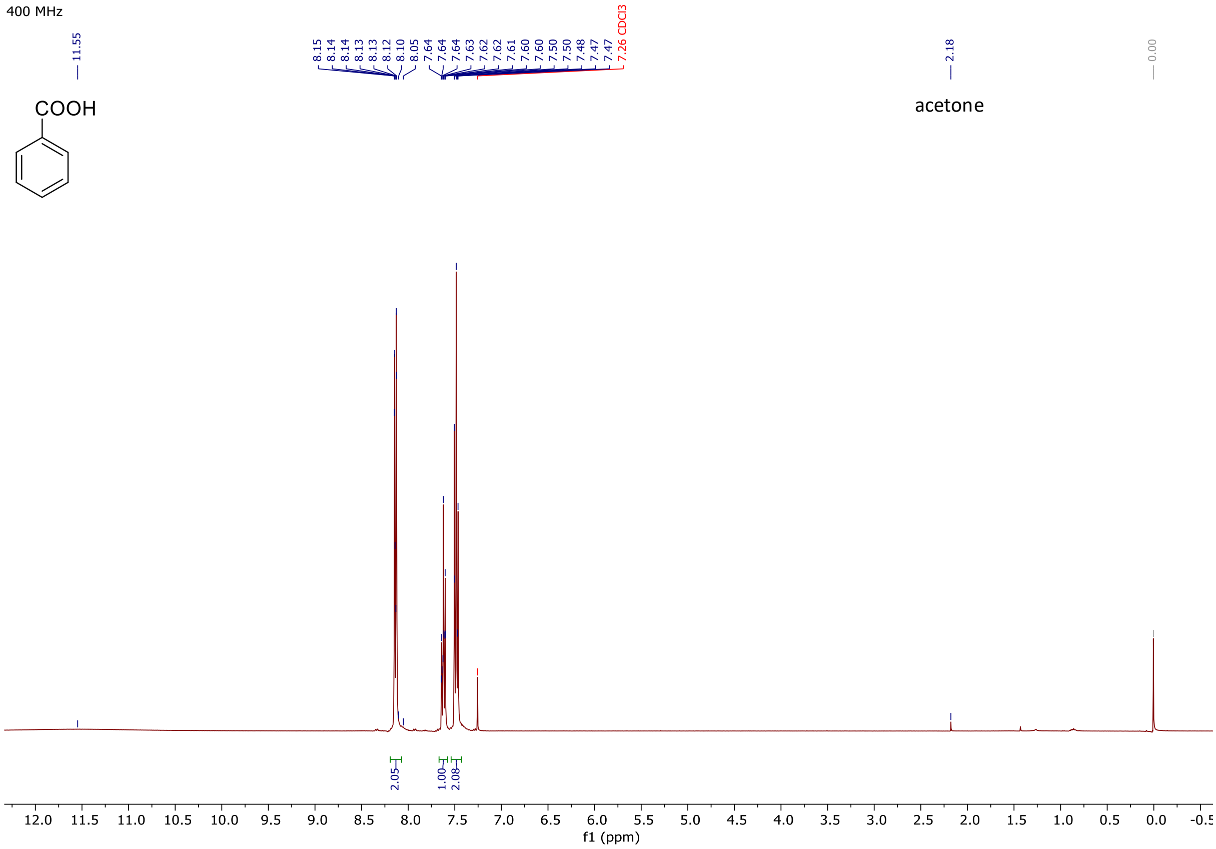

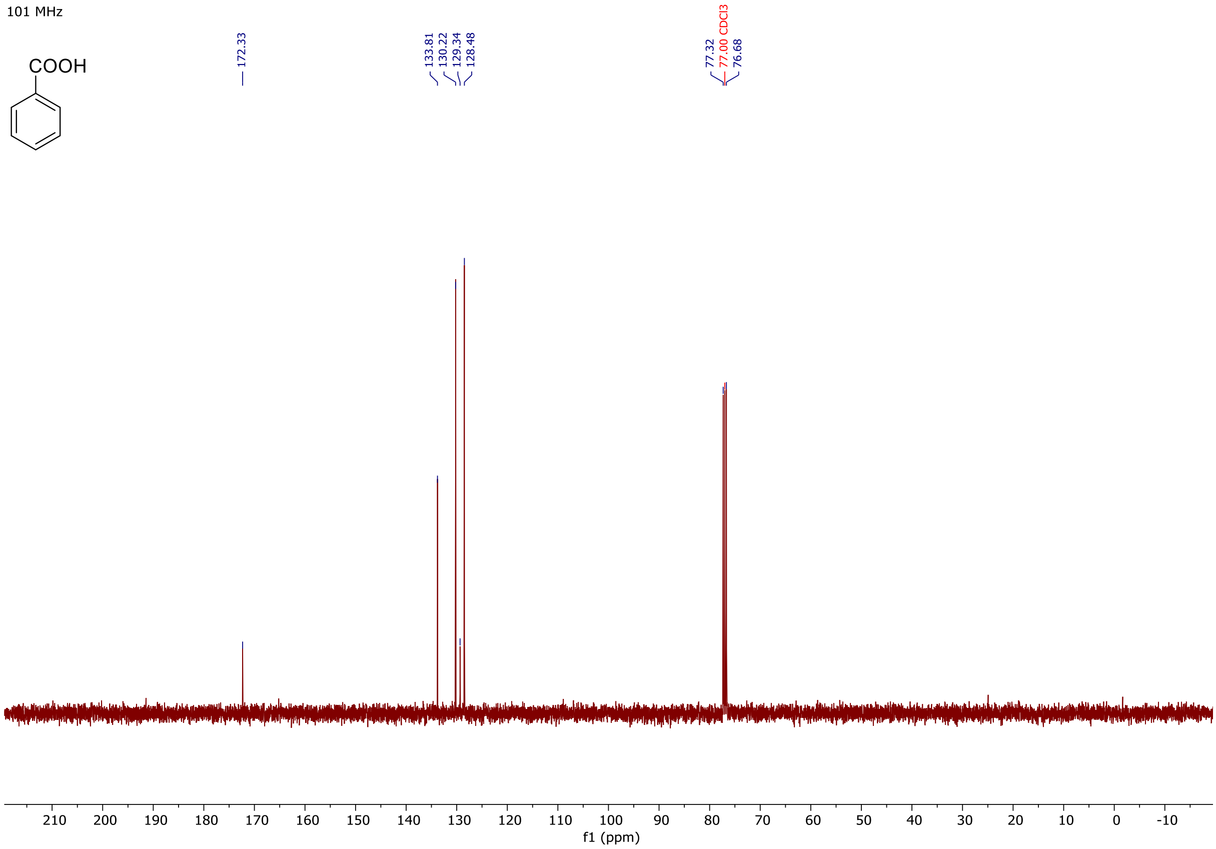

9H-fluorene (**34b**)

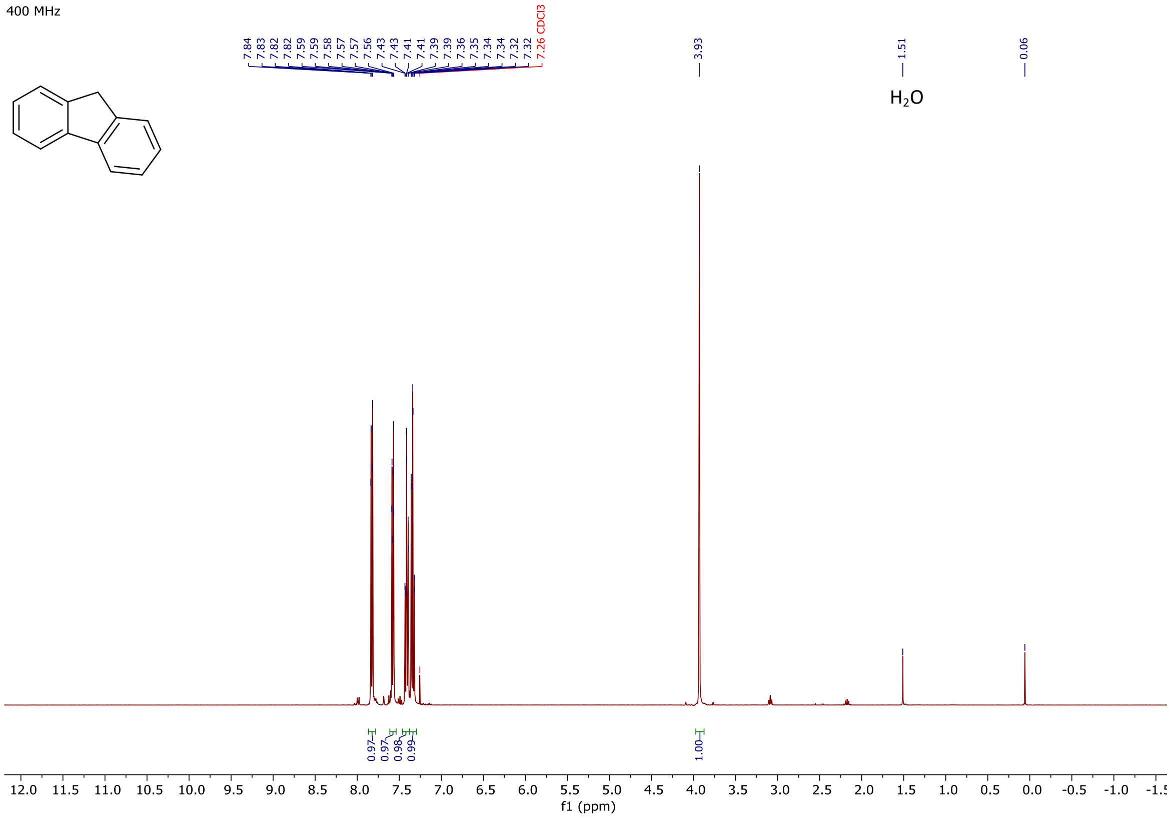

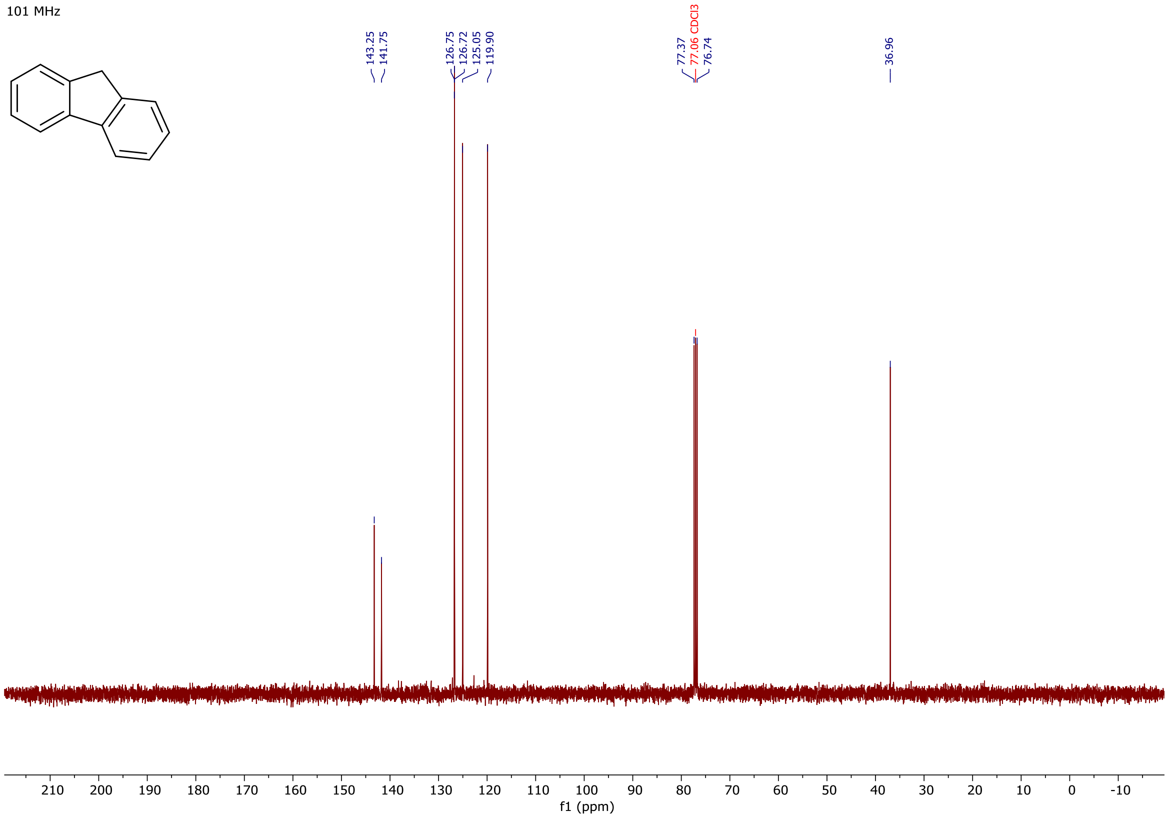

Naphthalene (35b)

400 MHz

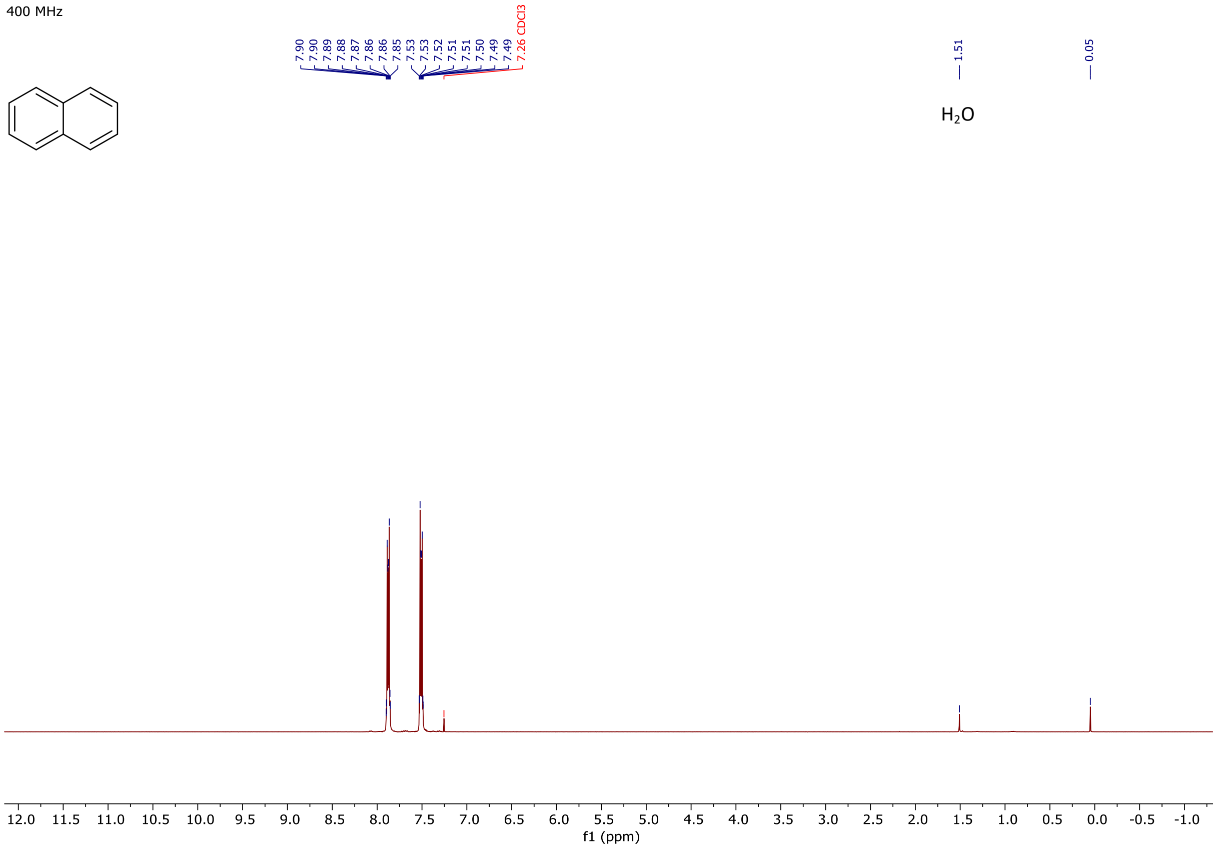

101 MHz

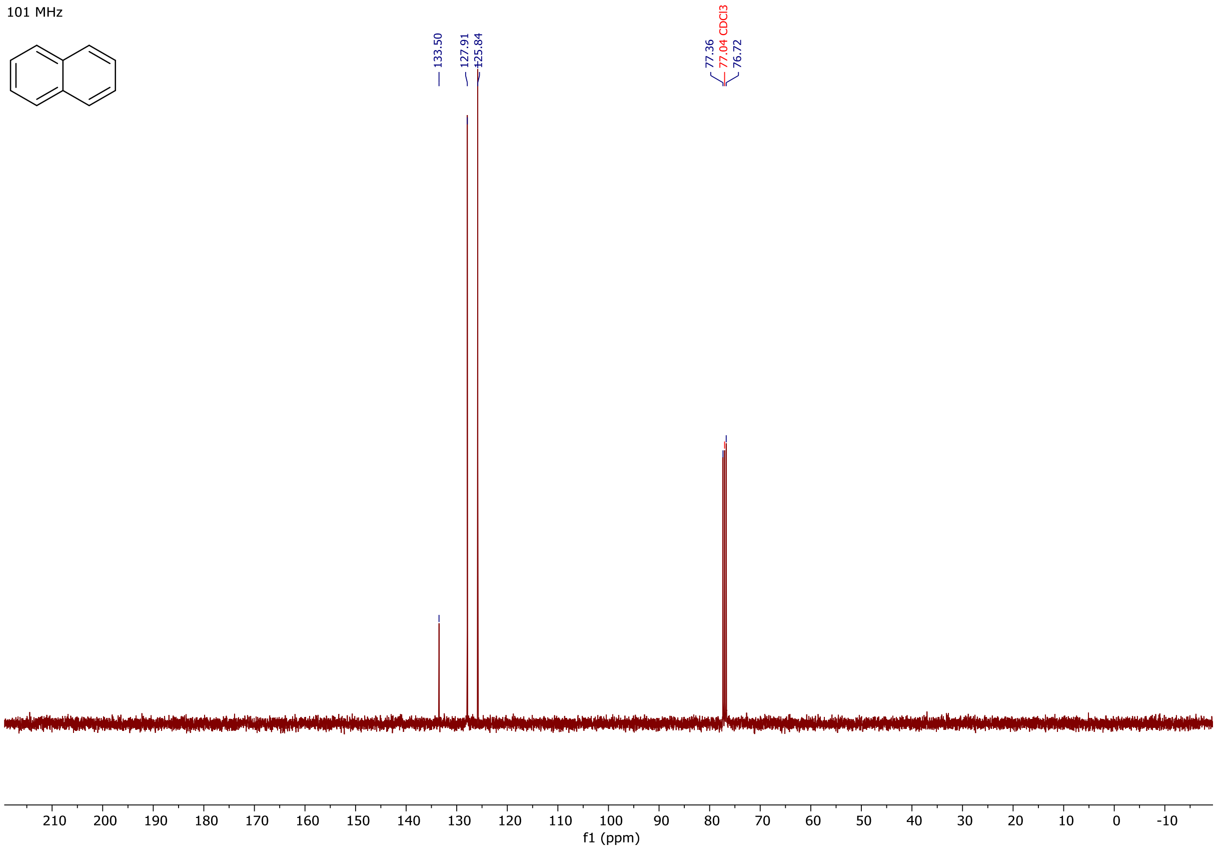

Anthracene (36b)

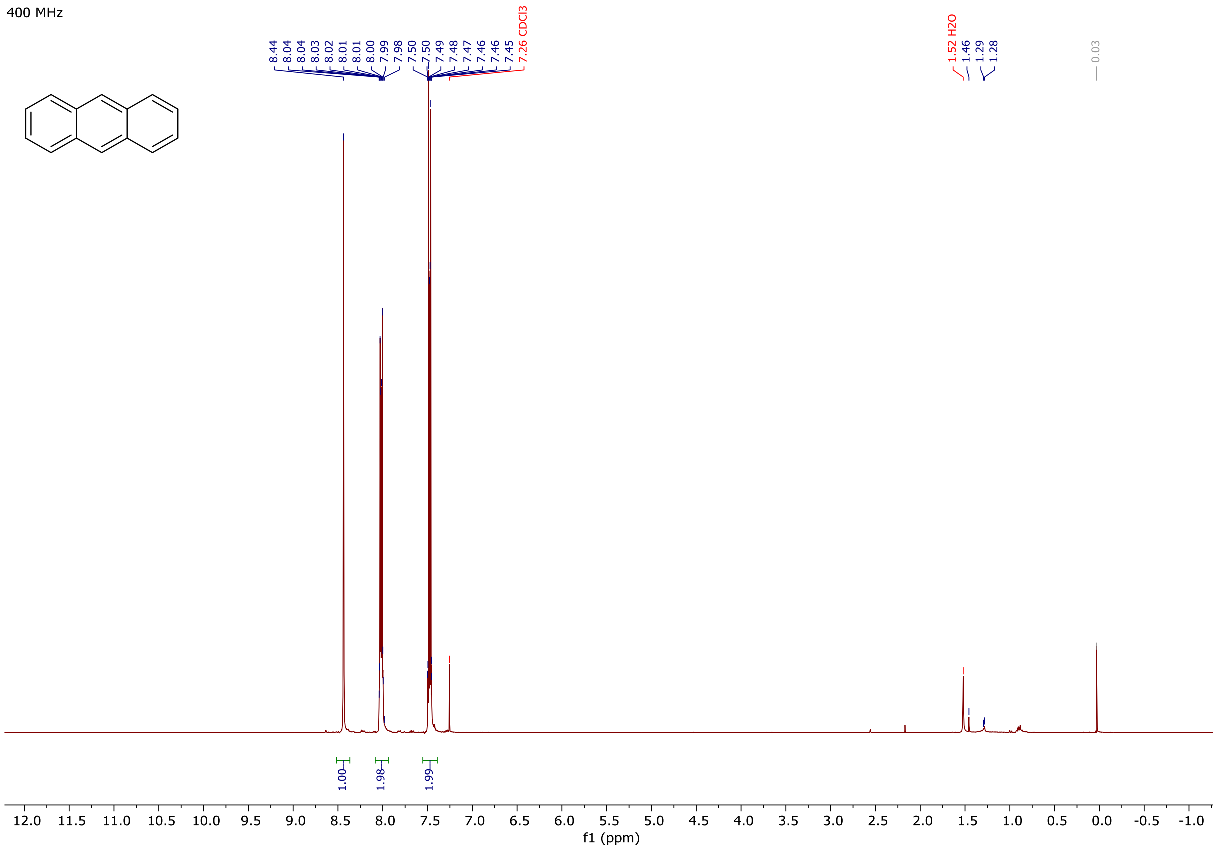

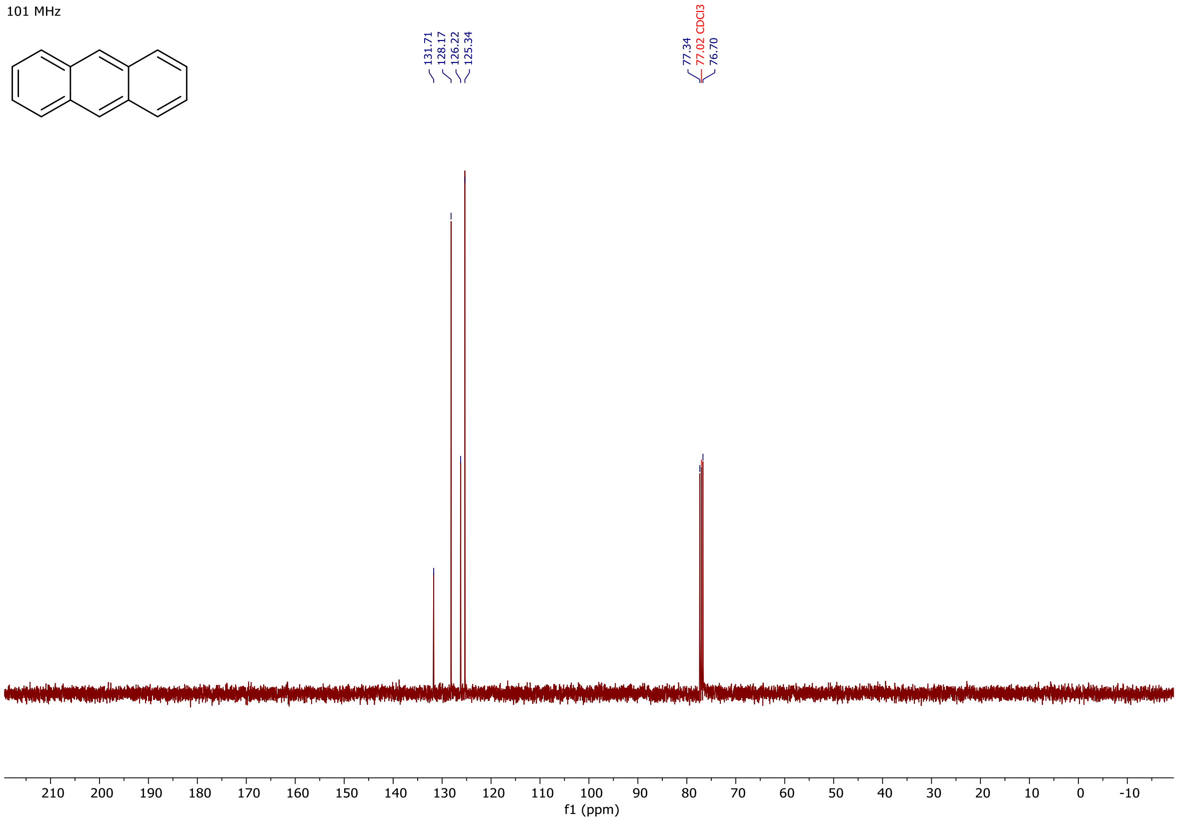

Indole (37b)

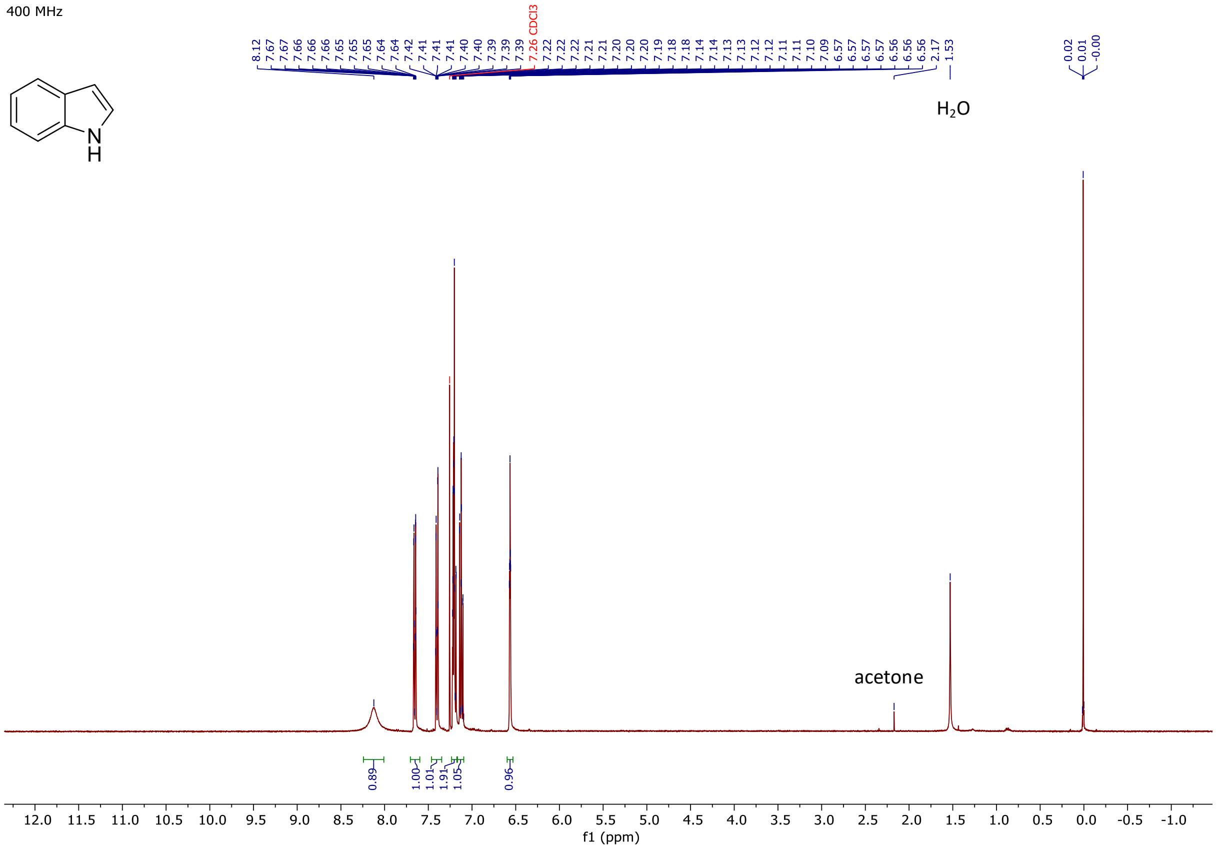
400 MHz
8.12 7.67 7.67 7.66 7.66 7.65 7.65 7.65 7.64 7.64 7.42 7.41 7.41 7.41 7.40 7.39 7.39 7.39 7.26 CDCl3 7.22 7.22 7.22 7.22 7.21 7.21 7.20 7.20 7.20 7.19 7.18 7.18 7.14 7.14 7.13 7.13 7.12 7.12 7.11 7.11 7.10 7.09 6.57 6.57 6.57 6.57 6.56 6.56 6.56 2.17 1.53
0.02 0.01 -0.00
N
H
H_2O
acetone
0.89 1.00 1.01 1.91 1.05 0.96
12.0 11.5 11.0 10.5 10.0 9.5 9.0 8.5 8.0 7.5 7.0 6.5 6.0 5.5 5.0 4.5 4.0 3.5 3.0 2.5 2.0 1.5 1.0 0.5 0.0 -0.5 -1.0
f1 (ppm)

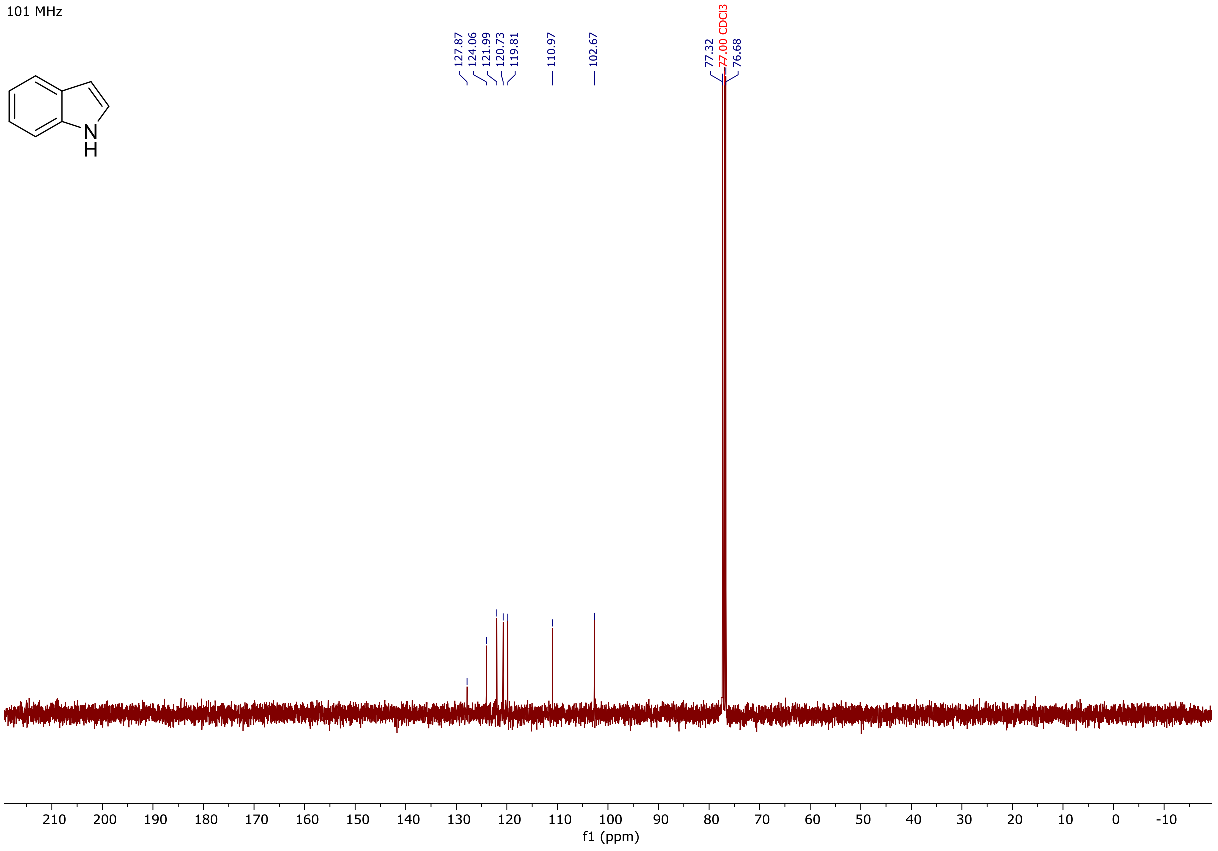
101 MHz
127.87 124.06 121.99 120.73 119.81
110.97
102.67
77.32 77.00 CDCl3 76.68
N
H
210 200 190 180 170 160 150 140 130 120 110 100 90 80 70 60 50 40 30 20 10 0 -10
f1 (ppm)

Isoquinoline (38b)

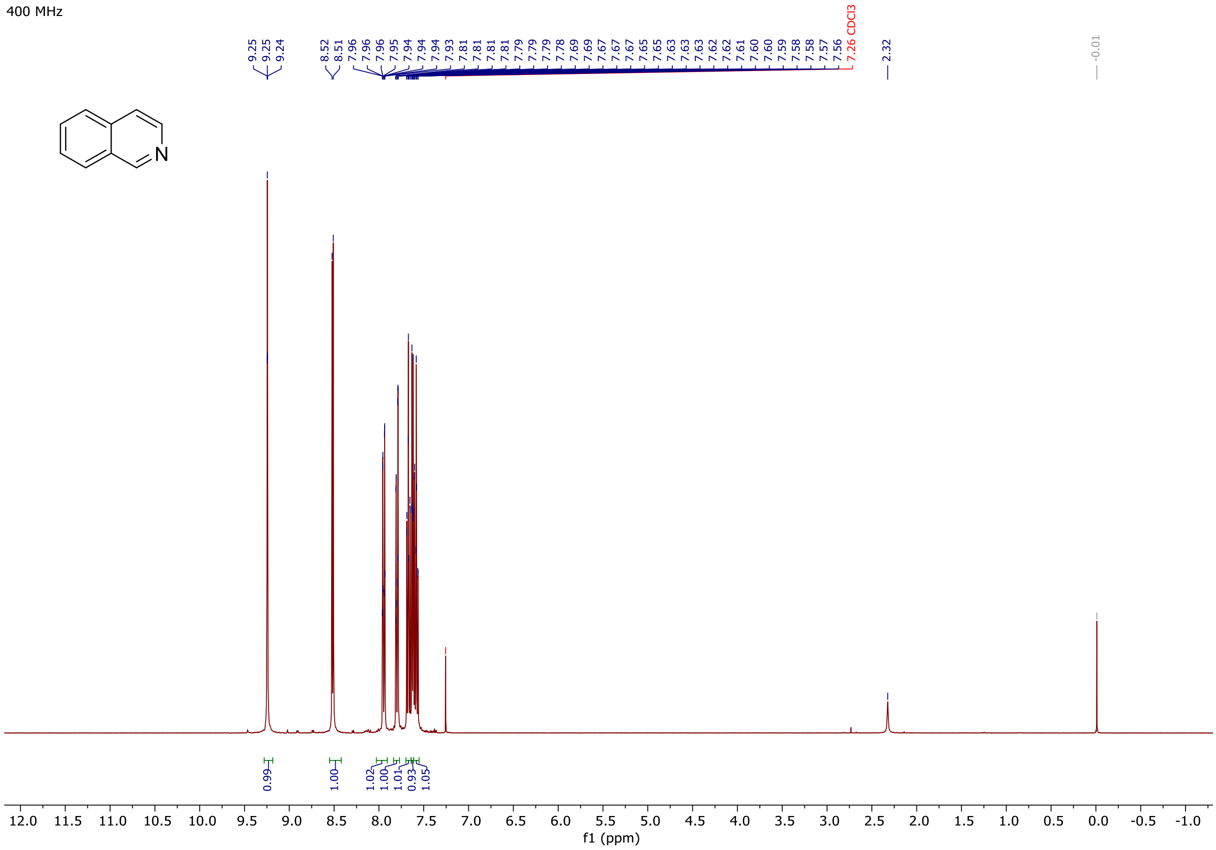

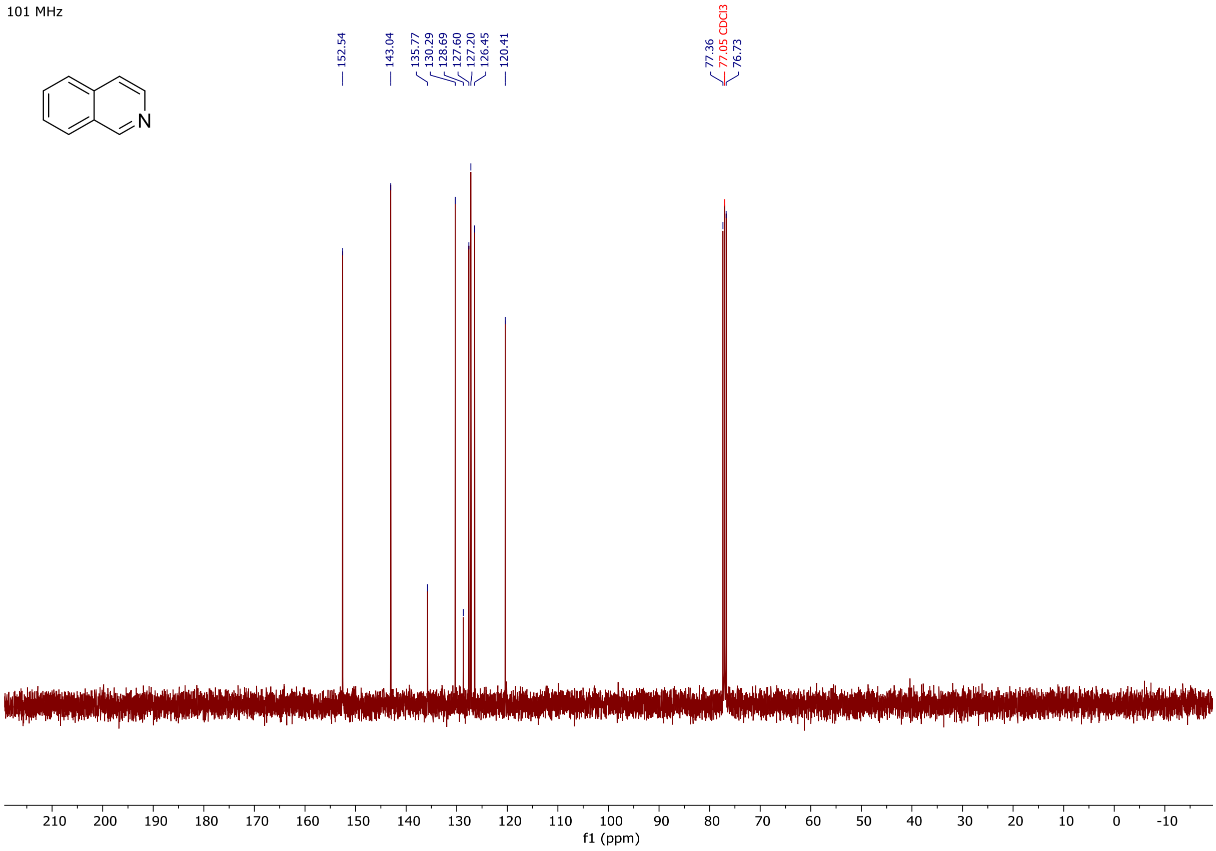

Supplement: Supplementary file 1 — jo4c00450_si_001.pdf [file jo4c00450_si_001.pdf]
